# Modulation of the Meisenheimer complex metabolism of nitro-benzothiazinones by targeted C-6 substitution

**DOI:** 10.1038/s42004-024-01235-x

**Published:** 2024-07-06

**Authors:** François Keiff, Freddy A. Bernal, Melanie Joch, Thibault J. W. Jacques dit Lapierre, Yan Li, Phil Liebing, Hans-Martin Dahse, Ivan Vilotijevic, Florian Kloss

**Affiliations:** 1 Transfer Group Anti-infectives, Leibniz Institute for Natural Product Research and Infection Biology—Leibniz-HKI, Beutenbergstr. 11a, 07745 Jena, Germany; 2 Institute for Inorganic and Analytical Chemistry, Friedrich-Schiller-Universität Jena, Humboldtstr. 8, 07743 Jena, Germany; 3 Department of Infection Biology, Leibniz Institute for Natural Product Research and Infection Biology—Leibniz-HKI, Beutenbergstr. 11a, 07745 Jena, Germany; 4 Institute of Organic Chemistry and Macromolecular Chemistry, Friedrich Schiller University Jena, Humboldtstr. 10, Jena, 07743 Germany

**Keywords:** Computational chemistry, Cheminformatics, Lead optimization, Small molecules

## Abstract

Tuberculosis, caused by *Mycobacterium tuberculosis*, remains a major public health concern, demanding new antibiotics with innovative therapeutic principles due to the emergence of resistant strains. Benzothiazinones (BTZs) have been developed to address this problem. However, an unprecedented in vivo biotransformation of BTZs to hydride-Meisenheimer complexes has recently been discovered. Herein, we present a study of the influence of electron-withdrawing groups on the propensity of HMC formation in whole cells for a series of C-6-substituted BTZs obtained through reductive fluorocarbonylation as a late-stage functionalization key step. Gibbs free energy of reaction and Mulliken charges and Fukui indices on C-5 at quantum mechanics level were found as good indicators of in vitro HMC formation propensity. These results provide a first blueprint for the evaluation of HMC formation in drug development and set the stage for rational pharmacokinetic optimization of BTZs and similar drug candidates.

## Abstract (web summary)

Benzothiazinones (BTZs) are being developed as new antibiotics against the infection caused by *Mycobacterium tuberculosis*, however, BTZs can undergo an in vivo biotransformation to hydride-Meisenheimer complexes (HMC). Here, the authors show that HMC formation can be modulated by C-6 substitution of BTZ.

## Introduction

With over 1.5 million deaths in 2021, tuberculosis remained the deadliest disease owing to a single bacterial infectious agent, ranking well-above HIV and AIDS^1^. Caused by *Mycobacterium tuberculosis* (*Mtb*), its typical treatment consists of a combination therapy of four different antibiotics over six months^2^. However, the emergence of multidrug-resistant (MDR) and extensively drug-resistant (XDR) strains substantially reduces treatment success, and new replacement antibiotics are needed to innovate drug regimens^3^. Moreover, in the last 10 years, only three new antitubercular drugs, bedaquiline, delamanid and pretomanid were approved by health authorities^4,5^.

**Fig. 1:**
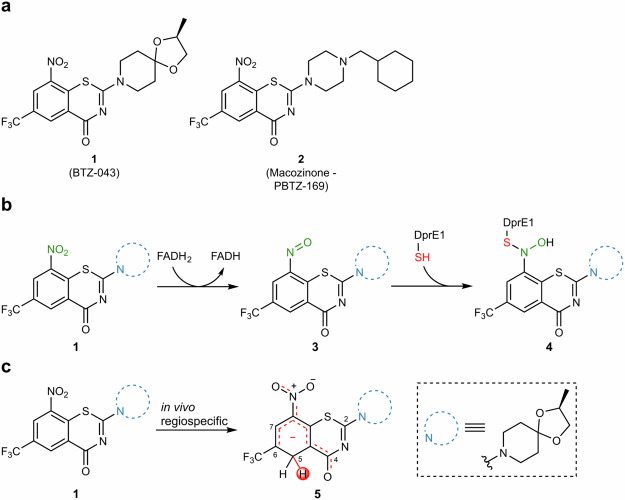
Structure, mode of action and hydride-Meisenheimer complex biotransformation of BTZs. **a** Structure of BTZ-043 (**1**) and macozinone (**2**), both currently in clinical development (phase II). **b** Mechanism of BTZs on DprE1. Nitro-BTZ **1** is transformed *via* a FADH_2_-mediated reduction to nitroso intermediate **3**, which reacts with a cysteine residue to form a covalent linkage **4**. **c** In vivo formation of a hydride-Meisenheimer complex as the main metabolite of **1**. Only regioisomer **5** is observed.

Novel classes addressing yet untapped targets are currently being developed, including BTZ-043 (**1**) and PBTZ-169 (**2**) as two members of the nitro-benzothiazinone (BTZ) family (Fig. 1a)^6^. BTZs demonstrated excellent activity against susceptible and resistant *Mtb* strains through engagement of decaprenylphosphoryl-β-d-ribose oxidase 1 (DprE1), a key enzyme in arabinogalactan biosynthesis^7,8^. The mechanism of inhibition involves a FADH_2_ mediated reduction of the nitro moiety to the nitroso-intermediate **3**, followed by nucleophilic attack of an adjacent cysteine to afford semimercaptal **4** (Fig. 1b)^9^. Aside from their potent antibacterial activity, BTZs demonstrated a favorable toxicological profile in animals and safety in humans, whereupon phase IIa study on **1** has recently been completed^10,11^.

**Fig. 2:**
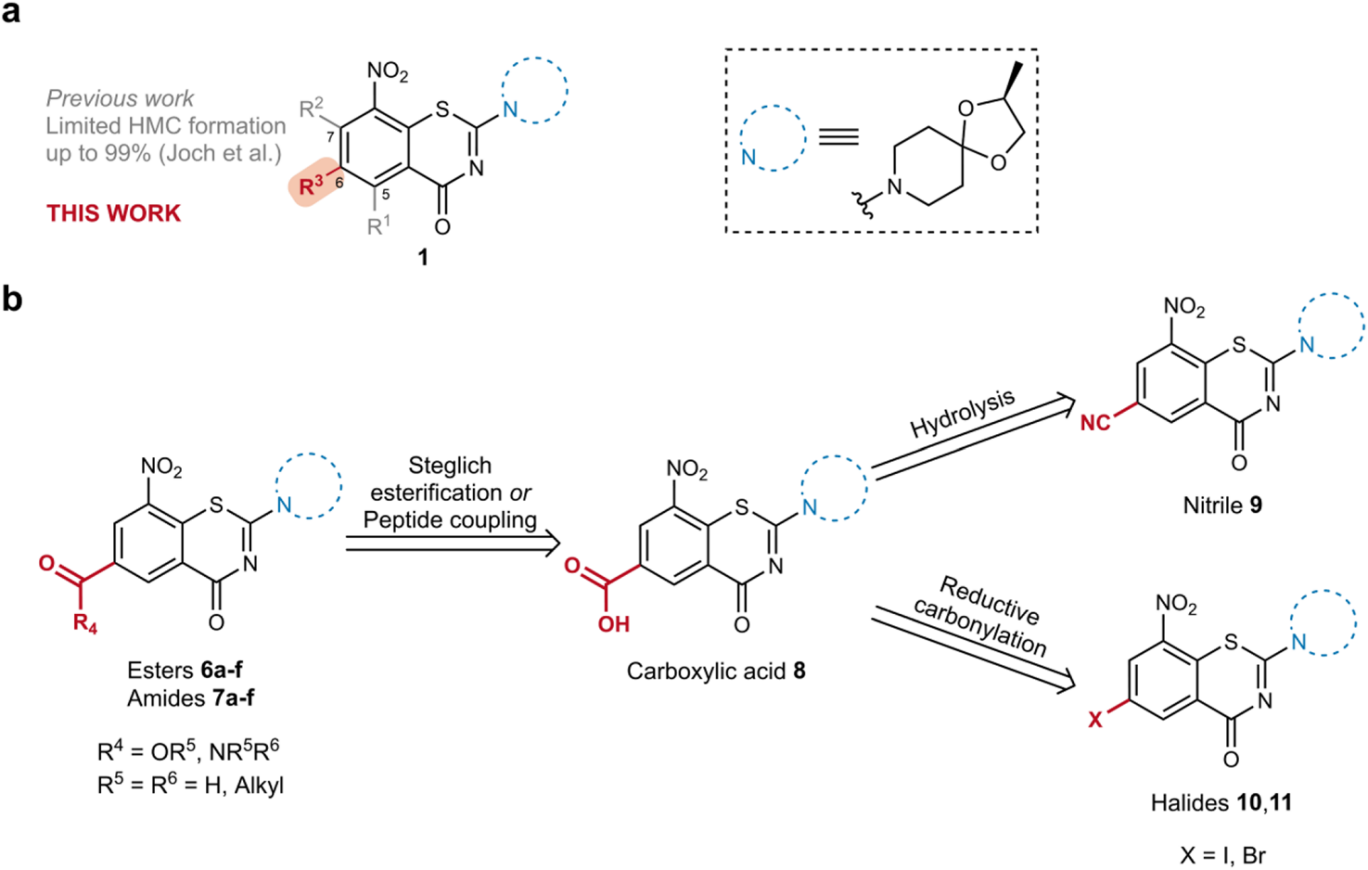
Substituent effects on hydride-Meisenheimer complex formation. **a** Previous work (gray) demonstrated that alkylation at positions C-5 and/or C-7 limits HMC formation. This work (red) focuses on the effect of different electron withdrawing groups (EWGs) on the HMC formation propensity. **b** Retrosynthetic analysis: Esters **6a–f** and amides **7a–f** are obtained from carboxylic acid **8**, which could be obtained either from hydrolysis of nitrile **9** or *via* a reductive carbonylation from halides **10** and **11**.

During preclinical studies, we discovered that **1** undergoes significant hydride-Meisenheimer complex (HMC) formation in vivo (Fig. 1c) *via* a yet unknown mechanism^12^. The relevance of this metabolic pathway has also been confirmed in humans^13,14^, providing a strong rationale for lead optimization towards fast-follower candidates. Recently, we described the first robust in vitro HMC biotransformation assay based on a RAW cell line^15^. This assay enabled a first round of metabolism-guided lead optimization, in which we could demonstrate that substitution at positions C-5 and C-7 dramatically decreased HMC formation while the antitubercular activity could be retained for small substituents such as methyl and ethyl (Fig. 2a)^15,16^.

Even though the presence of electron-withdrawing groups (EWGs) at position C-6 showed to be essential for the antimycobacterial activity^8,17–21^, the effects of C-6 substituents on HMC formation propensity remain unknown. This presented a unique opportunity to investigate the underlying parameters behind the formation of HMC *via* C-6 substituent modification of BTZs and to develop a reliable workflow to help understand this peculiar metabolism for potential future cases in drug development. We present herein the synthesis of a strategic library of BTZs bearing carboxylate groups on C-6 (Fig. 2b) and the evaluation of their propensity for HMC formation. In addition to experimental assay data, HMC formation was investigated by chemoinformatics analyses in order to identify and qualify a potential tool for future rational lead optimization endeavors. Moreover, reductive fluorocarbonylation is shown as a key step for late-stage functionalization of BTZs. Antimycobacterial and cytotoxic activities, as well as metabolic stability were assessed for most active analogues.

## Results and discussion

### Synthesis of nitrobenzothiazinones

**Fig. 3:**
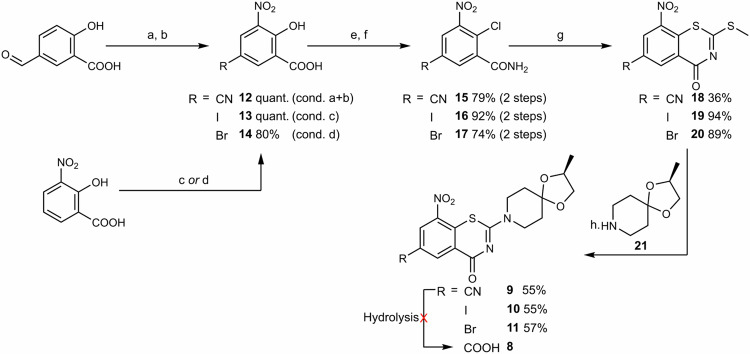
Synthesis of nitrile 9, iodide 10 and bromide 11. Synthesis of nitrile **9** from 5-formyl salicylic acid, and synthesis of halides **10** and **11** from 3-nitrosalicylic acid. Reaction conditions: **a** NH_2_OH·HCl (1.0 equiv.), DMF, reflux; **b** HNO_3_ (excess), 5 min., 0 °C; **c** I_2_ (1.0 equiv.), Ag_2_SO_4_ (1.0 equiv.), EtOH/H_2_O (10:1), r.t.; **d** NBS (1.1 equiv.), H_2_SO_4_ (1.1 equiv.), MeCN, reflux; **e** (COCl)_2_ (4.0 equiv.), DMF, −20 °C to 85 °C *then* H_2_O (excess); **f** SOCl_2_ (3.0 equiv.), toluene, reflux *then* NH_4_OH (excess), MeCN, −20 °C (for **15**) *or* NH_4_OH (excess), THF (for **16** and **17**), 0 °C to r.t.; **g** NaOH (50% aq.) (2.0 equiv.), CS_2_ (2.2 equiv.) *then* MeI (1.05 equiv.), DMSO, 10 °C; **h 21** (1.1 equiv.), EtOH, reflux. DMF dimethyl formamide, NBS *N*-bromosuccinimide, THF tetrahydrofuran, DMSO dimethylsulfoxide. See Supplementary Method 1 for details.

To access the carboxylic acid precursor **8**, our initial strategy involved the hydrolysis of nitrile **9** (Fig. 2b). Thioether **18** was produced *via* a modified synthetic route that was reported previously (Fig. 3)^21^. Condensation of 5-formyl salicylic acid with hydroxylamine, followed by nitration with fuming nitric acid quantitatively yielded nitrile **12**. Hydroxy-chloride exchange with oxalyl chloride and subsequent aminolysis gave amide **15** with 79% yield over 2 steps. Thioether **18** was obtained following a one-pot two-step cyclization in basic media^22^. Finally, substitution with amine **21** led to nitrile **9** in 55% yield. Hydrolysis attempts, however, resulted in decomposition products either by ketal cleavage or thiazinone hydrolysis under acidic or basic conditions, respectively.

To get access to the key carboxylic acid **8**, we sought after a different strategy. Ueda et al.^23^ showed that carboxylic acids, esters, and amides can be obtained in a single step from aryl halides *via* palladium-catalyzed fluorocarbonylation. This motivated us to synthesize iodide **10** and bromide **11** (Fig. 2b) using this approach. Direct halogenation of 3-nitrosalicylic acid gave iodide **13** and bromide **14** in high yields. Iodide **10** and bromide **11** were then obtained using the same route as for the nitrile in 5 steps from 3-nitro salicylic acid with total yields of 48 and 30%, respectively (Fig. 3).

**Fig. 4:**
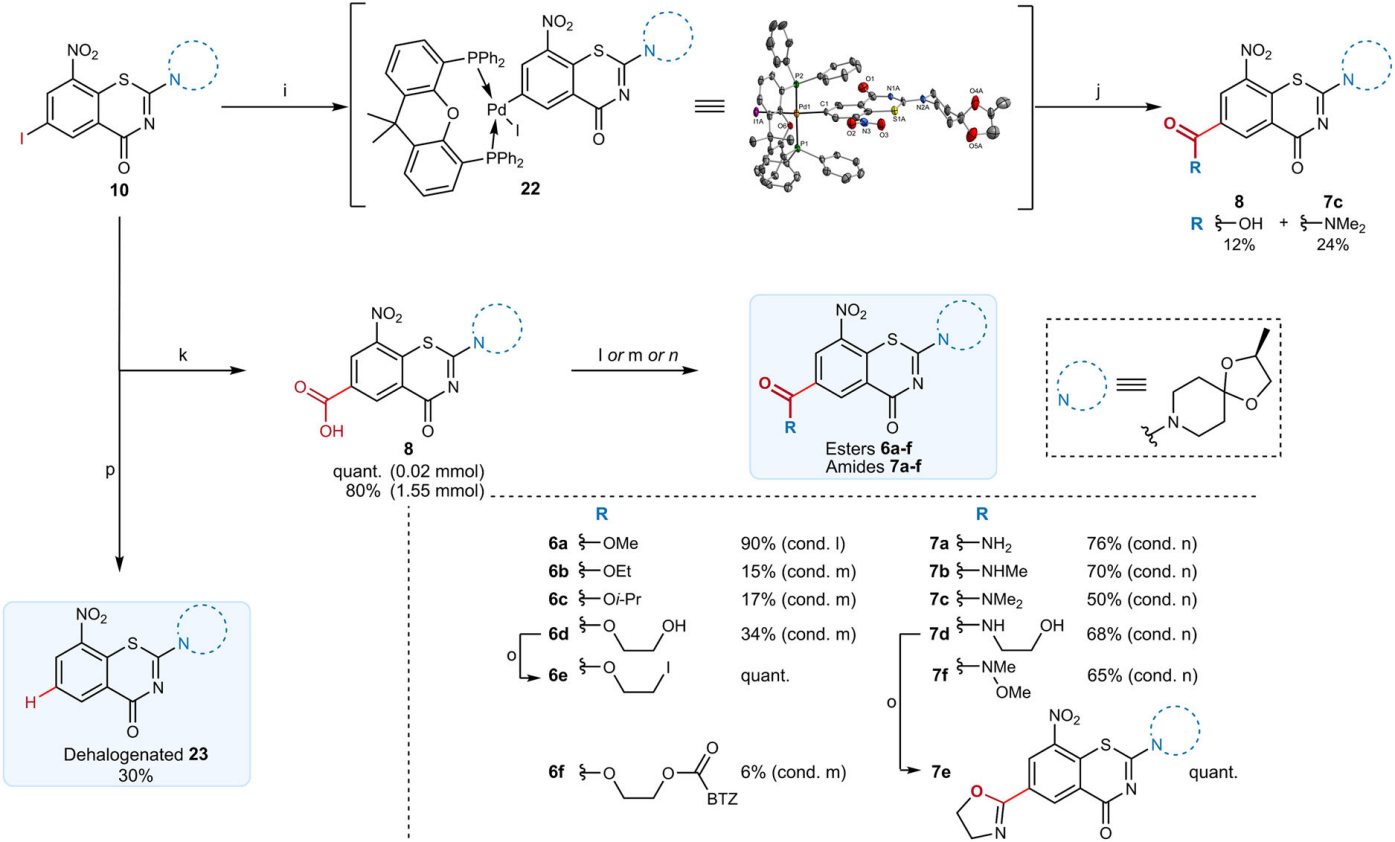
Optimization of the fluorocarbonylation and synthesis of ester and amide derivatives. Reaction conditions: **i** Pd(AcO_2_)_2_ (10 mol%), xantphos (15 mol%), *N*-formylsaccharin (3.0 equiv.), KF (5.0 equiv.), DMF, 80 °C, 18 h, N_2_, *then* NEt_3_ (2.5 equiv.), H_2_O (10.0 equiv.), r.t.; **j** Pd(AcO_2_)_2_ (10 mol%), xantphos (15 mol%), *N*-formylsaccharin (3.0 equiv.), KF (5.0 equiv.), DMF, 120 °C, 18 h, N_2_, *then* NEt_3_ (2.5 equiv.), H_2_O (10.0 equiv.), r.t.; **k** Pd(AcO)_2_ (6 mol%), dppp (9 mol%), *N*-formylsaccharin (3.0 equiv.), KF (5.0 equiv.), NMP, 120 °C, 18 h, N_2_, *then* NEt_3_ (2.5 equiv.), H_2_O (10.0 equiv.), r.t. **l** TMSCHN_2_ (20.0 equiv.), MeOH (0.05 M), r.t.; **m** RH (10.0 equiv.), EDC·HCl (1.5 equiv.), DMAP (1.0 equiv.), DMF, 45 °C; **n** RH (1.1 equiv.), HOBt (1.1 equiv.), HBTU (1.1 equiv.), DIPEA (1.1–3.0 equiv.), DMF, 45 °C; **o** I_2_ (2.0 equiv.), PPh_3_ (2.0 equiv.), imidazole (2.5 equiv.), THF, reflux. **p** Pd(AcO_2_)_2_ (6 mol%), dppp (9 mol%), *N*-formylsaccharin (3.0 equiv.), Na_2_CO_3_ (2.0 equiv.), Et_3_SiH (1.5 equiv.), DMF, 80 °C, 18 h, N_2_. Xantphos 4,5-Bis-(diphenylphosphino)-9,9-dimethylxanthen, dppp 1,3-Bis(diphenylphosphino)propane, EDC·HCl 1-ethyl-3-(3-dimethylaminopropyl)carbodiimide hydrochloride, DMAP *N*,*N*-dimethylpyridin-4-amine, DIPEA *N*,*N*-diisopropylethylamine, HOBt 1-hydroxy-1*H*-benzotriazol, HBTU 2-(1*H*-benzotriazole-1-yl)-1,1,3,3-tetramethyluronium hexafluorophosphate, MeOH methanol, DMF dimethylformamide, NMP *N*-methyl-2-pyrrolidone, TMSCHN_2_ trimethylsilyldiazomethane, cond. condition. See Supplementary Methods 2–4 for details.

Carboxylic acid **8** was obtained *via* one-pot two-step palladium-catalyzed fluorocarbonylation after extensive optimization (see Table S1 for details). Under the conditions described by Ueda et al.^23^, only iodide **10** could be transformed into carboxylic acid **8** (Fig. 4 and Fig. S1). The stepwise catalytic carbonylation led to a remarkably stable oxidative addition intermediate **22** (Fig. S1), which could be purified on silica and crystallized. Its structure was determined by X-ray crystallography (Fig. S2, Table S2). The observation of this complex not only supported the mechanistic proposal of Ueda et al. citing oxidative addition as the initial reaction step^24^, but also confirmed that **10** was reactive under the applied conditions. Higher temperatures led to the desired product, but formation of amide **7c** was predominant, presumably due to decomposition of DMF, and yields were overall unsatisfying^25^. Eventually, exchange of the solvent to *N*-methyl-2-pyrrolidone led to exclusive formation of acid **8**. Under optimized reaction conditions, acid **8** was formed in up to 80% yield at 1.55 mmol scale.

Unfortunately, one-pot preparation of esters and amides by addition of alcohols and amines to the reaction media could not be achieved with previously optimized conditions, except for ester **6a** (Tables S3 and S4, Fig. S3). As an alternative route, esters **6b–d** and **6f** were prepared from acid **8** *via* Steglich esterification in low to moderate yields, whereas amides **7a–d** and **7f** were prepared *via* amide coupling in good yields. Additionally, ester **6a** was obtained by treatment of **8** using trimethylsilyldiazomethane^26^, while ester **6e** was synthesized *via* a modified Appel reaction from the corresponding alcohol. Surprisingly, when the same procedure was applied for **7d**, no hydroxy-iodide exchange was noted. Instead, only formation of oxazoline **7e** occurred (Fig. 4)^27,28^.

Interestingly, attempts to introduce an aldehyde moiety *via* reductive carbonylation under optimized conditions (cond. p, Fig. 4) only led to formation of dehalogenated BTZ **23**.

### Hydride Meisenheimer Complex formation

**Fig. 5:**
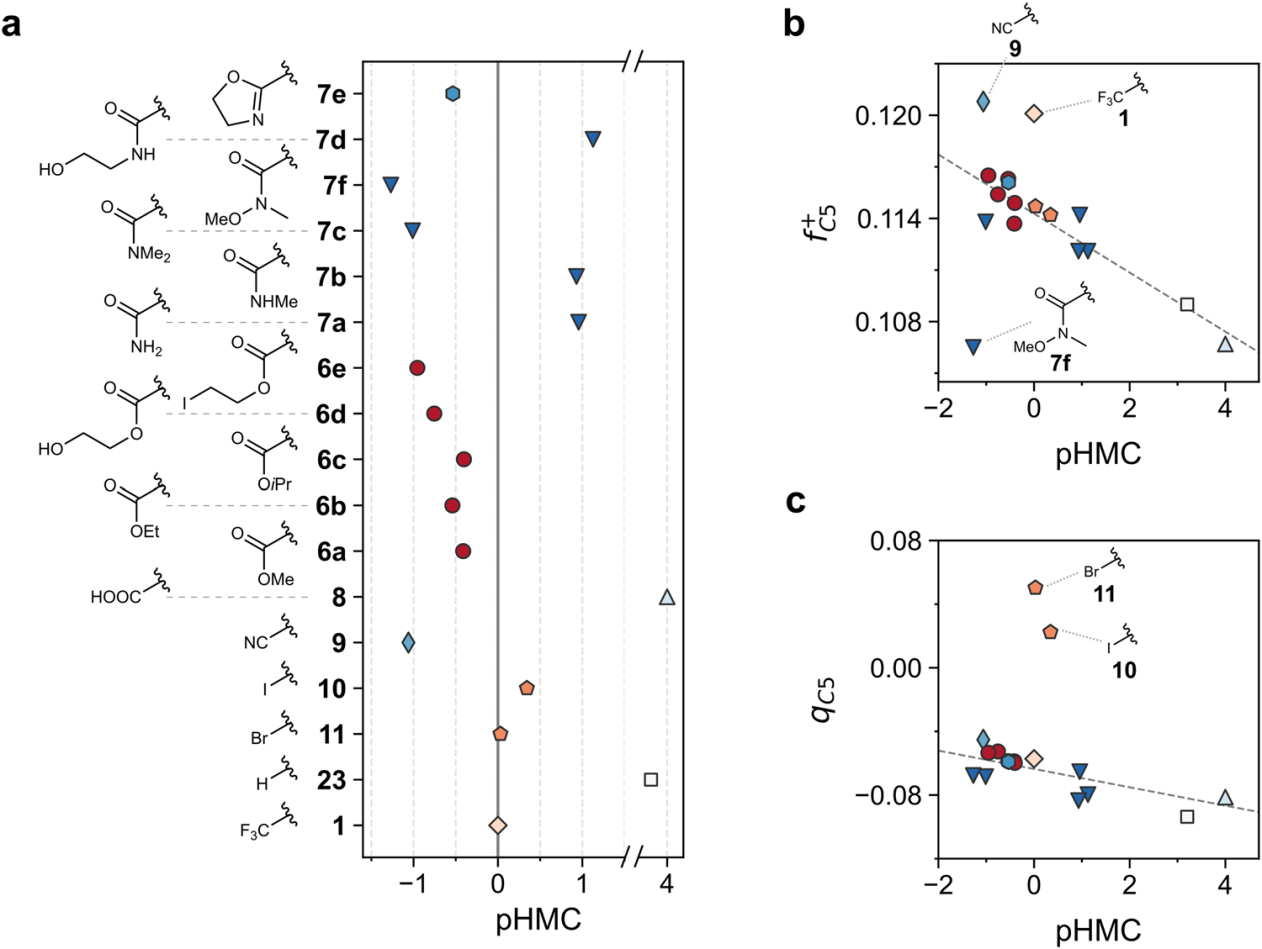
Propensity of Meisenheimer complex formation for synthesized compounds. **a** Meisenheimer complex formation relative to **1**, expressed as the negative decadic logarithm (pHMC). Vertical continuous line at zero highlights the propensity for reference compound **1**. Compounds to the right are less reactive, while those to the left are more prone to HMC formation. **b** Fukui indices and **c** Mulliken charges for C-5 calculated at CPCM(water)-B3LYP-D3/6-311+G(d,p)//B3LYP-D3/6-31+G(d,p) level, and their relationship with HMC propensity. Different colors and markers represent different compound types (e.g. red circles for esters, dark blue triangles for amides). Dashed lines in **b** and **c** represent linear trends irrespective of labeled outliers.

As the main goal of our work, we focused on the impact of C-6 substituents on the propensity to form HMC in our recently developed whole cell in vitro assay. Following our standardized protocol^15^, all measurements were normalized relative to BTZ-043 (**1**). Obtained HMC formation propensities (Table S5) were transformed into their negative decadic logarithm (pHMC) to ease data visualization and subsequent processing during chemoinformatics investigations (Fig. 5). Surprisingly, HMC formation did not appear to be directly related to the respective electron-withdrawing nature of each substituent, with amides showing a wide range of values despite small structural differences (Fig. 5a). In the case of esters, a plausible pattern according to the substituent size was observed. Weinreb amide **7f** showed the highest tendency to form HMC, producing 19-fold more than **1**. Similarly, dimethyl amide **7c** and nitrile **9** produced a 14- and 12-fold larger ratio of HMC compared to **1**, respectively. Esters **6a–e** and oxazoline **7e** were also more prone to HMC formation than **1**, albeit to a considerably lesser extent (2.5–6-fold). Bromide **11** showed about the same level as **1**. In contrast, monoalkylated amides **7b** and **7d**, and primary amide **7a** exhibited lower propensity to HMC formation with up to 10-fold less than reference compound **1**. Traces to no detectable HMC formation were observed for acid **8** and unsubstituted BTZ **23**, respectively. This indicated that the presence of a charged EWG (**8**) or the absence of any EWG (**23**) arguably prevents the stabilization of the negative charge resulting from HMC formation.

In an attempt to understand which parameters influence HMC formation, we calculated quantum mechanics-derived chemical descriptors through conceptual density functional theory (CDFT)^29–31^. The optimized structures of the lowest-energy conformer for each compound were used for calculation of CDFT descriptors at B3LYP-D3/6-311+G(d,p) level in Jaguar^32^. Solvent effects were considered by the CPCM model using water^33^. Neither global electrophilicity nor chemical potential showed a uniform correlation for the whole set of compounds (Fig. S4a, b). Nevertheless, acid **8** and non-substituted BTZ **23** showed the lowest electrophilicity, in agreement with their apparent lack of reactivity to form HMC. The HOMO-LUMO gap was also unable to reflect the experimentally observed propensity of HMC formation (Fig. S4c).

Based on the failure of global descriptors to properly describe the observed phenomena, we drew our attention to atomic descriptors. Thus, Mulliken charges and condensed Fukui indices were calculated^34,35^. Thorough analysis of the Fukui indices (${f}_{{A}_{i}}^{+}$) demonstrated that positions C-5 and C-7 are highly reactive toward nucleophilic attack, which supports the assessed ability of those compounds to form HMC. As it could be anticipated, for most compounds, an increase in ${f}_{C5}^{+}$ translated into higher propensity of HMC formation (Fig. 5b). To our surprise, BTZ-043 (**1**), nitrile **9**, and amide **7f** were outliers, though, showing marked deviations from the expected reactivity-based behavior. The currently available information is insufficient to understand their peculiar behavior. Similar analysis on ${f}_{C7}^{+}$ was not conclusive (Fig. S4d). In contrast, Mulliken charges on C-5 displayed good correlation with HMC propensity for the compounds bearing a carboxylate (Pearson’s correlation = −0.79, *p* < 0.01; Fig. 5c). Arguably, the differences in Mulliken charges for **10** and **11** with respect to the remaining set of compounds can be explained by their significantly different electronic effects compared to the carboxylate group.

**Fig. 6:**
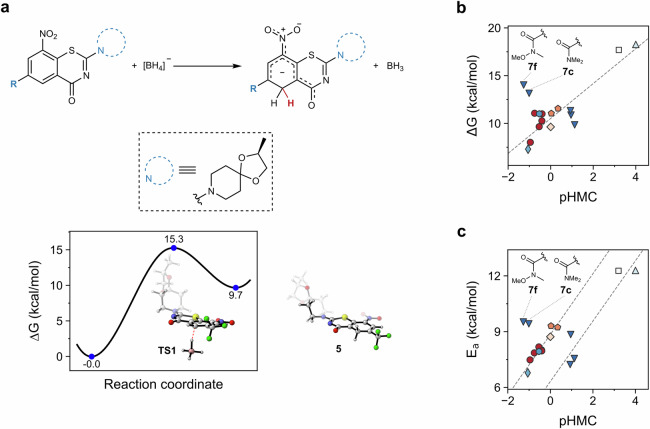
Reaction profiling for hydride Meisenheimer complex formation. **a** Hydride addition to **1** to form **5**, and its reaction profile. **b** Gibbs free energy and **c** activation energy for the set of synthesized compounds at CPCM(water)-PBE0-D3BJ/ma-def2-TZVP//PBE0-D3BJ/ma-def2-SVP level. Complete modeling and automatic transition state search performed using autodE^36^. Different colors and markers represent different compound types (e.g. red circles for esters, dark blue triangles for amides). Dashed lines in **b** and **c** represent linear trends irrespective of labeled outliers.

Encouraged by those results, we decided to determine the reaction energetics of hydride addition on C-5 using a surrogate model reaction with borohydride anion as hydride donor (Fig. 6a). The complete set of calculations was automated using the freely available Python package autodE^36^, with ORCA^37,38^ as quantum mechanics engine. We observed a significant, positive correlation between the reaction free energy (Δ*G*) and HMC formation propensity (Fig. 6b), where only amides **7c** and **7f** deviated (Pearson’s correlation = 0.90, *p* < 0.01, after removal of outliers **7c** and **7f**). Larger Δ*G* accounted for lower product stability compared to the original compound. According to the calculated Δ*G*, amides **7c** and **7f** would be expected to have lower propensity of HMC formation. Their high HMC formation propensity might be due to additional factors beyond thermodynamic considerations. This is plausible, as the whole cell biotransformation is likely discriminated by steric constraints, specific interactions within the catalyzing enzyme as well as transport/permeation kinetics. On the other hand, comparison of HMC formation propensity with the activation energy (*E*_a_) revealed the existence of two separate trends (Fig. 6c), one for compounds prone to HMC formation, and one for non-reactive and/or poorly reactive compounds. This implies that *E*_a_ itself cannot be used for HMC formation propensity-based decision making. The two reactivity patterns found herein are however in good agreement with expectations derived from chemical knowledge, i.e., the higher the activation energy, the lower the reactivity. The high *E*_a_ calculated for amides **7c** and **7f** might be partially attributed to steric hindrance caused by the substituents of the tertiary amide.

### Antimycobacterial activity

**Fig. 7:**
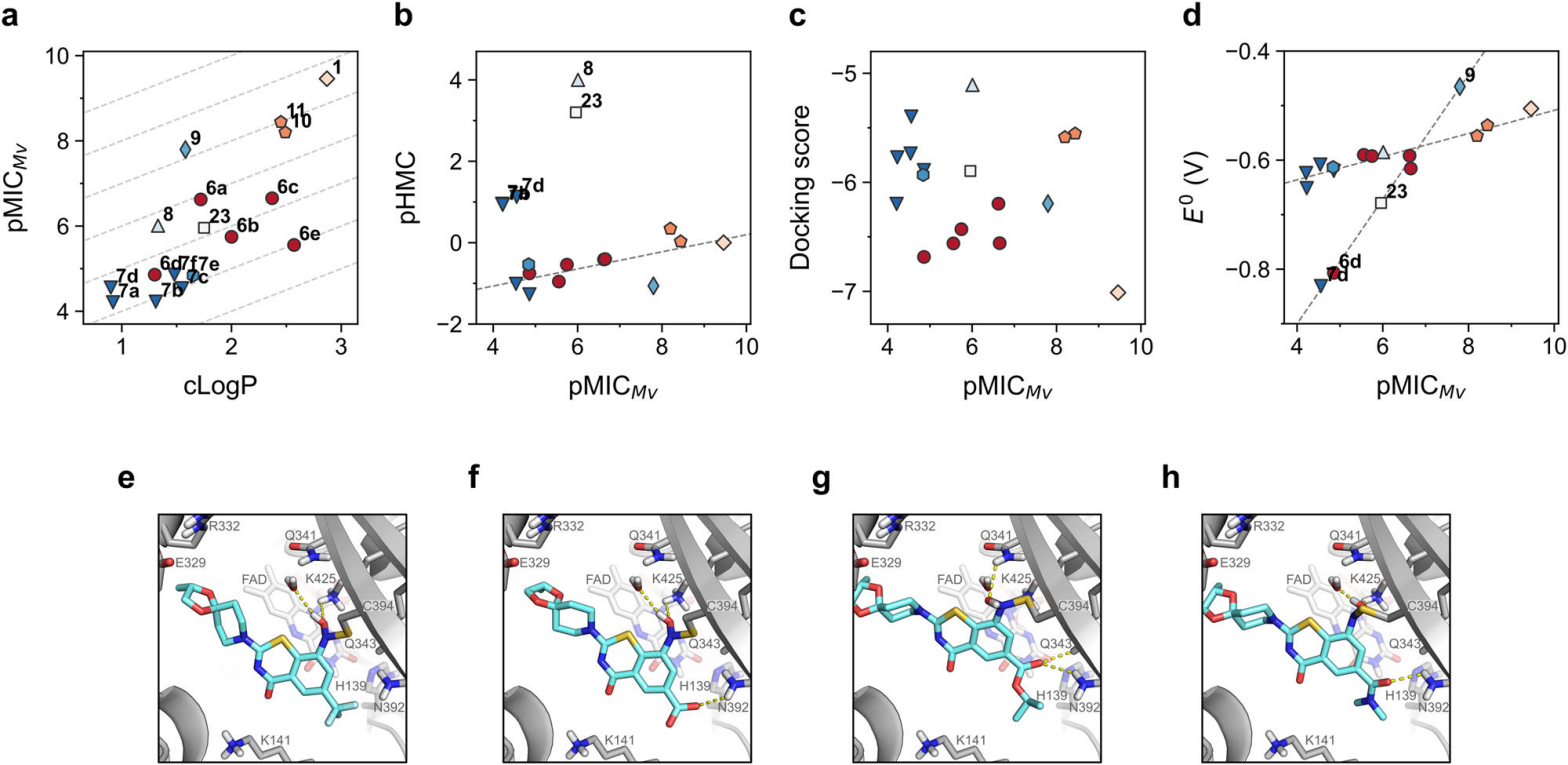
Antimycobacterial activity and molecular modeling. **a** Activity against *Mycobacterium vaccae* as a function of cLogP. pMIC = negative decadic logarithm of the minimum inhibitory concentration (MIC) against *M. vaccae*. clogP was obtained from swissADME^46^. LLE was calculated as the difference between the pMIC and clogP. **b** Antimycobacterial activity compared to in vitro HMC propensity. **c** Docking scores from covalent docking in relationship to antimycobacterial activity. **d** Calculated standard redox potential for synthesized compounds at CPCM(water)-PBE0-D3BJ/ma-def2-TZVP//PBE0-D3BJ/ma-def2-SVP level. Values are calculated for 298 K, relative to the standard hydrogen electrode. Different colors and markers in **a**–**d** represent different compound types (e.g. red circles for esters, dark blue triangles for amides). Dashed lines in **a** represent different LLE values. Dashed lines in **b** and **d** represent linear trends irrespective of labeled outliers. Representative docking poses for **e** BTZ-043 (**1**), **f** acid **8**, **g** ester **6c**, and **h** amide **7c**, showing only selected residues to ease visualization. Cyan licorice for covalently bound ligand. Docking poses were obtained using the CovDock workflow in Maestro.

Antimycobacterial and cytotoxic activity were assessed for the complete set of synthesized BTZs as shown in Table S5. All tested compounds were found to be non-cytotoxic. The structural modifications studied herein led to similar changes in activity against both *M. vaccae* and *M. smegmatis*, which turned into a strong positive correlation between them (Pearson correlation = 0.97, *p* < 0.001, Fig. S5). Therefore, only the former was used for subsequent analyses. The antimycobacterial activity against *M. vaccae* ranged from 50 µM to 3 nM (Fig. 7a), in agreement with the already known influence of C-6-substitution on the antimicrobial activity. As expected, nitrile **9** and halides **10** and **11** were similarly active as BTZ-043 (**1**), as they are bioisosteres of the CF_3_ moiety^39^. In contrast, unsubstituted BTZ **23** showed significantly lower activity than **1** and its bioisosteres, confirming once again the fundamental role of EWGs at C-6. Carboxylic acid **8** showed moderate activity, comparable to the esters **6a**–**6f**, whereas amides **7a**–**7d** and **7f** and oxazoline **7e** were pronouncedly less active. Based on lipophilic ligand efficiency (LLE, Fig. 7a)^40,41^, this is anticipated to be owing to the relatively high polarity of the amides. For the set of synthesized compounds, we found a positive, significant correlation between cLogP and activity (Pearson correlation = 0.76, *p* < 0.001). The impact of lipophilicity on the antimycobacterial potential of BTZs has already been observed for a larger and more diverse set of compounds during machine learning-based modeling^42^. Notably, there was no gain in activity for the amides with similar LLE (~3) despite structural changes. This fact indicates that the antimycobacterial activity is not exclusively driven by lipophilicity. In the case of esters (**6a**–**6f**), a wider range of LLE was observed (Fig. 7a). Strikingly, compounds prone to HMC formation (HMC > 0.3) exhibited a somewhat linear correlation (Pearson correlation = 0.71, *p* < 0.01) between the experimental propensity to form HMC and inhibition of mycobacterial growth (Fig. 7b). Thus, for those compounds, HMC formation is arguably detrimental for the antimycobacterial activity.

In order to better understand, which parameters control the observed bioactivity, membrane permeability was calculated, using the Schrödinger Suite (Fig. S6). Notably, with the exception of acid **8**, a clear linear correlation between predicted permeability and activity was found (Pearson correlation = 0.88, *p* < 0.001), indicating that permeability is one key parameter for the compounds studied.

Furthermore, docking simulations were carried out using the CovDock workflow of the Schrödinger Suite^43^. Bond formation between the nitroso moiety of the reduced BTZs and Cys394 in the binding pocket of DprE1 was assumed, following the known mechanism of action for this kind of compounds (as described above; see Fig. 1). In agreement with previous observations^42^, no clear correlation between the antimycobacterial activity and docking scores could be found (Fig. 7c). This supports the expected rationale that biochemical potency at molecular target level does not guarantee antimycobacterial activity. Notably, binding free energies for the non-covalent inhibition (prior to covalent bond formation) showed a trend for most compounds (Fig. S7a). Binding pose examination showed hydrogen bond formation between the carboxylate group of the newly synthesized compounds with Asn392 (Fig. 7e–h). Unbiased molecular dynamics (MD) simulations for representative compounds confirmed the persistence of this interaction for at least 72% of the simulated time (Fig. S8). In contrast, hydrogen bonding between the N–OH group of the ligand and Lys425 initially observed in the docked pose was not maintained along the MD trajectories. Stable interaction with Gln341 was observed instead for compound **1** (Fig. S8a), whereas acid **8**, ester **6c**, and amide **7c** exhibited contacts with Gln343 (Fig. S8b–d), whereupon comparable target inhibition to **1** would be expected. However, as mentioned earlier, unaccounted differences in MIC values might arise from significant changes in polarity and membrane permeability, ultimately leading to a drop in activity. The observed changes in protein–ligand interactions are due to the larger size of the C-6 substituents, which induce an outward displacement of the BTZ core within the binding pocket (compared to **1**; Fig. S7b). Based on the close proximity between the NO_2_ group of the ligand and Cys384 required for the nucleophilic attack (Fig. 1b), we further assessed whether poses showing a preferred orientation in non-covalent docking are more stable using binding pose metadynamics^44^. In general, the best pose for esters and amides showed high resemblance to those observed by covalent docking (Fig. S7c). Remarkably, a strong interaction (hydrogen bond/ionic) between the NO_2_ group and Lys425 appeared responsible for guiding and keeping the binding orientation (Fig. S7d–g). Acid **8** showed however a 180° binding pose flip, where polar interactions between NO_2_ and Lys141 are likely (Fig. S7e), partially explaining the measured low activity.

We have previously observed that differences in calculated redox potential may be related to changes in antimycobacterial potency for fused-ring analogues of BTZ^45^. Thus, *E*^0^ values for the synthesized compounds were obtained at DFT level (Fig. 7d), taking advantage of the automation capabilities provided by autodE^36^. The first redox potential correlates well with the biological activity for most of the compounds. Even though nitrile **9**, unsubstituted **23**, and hydroxyethyl-containing compounds **6d** and **7d** are out of the main correlation observed (Pearson’s correlation = 0.92, *p* < 0.01, after removal of the anticipated outliers), they represent another linear trend. Therefore, modification of the electron density of the BTZ core by substitution on C-6 is also responsible for perturbations in the compound’s reduction readiness, which is necessary to reach subsequent covalent binding (Fig. 1b).

### Microsomal stability

The metabolic stability of selected compounds was evaluated in terms of in vitro half-life (t_1/2_, Table S6). Half-lives decrease in the order: amides **7c** and **7f** > nitrile **9** > bromide **11** > iodide **10** > esters **6a** and **6c**. Esters are characterized by poor metabolic stability due to rapid hydrolysis, whereas amides are rather stable. Among the selected compounds, halides **10** and **11** showed the best balance between activity and stability.

## Conclusions

In summary, hydride-Meisenheimer complex (HMC) formation can be effectively modulated on BTZs by C-6 substitution as demonstrated by our in vitro assay results. From in depth analysis of reactivity patterns using quantum mechanics, we conclude that Mulliken charges and Fukui indices for C-5 represent the most suitable atomic properties to anticipate HMC formation propensity. Gibbs free energy of reaction with borohydride may provide a further valuable predictive link.

The hydride-Meisenheimer metabolic pathway is an abundant feature of BTZs and its modulation and prediction are important to assess prior to preclinical and clinical development, and therefore must play an essential role as part of multi-parameter lead optimization. However, reducing HMC formation without compromising antimycobacterial activity remains a challenge. Covalent docking simulations suggest that the antimicrobial activity of carboxylate-containing BTZ derivatives is unrelated to target inhibition, while calculated redox potentials indicate that the readiness of nitro reduction drives, at least partially, the biological potency of these compounds.

The results presented here significantly streamline the rational design of next generation benzothiazinones and may also inspire programs beyond the BTZ scaffold. Our fundamentally new workflow has the potential to prioritize compounds at design level, well in advance to actual synthesis and in vitro testing, while avoiding potential conflicts with HMC metabolism.

## Methods

Chemical and biological experimental and computational methods are described in the Supplementary Information. General information about the analysis and purification techniques (NMR, UHPLC-HRMS, X-Ray crystallography) are described in the Supplementary Note 3. Experimental procedures associated to the synthesis of compounds **9,** **10** and **11** (Fig. 3) are described in the Supplementary Method 1. Experimental procedures associated to the reductive carbonylation reaction, and the formation of the palladium complexes (Fig. 4) are described in the Supplementary Method 2 and 3, respectively. Experimental procedures associated to the synthesis of the ester (**6a–f**) and amide (**7a–f**) libraries (Fig. 4) are described in the Supplementary Method 4. Performed biological assays are described in the Supplementary Method 5. Computational modeling and chemoinformatics analyses are described in the Supplementary Method 6.

### Reporting summary

Further information on research design is available in the Nature Portfolio Reporting Summary linked to this article.

### Supplementary information

Supplementary Information

Description of Additional Supplementary Files

Supplementary Data 1

Supplementary Data 2

Supplementary Data 3

Supplementary Data 4

Supplementary Data 5

Supplementary Data 6

Reporting Summary

## Data Availability

The authors declare that the data supporting the findings of this study are available within the article and Supplementary Information. All cited Supplementary Tables and Supplementary Figs. are grouped in the Supplementary Information under the subsections Supplementary Notes 1 and 2, respectively. For experimental details and compounds characterization, see Supplementary Methods 1–6. For NMR Spectra, see Supplementary Data 1. For X-Ray crystallography, see Supplementary Data 2–4. The full crystallographic data can be obtained free of charge from the Cambridge Crystallographic Data Centre with the accession codes CDCC #2278946 (**22**), #2278947 (**S7**), #2278948 (**S5**). For cartesian coordinates of all modeled compounds, see Supplementary Data 5. For the complete computational modeling data, see Supplementary Data 6.

## Code availability

Code used for data analysis in this study is publicly available at https://github.com/BernalFA/HMC-carboxy-BTZs.
