# Supplementary material for: Modulation of the Meisenheimer complex metabolism of nitro-benzothiazinones by targeted C-6 substitution
Source: Commun Chem. 2024 Jul 6;7:153. doi: 10.1038/s42004-024-01235-x (PMC11227536; doi:10.1038/s42004-024-01235-x)
Supplement: Supplementary file 1 — Supplementary Information [file 42004_2024_1235_MOESM1_ESM.pdf]

Supplementary Information

Modulation of the Meisenheimer complex metabolism of nitro-benzothiazinones by targeted C-6 substitution

François Keiff,[1] Freddy A. Bernal,[1] Melanie Joch,[1] Thibault Joseph William Jacques-dit-Lapierre,[1] Yan Li,[1] Phil Liebing,[2] Hans-Martin Dahse,[3] Ivan Vilotijevic,[4] Florian Kloss*[1]

[1] Transfer Group Anti-infectives, Leibniz Institute for Natural Products Research and Infection Biology – Leibniz-HKI, Beutenbergstr. 11a, 07745 Jena, Germany.

[2] Institute for Inorganic and Analytical Chemistry, Friedrich-Schiller-Universität Jena, Humboldtstr. 8, 07743 Jena, Germany.

[3] Department of Infection Biology, Leibniz Institute for Natural Product Research and Infection Biology – Leibniz-HKI, Beutenbergstr. 11a, 07745 Jena, Germany.

[4] Institute of Organic Chemistry and Macromolecular Chemistry, Friedrich Schiller University Jena, Humboldtstr. 10, Jena 07743, Germany.

Table of content

Table of content 2

List of Supplementary Tables 3

List of Supplementary Figures 3

List of abbreviations 4

Supplementary Note 1: Supplementary Tables 5

Supplementary Note 2: Supplementary Figures 13

Supplementary Note 3: General Information 18

Supplementary Method 1: Synthesis of cyanide **9**, iodide **10** and bromide **11** 19

Supplementary Method 2: Reductive carbonylation condition screening 27

1. General screening conditions for carboxylic acid **8** 27
2. Reductive carbonylation on larger scale (entry 50 of Table S1) 28
3. Reductive carbonylation towards methyl ester **6a** 28
4. Dehalogenation and isolation of compound **23** 30

Supplementary Method 3: Palladium complex formation and single crystal structure determination 31

1. Synthesis of palladium complex (xantphos)PdIBTZ **22** and (xantphos(O))PdIBTZ **S7** 31
2. Synthesis of palladium complex (dppp)PdIBTZ **S5** 35

Supplementary Method 4: Synthesis of the carboxylic acid derivative library 36

1. General Procedure A – Steglich esterification 36
2. General Procedure B – Amide coupling 40

Supplementary Method 5: Biological Assays 44

1. MIC determination 44
2. Proliferation and cytotoxicity assay 44
3. Hydride Meisenheimer complex formation propensity assay 46
4. Microsomal stability assay 49

Supplementary Method 6: Computational modeling 51

1. Conceptual density functional theory (CDFT) descriptors 51
2. Reactivity – Hydride addition 52
3. Covalent docking 52
4. Non-covalent docking 52
5. Molecular dynamics simulations 53
6. Redox potentials 53
7. Membrane permeability 53

Supplementary References 54

List of Supplementary Tables

Table S1. Complete condition screening of the fluorocarbonylation reaction. 5
Table S2. Crystal data and structure refinement for **22 ·solvent**. 7
Table S3. Effect of the temperature on the formation of methyl ester **6a**.[a] 8
Table S4. Crystal data and structure refinement for **S5 · CH_2Cl_2**. 8
Table S5. Complete data set of performed biological assays. 10
Table S6. Microsomal stability assay of most active compounds. 12
Table S7. Crystal data and structure refinement for **S7 ·2 DMF**. 34
Table S8. List of calculated and found accurate mass for $[HMC+H]^+$ and the corresponding ion fragment $[HMC–2H]^+$.[a] 47

List of Supplementary Figures

Figure S1. UHPLC-HRMS analysis of the raw extract of reaction 1 (entry 1 - Table S1). 13
Figure S2. Molecular structure of **22** 14
Figure S3. Molecular structure of **S5**, as determined from a crystalline solvate **S5** · CH_2Cl_2 14
Figure S4. Global and local CDFT descriptors. 15
Figure S5. Comparison of antimycobacterial activity against two Mycobacteria. 15
Figure S6. Predicted membrane permeability (RRCK cells). 16
Figure S7. Compounds targeting DprE1 by molecular docking simulations. 16
Figure S8. Protein-ligand interactions from MD simulations of the covalently bound complex for representative compounds 17
Figure S9. Molecular structure of **S7**, as determined from a crystalline solvate **S7** · 2 DMF. 33

List of abbreviations

(U)HPLC	(ultra) high performance liquid chromatography
DCM	dichloromethane
(br) s	(broad) singlet
BTZ	benzothiazinone
d	doublet
dba	(1*E*,4*E*)-1,5-diphenylpenta-1,4-dien-3-one
dd	doublet of doublets
DMAP	4-dimethylaminopyridine
DMF	dimethylformamide
DMSO	dimethyl sulfoxide
dppb	(butane-1,4-diyl)bis(diphenylphosphane)
dppe	(ethane-1,2-diyl)bis(diphenylphosphane)
dppp	(propane-1,3-diyl)bis(diphenylphosphane)
dt	doublet of triplets
EDC	3-{[(ethylimino)methylidene]amino}-*N*,*N*-dimethylpropan-1-amine
ESI	electrospray ionization
EtOAc	ethyl acetate
EtOH	ethanol
FA	formic acid
hept	heptet
HRMS	high resolution mass spectrometry
m	multiplet
MeCN	acetonitrile
MeOH	methanol
NBS	N-bromosuccinimide
NEt_3	triethylamine
NMP	*N*-methyl-2-pyrrolidone
NMR	nuclear magnetic resonance
PDA	photodiode array
PE	petroleum ether
PPh_3	triphenylphosphine
q	quintet
quant.	quantitative
r.t.	room temperature
t	triplet
TFA	trifluoroacetic acid
THF	tetrahydrofuran
TIC	total ion current
TLC	thin layer chromatography
TMS	trimethylsilane
Xantphos	(9,9-dimethyl-9*H*-xanthene-4,5-diyl)bis(diphenylphosphane)

Supplementary Note 1: Supplementary Tables

Table S1. Complete condition screening of the fluorocarbonylation reaction.

Entry	Catalyst (mol%)	Ligand (mol%)	Solvent	Temperature (°C)	Work-up	Yield (%) (UHPLC)	Yield (%) (isolated)
1[a]	$Pd(AcO)_2$ (20)	Xantphos (30)	DMF	80	Water	Traces[b]	n.d.
2[c]	$Pd(AcO)_2$ (20)	Xantphos (30)	DMF	115	Water	-	-
3	$Pd(AcO)_2$ (10)	Xantphos (15)	DMF	80	Water	25%[b]	n.d.
4	$Pd(PPh_3)_4$ (50)	None	DMF	90	Water	-	-
5	$Pd(AcO)_2$ (10)	Xantphos (15)	DMF	120	Water	12%	12%[d]
6	$Pd(AcO)_2$ (10)	Xantphos (15)	NMP	80	Water	13%	n.d.
7	$Pd(AcO)_2$ (10)	Xantphos (15)	NMP	120	Water	32%	n.d.
8	$Pd(AcO)_2$ (10)	Xantphos (15)	NMP	150	Water	25%	n.d.
9[e,f]	$Pd(AcO)_2$ (10)	Xantphos (15)	NMP	120	Water	26%	18%
10[e,f,g]	$Pd(AcO)_2$ (10)	Xantphos (15)	NMP	120	Water	16%	13%
11	$Pd(PPh_3)_4$ (6)		NMP	120	Water	24%	24%
12	$Pd(PPh_3)_4$ (10)		NMP	120	Water	59%	54%
13[e]	$Pd(PPh_3)_4$ (10)		NMP	120	Water	44%	16%
14[h]	$Pd(PPh_3)_5$ (10)		NMP	120	Water	10%	n.d.
15	$Pd(PPh_3)_4$ (5)	PPh_3 (10)	1,4-dioxane	100	Water	n.d.	4%
16	$Pd(AcO)_2$ (10)	Xantphos (15)	1,4-dioxane	100	Water	n.d.	12%
17	$Pd(AcO)_2$ (10)	Xantphos (15)	1,4-dioxane	160	Water	16%	n.d.
18	$Pd(AcO)_2$ (5)	Xantphos (8)	NMP	120	Water	53%	48%
19[e]	$Pd(AcO)_2$ (5)	Xantphos (8)	NMP	120	Water	43%	35%
20	$Pd(AcO)_2$ (6)	Xantphos (9)	NMP	120	Water	21%	32%
21	$Pd(AcO)_2$ (6)	Xantphos (9)	DMF	120	Water	11%	Traces
22	$Pd(AcO)_2$ (6)	Xantphos (9)	NMP/DMF (1:3)	120	Water	7%	Traces
23	$Pd(AcO)_2$ (6)	Xantphos (9)	NMP/DMF (1:1)	120	Water	13%	Traces
24	$Pd(AcO)_2$ (6)	Xantphos (9)	NMP/DMF (3:1)	120	Water	21%	36%
25	$Pd(AcO)_2$ (6)	PPh_3 (9)	NMP	120	Water	2%	Traces
26	$Pd(AcO)_2$ (6)		NMP	120	Water	2%	Traces
27	$Pd(AcO)_2$ (6)	DPPE (9)	NMP	120	Water	32%	71%
28	$Pd(AcO)_2$ (6)	DPPB (9)	NMP	120	Water	20%	37%
29	$Pd(PPh_3)_4$ (6)	Xantphos (9)	NMP	120	Water	10%	5%
30	$Pd(AcO)_2$ (6)	Xantphos (9)	NMP	120	Citric acid	n.d.	69%
31	$Pd(PPh_3)_2Cl_2$ (6)	Xantphos (9)	NMP	120	Citric acid	n.d.	49%
32	$Pd(PPh_3)_4$ (6)	Xantphos (9)	NMP	120	Citric acid	n.d.	38%
33	Pd_2dba_3 (6)		NMP	120	Citric acid	n.d.	51%
34	Pd_2dba_3 (6)	Xantphos (9)	NMP	120	Citric acid	n.d.	56%
35	Pd_2dba_3 (6)	DPPP (9)	NMP	120	Citric acid	n.d.	54%
36	$Pd(AcO)_2$ (6)	PPh_3 (9)	NMP	120	Citric acid	44%	36%
37	$Pd(AcO)_2$ (6)	DPPE (9)	NMP	120	Citric acid	63%	40%
38	$Pd(AcO)_2$ (6)	DPPB (9)	NMP	120	Citric acid	17%	36%
**39**	**$Pd(AcO)_2$ (6)**	**DPPP (9)**	**NMP**	**120**	**Citric acid**	**n.d.**	**Quant.**
40	$Pd(AcO)_2$ (6)	DPPP (18)	NMP	120	Citric acid	n.d.	42%
41	$Pd(AcO)_2$ (6)	DPPP (9)	DMF	120	Citric acid	99%	85%

Entry	Catalyst (mol%)	Ligand (mol%)	Solvent	Temperature (°C)	Work-up	Yield (%) (UHPLC)	Yield (%) (isolated)
42	$Pd(AcO)_2$ (6)	DPPP (9)	1,4-dioxane	120	Citric acid	Traces	n.d.
43	$Pd(AcO)_2$ (6)	DPPP (9)	toluene	120	Citric acid	27%	48%
44	$Pd(AcO)_2$ (6)	DPPP (9)	NMP	80	Citric acid	n.d.	35%
45	$Pd(AcO)_2$ (6)	DPPP (9)	NMP	90	Citric acid	50%	68%
46	$Pd(AcO)_2$ (6)	DPPP (9)	NMP	100	Citric acid	100%	77%
47	$Pd(AcO)_2$ (6)	DPPP (9)	NMP	110	Citric acid	95%	Quant.
48[f,i]	$Pd(AcO)_2$ (6)	DPPP (9)	NMP	120	Citric acid	n.d.	55%
49[c,i,f]	$Pd(AcO)_2$ (6)	DPPP (9)	NMP	120	Citric acid	n.d.	34%
50[j]	$Pd(AcO)_2$ (10)	DPPP (15)	NMP	120	Citric acid	n.d.	80%

n.d.: not determined. [a]Reaction performed in a Schlenk tube. [b]Palladium complex **22** was observed on high-resolution mass spectrometry (HRMS). [c]Reaction performed on bromide **11**. [d]Dimethylamide **7c** was isolated with 24% yield. [e]Reaction performed on 0.06 mmol scale. [f]Reaction performed in a 0.5 - 2.0 mL microwave vial. [g]Final concentration of iodide **10** of 0.6 M. [h]Final concentration of iodide **10** of 0.1 M. [i]Reaction performed on 0.100 mmol scale. [j]Reaction performed on 1.50 mmol scale.

Table S2. Crystal data and structure refinement for 22 ·solvent.

CCDC deposition code	2278946
Empirical formula	$C_{55}H_{48}IN_3O_6P_2PdS$ [+ solvent] [a]
Formula weight	1174.26 [+ solvent] [a]
Crystal system	triclinic
Space group	P-1
a/Å	12.2881(10)
b/Å	13.7686(12)
c/Å	16.6116(15)
α/°	99.933(4)
β/°	97.976(4)
γ/°	91.227(4)
Volume/Å^3	2738.6(4)
Z	2
ρcalcg/cm^3	1.424
μ/mm-1	1.049
F(000)	1184.0
Crystal size/mm^3	0.23 × 0.14 × 0.11
2Θ range for data collection/°	3.006 to 56.564
Index ranges	-16 ≤ h ≤ 16, -18 ≤ k ≤ 18, -22 ≤ l ≤ 22
Reflections collected	70105
Independent reflections	13586 [Rint = 0.0368]
Data/restraints/parameters	13586/235/761
Goodness-of-fit on F2	1.066
Final R indexes [I>=2σ (I)]	R1 = 0.0643, wR2 = 0.1554
Final R indexes [all data]	R1 = 0.0936, wR2 = 0.1817
Largest diff. peak/hole / e Å-3	1.89/-1.40

[a] Diffuse solvent electron density (DMF and/or CH_2Cl_2) was considered using the BYPASS routine of Olex 1.2.[3]

Table S3. Effect of the temperature on the formation of methyl ester 6a.[a]

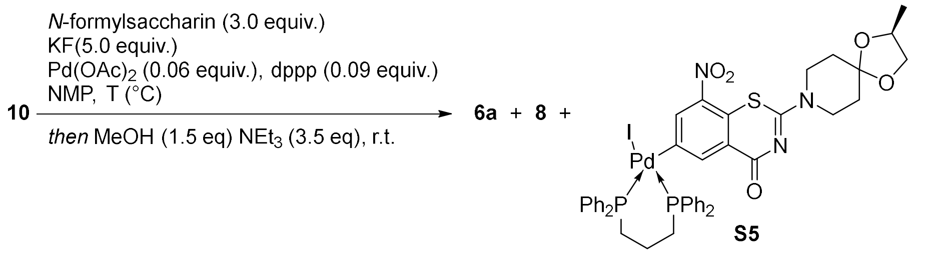

Entry	Temperature (°C)	Yield (%)[a]		
		**6a**	**8**	**S5**
1[b]	80	12	35	traces
2	85	traces	65	-
3	90	-	68	-
4	120	-	60	-

[a]Isolated yield. [b]50% of iodide **10** was recovered.

Table S4. Crystal data and structure refinement for S5 · CH_2Cl_2.

CCDC deposition code	2278948
Empirical formula	$C_{44}H_{44}Cl_2IN_3O_5P_2PdS$
Formula weight	1093.02
Crystal system	triclinic
Space group	P-1
a/Å	13.1030(12)
b/Å	13.4601(12)
c/Å	14.1658(12)
α/°	97.301(2)
β/°	92.220(2)
γ/°	116.000(2)
Volume/Å^3	2214.8(3)
Z	2
ρ_{calc}g/cm^3	1.639
μ/mm^{-1}	1.404
F(000)	1096.0
Crystal size/mm^3	0.24 × 0.12 × 0.07
2Θ range for data collection/°	3.41 to 61.016
Index ranges	$-18 \le h \le 18, -19 \le k \le 19, -20 \le l \le 20$
Reflections collected	72270
Independent reflections	13509 [R_{int} = 0.0272]
Data/restraints/parameters	13509/37/588
Goodness-of-fit on F^2	1.071

Final R indexes [I>=2σ (I)]	R_1 = 0.0371, wR_2 = 0.0854
Final R indexes [all data]	R_1 = 0.0491, wR_2 = 0.0974
Largest diff. peak/hole / e Å^{-3}	1.67/-1.95

Table S5. Complete data set of performed biological assays.

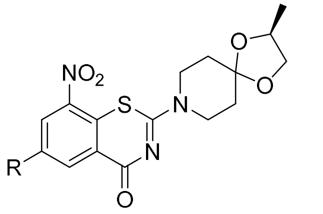

Compound	R	HMC[a]	MIC (µM)[b]		CC_{50} (µM)[c]	GI_{50} (µM)[c]		clogP[d]	LLE[e]	Selectivity index[f]
			M. vaccae	*M. smegmatis*	HeLa	HUVEC	K-562			
**1**	CF_3	1.00	0.004	0.058	>115.9	>116	>116	2.87	5.56	>31248
**8**	COOH	0.00	0.98	15.3	>122.7	>123	>123	1.33	4.68	>125
**9**	CN	11.51 ± 2.24	0.016	0.13	>128.7	49.9 ± 4.1	94.4 ± 5.9	1.58	6.22	> 8062
**10**	I	0.45 ± 0.30	0.006	0.10	>102.1	>102	>102	2.49	5.71	>16114
**11**	Br	0.95 ± 0.28	0.004	0.057	>113.0	>113	>113	2.45	5.99	>31236
**6a**	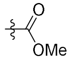	2.58 ±0.31	0.24	3.70	>118.6	80.6 ± 5.0	66.9 ± 12.6	1.72	4.90	>500
**6b**	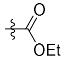	3.47 ± 0.56	1.79	57.4	>114.8	49.1 ± 1.1	50.7 ± 6.9	2.00	3.75	>64
**6c**	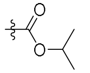	2.53 ± 0.51	0.22	6.94	>111.2	>111	>111	2.37	4.28	>500
**6d**	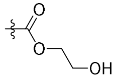	5.68 ± 0.64	13.8	55.4	>110.7	73.3 ± 4.2	17.9 ± 1.1	1.30	3.56	>8
**6e**	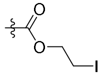	9.02 ± 0.78	2.78	44.5	78.2 ± 1.4	30.8 ± 0.5	27.6 ± 0.9	2.57	2.99	28
**6f**	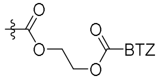	0.51 ± 0.06	14.9	118.9	>59.4	>59	>59	2.88	1.95	>4
**7a**	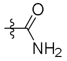	0.111±0.003	61.5	123.0	>123	>123	>123	0.92	3.29	>2

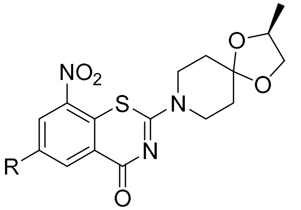

Compound	R	HMC[a]	MIC (µM)[b]		CC_{50} (µM)[c]	GI_{50} (µM)[c]		clogP[d]	LLE[e]	Selectivity index[f]
			M. vaccae	*M. smegmatis*	HeLa	HUVEC	K-562			
**7b**	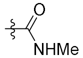	0.11 ±0.03	59.5	118.9	>118.9	>119	>119	1.31	2.92	>2
**7c**	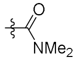	13.53 ± 3.62	28.8	230.2	>115	>115	>115	1.55	2.99	>4
**7d**	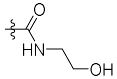	0.075 ±0.007	27.7	111.0	>110.9	>111	>111	0.90	3.66	>4
**7e**	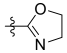	3.42 ± 0.53	14.5	28.9	>115.6	>116	>116	1.89	2.95	>8
**7f**	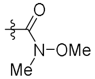	18.59 ± 8.07	13.9	111.0	>110.9	98.5 ± 3.3	>111	1.48	3.38	>8
**23**	H	0.00	1,10	8.59	>137.6	>137	>138	1.75	4.21	>125
**22**	Palladium complex	n.d.	0.021	0.66	29.5 ± 3.2	30.3 ± 3.9	17.9 ± 1.1	n.d.	n.d.	1386
**S7**	Palladium complex	n.d.	0.021	0.34	30.5 ± 1.8	34.4 ± 3.4	37.8 ± 3.2	n.d.	n.d.	1452
**S5**	Palladium complex	n.d.	0.40	12.4	16.7 ± 0.4	2.4 ± 0.2	2.0 ± 0.6	n.d.	n.d.	42

n.d. not determined. [a]Hydride Meisenheimer complex formation relative to BTZ-043 (**1**). A value of 1 means equal tendency to form hydride Meisenheimer complex as BTZ-043 (**1**). Assay was performed with RAW cells as described in our previous work.[6] Standard deviation from n=3. [b]n = 1. [c]n = 4. [d]Consensus $logP_{o/w}$ calculated on swissADME.ch.[8] [e]LLE = pMIC − clogP. LLE was calculated from the MIC against *M. vaccae.* [f]The selectivity index was determined as the ratio between the CC_{50} and the $MIC_{M.vaccae}$.

Table S6. Microsomal stability assay of most active compounds.

Compound	Group	$t_{1/2}$ (min)	$Cl_{int,micro}$ (µl.min^{-1}-mg^{-1} protein)	Cl_{int} (ml.min^{-1}.kg^{-1})	E_h (%)
**9**	Nitrile	105.02	13.3	15.4	43.5
**10**	Halide	84.53	16.1	19.1	48.8
**11**	Halide	87.74	16	18.4	47.9
**6a**	Ester	4.45	314.7	363.4	94.8
**6c**	Ester	26.46	52.9	61.1	75.3
**7c**	Amide	533.19	2.6	3	13
**7f**	Amide	147.48	9.5	11	35.5
**23**	Unsubstituted	277.26	5.1	5.8	22.5

Supplementary Note 2: Supplementary Figures

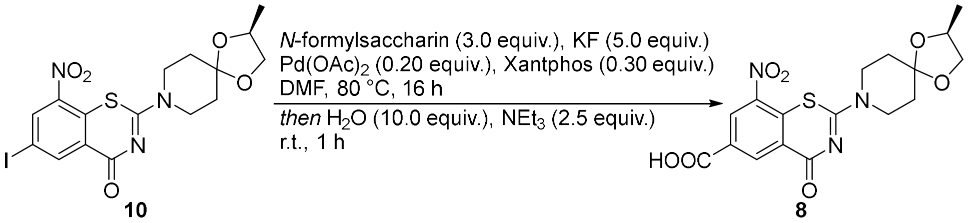

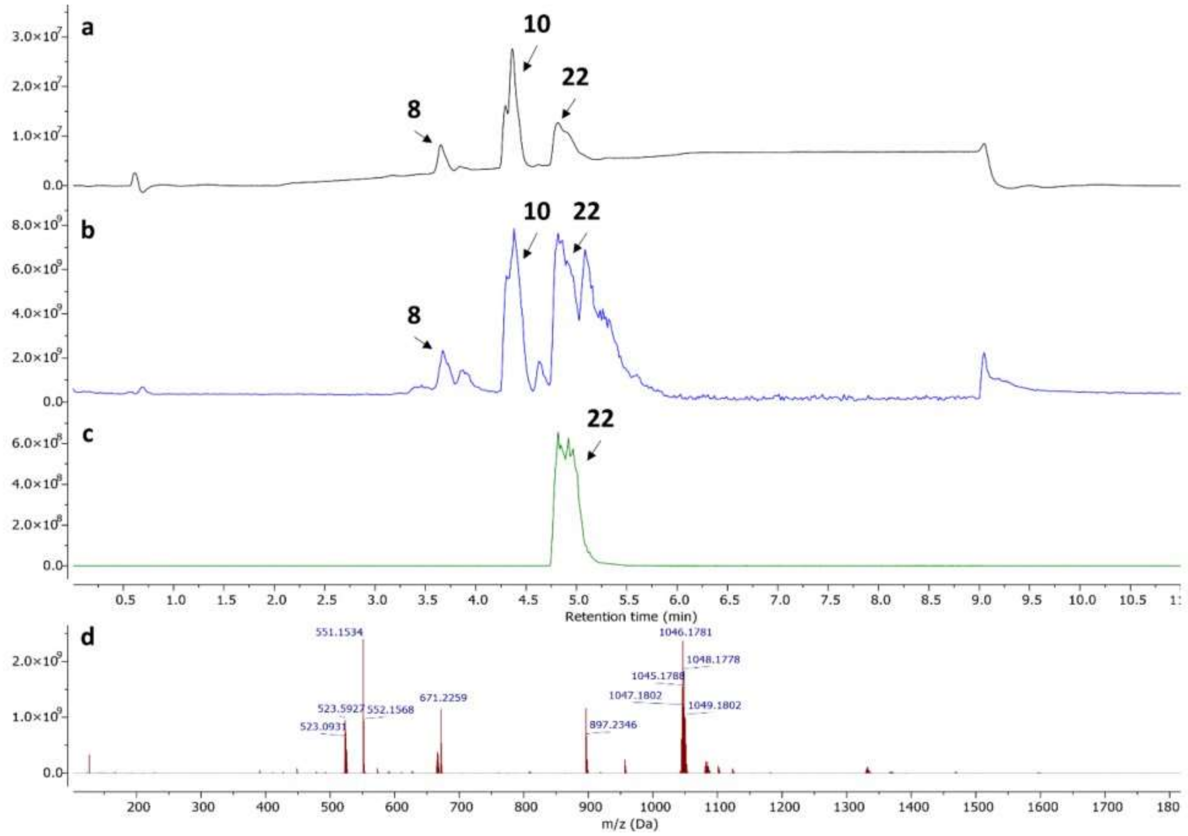

Figure S1. UHPLC-HRMS analysis of the raw extract of reaction 1 (entry 1 - Table S1). a. PDA chromatogram of the reaction mixture; b. TIC of the reaction mixture (positive mode); c. Extracted ion current chromatogram for complex **22** (positive mode – 1046.1768 ± 5 ppm); d. Mass spectrum of the peak at retention time 4.818 minutes, corresponding to complex **22**.

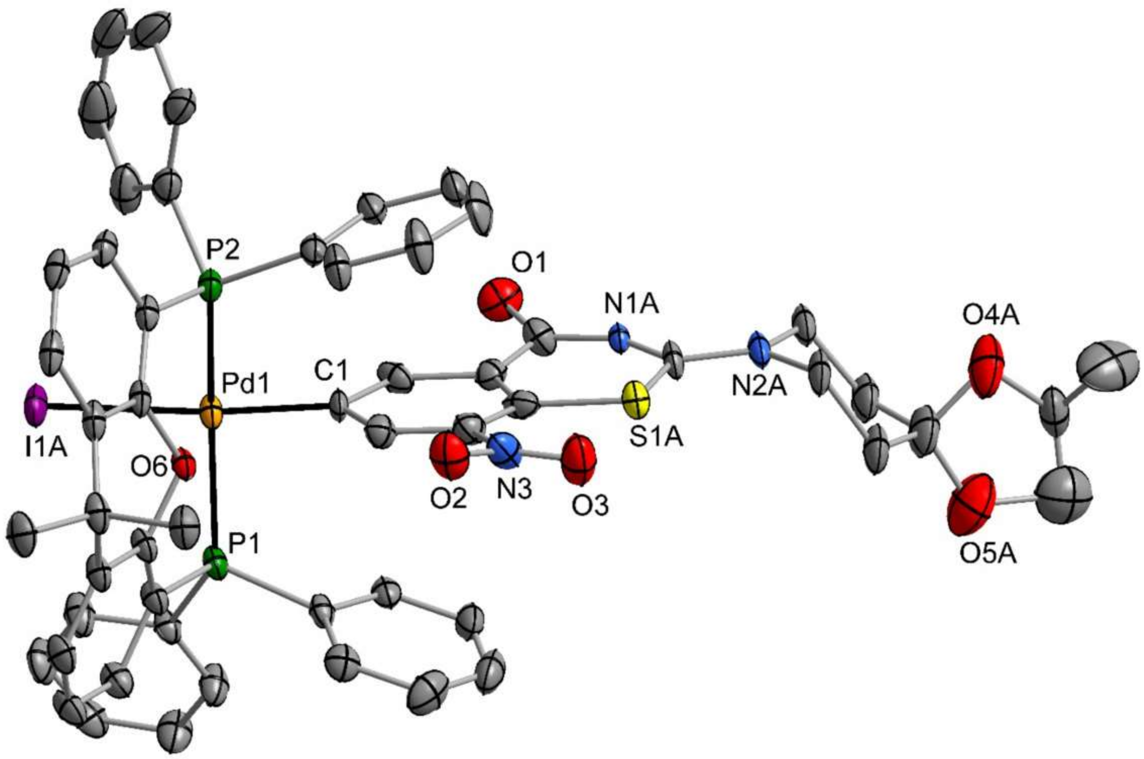

Figure S2. Molecular structure of 22. Displacement ellipsoids drawn at the 30% probability level, H atoms and disorder effects were omitted for clarity. The chiral $C_3H_3O_2Me$ residue violates the crystallographic centrosymmetry and was refined as disordered over two orientations with fixed occupational factors of 0.5. In addition, significant residual electron density was observed close to C1, which was addressed to mixed-crystal formation with a small portion of PdI_2(xantphos) (ratio of the two species refined to 0.96:0.04).

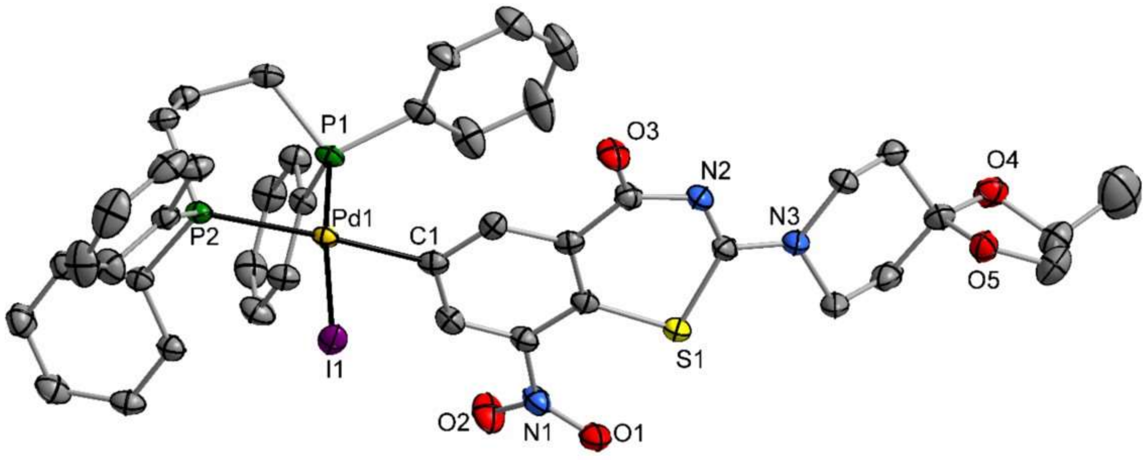

Figure S3. Molecular structure of S5, as determined from a crystalline solvate S5 · CH_2Cl_2. Displacement ellipsoids drawn at the 50% probability level; H atoms, disorder effects and solvent of crystallization omitted for clarity. The chiral $C_3H_3O_2Me$ residue violates the crystallographic centrosymmetry and was refined as disordered over two orientations with fixed occupational factors of 0.5.

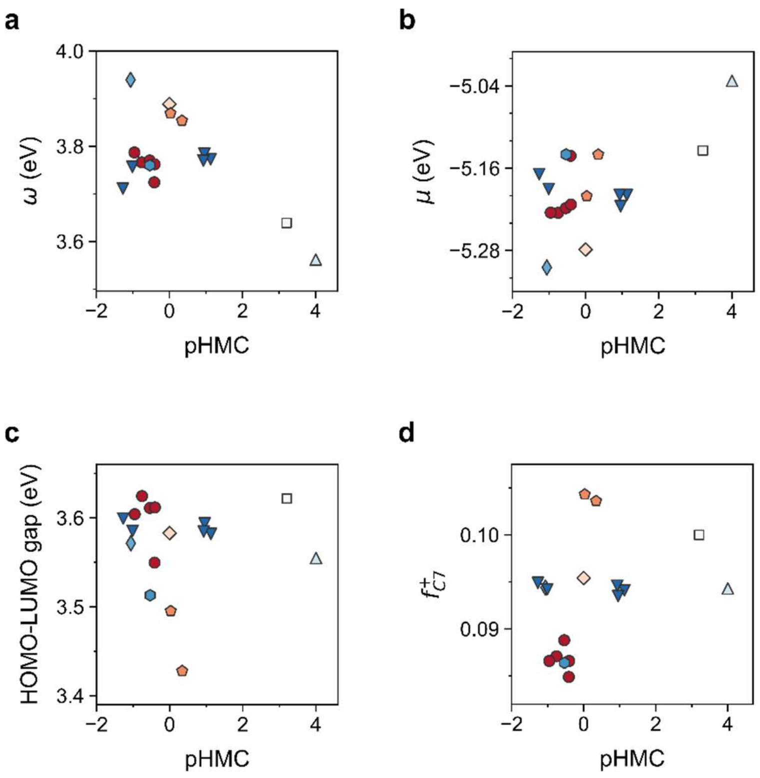

Figure S4. Global and local CDFT descriptors. a. Electrophilicity index. b. chemical potential. c. HOMO-LUMO energy gap. d. Fukui indices for C-7. Values obtained at CPCM(water)-B3LYP-D3/6-311+G(d,p)//B3LYP-D3/6-31+G(d,p) level. Different colors and markers represent different compound types (e.g. red circles for esters, dark blue triangles for amides).

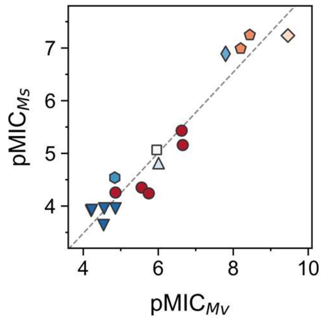

Figure S5. Comparison of antimicrobial activity of studied compounds against two *Mycobacteria* species. Different colors and markers represent different compound types (e.g. red circles for esters, dark blue triangles for amides). Mv: *M. vaccae*; Ms: *M. smegmatis*.

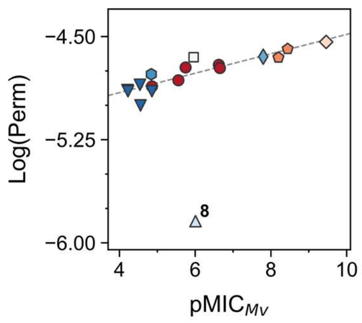

Figure S6. Predicted membrane permeability (RRCK cells). Values obtained with the membrane permeability model from the Schrödinger Suite.

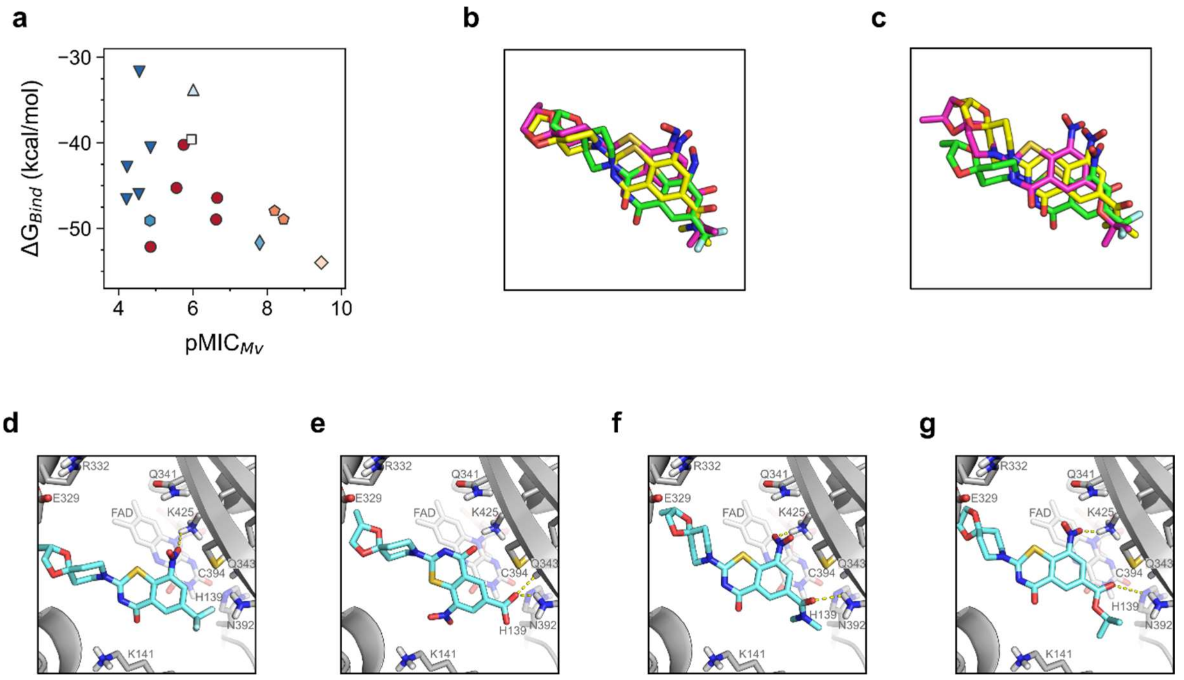

Figure S7. Compounds targeting DprE1 by molecular docking simulations. a. Free binding energy of nitroso intermediate species to DprE1 prior covalent binding by the MM/GBSA method. Different colors and markers represent different compound types (e.g. red circles for esters, dark blue triangles for amides). b. Superimposed docking poses from covalent docking of representative compounds. c. Superimposed docking poses from non-covalent docking of representative compounds on reduced DprE1. Licorice representation in green for BTZ-043 (**1**), yellow for amide **7c**, and pink for ester **6c**. Representative docking poses for d. BTZ-043 (**1**), e. acid **8**, f. ester **6c**, and g. amide **7c** (cyan licorice), showing only selected residues to ease visualization.

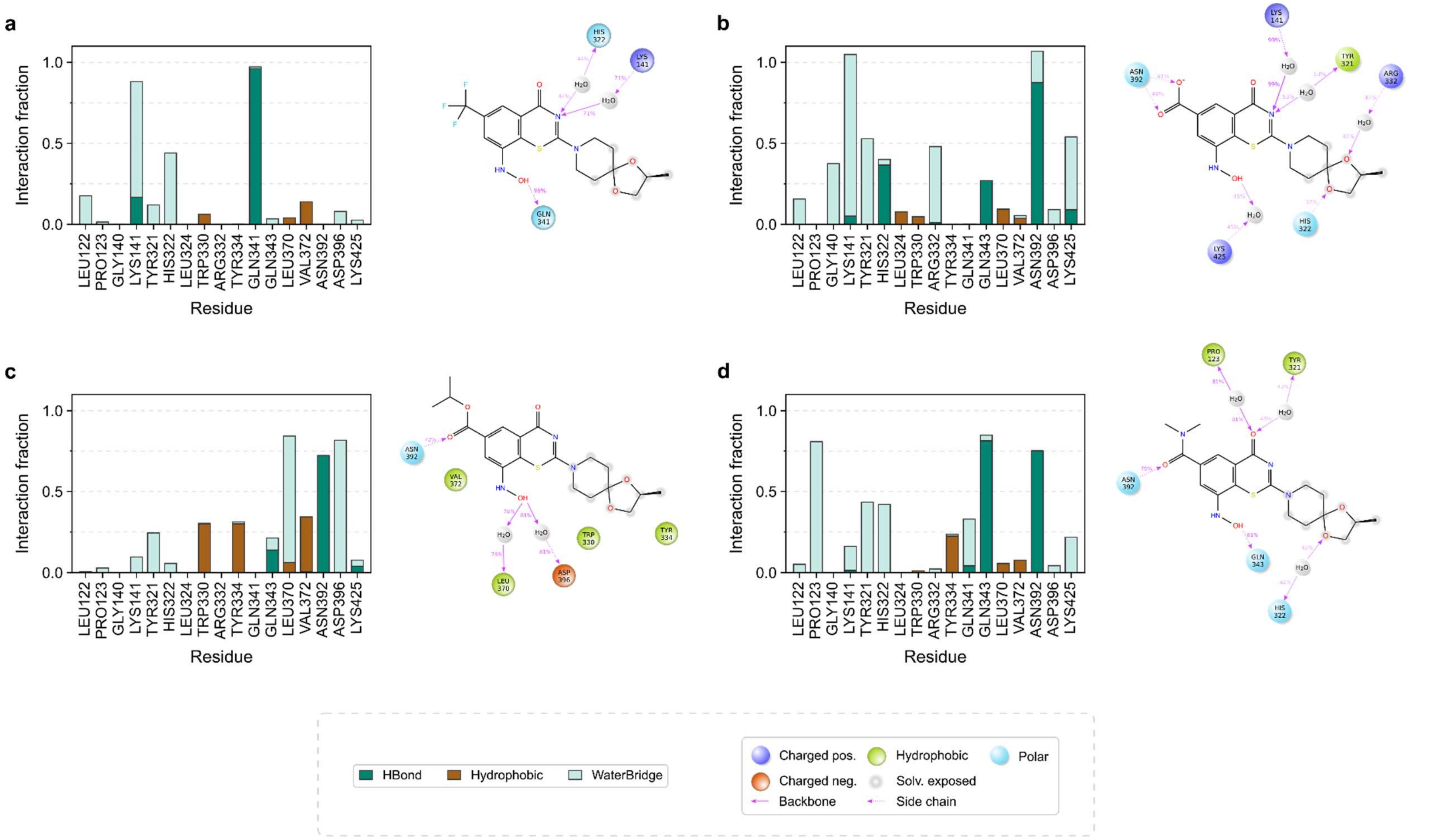

Figure S8. Protein-ligand interactions from MD simulations of the covalently bound complex for representative compounds. a. Clinical candidate **1**. b. Acid **8**. c. Ester **6c**. d. Amide **7c**. In each case, the left bar plot shows the fraction of interaction along the simulation (only residues with a fraction of at least 0.3 for one compound are shown). The right figure represents interactions occurring at least 30% of the time for each compound independently.

Supplementary Note 3: General Information

All chemicals were purchased from Acros Organics, Alfa-Aesar, Sigma-Aldrich, Hapila GmbH, Fluorochem and FisherScientific, and were used as received.

Dry solvents were purchased extra dry from Acros Organics under AcroSeal® over Molecular Sieve. Dry NMP used for reductive carbonylation was stored under nitrogen atmosphere in a glovebox.

NMR measurements were performed on a Bruker AVANCE II 300 MHz, Bruker AVANCE III 500 MHz and a Bruker AVANCE III 600 MHz spectrometer, equipped with a Bruker Cryoplatform. The chemical shifts are reported in parts per million (ppm) relative to the solvent residual peak of CD_3OD (^1H: 3.31 ppm, quintet; ^{13}C: 49.03 ppm, heptet), $CDCl_3$ (^1H: 7.26 ppm, singlet; ^{13}C: 77.16 ppm, triplet) or DMSO-d_6 (^1H: 2.50 ppm, quintet; ^{13}C: 39.52 ppm, septet).

Flash chromatography: Purifications were performed on a Teledyne Isco CombiFlash® Rf+ system. Purifications were run on packed Macherey-Nagel Chromabond® Flash BT SiOH cartridges (4 g, 15 g, 40 g, and 80 g) or on packed Macherey-Nagel Chromabond® Flash RS SPHERE SiOH 25µm cartridges (4 g) using either PE and EtOAc + 1 % FA or DCM/PE/acetone (12:12:1) + 1 % FA and DCM/PE/acetone (1:1:1) + 1 % FA, or gradients thereof as solvent.

UHPLC: Measurements were performed on a Thermo Vanquish Horizon system, equipped with a 60 mm Lightpipe diode array detector, using a Phenomenex Kinetex C8 column (100 x 2.1 mm, particle size 1.7 µm, pore diameter 100 Å).

HPLC-HRMS: Measurements were performed on a Thermo Vanquish Horizon UHPLC system coupled to a Thermo QExactive HF-X Orbitrap benchtop HRMS, using a Phenomenex Kinetex C8 column (100 x 2.1 mm, particle size 1.7 µm, pore diameter 100 Å).

Gradient of UHPLC-system: Solvent A = H_2O + 0.1 % acid, solvent B = MeCN + 0.1 % acid: 0 – 1 min: 10 % B; 1 – 5 min: 10 to 98 % B; 5 – 8 min: 98 % B; 8 – 8.1 min: 98 to 10 % B; 8.1 – 11 min: 10 % B with a flow rate of 0.400 mL/min.

For UHPLC analysis, TFA was used as acid. For HPLC-HRMS analysis, FA was used as acid.

X-Ray crystallography: The single-crystal X-ray intensity data were collected on a Bruker-Nonius Kappa-CCD diffractometer equipped with a Mo-Kα IµS microfocus source and an Apex2 CCD detector, at T = 120(2) K. The structures were solved using Intrinsic-phasing algorithms with SHELXT-2018/3[1] and refined by full matrix least-squares methods on F^2 with SHELXL-2018/3,[2] using the Olex 1.2 environment.[3] Multi-scan absorption correction was applied to the intensity data.[4]

Supplementary Method 1: Synthesis of cyanide 9, iodide 10 and bromide 11

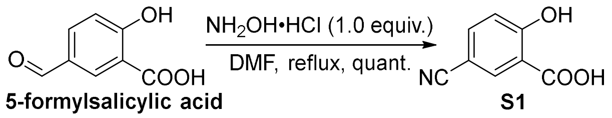

5-Cyanosalicylic acid S1. A solution of 5-formylsalicylic acid (2.10 g, 12.6 mmol) and hydroxylamine hydrochloride (878.4 mg, 12.6 mmol, 1.0 equiv.) in DMF (6 mL) was heated up and stirred at reflux for 30 minutes. The yellow clear solution was cooled to room temperature and the solvent was removed under reduced pressure. The residue was taken up in 2N HCl and filtered. The obtained solid was washed with cold water and dried for 16 hours in an oven to obtain **S1** as white solid (2.06 g, 12.6 mmol, quant.). No further purification was required for the next step.

^{1}H NMR (300 MHz, CD_3OD) δ 8.20 (d, J = 2.2 Hz, 1H), 7.77 (dd, J = 8.7, 2.2 Hz, 1H), 7.06 (d, J = 8.7 Hz, 1H); ^{13}C NMR (75 MHz, CD_3OD) δ 169.8, 164.3, 137.2, 134.5, 117.7, 117.1, 113.2, 101.5; HRMS(ESI) *m/z* [M-H]$^-$ calcd. for $C_8H_4NO_3$ 162.0197; found 162.0194.

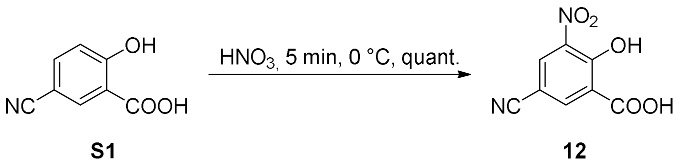

5-Cyano-3-nitrosalicylic acid 12. Fuming nitric acid (10 mL) was added to acid **S1** (1.68 g, 10.3 mmol) at 0 °C and the orange solution was stirred for 5 minutes at 0 °C. The mixture was quenched with ice water and the solid extracted with EtOAc three times. The organic layers were combined and washed once with brine, dried over $MgSO_4$, filtered and concentrated. The residue was purified by flash chromatography (0 to 100% EtOAc + 1% FA in PE) to obtain **12** as pale-yellow solid (2.14 g, 10.3 mmol, quant.).

^{1}H NMR (300 MHz, CD_3OD) δ 8.53 (d, J = 2.2 Hz, 1H), 8.48 (d, J = 2.2 Hz, 1H); ^{13}C NMR (75 MHz, CD_3OD) δ 171.4, 159.8, 140.0, 135.4, 118.5, 117.5, 103.3; HRMS(ESI) *m/z* [M-H]$^-$ calcd. for $C_8H_3N_2O_5$ 207.0047; found 207.0040.

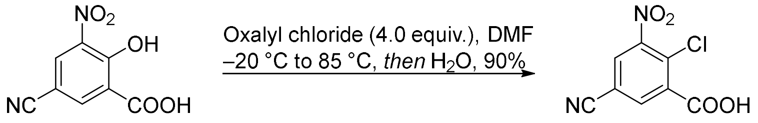

12 S2

2-Chloro-5-cyano-3-nitrobenzoic acid S2. Acid **12** (1.17 g, 5.6 mmol) was dissolved in DMF (10 mL) and cooled down to −20 °C. Oxalyl chloride (1.9 mL, 22.7 mmol, 4.0 equiv.) was added dropwise to the solution. The obtained suspension was stirred for 20 minutes at −20 °C, heated up to 85 °C and stirred for further two hours. The amber mixture was poured on ice water and the mixture was extracted three times with EtOAc. The combined organic layers were dried over Na_2SO_4, filtered and concentrated. The residue was purified by flash chromatography (0 to 100% EtOAc + 1% FA in PE) to obtain **S2** as pale amber solid (1.14g, 5.0 mmol, 90%).

^{1}H NMR (300 MHz, CD_3OD) δ 8.47 (d, *J* = 2.0 Hz, 1H), 8.40 (d, *J* = 2.0 Hz, 1H); ^{13}C NMR (75 MHz, CD_3OD) δ 163.3, 149.6, 135.7, 134.7, 129.2, 128.4, 114.5, 111.4; HRMS(ESI) *m/z* $[M\text{-}COOH]^-$ calcd. for $C_7H_2ClN_2O_2$ 180.9810; found 180.9806.

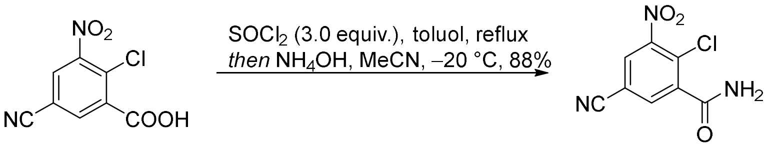

S2 15

2-Chloro-5-cyano-3-nitrobenzamide 15. Acid **S2** (1.67 g, 7.3 mmol) was dissolved in toluene (10 mL) with few drops of DMF and thionyl chloride (1.6 mL, 22.0 mmol, 3.0 equiv.) was added carefully. The yellow solution was stirred at reflux for two hours and cooled down to room temperature. The mixture was concentrated under reduced pressure and the obtained residue was dissolved in MeCN (4.2 mL) and added to an aqueous ammonia solution (35% w/w, 10 mL) at −20 °C. The yellow suspension was stirred for 10 minutes and taken up in EtOAc. The organic layer was then thoroughly washed with water until neutrality, dried over Na_2SO_4, filtered and concentrated. The residue was purified by flash chromatography (0 to 100% EtOAc in PE) to obtain **15** as yellow solid (1.45 g, 6.4 mmol, 88%).

^{1}H NMR (300 MHz, CD_3OD) δ 8.45 (d, *J* = 1.9 Hz, 1H), 8.14 (d, *J* = 1.9 Hz, 1H); ^{13}C NMR (75 MHz, CD_3OD) δ 168.2, 150.7, 141.8, 135.8, 130.4, 128.1, 116.7, 113.7; HRMS(ESI) *m/z* $[M\text{-}Cl\text{+}OH]^-$ calcd. for $C_8H_4N_3O_4$ 206.0207; found 206.0201.

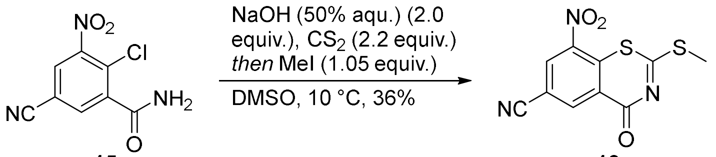

15 18

2-(Methylthio)-8-nitro-4-oxo-4*H*-benzo[*e*][1,3]thiazine-6-carbonitrile 18. In a flame-dried Schlenk tube under argon amide **15** (100.0 mg, 0.44 mmol) was solubilized in DMSO (450 µL). The clear solution was cooled to 10 °C and CS_2 (59 µL, 0.97 mmol, 2.20 equiv.) and an aqueous solution of NaOH (50% w/w, 35.5 mg, 0.89 mmol, 2.00 equiv.) were added under argon atmosphere. The reaction was stirred for one hour at 10 °C under argon atmosphere. Iodomethane (29 µL, 0.47 mmol, 1.05 equiv.) was then added and the dark red mixture was stirred further for 45 minutes at 10 °C. The sluggish reaction mixture was then cooled to 0 °C, quenched with ice water, stirred further for 30 minutes and filtered. The obtained solid was thoroughly washed with cold water and dried under vacuum to obtain **18** as orange solid (44.0 mg, 0.16 mmol, 36%). No further purification was required for the next step.
^{1}H NMR (500 MHz, DMSO-d_6) δ 9.21 (d, J = 1.9 Hz, 1H), 8.97 (d, J = 1.9 Hz, 1H), 2.75 (s, 3H); ^{13}C NMR (126 MHz, DMSO-d_6) δ 181.0, 162.7, 142.9, 139.8, 136.7, 133.2, 125.6, 116.0, 111.5, 14.5; HRMS(ESI) *m/z* [M+H]$^+$ calcd. for $C_{10}H_6N_3O_3S_2$ 279.9845; found 279.9844.

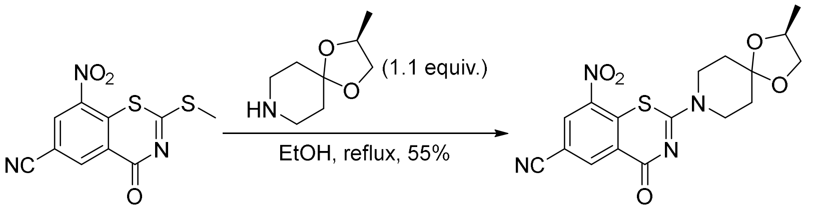

18 9

(*S*)-2-(2-Methyl-1,4-dioxa-8-azaspiro[4.5]decan-8-yl)-8-nitro-4-oxo-4*H*-benzo[*e*][1,3]thiazine-6-carbonitrile 9. Thioether **18** (44.0 mg, 0.16 mmol) was suspended in EtOH (1 mL) and (*S*)-2-methyl-1,4-dioxa-8-azaspiro[4.5]decane (**21**) (24.8 mg, 0.16 mmol, 1.0 equiv.) was added. The suspension was stirred under reflux for two hours. The mixture was cooled and filtered. The solid was washed with cold water and cold EtOH to obtain nitrile **9** as orange solid (34.2 mg, 0.09 mmol, 55%).
^{1}H NMR (600 MHz, $CDCl_3$ + 1% TMS) δ 9.06 (d, J = 1.9 Hz, 1H), 8.75 (d, J = 2.0 Hz, 1H), 4.32 – 4.27 (m, 1H), 4.21 (br s, 2H), 4.13 – 4.11 (m, 1H), 3.95 (br s, 2H), 3.52 – 3.48 (m, 1H), 1.86 (br s, 4H), 1.31 (d, J = 6.0 Hz, 3H); ^{13}C NMR (151 MHz, $CDCl_3$ + 1% TMS) δ 166.0, 161.5, 144.1, 139.5, 135.5, 131.8, 126.9, 115.7, 111.6, 106.4, 72.7, 71.1, 44.8, 36.5, 35.3, 18.4; HRMS(ESI) *m/z* [M+H]$^+$ calcd. for $C_{17}H_{17}N_4O_5S$ 389.0914; found 389.0908.

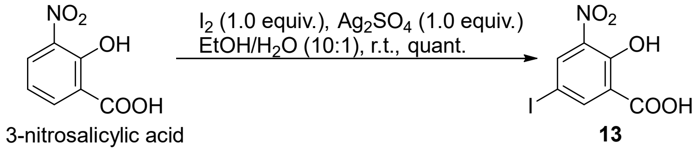

5-Iodo-3-nitrosalicylic acid 13. 3-nitrosalicylic acid (2.0 g, 10.9 mmol) was dissolved with iodine (2.8 g, 10.9 mmol, 1.0 equiv.) and silver sulfate (3.4 g, 10.9 mmol, 1.0 equiv.) in a mixture of EtOH/water (10 : 1; 65 mL). The amber suspension was stirred at room temperature and the reaction was monitored by UHPLC. At completion, the bright yellow suspension was filtered and the filtrate was concentrated. The residue was dissolved in DCM and the obtained organic layer thoroughly washed with 1 M aqueous HCl. The organic layer was then dried over Na_2SO_4, filtered and concentrated to obtain **13** as yellow solid (3.4 g, 10.9 mmol, quant.). No further purification was required for the next step.

^{1}H NMR (300 MHz, CD_3OD) δ 8.40 (d, J = 2.3 Hz, 1H), 8.37 (d, J = 2.3 Hz, 1H); ^{13}C-NMR (75 MHz, CD_3OD): δ 169.2, 154.3, 142.7, 138.4, 137.7, 117.0, 76.1; HRMS(ESI) *m/z* [M-H]$^-$ calcd. for $C_7H_3INO_5$ 307.9061; found 307.9059.

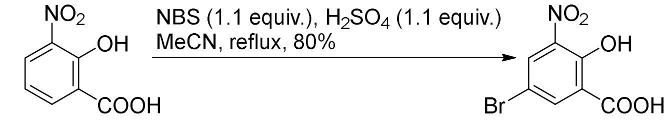

3-nitrosalicylic acid 14

5-Iodo-3-nitrosalicylic acid 14. To a solution of 3-nitrosalicylic acid (1.0 g, 5.5 mmol) in MeCN (10 mL) was added concentrated sulfuric acid (307 µL, 5.7 mmol, 1.05 equiv.) and the solution was stirred for 5 minutes at room temperature. NBS (1.0 g, 5.7 mmol, 1.05 equiv.) was added and the solution was heated up and stirred under reflux for at least one hour. The reaction was monitored by UHPLC. At completion, the solution was concentrated and the residue was purified by flash chromatography (0% to 100% EtOAc + 1% FA in PE) to obtain **14** as a yellow solid (1.2 g, 4.4 mmol, 80%).

^{1}H NMR (300 MHz, CD_3OD) δ 8.27 (d, J = 2.6 Hz, 1H), 8.26 (d, J = 2.6 Hz, 1H); ^{13}C NMR (75 MHz, CD_3OD) δ 170.6, 155.1, 139.6, 138.0, 133.4, 118.1, 109.2; HRMS(ESI) *m/z* [M-H]$^-$ calcd for $C_7H_3BrNO_5$ 259.9200; found 259.9202.

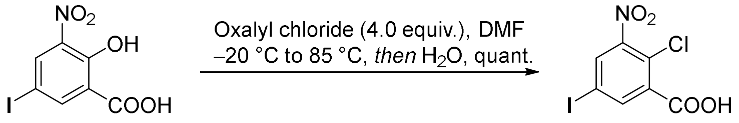

13 **S3**

2-Chloro-5-iodo-3-nitrobenzoic acid S3. Iodide **13** (3.2 mg, 10.4 mmol) was dissolved in DMF (40 mL) and cooled to −20 °C. Oxalyl chloride (3.5 mL, 41.6 mmol, 4.0 equiv.) was added dropwise to the solution. The obtained suspension was stirred for 20 minutes at –20 °C, heated up to 85 °C and stirred for further two hours. The clear amber mixture was poured on ice water and the mixture was extracted three times with EtOAc. The combined organic layers were dried over Na_2SO_4, filtered and concentrated. The residue was purified by flash chromatography (0 to 100% EtOAc + 1% FA in PE) to obtain **S3** as white solid (3.4 g, 10.4 mmol, quant.).

^{1}H NMR (300 MHz, CD_3OD) δ 8.33 (d, J = 2.1 Hz, 1H), 8.32 (d, J = 2.1 Hz, 1H); ^{13}C NMR (75 MHz, CD_3OD) δ 165.5, 151.4, 142.9, 136.3, 136.1, 125.0, 91.3; HRMS(ESI) *m/z* [M-COOH]$^-$ calcd. for $C_6H_2ClINO_2$ 281.8824; found 281.8815.

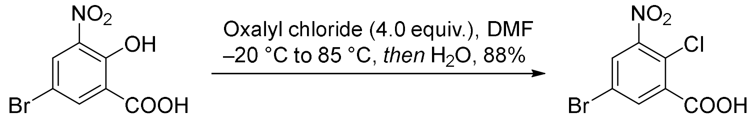

14 **S4**

5-Bromo-2-chloro-3-nitrobenzoic acid S4. Bromide **14** (900 mg, 3.4 mmol) was dissolved in DMF (17 mL) and cooled down to −20 °C. Oxalyl chloride (1.1 mL, 13.7 mmol, 4.0 equiv.) was added dropwise to the solution. The obtained suspension was stirred for 20 minutes at –20 °C, heated up to 85 °C and stirred for further two hours. The amber solution was poured on ice water and the mixture was extracted three times with EtOAc. The combined organic layers were dried over Na_2SO_4, filtered and concentrated. The residue was purified by flash chromatography (0 to 100% EtOAc + 1% FA in PE) to obtain **S4** as yellowish solid (851 mg, 3.0 mmol, 88%).

^{1}H NMR (500 MHz, CD_3OD) δ 8.22 (d, J = 2.4 Hz, 1H), 8.18 (d, J = 2.4 Hz, 1H); ^{13}C NMR (126 MHz, CD_3OD) δ 163.7, 149.7, 135.2, 134.6, 128.7, 122.5, 119.4; HRMS(ESI) *m/z* [M-COOH]$^-$ calcd. for $C_6H_2BrClNO_2$ 233.8963; found. 233.8959.

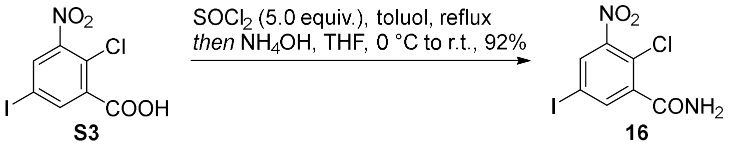

S3 **16**

2-Chloro-5-iodo-3-nitrobenzamide 16. Acid **S3** (3.4 g, 10.4 mmol) was dissolved in toluene (12.7 mL) with few drops of DMF and thionyl chloride (3.8 mL, 52.0 mmol, 5.0 equiv.) was added carefully. The yellow suspension was stirred under reflux for two hours and cooled down at room temperature. The mixture was distilled under vacuum, and the obtained residue was dissolved in THF (12.7 mL) and added to an aqueous ammonia solution (35% w/w, 22.8 mL) at 0 °C. The orange suspension was warmed up to room temperature and stirred for one hour. The solvent was removed under reduced pressure and the suspension was filtered. The obtained orange solid was thoroughly washed with cold water and dried under high vacuum to yield **16** as orange solid (3.1 g, 9.5 mmol, 92%). No further purification was required for the next step.

^{1}H NMR (500 MHz, DMSO-d_6) δ 8.45 (d, J = 2.0 Hz, 1H), 8.13 (s, 1H), 8.07 (d, J = 2.0 Hz, 1H), 7.89 (s, 1H); ^{13}C NMR (126 MHz, DMSO-d_6) δ 165.0, 149.0, 140.7, 139.6, 132.9, 121.1, 93.0; HRMS(ESI) *m/z* $[M+H]^+$ calcd. for $C_7H_5ClIN_2O_3$ 326.9028; found 326.9025.

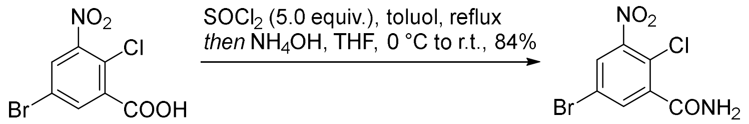

S4 **17**

5-Bromo-2-chloro-3-nitrobenzamide 17. Acid **S4** (600 mg, 2.1 mmol) was dissolved in toluene (3 mL) with few drops of DMF and thionyl chloride (777 µL, 10.7 mmol, 5.0 equiv.) was added carefully. The yellow suspension was stirred under reflux for two hours and cooled down at room temperature. The mixture was distilled under reduced pressure, and the obtained residue was dissolved in THF (3 mL) and added to an aqueous ammonia solution (35% w/w, 5.5 mL) at 0 °C. The yellow suspension was warmed up to room temperature and stirred for one hour. THF removed under reduced pressure and the suspension was filtered. The obtained bright yellow solid was thoroughly washed with cold water and dried under high vacuum to yield **17** as yellow solid (505 mg, 1.5 mmol, 84%). No further purification was required for the next step.

^{1}H NMR (500 MHz, CD_3OD) δ 8.20 (d, J = 2.3 Hz, 1H), 7.92 (d, J = 2.3 Hz, 1H); ^{13}C NMR (126 MHz, CD_3OD) δ 166.6, 148.8, 139.6, 133.3, 127.5, 120.8, 119.7; HRMS(ESI) *m/z* $[M+H]^+$ calcd. for $C_7H_5BrClN_2O_3$ 278.9167; found 278.9165.

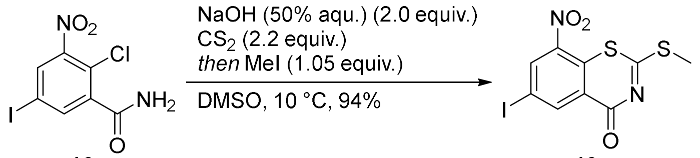

6-Iodo-2-(methylthio)-8-nitro-4*H*-benzo[*e*][1,3]thiazin-4-one 19. In a flame-dried Schlenk tube under argon amide **16** (2.4 g, 7.26 mmol) was dissolved in DMSO (11.9 mL). The clear solution was cooled to 10 °C and CS_2 (965 µL, 15.97 mmol, 2.20 equiv.) and an aqueous solution of NaOH (50% w/w, 581 mg, 14.52 mmol, 2.00 equiv.) were added under argon atmosphere. The reaction was stirred for one hour at 10 °C under argon atmosphere. Iodomethane (475 µL, 7.62 mmol, 1.05 equiv.) was added and the dark red mixture was stirred further for 45 minutes at 10 °C. The sluggish reaction mixture was then cooled down at 0 °C, quenched with ice water and stirred further for 30 minutes prior to filtration. The obtained solid was thoroughly washed with cold water and dried under reduced pressure to obtain **19** as yellow solid (2.6 g, 6.80 mmol, 94%). No further purification was required for the next step.

^{1}H NMR (300 MHz, DMSO-d_6) δ 8.91 (d, J = 2.0 Hz, 1H), 8.83 (d, J = 2.0 Hz, 1H), 2.72 (s, 3H); ^{13}C NMR (75 MHz, DMSO-d_6) δ 180.9, 162.7, 144.5, 142.7, 137.9, 131.2, 125.6, 93.5, 14.4; HRMS(ESI) *m/z* $[M+H]^+$ calcd. for $C_9H_6IN_2O_3S_2$ 380.8859; found 380.8851.

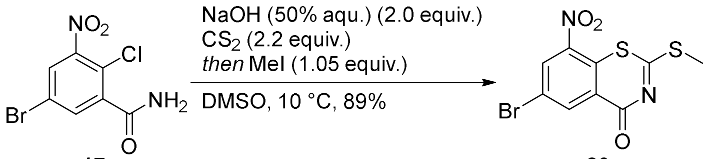

6-Bromo-2-(methylthio)-8-nitro-4*H*-benzo[*e*][1,3]thiazin-4-one 20. In a flame-dried Schlenk tube under argon amide **17** (400.0 mg, 1.43 mmol) was dissolved in DMSO (2.5 mL). The clear solution was cooled to 10 °C and CS_2 (191 µL, 3.16 mmol, 2.21 equiv.) and an aqueous solution of NaOH (50% w/w, 114.5 mg, 2.86 mmol, 2.20 equiv.) were added under an argon atmosphere. The reaction was stirred for one hour at 10 °C under argon atmosphere. Iodomethane (94 µL, 1.50 mmol, 1.05 equiv.) was then added and the dark red mixture was stirred further for 45 minutes at 10 °C. The sluggish suspension was cooled to 0 °C, quenched with ice water and stirred further for 30 minutes prior to filtration. The obtained solid was thoroughly washed with cold water and dried under reduced pressure to obtain **20** as orange solid (425.2 mg, 1.27 mmol, 89%). No further purification was required for the next step.

^{1}H NMR (500 MHz, $CDCl_3$ + 1% TMS) δ 8.91 (d, J = 2.2 Hz, 1H), 8.73 (d, J = 2.3 Hz, 1H), 2.75 (s, 3H); ^{13}C NMR (126 MHz, $CDCl_3$ + 1% TMS) δ 182.2, 163.4, 143.0, 140.2, 132.8, 131.5, 126.5, 121.8, 14.9; HRMS(ESI) *m/z* $[M+H]^+$ calcd. for $C_9H_6BrN_2O_3S_2$ 332.8998; found 332.8997.

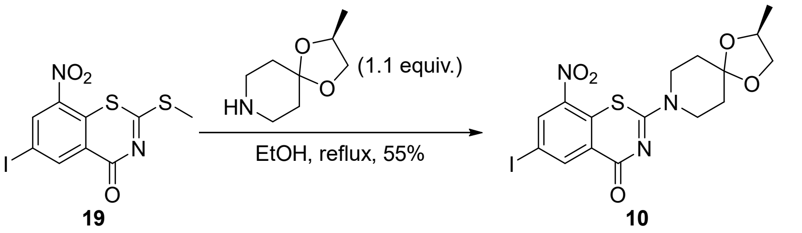

(*S*)-6-Iodo-2-(2-methyl-1,4-dioxa-8-azaspiro[4.5]decan-8-yl)-8-nitro-4*H*-benzo[*e*][1,3]thiazin-4-one 10. Thioether **19** (2.7 g, 7.1 mmol) was suspended in EtOH (28 mL) and (*S*)-2-methyl-1,4-dioxa-8-azaspiro[4.5]decane (**21**) (1.2 g, 7.8 mmol, 1.1 equiv.) was added. The suspension was stirred under reflux for 2 hours. The mixture was cooled and filtrated. The solid was washed extensively with cold water and briefly with cold EtOH to obtain iodide **10** as yellow solid (1.9 g, 3.9 mmol, 55%).

^{1}H NMR (300 MHz, DMSO-*d₆*) δ 8.77 (s, 2H), 4.33 – 4.20 (m, 1H), 4.12 – 4.07 (m, 1H), 3.91 (br s, 4H), 3.45 (t, J = 7.7 Hz, 1H), 1.83 – 1.76 (m, 4H), 1.22 (d, J = 6.0 Hz, 3H); ^{13}C NMR (75 MHz, DMSO-d_6) δ 165.0, 161.3, 144.0, 143.6, 137.2, 129.2, 125.8, 106.2, 92.1, 71.8, 70.0, 44.0, 35.6, 34.4, 18.4; HRMS(ESI) *m/z* [M+H]$^+$ calcd. for $C_{16}H_{17}IN_3O_5S$ 489.9928; found 489.9928.

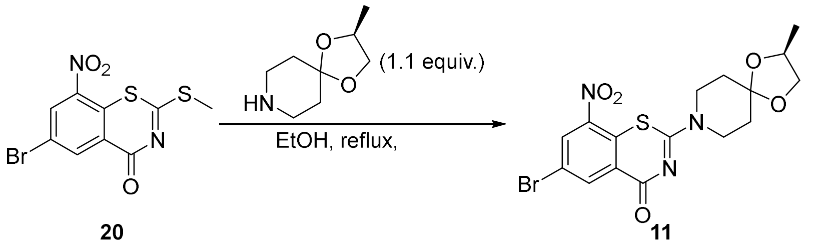

(*S*)-6-Bromo-2-(2-methyl-1,4-dioxa-8-azaspiro[4.5]decan-8-yl)-8-nitro-4*H*-benzo[*e*][1,3]thiazin-4-one 11. Thioether **20** (300 mg, 0.90 mmol) was suspended in EtOH (4.5 mL) and (*S*)-2-methyl-1,4-dioxa-8-azaspiro[4.5]decane (**21**) (155.7 mg, 0.99 mmol, 1.10 equiv.) was added. The suspension was stirred under reflux for 2 hours. The mixture was cooled and filtered. The solid was washed extensively with cold water and briefly with cold EtOH to obtain bromide **11** as yellow solid (228.1 mg, 0.52 mmol, 57%).

^{1}H NMR (300 MHz, $CDCl_3$) δ 8.97 (d, J = 2.1 Hz, 1H), 8.64 (d, J = 2.3 Hz, 1H), 4.34 – 4.24 (m, 1H), 4.14 – 4.09 (m, 1H), 4.00 (br s, 4H), 3.50 (t, J = 7.9 Hz, 1H), 1.85 (br s, 4H), 1.31 (d, J = 6.0 Hz, 3H); ^{13}C NMR (75 MHz, $CDCl_3$) δ 166.5, 162.2, 144.2, 139.5, 132.2, 129.1, 126.7, 120.8, 106.4, 72.5, 70.9, 44.5, 36.4, 35.2, 18.3; HRMS(ESI) *m/z* [M+H]$^+$ calcd. for $C_{16}H_{17}BrN_3O_5S$ 442.0067; found 442.0070.

Supplementary Method 2: Reductive carbonylation condition screening

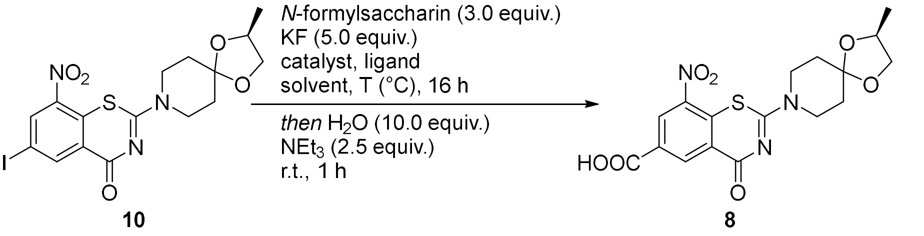

1. General screening conditions for carboxylic acid 8

In a nitrogen-filled glovebox, a flame-dried 0.1 – 0.5 mL Biotage® microwave vial equipped with a magnetic stir bar was charged with iodide **10** (10 mg, 0.02 mmol, 1.00 equiv.), ligand, *N*-formylsaccharin (12.9 mg, 0.06 mmol, 3.00 equiv.) and KF (5.9 mg, 0.10 mmol, 5.00 equiv.). Solvent (100 µL) and a solution of catalyst in the solvent system (40 µg/µL) were added. The vial was capped, removed from the glovebox, and heated using a heating block. The mixture was stirred for 16 hours and cooled to room temperature. The vial was sealed off in the glovebox. Water (0.20 mmol, 10.00 equiv.) and NEt_3 (0.05 mmol, 2.50 equiv.) were added and the vial was capped again. The reaction mixture was stirred for one hour at room temperature and quenched either with water or aqueous citric acid (10% w/w). The aqueous phase was extracted three times with EtOAc and the obtained organic layers were combined and concentrated.

UHPLC-yield: The organic residue was dissolved in 1 mL of MeOH and 1 mL of MeCN. 10 µL were taken up in 990 µL MeCN and the solution was analyzed by UHPLC. Peak area of carboxylic acid **8** was measured, and yield determined *via* an external calibration curve.

Isolated yield: The organic residue was purified by flash chromatography (gradient from 12:12:1 to 1:1:1 DCM/PE/acetone + 1% FA) to yield **8** as a yellow solid.

2. Reductive carbonylation on larger scale (entry 50 of Table S1)

In a nitrogen-filled glovebox, iodide **10** (750 mg, 1.53 mmol), palladium acetate (34.4 mg, 0.15 mmol, 0.10 equiv.), dppp (94.8 mg, 0.23 mmol, 0.15 equiv.), *N*-formylsaccharin (809.3 mg, 3.83 mmol, 2.50 equiv.) and KF (445.3 mg, 7.66 mmol, 5.00 equiv.) were suspended in NMP (7.5 mL) in a flame-dried 10 – 20 mL Biotage® microwave vial equipped with a magnetic stir bar. The vial was capped, removed from the glovebox and heated up at 120 °C. The yellow suspension was stirred for 16 hours at 120 °C. The mixture was then cooled to room temperature and the vial was sealed off in the glovebox. Water (276 µL, 15.33 mmol, 10.00 equiv.) and NEt_3 (517 µL, 3.83 mmol, 2.50 equiv.) were added and the vial was capped again. The reaction mixture was stirred for one hour at room temperature before being quenched with aqueous citric acid (10% w/w). The suspension was filtered through Celite® and the Celite® pad was washed three times with EtOAc and three times with aqueous citric acid (10% w/w). The two phases of the filtrate were separated in a separatory funnel and the aqueous phase was further extracted with EtOAc. The combined organic layers were dried over Na_2SO_4, filtered and concentrated. The obtained residue was purified by flash chromatography (gradient from 12:12:1 to 1:1:1 DCM/PE/acetone + 1% FA) to obtain **8** as yellow solid (496.6 mg, 1.22 mmol, 80%).

^{1}H NMR (500 MHz, DMSO-d_6) δ 13.95 (s, 1H), 8.98 (d, J = 2.0 Hz, 1H), 8.84 (d, J = 2.0 Hz, 1H), 4.29 – 4.23 (m, 1H), 4.11 – 4.09 (m, 1H), 3.92 (br s, 4H), 3.45 (t, J = 7.8 Hz, 1H), 1.80 (br s, 4H), 1.23 (d, J = 6.0 Hz, 3H); ^{13}C NMR (126 MHz, DMSO-d_6) δ 165.7, 164.6, 161.1, 143.8, 135.2, 133.9, 129.5, 129.2, 125.1, 106.1, 71.8, 70.0, 43.9, 35.6, 34.4, 18.3; HRMS (ESI) m/z [M-H]$^-$ calcd. for $C_{17}H_{16}N_3O_7S$: 406.0714; found: 406.0715.

3. Reductive carbonylation towards methyl ester 6a

In a nitrogen-filled glovebox, a flame-dried 0.1 – 0.5 mL Biotage® microwave vial equipped with a magnetic stir bar was charged with iodide **10** (10 mg, 0.02 mmol, 1.00 equiv.), dppp (0.76 mg, 0.002 mmol, 0.09 equiv.), *N*-formylsaccharin (12.9 mg, 0.06 mmol, 3.00 equiv.) and KF (5.9 mg, 0.10 mmol, 5.00 equiv.). NMP (93 µL) and a solution of palladium acetate in dry NMP (40 µg/µL, 7 µL, 0.001 mmol, 0.06 equiv.) were added. The vial was capped, removed from the glovebox and heated up via a heating block. The reaction mixture was stirred for 16 hours, cooled to room temperature and sealed off in the glovebox. MeOH (1.6 µL, 0.05 mmol, 2.50 equiv.) and NEt_3 (9.7 µL, 0.07 mmol, 3.50 equiv.) were added and the vial was capped again. The reaction mixture was stirred for one hour at room temperature and quenched with aqueous citric acid (10% w/w). The aqueous phase was extracted three times with EtOAc and the obtained organic layers were combined and concentrated. The organic residue was purified by flash chromatography (gradient from 12:12:1 to 1:1:1 DCM/PE/acetone + 1% formic acid) to yield ester **6a** and carboxylic acid **8**.

Note: At 80 °C, oxidative addition complex S5 was observed (entry 1, Table S3). Synthesis was performed to confirm structure (see section F.2 – Figure S3 – Table S4. Crystal data and structure refinement for S5 · CH_2Cl_2.Table S4).

4. Dehalogenation and isolation of compound 23

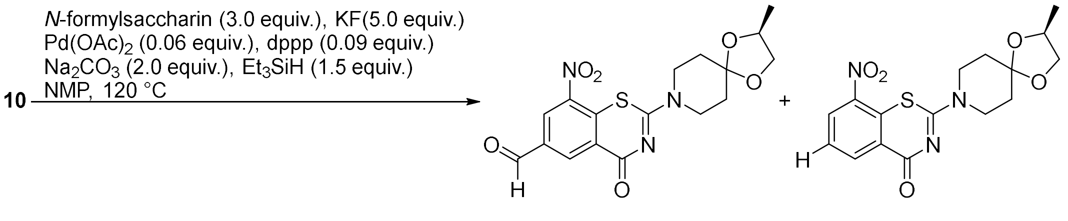

In a nitrogen-filled glovebox, a flame-dried 0.1 – 0.5 mL Biotage® microwave vial equipped with a magnetic stir bar was charged with iodide **10** (100 mg, 0.20 mmol, 1.00 equiv.), dppp (7.6 mg, 0.02 mmol, 0.09 equiv.), *N*-formylsaccharin (12.9 mg, 0.06 mmol, 3.00 equiv.) and Na_2CO_3 (43.3 mg, 0.41 mmol, 2.00 equiv.). NMP (880 µL), triethylsilane (49 µL, 0.31 mmol, 1.50 equiv.) and a solution of palladium acetate in dry NMP (40 µg/µL, 69 µL, 0.01 mmol, 0.06 equiv.) were added. The vial was capped, removed from the glovebox and heated up *via* a heating block to 120 °C and the orange suspension was stirred for 16 hours. The mixture was then cooled to room temperature and the vial was sealed off. The amber solution was diluted with DCM and washed three times with water. The combined aqueous layers were extracted three times with DCM. The organic layers were combined, dried over Na_2SO_4, filtered and concentrated. The organic residue was purified by flash chromatography (gradient from 12:12:1 to 1:1:1 DCM/PE/acetone) to yield **23** as orange oil (22.0 mg, 0.06 mmol, 30%). Formation of **S6** was not observed.

Note: Formation of **23** is in accord to observation made by Ueda et al., as electron-withdrawing groups tend to promote reductive dehalogenation.[5]

^{1}H NMR (500 MHz, $CDCl_3$) δ 8.85 (dd, *J* = 7.8, 1.6 Hz, 1H), 8.55 (dd, *J* = 8.2, 1.5 Hz, 1H), 7.60 (t, *J* = 8.0 Hz, 1H), 4.32 – 4.26 (m, 1H), 4.17 (br s, 2H), 4.13 – 4.10 (m, 1H), 3.96 (br s, 2H), 3.50 (t, *J* = 7.9 Hz, 1H), 1.86 (s, 2H), 1.83 (s, 2H), 1.31 (d, *J* = 6.1 Hz, 3H); ^{13}C NMR (126 MHz, $CDCl_3$) δ 168.1, 162.7, 143.8, 137.1, 130.5, 129.6, 127.0, 125.7, 106.6, 72.6, 71.0, 44.5, 36.5, 35.4, 18.5; HRMS (ESI) m/z $[M+H]^+$ calcd. for $C_{16}H_{18}N_3O_5S$: 364.0962; found: 364.0962.

Supplementary Method 3: Palladium complex formation and single crystal structure determination

1. Synthesis of palladium complex (xantphos)PdIBTZ 22 and (xantphos(O))PdIBTZ S7

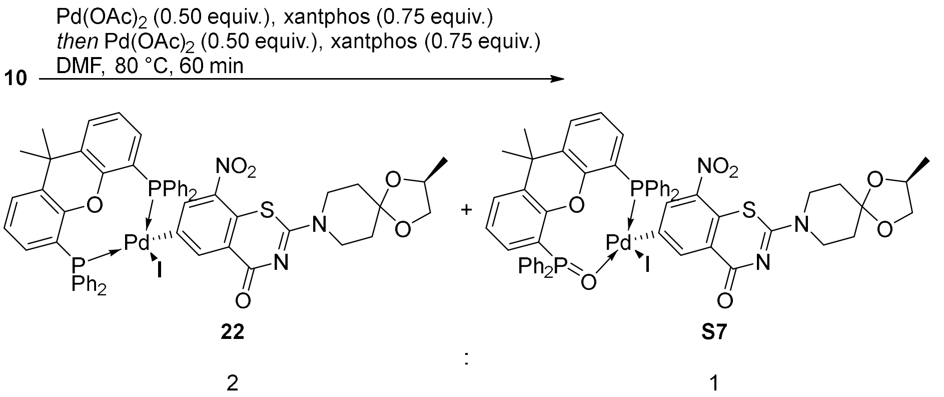

Iodide **10** (200.0 mg, 0.41 mmol), palladium acetate (45.9 mg, 0.20 mmol, 0.50 equiv.) and xantphos (177.4 mg, 0.31 mmol, 0.75 equiv.) were added to an argon flushed flame-dried Schlenk tube. Dry DMF (1 mL) was added and the yellow mixture was stirred at 80 °C under argon atmosphere. The reaction was monitored by UHPLC. After 30 minutes, consumption of iodide **10** stopped, and palladium acetate (45.9 mg, 0.20 mmol, 0.50 equiv.) and xantphos (177.4 mg, 0;31 mmol, 0.75 equiv.) were added again. Reaction was further monitored by UHPLC and iodide **10** was fully consumed after 30 minutes. The mixture was filtered through Celite®, and the Celite® pad was washed with DCM. The filtrate was concentrated under reduced pressure and the residue was purified twice by flash chromatography (gradient from 12:12:1 to 1:1:1 DCM/PE/acetone) in order to obtain complex **22** (200 mg, 0.17 mmol, 42%) and partially oxidized complex **S7** (100.0 mg, 0.08 mmol, 21%) as pure fractions, and as yellow and brown oils, respectively.

To initiate crystallization of **22**, a fraction was dissolved in a minimum volume of DCM and added to a 4 mL HPLC vial. Hexane was added slowly with a syringe to obtain a biphasic mixture of hexane/DCM. The vial was sealed, and the seal pierced with a needle. The biphasic system was let to rest, and crystal formation was observed. The crystals were grown over a week prior to analysis.

Crystallization of **S7** was initiated by solubilizing the complex in a minimum volume of DMF and disposed in a 4 mL HPLC vial. Hexane was added slowly with a syringe to obtain a biphasic mixture of hexane/DMF. The vial was sealed, and the biphasic system was let to rest. The crystals were let to grow over a week before analysis.

Note: Complex **22** tends to oxidize to complex **S7** when stored over longer periods at room temperature. The solution then turns from clear yellow to dark brown.

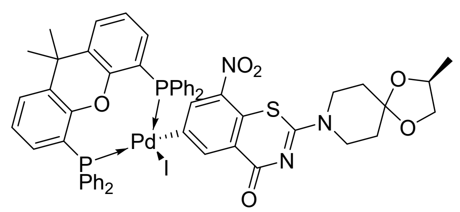

22

^{1}H NMR (500 MHz, $CDCl_3$ + 1% TMS) δ 8.29 (br s, 3H), 8.24 – 8.22 (m, 1H), 7.65 (dd, J = 7.6, 1.6 Hz, 2H), 7.55 – 7.44 (m, 6H), 7.31 – 7.27 (m, 4H), 7.19 (t, J = 7.6 Hz, 2H), 6.88 (br s, 3H), 6.76 (br s, 4H), 6.46 (br s, 3H), 4.31 – 4.25 (m, 1H), 4.12 – 4.09 (m, 1H), 3.97 (br s, 4H), 3.49 (t, J = 7.9 Hz, 1H), 1.98 (s, 3H), 1.83 – 1.79 (m, 4H), 1.68 (s, 3H), 1.30 (d, J = 6.0 Hz, 3H); ^{13}C NMR (126 MHz, $CDCl_3$ + 1% TMS) δ 168.9, 164.1, 163.1, 155.2 (t, J = 5.5 Hz), 141.2, 138.5, 136.6 (t, J = 6.4 Hz), 135.1, 134.9, 132.3 – 132.1 (m), 131.7 – 131.5 (m), 130.5, 128.5, 127.7, 127.4, 124.5, 122.7 (t, J = 22.9 Hz), 122.5, 119.7, 106.7, 72.4, 36.4, 70.8, 43.9, 36.2, 35.2, 32.9, 23.4, 18.4; ^{31}P NMR (202 MHz, DMSO-D6-d_6) 10.1; HRMS (ESI) m/z $[M-I]^+$ calcd. for $C_{55}H_{48}N_3O_6P_2PdS$: 1046.1768; found: 1046.1764.

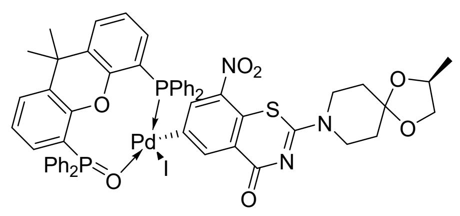

S7

^{1}H NMR (300 MHz, $CDCl_3$ + 1% TMS) δ 8.17 (s, 1H), 7.83 – 7.78 (m, 2H), 7.60 (s, 1H), 7.46 – 7.40 (m, 6H), 7.22 (br s, 6H), 7.18 – 7.12 (m, 3H), 7.11 – 7.08 (m, 3H), 7.04 – 6.95 (m, 4H), 6.86 – 6.81 (m, 2H), 4.30 – 4.23 (m, 1H), 4.10 – 4.07 (m, 1H), 3.94 (br s, 4H), 3.47 (t, J = 7.9 Hz, 1H), 1.87 (br s, 6H), 1.80 – 1.74 (m, 4H), 1.29 (d, J = 6.0 Hz, 3H); ^{13}C NMR (126 MHz, $CDCl_3$ + 1% TMS) δ 168.4, 162.9, 154.5, 152.7, 145.8, 140.7, 138.3, 138.0, 134.2, 133.8 (d, J = 13.4 Hz), 133.3, 133.1 (d, J = 10.2 Hz), 132.6 (d, J = 3.8 Hz), 132.4, 132.4, 131.9, 131.5, 131.1, 130.9, 130.6, 130.2, 129.9, 128.5, 128.4, 128.2, 128.1, 124.4, 124.3 (d, J = 7.3 Hz), 123.4, 120.8, 117.8, 117.0, 115.9, 115.6, 106.9, 72.5, 70.9, 43.9, 36.5, 35.3, 31.3, 18.5; ^{31}P NMR (202 MHz, $CDCl_3$ + 1% TMS) δ 41.3 (q, J = 11.7 Hz), 11.8 (q, J = 10.6 Hz); HRMS (ESI) m/z $[M-I]^+$ calcd. for $C_{55}H_{48}N_3O_7P_2PdS$: 1062.1718; found: 1062.1719.

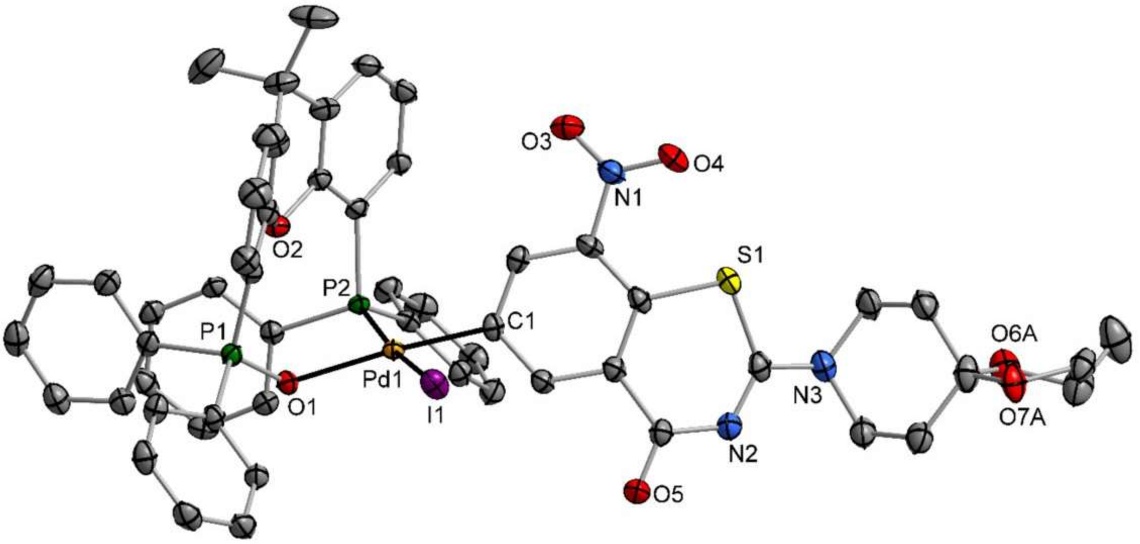

Figure S9. Molecular structure of S7, as determined from a crystalline solvate S7 · 2 DMF. Displacement ellipsoids drawn at the 50% probability level; H atoms, disorder effects and solvent of crystallization omitted for clarity. The chiral $C_3H_3O_2Me$ residue violates the crystallographic centrosymmetry and was refined as disordered over two orientations with fixed occupational factors of 0.5.

Table S7. Crystal data and structure refinement for S7 ·2 DMF.

CCDC deposition code	2278947
Empirical formula	$C_{61}H_{62}IN_5O_9P_2PdS$
Formula weight	1336.45
Crystal system	triclinic
Space group	P-1
a/Å	11.6745(8)
b/Å	13.3284(9)
c/Å	18.6559(13)
α/°	81.132(3)
β/°	89.824(3)
γ/°	86.919(3)
Volume/Å^3	2864.0(3)
Z	2
ρ_{calc}g/cm^3	1.550
μ/mm^{-1}	1.018
F(000)	1360.0
Crystal size/mm^3	0.16 × 0.13 × 0.07
2Θ range for data collection/°	3.494 to 56.564
Index ranges	-15 ≤ h ≤ 15, -17 ≤ k ≤ 17, -24 ≤ l ≤ 24
Reflections collected	74083
Independent reflections	14219 [R_{int} = 0.0331]
Data/restraints/parameters	14219/62/793
Goodness-of-fit on F^2	1.079
Final R indexes [I>=2σ (I)]	R_1 = 0.0323, wR_2 = 0.0708
Final R indexes [all data]	R_1 = 0.0453, wR_2 = 0.0792
Largest diff. peak/hole / e Å^{-3}	1.20/-0.64

2. Synthesis of palladium complex (dppp)PdIBTZ S5

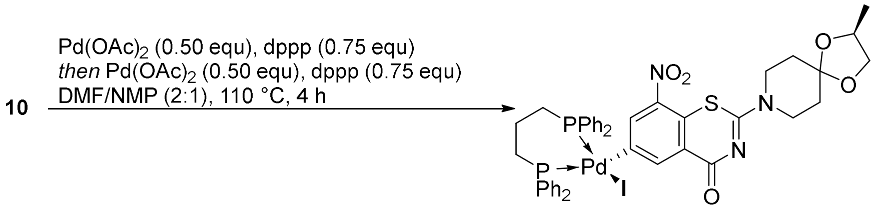

S5

Iodide **10** (100.0 mg, 0.20 mmol), palladium acetate (22.9 mg, 0.10 mmol, 0.50 equiv.) and dppp (63.2 mg, 0.15 mmol, 0.75 equiv.) were added to an argon flushed flame-dried Schlenk. DMF (500 µL) and NMP (250 µL) were added and the yellow mixture was stirred at 110 °C under an argon atmosphere. The reaction was monitored by UHPLC. After 2 hours, consumption of iodide **10** stopped, and palladium acetate (22.9 mg, 0.10 mmol, 0.50 equiv.) and dppp (63.2 mg, 0.15 mmol, 0;75 equiv.) were added again. The reaction was further monitored by UHPLC and iodide **10** was fully consumed after further 2 hours. The mixture was filtered through Celite®, and the Celite® pad was washed with DCM. The filtrate was thoroughly washed with water and brine, dried over Na_2SO_4, and concentrated under vacuum. The residue was purified twice by flash chromatography (gradient from 12:12:1 to 1:1:1 DCM/PE/acetone) to obtain complex **S5** (40 mg, 0.04 mmol, 39%) as yellow oil.

^{1}H NMR (500 MHz, $CDCl_3$ + 1% TMS) δ 8.58 (dt, *J* = 7.5, 1.6 Hz, 1H), 8.05 (dt, *J* = 8.0, 1.6 Hz, 1H), 7.79 – 7.99 (m, 5H), 7.46 – 7.39 (m, 7H), 7.32 – 7.28 (m, 2H), 7.25 – 7.22 (m, 2H), 7.14 (br s, 4H), 4.29 – 4.23 (m, 1H), 4.10 – 4.07 (m, 1H), 3.95 (br s, 4H), 3.49 – 3.45 (m, 1H), 2.62 – 2.58 (m, 2H), 2.43 (br s, 2H), 2.04 – 1.88 (m, 2H), 1.83 – 1.74 (m, 4H), 1.29 (d, *J* = 6.0 Hz, 3H); ^{13}C NMR (126 MHz, $CDCl_3$ + 1% TMS) δ 168.89, 163.10, 159.25, 158.14, 145.16, 142.81, 141.40 (d, *J* = 9.8 Hz), 137.72, 133.68, 132.79, 131.39, 130.75, 129.29, 128.78, 126.66, 124.20, 123.42, 121.35 (d, *J* = 8.5 Hz), 106.84, 92.62, 72.46, 70.90, 43.94, 36.47, 35.31, 27.04 (dd, *J* = 26.5, 6.6 Hz), 26.37 (dd, *J* = 23.8, 4.6 Hz), 19.01, 18.47; HRMS (ESI) m/z $[M-I]^+$ calculated for $C_{43}H_{42}N_3O_5P_2PdS$: 880.1350; found: 880.1348.

Note: The complex tends to decompose in solution.

To initiate crystallization, the oil was dissolved in a minimum volume of DCM and added to a 1.5 mL HPLC vial. *n*-Hexane was added slowly with a syringe to obtain a biphasic mixture of *n*-hexane/DCM. The vial was sealed, and the seal pierced with a needle. The biphasic system was stored and crystal formation was observed. The crystals were grown over a week prior to analysis.

Supplementary Method 4: Synthesis of the carboxylic acid derivative library

1. General Procedure A – Steglich esterification

Acid **8** (50 mg, 0.12 mmol) was added to a flame-dried, argon-flushed Schlenk tube and dissolved in DMF (1.2 mL). DMAP (15.0 mg, 0.12 mmol, 1.00 equiv.) and EDC hydrochloride (28.6 mg, 0.18 mmol, 1.50 equiv.) were added, followed by the corresponding alcohol ROH (10.0 equiv.). The mixture was warmed up and stirred at 40 °C under an argon atmosphere until completion and the reaction was monitored by TLC. Further addition of EDC hydrochloride (28.6 mg, 0.18 mmol, 1.50 equiv.), DMAP (15.0 mg, 0.12 mmol, 1.00 equiv.) and ROH (10.0 equiv.) after 16 hours was sometimes necessary to complete the reaction. At completion, the mixture was diluted in DCM and washed with 1M HCl. The aqueous phase was extracted with DCM and the combined organic layers were washed with brine, dried over Na_2SO_4, filtered and concentrated. The residue was purified *via* flash chromatography (gradient from 100:1 to 9:1 DCM/MeOH) to yield the corresponding product.

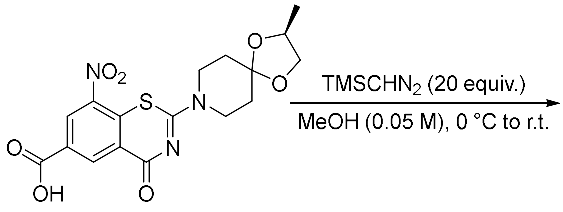

Methyl (*S*)-2-(2-methyl-1,4-dioxa-8-azaspiro[4.5]decan-8-yl)-8-nitro-4-oxo-4*H*-benzo[*e*][1,3]thiazine-6-carboxylate 6a. Acid **8** (50 mg, 0.12 mmol) was dissolved in MeOH (2.6 mL) in a flame-dried Schlenk tube flushed with argon. The yellow solution was cooled to 0 °C and trimethylsilyldiazomethane (2.0 M in hexanes, 1.2 mL, 2.46 mmol, 20 equiv.) was added dropwise. Gas evolution was observed and the solution turned slightly red. The mixture was stirred at room temperature for 15 minutes and monitored by TLC. At completion, the reaction mixture was concentrated and the residue purified by flash chromatography (gradient from 12/12/1 to 1/1/1 DCM/PE/acetone) to obtain **6a** as an orange oil (51.7 mg, 0.12 mmol, quant.).

^{1}H NMR (500 MHz, $CDCl_3$ + 1% TMS) δ 9.40 (d, *J* = 2.0 Hz, 1H), 9.12 (d, *J* = 2.0 Hz, 1H), 4.32 – 4.26 (m, 1H), 4.13 – 4.06 (m, 1H), 4.01 (br s, 4H), 4.01 (s, 3H), 3.50 (t, *J* = 7.9 Hz, 1H), 1.87 (br s, 2H), 1.84 (br s, 2H), 1.31 (d, *J* = 6.0 Hz, 3H); ^{13}C NMR (126 MHz, $CDCl_3$ + 1% TMS) δ 167.5, 164.1, 162.2, 144.1, 137.2, 134.8, 130.0, 129.5, 125.9, 106.5, 72.7, 71.0, 53.2, 44.7, 36.6, 35.4, 18.5; HRMS (ESI) m/z $[M+H]^+$ calcd. for $C_{18}H_{20}N_3O_7S$: 422.1017; found: 422.1015.

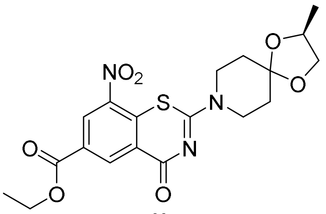

6b

Ethyl (*S*)-2-(2-methyl-1,4-dioxa-8-azaspiro[4.5]decan-8-yl)-8-nitro-4-oxo-4*H*-benzo[*e*][1,3]thiazine-6-carboxylate 6b. According to general procedure A, from ethanol as ROH (72.5 μL, 1.23 mmol), **6b** was obtained as yellow powder (9.0 mg, 0.02 mmol, 17%).

^{1}H NMR (500 MHz, $CDCl_3$) δ 9.38 (d, *J* = 2.0 Hz, 1H), 9.10 (d, *J* = 2.0 Hz, 1H), 4.46 (q, *J* = 7.1 Hz, 2H), 4.31 – 4.25 (m, 1H), 4.16 – 4.09 (m, 1H), 3.97 (br s, 4H), 3.49 (t, *J* = 7.9 Hz, 1H), 1.87 (br s, 2H), 1.83 (br s, 2H), 1.43 (t, *J* = 7.1 Hz, 3H), 1.31 (d, *J* = 6.1 Hz, 3H); ^{13}C NMR (126 MHz, $CDCl_3$) δ 167.2, 163.5, 161.9, 144.0, 136.9, 134.4, 129.8, 129.7, 125.9, 106.4, 72.5, 70.9, 62.3, 44.5, 36.4, 35.3, 18.3, 14.3; HRMS (ESI) m/z $[M+H]^+$ calculated for $C_{19}H_{22}N_3O_7S$: 436.1173; found: 436.1170.

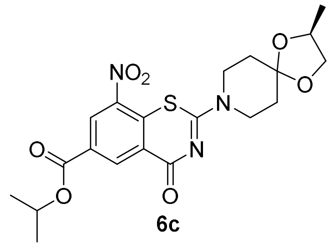

6c

Isopropyl (*S*)-2-(2-methyl-1,4-dioxa-8-azaspiro[4.5]decan-8-yl)-8-nitro-4-oxo-4*H*-benzo[*e*][1,3]thiazine-6-carboxylate 6c. According to general procedure A, from isopropanol as ROH (93.2 μL, 1.23 mmol), **6c** was obtained as a yellow powder (22.0 mg, 0.05 mmol, 41%).

^{1}H NMR (500 MHz, $CDCl_3$) δ 9.36 (d, *J* = 2.0 Hz, 1H), 9.10 (d, *J* = 2.0 Hz, 1H), 5.32 (hept, *J* = 6.3 Hz, 1H), 4.32 – 4.25 (m, 1H), 4.12 – 4.10 (m, 1H), 3.98 (br s, 4H), 3.50 (t, *J* = 7.9 Hz, 1H), 1.87 (br s, 2H), 1.83 (br s, 2H), 1.41 (d, *J* = 6.3 Hz, 6H), 1.30 (d, *J* = 6.0 Hz, 3H); ^{13}C NMR (126 MHz, $CDCl_3$) δ 167.2, 163.0, 161.9, 143.9, 136.8, 134.2, 130.1, 129.9, 125.8, 106.4, 72.5, 70.9, 70.3, 44.5, 36.4, 35.2, 21.9, 18.3; HRMS (ESI) m/z $[M+H]^+$ calcd. for $C_{20}H_{24}N_3O_7S$: 450.1329; found: 450.1328.

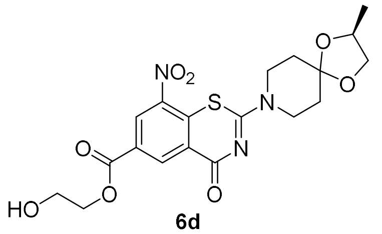

6d

2-Hydroxyethyl (*S*)-2-(2-methyl-1,4-dioxa-8-azaspiro[4.5]decan-8-yl)-8-nitro-4-oxo-4*H*-benzo[*e*][1,3]thiazine-6-carboxylate 6d. According to the general procedure A, from ethylene glycol as ROH (68.6 µL, 1.23 mmol), **6d** was obtained as a foamy yellow oil (34.5 mg, 0.08 mmol, 62%).

1H NMR (600 MHz, DMSO-d6) δ 9.11 (d, J = 2.1 Hz, 1H), 9.02 (d, J = 2.1 Hz, 1H), 4.79 (t, J = 5.7 Hz, 1H), 4.30 – 4.20 (m, 1H), 4.12 – 4.08 (m, 1H), 3.93 (s, 4H), 3.55 (q, J = 6.0 Hz, 2H), 3.45 (t, J = 7.8 Hz, 1H), 3.38 (q, J = 5.9 Hz, 2H), 1.80 (s, 4H), 1.22 (s, 3H); 13C NMR (151 MHz, DMSO-d6_2.5mm) δ 166.1, 162.9, 161.3, 143.8, 133.8, 132.8, 132.2, 128.0, 125.0, 106.1, 71.8, 70.0, 59.4, 42.5, 35.6, 34.4, 18.3; HRMS (ESI) m/z [M+H]+ calculated for C19H22N3O8S: 452.1122; found: 452.1121.

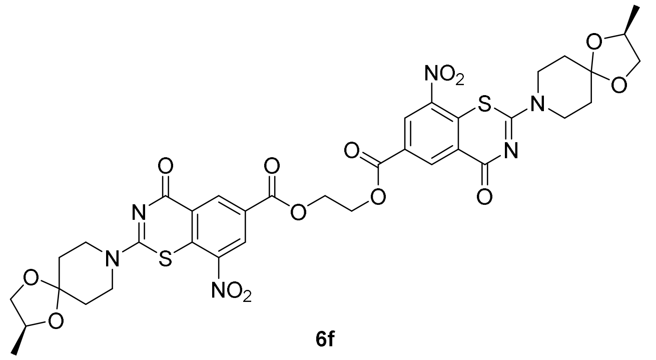

6f

Ethane-1,2-diyl bis(2-((*S*)-2-methyl-1,4-dioxa-8-azaspiro[4.5]decan-8-yl)-8-nitro-4-oxo-4*H*-benzo[*e*][1,3]thiazine-6-carboxylate) 6f. 6f was obtained as a side-product from the previous reaction as white powder (6.3 mg, 0.01 mmol, 6%).

^{1}H NMR (500 MHz, DMSO-d_6) δ 8.97 (d, *J* = 2.0 Hz, 2H), 8.85 (d, *J* = 2.0 Hz, 2H), 4.79 (s, 4H), 4.28 – 4.22 (m, 2H), 4.11 – 4.08 (m, 2H), 3.91 (s, 8H), 3.45 (t, *J* = 7.8 Hz, 2H), 1.80 (s, 8H), 1.22 (d, *J* = 5.9 Hz, 6H); ^{13}C NMR (126 MHz, DMSO-d_6) δ 165.4, 163.2, 161.0, 143.8, 135.1, 134.6, 129.1, 127.9, 125.2, 106.1, 71.8, 70.0, 63.7, 44.1, 35.6, 34.4, 18.3; HRMS (ESI) m/z [M+H]$^+$ calculated for $C_{36}H_{37}N_6O_{14}S_2$: 841.1804; found: 841.1791.

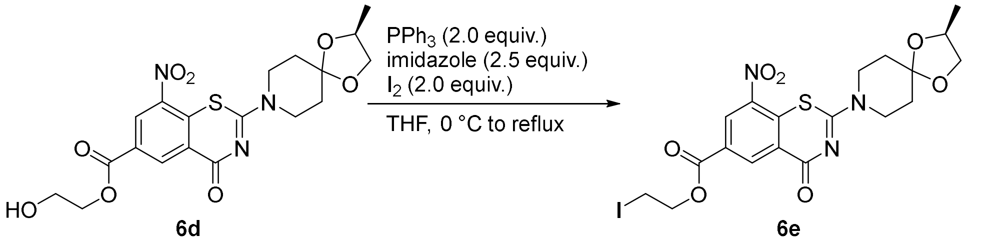

2-Iodoethyl (*S*)-2-(2-methyl-1,4-dioxa-8-azaspiro[4.5]decan-8-yl)-8-nitro-4-oxo-4*H*-benzo[*e*][1,3]thiazine-6-carboxylate 6e. Ester **6d** (100 mg, 0.22 mmol) was added to a flame-dried argon-flushed Schlenk tube and dissolved in dry THF (3.0 mL). The yellow mixture was cooled to 0 °C, and triphenylphosphine (116.3 mg, 0.44 mmol, 2.00 equiv.), imidazole (37.7 mg, 0.55 mmol, 2.50 equiv.) and iodine (56.3 mg, 0.44 mmol, 2.00 equiv.) were added. The reaction mixture was warmed up and stirred under reflux under an argon atmosphere. The reaction was monitored by TLC. At completion, the suspension was cooled down at room temperature and water was added. The mixture was extracted three times with DCM. The combined organic layers were washed with a saturated aqueous solution of $Na_2S_2O_3$ and water, and dried over Na_2SO_4, filtered and concentrated. The residue was purified by flash chromatography (gradient from 12/12/1 to 1/1/1 DCM/PE/acetone) to obtain **6e** as yellow solid (124.4 mg, 0.22 mmol, quant.).

^{1}H NMR (300 MHz, $CDCl_3$ + 1% TMS) δ 9.40 (d, *J* = 2.0 Hz, 1H), 9.12 (d, *J* = 2.0 Hz, 1H), 4.66 (t, *J* = 7.0 Hz, 2H), 4.35 – 4.24 (m, 1H), 4.14 – 4.09 (m, 1H), 4.04 (br s, 4H), 3.49 (q, *J* = 7.7 Hz, 1H), 3.46 (t, *J* = 7.0 Hz, 2H), 1.86 (br s, 4H), 1.31 (d, *J* = 6.1 Hz, 3H); ^{13}C NMR (75 MHz, $CDCl_3$ + 1% TMS) δ 167.0, 162.9, 161.7, 144.0, 137.0, 134.9, 129.9, 128.8, 126.1, 106.4, 72.5, 70.9, 66.0, 44.5, 36.4, 35.3, 18.3, -0.7; HRMS (ESI) m/z $[M+H]^+$ calculated for $C_{19}H_{21}IN_3O_7S$: 562.0139; found: 562.0134.

2. General Procedure B – Amide coupling

Acid **8** (20 mg, 0.05 mmol) was added to a flame-dried, argon-flushed Schlenk tube and dissolved in DMF (500 µL). Hydroxybenzotriazole (HOBt) (7.3 mg, 0.05 mmol, 1.10 equiv.), 2-(1H-Benzotriazole-1-yl)-1,1,3,3-tetramethyluronium hexafluorophosphate (HBTU) (20.5 mg, 0.05 mmol, 1.10 equiv.) and *N*,*N*-diisopropylethylamine (DIPEA) (9.2 µL, 0.05 mmol, 1.10 equiv.) were added, followed by the corresponding amine HNRR' (1.10 equiv.). The mixture was warmed up and stirred at 40 °C under argon atmosphere. Further addition of HOBt (7.3 mg, 0.05 mmol, 1.10 equiv.), HBTU (20.5 mg, 0.05 mmol, 1.10 equiv.), DIPEA (9.2 µL, 0.05 mmol, 1.10 equiv.) and HNRR' (1.1 equiv.) after 16 hours was sometimes necessary to bring the reaction to completion. At completion, the mixture was diluted in DCM and washed with water. The aqueous phase was extracted with DCM and the combined organic layers were washed with an aqueous saturated $NaHCO_3$ solution, dried over Na_2SO_4, filtered and concentrated. The residue was purified *via* flash chromatography (gradient from 50:1 to 1:1 DCM/MeOH) to yield the corresponding product.

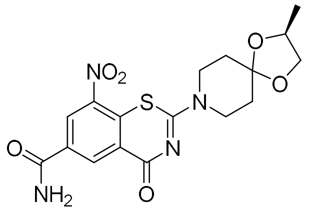

7a

(*S*)-2-(2-Methyl-1,4-dioxa-8-azaspiro[4.5]decan-8-yl)-8-nitro-4-oxo-4*H*-benzo[*e*][1,3]thiazine-6-carboxamide 7a. According to general procedure B, from ammonium chloride as HNRR' (2.9 mg, 0.05 mmol), **7a** was obtained as yellow solid (15.2 mg, 0.04 mmol, 76%). Instead of 1.10 equivalents, 2.20 equivalents of DIPEA (19.2 µL, 0.11 mmol) were used.

^{1}H NMR (600 MHz, DMSO-*d$_6$*) δ 9.11 (d, *J* = 2.0 Hz, 1H), 9.02 (d, *J* = 2.0 Hz, 1H), 8.58 (s, 1H), 7.83 (s, 1H), 4.28 – 4.23 (m, 1H), 4.11 – 4.09 (m, 1H), 3.93 (br s, 4H), 3.45 (t, *J* = 7.8 Hz, 1H), 1.80 (br s, 4H), 1.23 (d, *J* = 5.9 Hz, 3H); ^{13}C NMR (151 MHz, DMSO-*d$_6$*) δ 166.1, 164.5, 161.3, 143.8, 134.1, 132.7, 132.4, 128.1, 125.0, 106.1, 71.8, 70.0, 43.9, 35.6, 34.3, 18.3; HRMS (ESI) m/z $[M+H]^+$ calcd. for $C_{17}H_{19}N_4O_6S$: 407.1020; found: 407.1013.

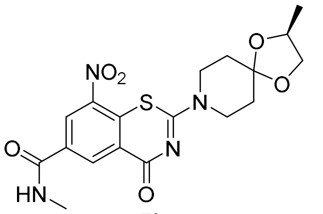

7b

(*S*)-*N*-Methyl-2-(2-methyl-1,4-dioxa-8-azaspiro[4.5]decan-8-yl)-8-nitro-4-oxo-4*H*-benzo[*e*][1,3]thiazine-6-carboxamide 7b. According to general procedure B, from methylamine hydrochloride as HNRR' (3.5 mg, 0.05 mmol), **7b** was obtained as yellow solid (14.4 mg, 0.04 mmol, 70%). Instead of 1.10 equivalents, 3.00 equivalents of DIPEA (25.0 µL, 0.15 mmol) were used.

^{1}H NMR (500 MHz, DMSO-d_6) δ 9.11 (q, J = 4.4 Hz, 1H), 9.09 (d, J = 2.1 Hz, 1H), 8.99 (d, J = 2.1 Hz, 1H), 4.28 – 4.22 (m, 1H), 4.11 – 4.08 (m, 1H), 3.91 (br s, 4H), 3.46 (t, J = 7.8 Hz, 1H), 2.83 (d, J = 4.5 Hz, 3H), 1.79 (s, 4H), 1.22 (d, J = 6.0 Hz, 3H); ^{13}C NMR (126 MHz, DMSO-d_6) δ 166.3, 163.4, 161.6, 144.0, 133.8, 132.8, 132.5, 128.0, 125.2, 106.3, 71.9, 70.2, 35.8, 34.6, 26.6, 18.5; HRMS (ESI) m/z $[M+H]^+$ calculated for $C_{18}H_{21}N_4O_6S$: 421.1176; found: 421.1176.

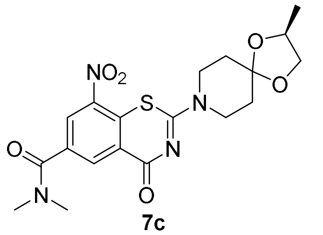

7c

(*S*)-*N*,*N*-Dimethyl-2-(2-methyl-1,4-dioxa-8-azaspiro[4.5]decan-8-yl)-8-nitro-4-oxo-4*H*-benzo[*e*][1,3]thiazine-6-carboxamide 7c. According to general procedure B, from dimethylamine as HNRR' (2M in THF, 27.0 µL, 0.05 mmol), **7c** was obtained as yellow oil (10.7 mg, 0.02 mmol, 50%).

^{1}H NMR (500 MHz, $CDCl_3$ + 1% TMS) δ 8.86 (d, J = 1.9 Hz, 1H), 8.68 (d, J = 1.7 Hz, 1H), 4.33 – 4.26 (m, 1H), 4.20 (br s, 2H), 4.13 – 4.10 (m, 1H), 3.97 (br s, 2H), 3.50 (t, J = 7.9 Hz, 1H), 3.16 (s, 3H), 3.06 (s, 3H), 1.88 (br s, 2H), 1.84 (br s, 2H), 1.31 (d, J = 6.0 Hz, 3H); ^{13}C NMR (126 MHz, $CDCl_3$) δ 167.8, 167.5*, 162.8, 144.2, 135.2, 134.8, 131.7, 129.1, 125.5*, 106.5, 72.7, 71.0, 44.8, 39.8, 36.5, 35.9, 35.4, 18.5; HRMS (ESI) m/z $[M+H]^+$ calcd. for $C_{19}H_{23}N_4O_6S$: 435.1333; found: 435.1336.

*signals observed on not entirely purified **7c**.

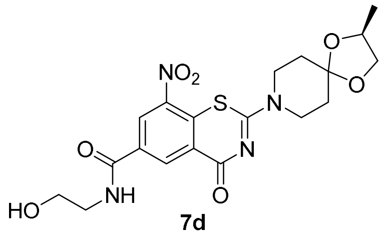

7d

(*S*)-*N*-(2-Hydroxyethyl)-2-(2-methyl-1,4-dioxa-8-azaspiro[4.5]decan-8-yl)-8-nitro-4-oxo-4*H*-benzo[*e*][1,3]thiazine-6-carboxamide 7d. According to general procedure B, from ethanolamine as HNRR' (3.3 µL, 0.05 mmol), **7d** was obtained as yellow solid (15.2 mg, 0.03 mmol, 69%).

^{1}H NMR (600 MHz, DMSO-d_6) δ 9.12 (t, J = 5.6 Hz, 1H), 9.11 (d, J = 2.0 Hz, 1H), 9.02 (d, J = 2.1 Hz, 1H), 4.79 (t, J = 5.7 Hz, 1H), 4.28 – 4.23 (m, 1H), 4.12 – 4.08 (m, 1H), 3.93 (br s, 4H), 3.55 (q, J = 6.0 Hz, 2H), 3.45 (t, J = 7.8 Hz, 1H), 3.38 (q, J = 5.9 Hz, 2H), 1.80 (br s, 4H), 1.23 (d, J = 6.0 Hz, 3H); ^{13}C NMR (151 MHz, DMSO-d_6) δ 166.1, 162.9, 161.3, 143.8, 133.8, 132.8, 132.2, 128.0, 125.0, 106.1, 71.8, 70.0, 59.4, 43.9, 42.5, 35.6, 34.4, 18.3; HRMS (ESI) m/z [M+H]$^+$ calcd. for $C_{19}H_{23}N_4O_7S$: 451.1282; found: 451.1280.

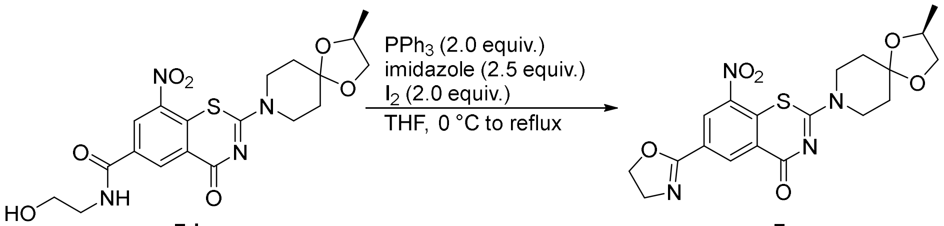

(*S*)-6-(4,5-Dihydrooxazol-2-yl)-2-(2-methyl-1,4-dioxa-8-azaspiro[4.5]decan-8-yl)-8-nitro-4*H*-benzo[*e*][1,3]thiazin-4-one 7e. Amide **7d** (50.0 mg, 0.11 mmol) was added to a flame-dried argon-flushed Schlenk tube and dissolved in dry THF (1.5 mL). The yellow mixture was cooled to 0 °C, and triphenylphosphine (58.3 mg, 0.22 mmol, 2.00 equiv.), imidazole (18.9 mg, 0.28 mmol, 2.50 equiv.) and iodine (28.2 mg, 0.44 mmol, 2.00 equiv.) were added. The reaction mixture was warmed and stirred under reflux and an argon atmosphere. At completion, the suspension was cooled to room temperature and water was added. The mixture was extracted three times with DCM. The combined organic layers were washed with a saturated aqueous solution of $Na_2S_2O_3$ and water, dried over Na_2SO_4 and concentrated. The residue was purified by flash chromatography (gradient from 50:1 to 1:1 DCM/MeOH) to obtain **7e** as yellow solid (48.0 mg, 0.14 mmol, quant.).

^{1}H NMR (300 MHz, $CDCl_3$) δ 9.31 (d, J = 2.0 Hz, 1H), 9.09 (d, J = 2.0 Hz, 1H), 4.52 (t, J = 9.7 Hz, 2H), 4.35 – 4.24 (m, 1H), 4.14 (t, J = 9.5 Hz, 2H), 4.14 – 4.02 (m, 1H), 4.02 (br s, 4H), 3.50 (t, J = 7.9 Hz, 1H), 1.87 – 1.83 (m, 4H), 1.31 (d, J = 6.1 Hz, 3H); ^{13}C NMR (75 MHz, $CDCl_3$) δ 167.4, 162.2, 162.0, 144.0, 135.8, 132.7, 128.8, 127.3, 126.0, 106.6, 72.7, 71.0, 68.6, 55.4, 44.6, 36.6, 35.4, 18.5; HRMS (ESI) m/z [M+H]$^+$ calcd. for $C_{19}H_{21}N_4O_6S$: 433.1176; found: 433.1175.

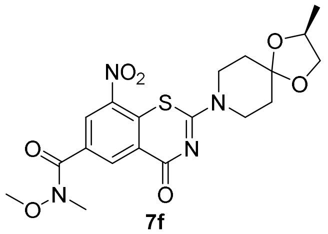

7f

(*S*)-*N*,*N*-Dimethyl-2-(2-methyl-1,4-dioxa-8-azaspiro[4.5]decan-8-yl)-8-nitro-4-oxo-4*H*-benzo[*e*][1,3]thiazine-6-carboxamide 7f. According to the general procedure B, from N,O-dimethylhydroxylamine hydrochloride as HNRR' (5.3 mg, 0.05 mmol), **7f** was obtained as a yellow oil (15.0 mg, 0.03 mmol, 68%). Instead of 1.10 equivalents, 2.20 equivalents of DIPEA (18.4 µL, 0.11 mmol) were used.

^{1}H NMR (500 MHz, $CDCl_3$ + 1% TMS) δ 9.14 (d, J = 2.0 Hz, 1H), 8.89 (d, J = 2.0 Hz, 1H), 4.33 – 4.26 (m, 1H), 4.13 – 4.11 (m, 1H), 3.97 (br s, 4H), 3.61 (s, 3H), 3.51 (t, J = 7.9 Hz, 1H), 3.41 (s, 3H), 1.89 (br s, 2H), 1.85 (br s, 2H), 1.31 (d, J = 6.1 Hz, 3H); ^{13}C NMR (126 MHz, $CDCl_3$ + 1% TMS) δ 167.3*, 165.9, 162.3, 143.8, 136.3, 133.0, 132.6, 129.6, 125.4*, 106.4, 72.5, 70.9, 61.5, 44.6, 36.4, 35.2, 33.3, 18.3.; HRMS (ESI) m/z $[M+H]^+$ calculated for $C_{19}H_{23}N_4O_7S$: 451.1282; found: 451.1285.

*signals observed on not entirely purified **7f**.

Supplementary Method 5: Biological Assays

1. MIC determination

Minimal inhibitory concentrations were determined against *Mycobacterium vaccae* (JMRC:STI:10670) and *Mycobacterium smegmatis* (JMRC:STH:00056). *M. vaccae* and *M. smegmatis* cultures were grown 48 h at 32 °C in a medium consisting of glycerol (1%), beef extract (0.5%), peptone from casein (0.5%) and sodium chloride (0.3%). Serial dilutions of the DMSO test item solutions (1 mg·mL^{-1}) were prepared at 100 µL scale in 96 well plates in Mueller Hinton broth starting at 200 µg·mL^{-1} (highest) to cover a final concentration range of 100 to 0.05 µg·mL^{-1}. Each plate was equipped with a series of DMSO and ciprofloxacin dilutions as quality controls. 96 well plates were inoculated with 100 µL diluted culture (Mueller Hinton bullion) at a final concentration of $5 \cdot 10^5$ and incubated at 37 °C for 42 h. Growth/no growth was visually inspected.

2. Proliferation and cytotoxicity assay

Cells and culture conditions

The following cell lines and media were used:

Cell line	Cell culture medium
HUVEC (ATCC CRL-1730)	DMEM (CAMBREX 12-614F)
K-562 (DSM ACC 10)	RPMI 1640 (CAMBREX 12-167F)
HeLa (DSM ACC 57)	RPMI 1640 (CAMBREX 12-167F).

Cells were grown in the appropriate cell culture medium supplemented with 10 mL/L ultraglutamine 1 (CAMBREX 17-605E/U1), 550 µL / L (50 mg/mL) gentamicin sulfate (CAMBREX 17-518Z), and 10% heat inactivated fetal bovine serum (GIBCO Life Technologies 10270-106) at 37 °C in 5% CO_2 in high density polyethylene flasks (NUNC 156340).

To decide if the compounds have an antiproliferative and/or a cytotoxic effect on human cells, cell death and retardation of cell proliferation were assessed. Under our experimental conditions, the optical density measured from the CellTiter-Blue® reagent and methylene blue assay is proportional to the number of viable cells.

Proliferation assay

The test substances were dissolved in DMSO and further diluted in the cell culture medium. The adherent cells were harvested at the logarithmic growth phase after soft trypsinization using 0.25% trypsin in PBS containing 0.02% EDTA (Biochrom KG L2163). For each experiment, approximately 10,000 cells were seeded with 0.1 mL culture medium per well of the 96-well microplates (HUVEC: flat bottomed NUNC 167008, K-562: round bottomed NUNC 163320). To test the antiproliferative

effect of test compounds on HUVEC and K-562, the cells were incubated for 72 hours in plates prepared with control and different dilutions of test substances. The GI_{50} values were defined as 50% inhibition of proliferation compared to the untreated control.

Cytotoxicity assay

For the cytotoxicity assay, HeLa cells were preincubated for 48 hours without the test substances. To test the cytotoxic effect on HeLa, dilutions of the compounds were carried out carefully on the subconfluent monolayers of HeLa cells after the preincubation time. After incubation time, the cytolytic effect of compounds was analysed in comparison to the negative control. The 50% cytotoxic concentration (CC_{50}) was defined as the test compound concentration required for destruction of the cell monolayer by 50% compared to untreated control.

Incubation conditions

Cells were incubated with dilutions of the test substances in microplates for 72 hours at 37 °C in a humidified atmosphere and 5% CO_2. This incubation was found to be optimal for the evaluation of the cytotoxicity and the inhibition of cell proliferation. The number of viable cells was determined subsequent to staining with CellTiter-Blue® reagent or methylene blue.

Methods of evaluation

K-562 cells: The influence of test substances on cell proliferation of K-562 was determined *via* the CellTiter-Blue® assay (PROMEGA). Briefly, viable cells are able to reduce the indicator dye resazurin into resorufin, whereas nonviable cells rapidly lose metabolic capacity and do not reduce the indicator dye. Thus, formation of resorufin was followed by measuring absorption at 570 nm and subtracting absorption measured at 600 nm. Values are compared to blank well containing CellTiter-Blue® reagent without cells.

HUVEC and HeLa cells: The adherent HUVEC and HeLa cells were fixed by glutaraldehyde (MERCK 1.04239.0250) and stained with a 0.05 % solution of methylene blue (SERVA 29198) for 15 min. After gently washing, the stain was eluted by adding 0.2 mL of hydrochloric acid (0.33 M) to each well. The optical densities were measured at 660 nm (methylene blue) in SUNRISE microplate reader (TECAN).

General remarks: Signals from the methylene blue and CellTiter-Blue® reagent are proportional to the number of viable cells. A repeat determination has been conducted in all experiments, four replicates were assayed. The calculations of the different values of GI_{50} and CC_{50} were performed with the software Magellan (TECAN).

3. Hydride Meisenheimer complex formation propensity assay

The assay was performed as described previously.[6]

RAW 264.7 cell cultivation

The murine macrophage cell line RAW 264.7 (91062702, ECACC) was cultured in DMEM medium (DMEM (Dulbecco's Modified Eagle's Medium; Lonza) supplemented with 10% (v/v) FCS (fetal bovine serum; Sigma Aldrich) and 2 mM UltraGlutamine (alanyl-L-glutamine, Biozym)) at 37 °C, in a humidified atmosphere and 5% CO_2. For routine maintenance, cells (viability ≥ 95%) were seeded with a confluency of 10% and harvested by scraping at a confluency of 90%. Cultures were not maintained beyond three months or 25 passages and were tested for mycoplasma contamination with the MycoSPY® Master Mix (Biontex) according to the producer's manual.

Assay

200 µL RAW 264.7 cell suspension (3×10^7 cells/mL in DMEM medium (DMEM (Lonza) supplemented with 10 % (v/v) FCS (Sigma Aldrich) and 2 mM UltraGlutamine (Biozym) was incubated with 0.33 µL BTZ derivative or **1** solution (20 mg/mL in DMSO) at 37 °C, orbital shaking at 1000 rpm, 3 h in 1.5 mL Eppendorf tubes in a Thermomixer C (Eppendorf, Germany) (n = 3). After incubation, the samples were centrifuged at 4 °C, 300×*g*, for 5 min; the supernatant was collected and immediately processed at 4 °C or stored at −80 °C. For sample preparation, a 25 µL sample was precipitated with 75 µL methanol (degassed, ice cold) and centrifuged for 5 min, 4 °C, 16.000×*g*. The supernatant was kept at 4 °C and subjected to LC-HRMS without further treatment.

Analysis

Samples were analyzed with a Thermo Vanquish Horizon UHPLC system (Thermo Fisher Scientific, Bremen, Germany) coupled to a Thermo Scientific QExactive Orbitrap mass spectrometer (Thermo Fisher Scientific, Bremen, Germany) by using a UPLC column (ACQUITY HSS T3, Waters, 1.8 µm particle size, 2.1 × 50 mm). The column temperature was maintained at 25 °C, and the samples were kept at 4 °C. The UPLC system was operated without UV at a 0.6 mL/min flow rate with an injection volume of 5 µL. Mobile phases consisted of 0.1% (v/v) formic acid in water (Eluent A) and 0.1% (v/v) formic acid in acetonitrile (Eluent B). Chromatographic separation was obtained: a step with an increase from 5% to 98% of Eluent B in 4 min, followed by an isocratic hold for 2.5 min, and a step decrease to 5% Eluent B in 0.5 min followed by an isocratic hold for 2 min. The high-resolution mass spectra were acquired with electrospray ionization in the positive mode. Full scan data were obtained at a resolving power of 60,000 full widths at half maximum. Ion source parameters were: spray voltage 3.2 kV, capillary temperature 320 °C, RF level 40, sheath gas pressure (N_2 > 95%) 50, auxiliary gas (N_2 > 95%) 10, auxiliary gas heater temperature 200 °C. The value for the automatic gain control (AGC) target was

set at 10^6, a scan range of m/z 120 to 1800 was chosen, and the injection time was set to 200 ms. The scan rate was set at 2 scans/s.

Mass data of BTZs and their correlating HMC were analyzed and processed with the software XCalibur 4.1 (Thermo Fisher, USA). The mean and the standard deviation of three biological and three technical replicates were calculated and relative HMC formation calculated with the equation:

$$relative\ HMC\ propensity = \frac{\left(\frac{\int EIC_{DHMC}}{\int EIC_D + \int EIC_{DHMC}}\right)}{\left(\frac{\int EIC_{RHMC}}{\int EIC_R + \int EIC_{RHMC}}\right)}$$

HMC formation of BTZ-043 served as reference and quality control in each biological replicate series.

HMC accurate mass for each derivative was extracted from the corresponding TIC. MS spectra of the HMC metabolites showed the characteristic $[HMC–2H+H]^+$ peak, as reported by Desfontaine et al.[7]

Table S8. List of calculated and found accurate mass for $[HMC+H]^+$ and the corresponding ion fragment $[HMC–2H+H]^+$.[a]

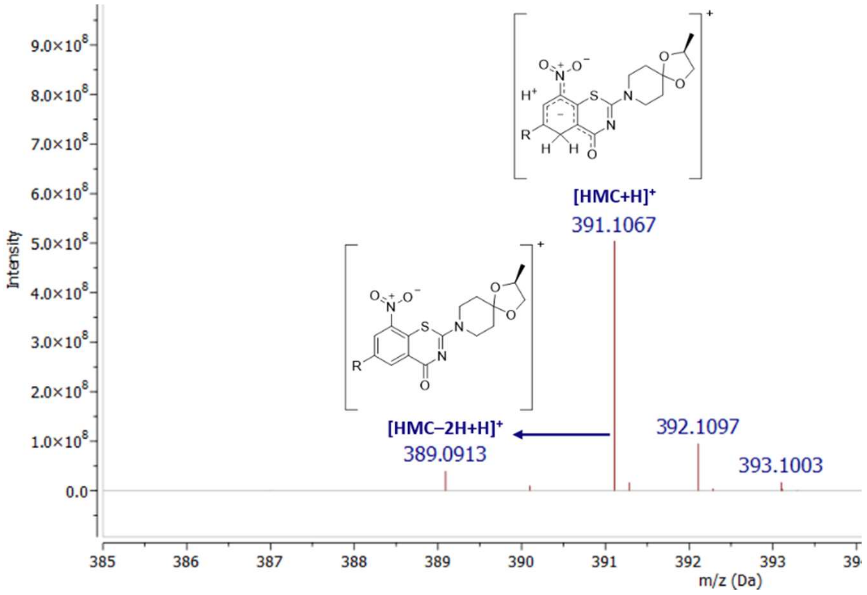

	m/z [HMC+H]+		*m/z* [HMC–2H+H]+	
Compound	calcd.	found	calcd.	found
**9**	391.1071	391.1067	389.0914	389.0913
**10**	492.0085	492.0088	489.9928	489.9937
**11**	444.0223	444.0223	442.0067	442.0067
**6a**	424.1173	424.1176	422.1016	422.1016
**6b**	438.1329	438.1334	436.1173	436.1175
**6c**	452.1486	452.1492	450.1329	450.1331
**6d**	454.1279	454.1284	452.1122	452.1125
**6e**	564.0296	564.0301	562.0139	562.0148
**7a**	409.1176	409.1181	407.1020	407.1028

	m/z [HMC+H]$^+$		*m/z* [HMC–2H+H]$^+$	
Compound	calcd.	found	calcd.	found
**7b**	423.1333	423.1342	421.1176	421.1182
**7c**	437.1489	437.1500	435.1333	435.1339
**7d**	453.1438	453.1441	451.1282	451.1282
**7e**	435.1333	435.1336	433.1176	433.1178
**7f**	453.1438	453.1440	451.1282	451.1289

[a] HMC spectra analysis. Here, analysis of the mass spectrum of the HMC of compound **9** (R = CN) is used as example.

4. Microsomal stability assay

Assay

A microsomal stability assay was performed according to an internal standard procedure. Positive control compound diclofenac and three test items were freshly prepared as sub-dilutions in phosphate buffer (100 mM, pH 7.4) at concentrations of 200 µM and 20 µM, respectively. All incubations were conducted in triplicate in glass screw neck vials (1.5 mL, flat bottom, Macherey-Nagel, Düren, Germany) on a Thermomixer C (Eppendorf, Germany) at 37 °C and 1500 rpm. The incubation mixtures contained 270 µL human liver microsomes (HLM, 1.1 $mg{\cdot}mL^{-1}$, UltraPool HLM 150, Corning B.V. Life Science, Amsterdam, the Netherlands), 270 µL NADP regeneration mix (1 mM NADP, 5 mM glucose-6-phosphate, 5 $units{\cdot}mL^{-1}$ glucose-6-phosphate dehydrogenase and 5 mM $MgCl_2$) and 30 µL phosphate buffer to reach a total volume of 570 µL. Negative controls were performed with heat inactivated (80 °C, 30 min) HLM solution. Reactions were initiated by addition of diclofenac or the test item solution (30 µL). Aliquots of 100 µL were taken at 1, 15, 30, 60, and 90 minutes and immediately processed with 100 µL acetonitrile at 4°C to terminate the reactions, and shaken for 2 minutes at 4°C with 1600 rpm. The mixed samples were further centrifuged for 2 minutes at 4 °C with 16.1 krcf. The supernatants were withdrawn and subjected to high resolution mass spectrometry analysis without further treatment.

Analysis of microsomal stability assay

Samples were analyzed using a Vanquish Horizon UHPLC system (Thermo Fisher Scientific, Bremen, Germany) coupled to a Thermo Scientific QExactive HF-X Orbitrap mass spectrometer (Thermo Fisher Scientific, Bremen, Germany) using a UPLC column (ACQUITY HSS T3, Waters, 1.8 µm particle size, 2.1 × 50 mm dimensions) equipped with a pre-column (ACQUITY HSS T3 VanGuard Pre-column, Waters, 1.8 µm particle size, 2.1 × 5 mm dimensions). The column temperature was maintained at 25°C, and the samples were kept at 4°C. The UHPLC system was operated at a flow rate of 0.6 mL/min and an injection volume of 5 µL. Mobile phases consisted of 0.1% (v/v) formic acid in water (Eluent A) and 0.1% (v/v) formic acid in acetonitrile (Eluent B). Chromatographic separation was obtained as follows: 0 to 4 min: a linear from 5 to 98% of Eluent B; 4 to 6.5 min: isocratic at 98% B; 6.5 to 7 min: linear from 98 to 5% B; 7 to 9 min: isocratic at 5% B. The high-resolution mass spectra were acquired with electrospray ionization at both positive and negative modes. A scan range of *m*/*z* 120 to 1800 was chosen, and the maximum injection time was set to 200 ms. Ion source parameters were: spray voltage 3.5 kV, capillary temperature 320°C, RF level 40, sheath gas pressure 50 (N_2 > 95%), auxiliary gas 10 (N_2 > 95%), auxiliary gas heater temperature 300°C. The value for the automatic gain control (AGC) target was set to 10^6, resolution was 60000, and chromatographic peak width (FWHM) was 15 s.

Calculation of the results from microsomal stability assay

Elimination rate constant (k) = (- gradient)

Half-life ($t_{1/2}$) (min) = $\frac{ln}{k}$

V (µL·mg^{-1}) = $\frac{volume\ of\ incubation\ (\mu L)}{protein\ in\ the\ incubation\ (mg)}$

Intrinsic clearance in liver microsomes ($Cl_{int,\ micro}$) (µL·min·mL^{-1}protein) = $\frac{V \times ln2}{t_{1/2}}$

Intrinsic clearance (Cl_{int}) (mL·min^{-1}·kg^{-1}) = $\frac{ln}{t_{1/2}} \times \frac{V}{1000} \times \frac{mg\ microsomal\ protein}{g\ liver\ weight} \times \frac{g\ liver}{kg\ body\ weight}$

(52.5 mg protein· g^{-1} liver used for human; 22.0 g· kg^{-1} liver to body weight used for human)

Hepatic extraction ratio (E_h) = $\frac{f_u \times Cl_{int}}{Q+f_u \times Cl_{int}}$

(f_u: fraction of drug unbound in plasma, f_u = 1 used for the calculation; Q: hepatic blood flow, 20.0 mL·min^{-1}·kg^{-1} for human).

Supplementary Method 6: Computational modeling

1. Conceptual density functional theory (CDFT) descriptors

Details

Application of conceptual DFT for the prediction of chemical reactivity for the studied compounds was accomplished using the Schrödinger suite (version 2022-2). Initially, the compound structures were submitted to conformational search using MacroModel (version 13.1) and the OPLS4 force field.[9] The lowest energy conformer was then submitted to structure optimization using the AM1 method. The resulting structures were optimized in the gas phase at DFT level in Jaguar (version 11.1),[10] using the B3LYP[11,12] functional with dispersion correction (D3),[13] in combination with the 6-31+G(d,p) basis set.[14–16] Real minima were confirmed by absence of imaginary values upon frequency analysis calculation at the same level of theory. Single point energy calculations were performed using the same functional in combination with the 6-311+G(d,p) basis set,[16,17] considering solvent effects by the CPCM[18] solvation model with parameters appropriate for water. The LANL2DZ basis set was used to model Br and I atoms.[19] Mulliken charges and atomic Fukui indices were directly calculated by Jaguar.[20,21] A combination of Schrödinger's qm_descriptors.py and *in-house* Python scripts was employed for data retrieval, processing, and analysis. The Python code is available at https://github.com/BernalFA/HMC-carboxy-BTZs.

Global reactivity descriptors

HOMO and LUMO energies were used to calculate the electronic chemical potential (μ), the hardness (η), and the electrophilicity index (ω) as global reactivity descriptors. Equations 1-3 show the respective definitions.[22–24]

$$\mu = -\frac{I + A}{2} \approx \frac{1}{2}(E_{HOMO} + E_{LUMO}) \quad (1)$$

$$\eta = I - A \approx -(E_{HOMO} - E_{LUMO}) \quad (2)$$

$$\omega = \frac{\mu^2}{2\eta} \quad (3)$$

where I and A represent ionization energy and electron affinity, respectively. E represent energy.

2. Reactivity – Hydride addition

Automated reaction profile calculations were accomplished in autodE[25] (version 1.2.3) with ORCA[26,27] (version 5.0.3) as QM engine. Structures for BTZs and their respective HMCs, as well as BH_4^- and BH_3 were optimized in the gas phase using the GGA functional PBE0[28,29] with dispersion correction (D3BJ)[13,30] and RIJCOSX[31] approximation, in combination with the ma-def2-SVP[32] basis set. Single point energy calculations were performed using the functional PBE0-DB3J in combination with the ma-def2-TZVP basis set.[32] Solvent effects were included using the CPCM[18] solvation model with parameters appropriate for water. Thermochemical analysis at 298.15 K and 1 atm was performed to obtain gas phase and solvation free energies. Real minima or saddle point were verified by manual inspection of the frequencies outputted by autodE. An *in-house* Python script was employed to compile and process data from all the individual runs. The Python code is available at https://github.com/BernalFA/HMC-carboxy-BTZs.

3. Covalent docking

Docking simulations were performed using the Schrödinger Suite (version 2022-2). Protein structure (PDB: 4F4Q) was prepared using the Protein Preparation Workflow with default parameters.[33] A missing loop (residues 323-338) was added using Prime.[34,35] H-bonding was refined by the sampling water orientation algorithm at pH of 7.4 using PROPKA.[36] Water molecules beyond 3 Å from heteroatoms were deleted. The structure was optimized by restrained energy minimization using the OPLS4 force field,[9] with convergence criterion of 0.3 Å for heavy atoms RMSD. Ligand structures were built in Maestro from SMILES strings and prepared using LigPrep with the OPLS4 force field.
Covalent docking was performed using the CovDock workflow from Schrödinger.[37] The geometric center of BTZ-043 was considered the grid centroid in a box size of 20 x 20 x 20 Å. Cys394 was selected as reactive residue and its nucleophilic addition to nitroso group was used for covalent docking. Pose prediction mode was always employed. Binding free energy estimations for the non-covalent complexes formed between the nitroso species and the protein prior to covalent binding were obtained by the molecular mechanics/generalized Born surface area (MM/GBSA) method[38,39] using Prime.

4. Non-covalent docking

Conventional docking of nitro compounds into the reduced DprE1 binding site was performed using Glide.[40,41] As for covalent docking, compound **1** was used to define the grid centroid of a 20 x 20 x 20 Å box. Ten poses per compound were obtained in standard precision mode. Epik[42] state penalization was included into the docking scoring.

5. Molecular dynamics simulations

MD simulations were performed using Desmond (Schrödinger release 2022-2) with the OPLS4 force field. The covalently docked structure for each representative compound (**1**, **6c**, **7c**, and **8**) was solvated using the simple point charge (SPC) water model in an orthorhombic simulation box with a 10 Å water buffer around the complex structure. The appropriate number of Na^+/Cl^- counterions were added to neutralize the system and to reach a physiological salt concentration of 0.15 M. A default equilibration protocol was used prior production runs, including solute relaxation at T = 10 K using the NVT ensemble, solute relaxation at T = 10 K for 12 ps using the NPT ensemble, solute equilibration at T = 300 K for 12 ps, and NPT system equilibration without restraints at T = 300 K for 24 ps. Afterward, a 100 ns production run in the NPT ensemble at 300 K and 1 atm was performed. The Nose–Hoover thermostat[43] and the Martyna–Tobias–Klein barostat[44] were used with default settings. A RESPA integrator was used with 2 fs timestep. Electrostatic forces were treated using the particle-mesh Ewald method[45] with a default cut-off radius of 9 Å. Protein–ligand interactions were obtained using the Simulation Interactions Diagram (SID) panel from Maestro. For analysis, the first 30 ns of simulation were considered equilibration and discarded. Interactions persisting less than 30% of the simulation time for at least one compound were considered irrelevant. The Python code for analysis is available at https://github.com/BernalFA/HMC-carboxy-BTZs.

6. Redox potentials

A Born-Haber cycle was implemented for calculation of redox potentials as described previously.[46] In brief, *in-house* python scripts were used to run DFT calculations using the wrappers and functionality of autodE (version 1.2.3).[25] The same conditions as those used for reaction profiling upon hydride addition were used. The Nernst equation was employed to transform free energy values into one-electron redox potentials. Redox potentials relative to the standard hydrogen electrode (SHE) were obtained using a reported reference value (4.28 V).[47,48] The Python code is available at https://github.com/BernalFA/HMC-carboxy-BTZs.

7. Membrane permeability

Membrane permeability predictions were carried out using the Schrödinger suite (version 2022-2). The physics-based model implemented reproduces passive permeation in the RRCK cell monolayer assay.[49,50]

Supplementary References

1. Sheldrick, G. M. SHELXT – Integrated space-group and crystal-structure determination. *Acta Crystallogr. Sect. A Found. Adv.* **71**, 3–8 (2015).
2. Sheldrick, G. M. Crystal structure refinement with SHELXL. *Acta Crystallogr. Sect. C Struct. Chem.* **71**, 3–8 (2015).
3. Dolomanov, O. V., Bourhis, L. J., Gildea, R. J., Howard, J. A. K. & Puschmann, H. OLEX2 : a complete structure solution, refinement and analysis program. *J. Appl. Crystallogr.* **42**, 339–341 (2009).
4. Bruker AXS Inc., Madison, Wisconsin, U. Bruker AXS. *Apex4 and SADABS* (2001).
5. Ueda, T., Konishi, H. & Manabe, K. Palladium-Catalyzed Reductive Carbonylation of Aryl Halides with N -Formylsaccharin as a CO Source. *Angew. Chemie Int. Ed.* **52**, 8611–8615 (2013).
6. Joch, M. *et al.* Whole cell hydride Meisenheimer complex biotransformation guided optimization of antimycobacterial benzothiazinones. *Eur. J. Med. Chem.* **264**, 116023 (2024).
7. Desfontaine, V. *et al.* Optimized LC-MS/MS quantification of tuberculosis drug candidate macozinone (PBTZ169), its dearomatized Meisenheimer Complex and other metabolites, in human plasma and urine. *J. Chromatogr. B* **1215**, 123555 (2023).
8. Daina, A., Michielin, O. & Zoete, V. SwissADME: A free web tool to evaluate pharmacokinetics, drug-likeness and medicinal chemistry friendliness of small molecules. *Sci. Rep.* **7**, 1–13 (2017).
9. Lu, C. *et al.* OPLS4: Improving Force Field Accuracy on Challenging Regimes of Chemical Space. *J. Chem. Theory Comput.* **17**, 4291–4300 (2021).
10. Bochevarov, A. D. *et al.* Jaguar: A high-performance quantum chemistry software program with strengths in life and materials sciences. *Int. J. Quantum Chem.* **113**, 2110–2142 (2013).
11. Becke, A. D. Density-functional thermochemistry. III. The role of exact exchange. *J. Chem. Phys.* **98**, 5648–5652 (1993).
12. Stephens, P. J., Devlin, F. J., Chabalowski, C. F. & Frisch, M. J. Ab Initio Calculation of Vibrational Absorption and Circular Dichroism Spectra Using Density Functional Force Fields. *J. Phys. Chem.* **98**, 11623–11627 (1994).
13. Grimme, S., Antony, J., Ehrlich, S. & Krieg, H. A consistent and accurate ab initio parametrization of density functional dispersion correction (DFT-D) for the 94 elements H-Pu. *J. Chem. Phys.* **132**, 154104 (2010).
14. Hehre, W. J., Ditchfield, R. & Pople, J. A. Self—Consistent Molecular Orbital Methods. XII. Further Extensions of Gaussian—Type Basis Sets for Use in Molecular Orbital Studies of Organic Molecules. *J. Chem. Phys.* **56**, 2257–2261 (1972).
15. Hariharan, P. C. & Pople, J. A. The influence of polarization functions on molecular orbital hydrogenation energies. *Theor. Chim. Acta* **28**, 213–222 (1973).
16. Clark, T., Chandrasekhar, J., Spitznagel, G. W. & Schleyer, P. V. R. Efficient diffuse function-augmented basis sets for anion calculations. III. The 3-21+G basis set for first-row elements, Li-F. *J. Comput. Chem.* **4**, 294–301 (1983).
17. Krishnan, R., Binkley, J. S., Seeger, R. & Pople, J. A. Self-consistent molecular orbital methods. XX. A basis set for correlated wave functions. *J. Chem. Phys.* **72**, 650–654 (1980).

18. Barone, V. & Cossi, M. Quantum Calculation of Molecular Energies and Energy Gradients in Solution by a Conductor Solvent Model. *J. Phys. Chem. A* **102**, 1995–2001 (1998).

19. Wadt, W. R. & Hay, P. J. Ab initio effective core potentials for molecular calculations. Potentials for main group elements Na to Bi. *J. Chem. Phys.* **82**, 284–298 (1985).

20. Yang, W. & Mortier, W. J. The use of global and local molecular parameters for the analysis of the gas-phase basicity of amines. *J. Am. Chem. Soc.* **108**, 5708–5711 (1986).

21. Fuentealba, P. & Contreras, R. Fukui function in chemistry. in *Reviews of Modern Quantum Chemistry* 1013–1052 (WORLD SCIENTIFIC, 2002). doi:10.1142/9789812775702_0034.

22. Parr, R. G. & Yang, W. Density-Functional Theory of the Electronic Structure of Molecules. *Annu. Rev. Phys. Chem.* **46**, 701–728 (1995).

23. Chakraborty, D. & Chattaraj, P. K. Conceptual density functional theory based electronic structure principles. *Chem. Sci.* **12**, 6264–6279 (2021).

24. Domingo, L., Ríos-Gutiérrez, M. & Pérez, P. Applications of the Conceptual Density Functional Theory Indices to Organic Chemistry Reactivity. *Molecules* **21**, 748 (2016).

25. Young, T. A., Silcock, J. J., Sterling, A. J. & Duarte, F. autodE: Automated Calculation of Reaction Energy Profiles— Application to Organic and Organometallic Reactions. *Angew. Chemie Int. Ed.* **60**, 4266–4274 (2021).

26. Neese, F. The ORCA program system. *WIREs Comput. Mol. Sci.* **2**, 73–78 (2012).

27. Neese, F. Software update: The ORCA program system—Version 5.0. *WIREs Comput. Mol. Sci.* **12**, 1–15 (2022).

28. Adamo, C. & Barone, V. Toward reliable density functional methods without adjustable parameters: The PBE0 model. *J. Chem. Phys.* **110**, 6158–6170 (1999).

29. Perdew, J. P., Burke, K. & Ernzerhof, M. Generalized Gradient Approximation Made Simple. *Phys. Rev. Lett.* **77**, 3865–3868 (1996).

30. Grimme, S., Ehrlich, S. & Goerigk, L. Effect of the damping function in dispersion corrected density functional theory. *J. Comput. Chem.* **32**, 1456–1465 (2011).

31. Neese, F., Wennmohs, F., Hansen, A. & Becker, U. Efficient, approximate and parallel Hartree–Fock and hybrid DFT calculations. A ‘chain-of-spheres’ algorithm for the Hartree–Fock exchange. *Chem. Phys.* **356**, 98–109 (2009).

32. Zheng, J., Xu, X. & Truhlar, D. G. Minimally augmented Karlsruhe basis sets. *Theor. Chem. Acc.* **128**, 295–305 (2011).

33. Madhavi Sastry, G., Adzhigirey, M., Day, T., Annabhimoju, R. & Sherman, W. Protein and ligand preparation: parameters, protocols, and influence on virtual screening enrichments. *J. Comput. Aided. Mol. Des.* **27**, 221–234 (2013).

34. Jacobson, M. P., Friesner, R. A., Xiang, Z. & Honig, B. On the Role of the Crystal Environment in Determining Protein Side-chain Conformations. *J. Mol. Biol.* **320**, 597–608 (2002).

35. Jacobson, M. P. *et al.* A hierarchical approach to all-atom protein loop prediction. *Proteins Struct. Funct. Bioinforma.* **55**, 351–367 (2004).

36. Olsson, M. H. M., Søndergaard, C. R., Rostkowski, M. & Jensen, J. H. PROPKA3: Consistent Treatment of Internal and Surface Residues in Empirical p K a Predictions. *J. Chem. Theory Comput.* **7**, 525–537 (2011).

37. Zhu, K. *et al.* Docking Covalent Inhibitors: A Parameter Free Approach To Pose Prediction and Scoring. *J. Chem. Inf. Model.* **54**, 1932–1940 (2014).

38. Bashford, D. & Case, D. A. Generalized Born Models of Macromolecular Solvation Effects. *Annu. Rev. Phys. Chem.* **51**, 129–152 (2000).

39. Wang, E. *et al.* End-Point Binding Free Energy Calculation with MM/PBSA and MM/GBSA: Strategies and Applications in Drug Design. *Chem. Rev.* **119**, 9478–9508 (2019).

40. Friesner, R. A. *et al.* Glide: A New Approach for Rapid, Accurate Docking and Scoring. 1. Method and Assessment of Docking Accuracy. *J. Med. Chem.* **47**, 1739–1749 (2004).

41. Halgren, T. A. *et al.* Glide: A New Approach for Rapid, Accurate Docking and Scoring. 2. Enrichment Factors in Database Screening. *J. Med. Chem.* **47**, 1750–1759 (2004).

42. Shelley, J. C. *et al.* Epik: a software program for pK a prediction and protonation state generation for drug-like molecules. *J. Comput. Aided. Mol. Des.* **21**, 681–691 (2007).

43. Martyna, G. J., Klein, M. L. & Tuckerman, M. Nosé–Hoover chains: The canonical ensemble via continuous dynamics. *J. Chem. Phys.* **97**, 2635–2643 (1992).

44. Martyna, G. J., Tobias, D. J. & Klein, M. L. Constant pressure molecular dynamics algorithms. *J. Chem. Phys.* **101**, 4177–4189 (1994).

45. Essmann, U. *et al.* A smooth particle mesh Ewald method. *J. Chem. Phys.* **103**, 8577–8593 (1995).

46. Keiff, F., Jacques dit Lapierre, T. J. W., Bernal, F. A. & Kloss, F. Design and synthesis of benzofuran- and naphthalene-fused thiazinones as antimycobacterial agents. *Arch. Pharm. (Weinheim).* **356**, (2023).

47. Kelly, C. P., Cramer, C. J. & Truhlar, D. G. Aqueous Solvation Free Energies of Ions and Ion−Water Clusters Based on an Accurate Value for the Absolute Aqueous Solvation Free Energy of the Proton. *J. Phys. Chem. B* **110**, 16066–16081 (2006).

48. Marenich, A. V., Ho, J., Coote, M. L., Cramer, C. J. & Truhlar, D. G. Computational electrochemistry: prediction of liquid-phase reduction potentials. *Phys. Chem. Chem. Phys.* **16**, 15068–15106 (2014).

49. Leung, S. S. F., Mijalkovic, J., Borrelli, K. & Jacobson, M. P. Testing Physical Models of Passive Membrane Permeation. *J. Chem. Inf. Model.* **52**, 1621–1636 (2012).

50. Leung, S. S. F., Sindhikara, D. & Jacobson, M. P. Simple Predictive Models of Passive Membrane Permeability Incorporating Size-Dependent Membrane-Water Partition. *J. Chem. Inf. Model.* **56**, 924–929 (2016).
