# Supplementary material for: Modulation of the Meisenheimer complex metabolism of nitro-benzothiazinones by targeted C-6 substitution
Source: Commun Chem. 2024 Jul 6;7:153. doi: 10.1038/s42004-024-01235-x (PMC11227536; doi:10.1038/s42004-024-01235-x)
Supplement: Supplementary file 2 — Description of Additional Supplementary Files [file 42004_2024_1235_MOESM2_ESM.pdf]

Description of Additional Supplementary Files

File name: Supplementary Data 1

Description: NMR Manuscript.

File name: Supplementary Data 2

Description: X-Ray structure of compound 22.

File name: Supplementary Data 3

Description: X-Ray structure of compound S7.

File name: Supplementary Data 4

Description: X-Ray structure of compound S5.

File name: Supplementary Data 5

Description: Cartesian Coordinates.

File name: Supplementary Data 6

Description: Cheminformatics Calculated Data
