# Supplementary material for: Modulation of the Meisenheimer complex metabolism of nitro-benzothiazinones by targeted C-6 substitution
Source: Commun Chem. 2024 Jul 6;7:153. doi: 10.1038/s42004-024-01235-x (PMC11227536; doi:10.1038/s42004-024-01235-x)
Supplement: Supplementary file 3 — Supplementary Data 1 [file 42004_2024_1235_MOESM3_ESM.docx]

**Supplementary Information**

**Modulation of the Meisenheimer complex metabolism of nitro-benzothiazinones by targeted C-6 substitution**

François Keiff,^1^ Freddy A. Bernal,^1^ Melanie Joch,^1^ Thibault Joseph William Jacques-dit-Lapierre, ^1^ Yan Li,^1^ Phil Liebing,^2^ Hans-Martin Dahse,^3^ Ivan Vilotijevic,^4^ Florian Kloss*^1^

^1^ Transfer Group Anti-infectives and 3Infection Biology, Leibniz Institute for Natural Products Research and Infection Biology – Leibniz-HKI, Beutenbergstr. 11a, 07745 Jena, Germany.

^2^ Institute for Inorganic and Analytical Chemistry, Friedrich-Schiller-Universität Jena, Humboldtstr. 8, 07743 Jena, Germany.

^3^ Department of Infection Biology, Leibniz Institute for Natural Product Research and Infection Biology – Leibniz-HKI, Beutenbergstr. 11a, 07745 Jena, Germany.

^4^ Institute of Organic Chemistry and Macromolecular Chemistry, Friedrich Schiller University Jena, Humboldtstr. 10, Jena 07743, Germany.

**Supplementary Data 1**

**NMR Spectra**

**Table of content**

[Supporting Figure DS1 ^1^H NMR of compound **S1** (300 MHz, 300K, CD_3_OD). 4](#_Toc169855918)

[Supporting Figure DS2. ^13^C NMR of compound **S1** (75 MHz, 300K, CD_3_OD). 5](#_Toc169855919)

[Supporting Figure DS3 ^1^H NMR of compound **12** (300 MHz, 300K, CD_3_OD). 6](#_Toc169855920)

[Supporting Figure DS4. ^13^C NMR of compound **12** (75 MHz, 300K, CD_3_OD). 7](#_Toc169855921)

[Supporting Figure DS5. ^1^H NMR of compound **S2** (300 MHz, 300K, CD_3_OD). 8](#_Toc169855922)

[Supporting Figure DS6. ^13^C NMR of compound **S2** (75 MHz, 300K, CD_3_OD). 9](#_Toc169855923)

[Supporting Figure DS7. ^1^H NMR of compound **15** (300 MHz, 300K, CD_3_OD). 10](#_Toc169855924)

[Supporting Figure DS8. ^13^C NMR of compound **15** (75 MHz, 300K, CD_3_OD). 11](#_Toc169855925)

[Supporting Figure DS9. ^1^H NMR of compound **18** (500 MHz, 300K, DMSO-*d_6_*). 12](#_Toc169855926)

[Supporting Figure DS10. ^13^C NMR of compound **18** (126 MHz, 300K, DMSO-*d_6_*). 13](#_Toc169855927)

[Supporting Figure DS11. ^1^H NMR of compound **9** (600 MHz, 300K, CDCl_3_ + 1% TMS). 14](#_Toc169855928)

[Supporting Figure DS12. ^13^C NMR of compound **9** (151 MHz, 300K, CDCl_3_ + 1% TMS). 15](#_Toc169855929)

[Supporting Figure DS13. ^1^H NMR of compound **13** (300 MHz, 300K, CD_3_OD). 16](#_Toc169855930)

[Supporting Figure DS14. ^13^C NMR of compound **13** (75 MHz, 300K, CD_3_OD). 17](#_Toc169855931)

[Supporting Figure DS15. ^1^H NMR of compound **14** (300 MHz, 300K, CD_3_OD). 18](#_Toc169855932)

[Supporting Figure DS16. ^13^C NMR of compound **14** (75 MHz, 300K, CD_3_OD). 19](#_Toc169855933)

[Supporting Figure DS17. ^1^H NMR of compound **S3** (300 MHz, 300K, CD_3_OD). 20](#_Toc169855934)

[Supporting Figure DS18. ^13^C NMR of compound **S3** (75 MHz, 300K, CD_3_OD). 21](#_Toc169855935)

[Supporting Figure DS19. ^1^H NMR of compound **S4** (500 MHz, 300K, CD_3_OD). 22](#_Toc169855936)

[Supporting Figure DS20. ^13^C NMR of compound **S4** (126 MHz, 300K, CD_3_OD). 23](#_Toc169855937)

[Supporting Figure DS21. ^1^H NMR of compound **16** (500 MHz, 300K, DMSO-*d_6_*). 24](#_Toc169855938)

[Supporting Figure DS22. ^13^C NMR of compound **16** (126 MHz, 300K, DMSO-*d_6_*). 25](#_Toc169855939)

[Supporting Figure DS23. ^1^H NMR of compound **17** (500 MHz, 300K, CD_3_OD). 26](#_Toc169855940)

[Supporting Figure DS24. ^13^C NMR of compound **17** (126 MHz, 300K, CD_3_OD). 27](#_Toc169855941)

[Supporting Figure DS25. ^1^H NMR of compound **19** (300 MHz, 300K, DMSO-*d_6_*). 28](#_Toc169855942)

[Supporting Figure DS26. ^13^C NMR of compound **19** (75 MHz, 300K, DMSO-*d_6_*). 29](#_Toc169855943)

[Supporting Figure DS27. ^1^H NMR of compound **20** (500 MHz, 300K, CDCl_3_ + 1% TMS). 30](#_Toc169855944)

[Supporting Figure DS28. ^13^C NMR of compound **20** (126 MHz, 300K, CDCl3 + 1% TMS). 31](#_Toc169855945)

[Supporting Figure DS29. ^1^H NMR of compound **10** (300 MHz, 300K, DMSO-*d_6_*). 32](#_Toc169855946)

[Supporting Figure DS30. ^13^C NMR of compound **10** (75 MHz, 300K, DMSO-*d_6_*). 33](#_Toc169855947)

[Supporting Figure DS31. ^1^H NMR of compound **11** (300 MHz, 300K, CDCl_3_). 34](#_Toc169855948)

[Supporting Figure DS32. ^13^C NMR of compound **11** (75 MHz, 300K, CDCl_3_). 35](#_Toc169855949)

[Supporting Figure DS33. ^1^H NMR of compound **8** (500 MHz, 300K, DMSO-*d_6_*). 36](#_Toc169855950)

[Supporting Figure DS34. ^13^C NMR of compound **8** (126 MHz, 300K, DMSO-*d_6_*). 37](#_Toc169855951)

[Supporting Figure DS35. ^1^H NMR of compound **24** (500 MHz, 300K, CDCl_3_). 38](#_Toc169855952)

[Supporting Figure DS36. ^13^C NMR of compound **24** (126 MHz, 300K, CDCl_3_). 39](#_Toc169855953)

[Supporting Figure DS37. ^1^H NMR of compound **22** (500 MHz, 300K, CDCl_3_ + 1% TMS). 40](#_Toc169855954)

[Supporting Figure DS38. ^13^C NMR of compound **22** (126 MHz, 300K, CDCl_3_ + 1% TMS). 41](#_Toc169855955)

[Supporting Figure DS39. ^31^P NMR of compound **22** (202 MHz, 300K, CDCl_3_ + 1% TMS). 42](#_Toc169855956)

[Supporting Figure DS40. COSY experiment of compound **22** (300K, CDCl3 + 1% TMS). 43](#_Toc169855957)

[Supporting Figure DS41. HSQC experiment of compound **22** (300K, CDCl_3_ + 1% TMS). 44](#_Toc169855958)

[Supporting Figure DS42. HMBC experiment of compound **22** (300K, CDCl_3_ + 1% TMS). 45](#_Toc169855959)

[Supporting Figure DS43. ^1^H NMR of compound **S7** (500 MHz, 300K, CDCl_3_ + 1% TMS). 46](#_Toc169855960)

[Supporting Figure DS44. ^13^C NMR of compound **S7** (126 MHz, 300K, CDCl_3_ + 1% TMS). 47](#_Toc169855961)

[Supporting Figure DS45. ^31^P NMR of compound **S7** (202 MHz, 300K, CDCl_3_ + 1% TMS). 48](#_Toc169855962)

[Supporting Figure DS46. COSY experiment of compound **S7** (300K, CDCl_3_ + 1% TMS). 49](#_Toc169855963)

[Supporting Figure DS47. HSQC experiment of compound **S7** (300K, CDCl_3_ + 1% TMS). 50](#_Toc169855964)

[Supporting Figure DS48. HMBC experiment of compound **S7** (300K, CDCl_3_ + 1% TMS). 51](#_Toc169855965)

[Supporting Figure DS49. ^1^H NMR of compound **S5** (500 MHz, 300K, CDCl_3_ + 1% TMS). 52](#_Toc169855966)

[Supporting Figure DS50. ^13^C NMR of compound **S5** (126 MHz, 300K, CDCl_3_ + 1% TMS). 53](#_Toc169855967)

[Supporting Figure DS51. COSY experiment of compound **S5** (300K, CDCl_3_ + 1% TMS). 54](#_Toc169855968)

[Supporting Figure DS52. HSQC experiment of compound **S5** (300K, CDCl_3_ + 1% TMS). 55](#_Toc169855969)

[Supporting Figure DS53. HMBC experiment of compound **S5** (300K, CDCl_3_ + 1% TMS). 56](#_Toc169855970)

[Supporting Figure DS54. ^1^H NMR of compound **6a** (500 MHz, 300K, CDCl_3_ + 1% TMS). 57](#_Toc169855971)

[Supporting Figure DS55. ^13^C NMR of compound **6a** (126 MHz, 300K, CDCl_3_ + 1% TMS). 58](#_Toc169855972)

[Supporting Figure DS56. ^1^H NMR of compound **6b** (500 MHz, 300K, CDCl_3_). 59](#_Toc169855973)

[Supporting Figure DS57. ^1^H NMR of compound **6b** (126 MHz, 300K, CDCl_3_). 60](#_Toc169855974)

[Supporting Figure DS58. ^1^H NMR of compound **6c** (500 MHz, 300K, CDCl_3_). 61](#_Toc169855975)

[Supporting Figure DS59. ^13^C NMR of compound **6c** (126 MHz, 300K, CDCl_3_). 62](#_Toc169855976)

[Supporting Figure DS60. ^1^H NMR of compound **6d** (600 MHz, 300K, DMSO-*d_6_*). 63](#_Toc169855977)

[Supporting Figure DS61. ^13^C NMR of compound **6d** (151 MHz, 300K, DMSO-*d_6_*). 64](#_Toc169855978)

[Supporting Figure DS62. ^1^H NMR of compound **6e** (300 MHz, 300K, CDCl_3_ + 1% TMS). 65](#_Toc169855979)

[Supporting Figure DS63. ^13^C NMR of compound **6e** (75 MHz, 300K, CDCl_3_ + 1% TMS). 66](#_Toc169855980)

[Supporting Figure DS64. ^1^H NMR of compound **6f** (500 MHz, 300K, DMSO-*d_6_*). 67](#_Toc169855981)

[Supporting Figure DS65. ^13^C NMR of compound **6f** (126 MHz, DMSO-*d_6_*). 68](#_Toc169855982)

[Supporting Figure DS66. ^1^H NMR of compound **7a** (600 MHz, 300K, DMSO-*d_6_*). 69](#_Toc169855983)

[Supporting Figure DS67. ^13^C NMR of compound **7a** (151 MHz, 300K, DMSO-*d_6_*). 70](#_Toc169855984)

[Supporting Figure DS68. ^1^H NMR of compound **7b** (500 MHz, 300K, DMSO-*d_6_*). 71](#_Toc169855985)

[Supporting Figure DS69. ^13^C NMR of compound **7b** (126 MHz, 300K, DMSO-*d_6_*). 72](#_Toc169855986)

[Supporting Figure DS70. ^1^H NMR of compound **7c** (500 MHz, 300K, CDCl_3_ + 1% TMS). 73](#_Toc169855987)

[Supporting Figure DS71. ^13^C NMR of compound **7c** (126 MHz, 300K, CDCl_3_ + 1% TMS). 74](#_Toc169855988)

[Supporting Figure DS72. ^13^C NMR of unpurified compound **7c** (75 MHz, 300K, CDCl_3_ + 1% TMS). 75](#_Toc169855989)

[Supporting Figure DS73. ^1^H NMR of compound **7d** (600 MHz, 300K, DMSO-*d_6_*). 76](#_Toc169855990)

[Supporting Figure DS74. ^13^C NMR of compound **7d** (151 MHz, 300K, DMSO-*d_6_*). 77](#_Toc169855991)

[Supporting Figure DS75. ^1^H NMR of compound **7e** (300 MHz, 300K, CDCl_3_). 78](#_Toc169855992)

[Supporting Figure DS76. ^13^C NMR of compound **7e** (75 MHz, 300K, CDCl_3_). 79](#_Toc169855993)

[Supporting Figure DS77. DEPT 135 NMR of compound **7e** (75 MHz, 300K, CDCl_3_). 80](#_Toc169855994)

[Supporting Figure DS78. COSY experiment of compound **7e** (300K, CDCl_3_). 81](#_Toc169855995)

[Supporting Figure DS79. HSQC experiment of compound **7e** (300K, CDCl_3_). 82](#_Toc169855996)

[Supporting Figure DS80. ^1^H NMR of compound **7f** (500 MHz, 300K, CDCl_3_ + 1% TMS). 83](#_Toc169855997)

[Supporting Figure DS81. ^13^C NMR of compound **7f** (126 MHz, 300K, CDCl_3_ + 1% TMS). 84](#_Toc169855998)

[Supporting Figure DS82. ^1^H NMR of unpurified compound **7f** (500 MHz, 300K, CDCl_3_ + 1% TMS). 85](#_Toc169855999)

[Supporting Figure DS83. ^13^C NMR of unpurified compound **7f** (126 MHz, 300K, CDCl_3_ + 1% TMS). 86](#_Toc169856000)


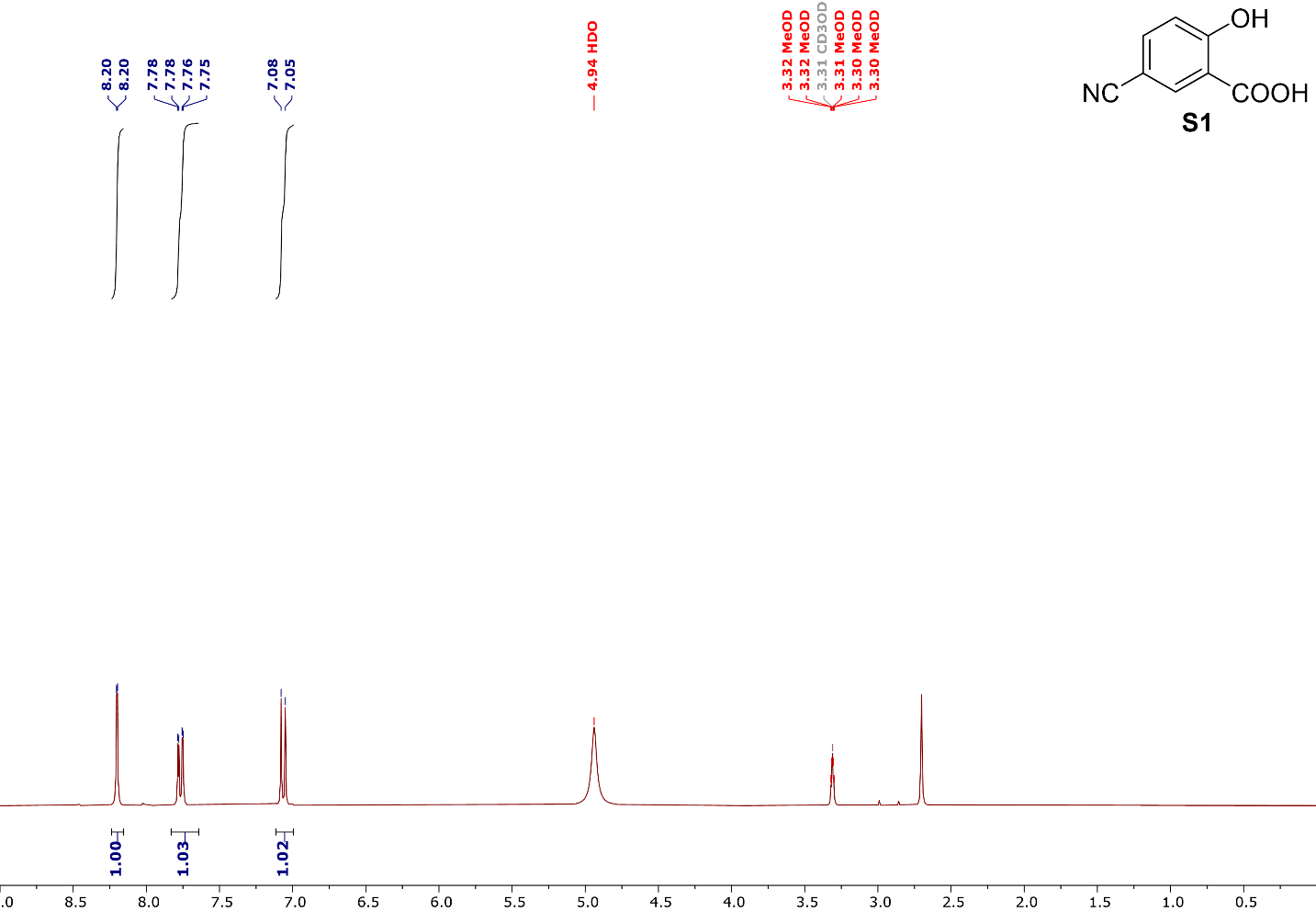


**Supporting Figure DS1** ^1^H NMR of compound S1 (300 MHz, 300K, CD_3_OD).


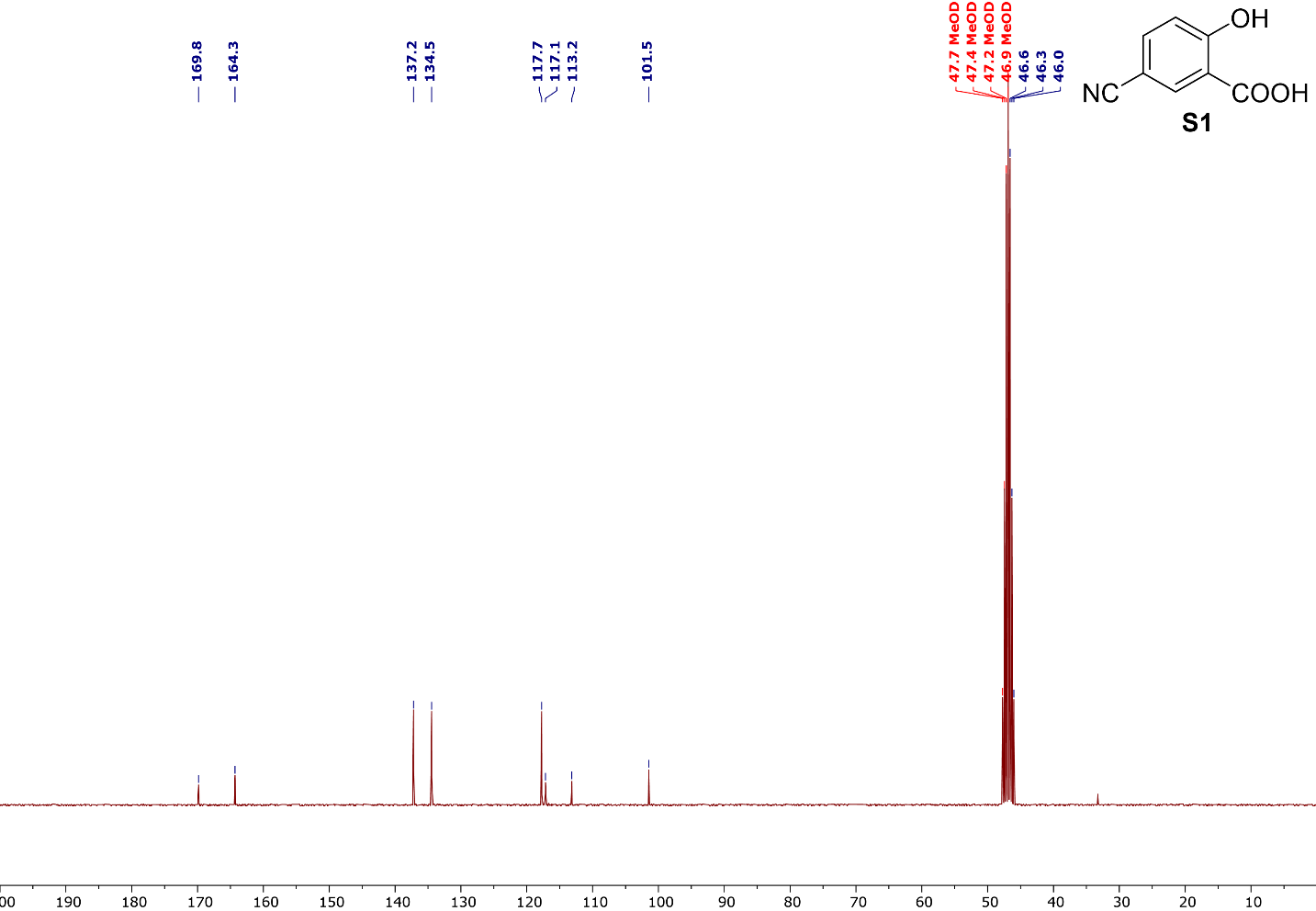


Supporting Figure DS2. ^13^C NMR of compound S1 (75 MHz, 300K, CD_3_OD).


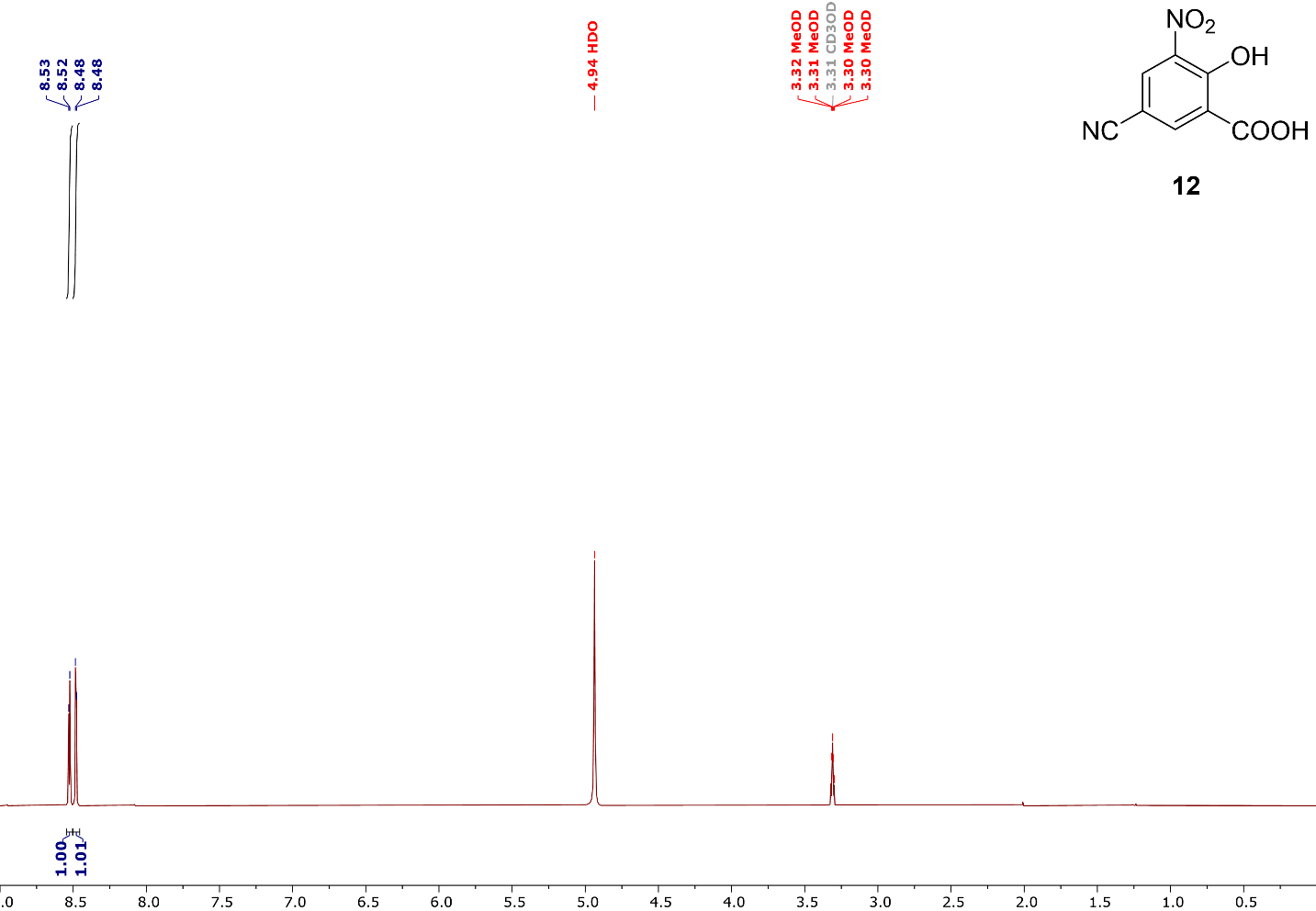


Supporting Figure DS3 ^1^H NMR of compound 12 (300 MHz, 300K, CD_3_OD).


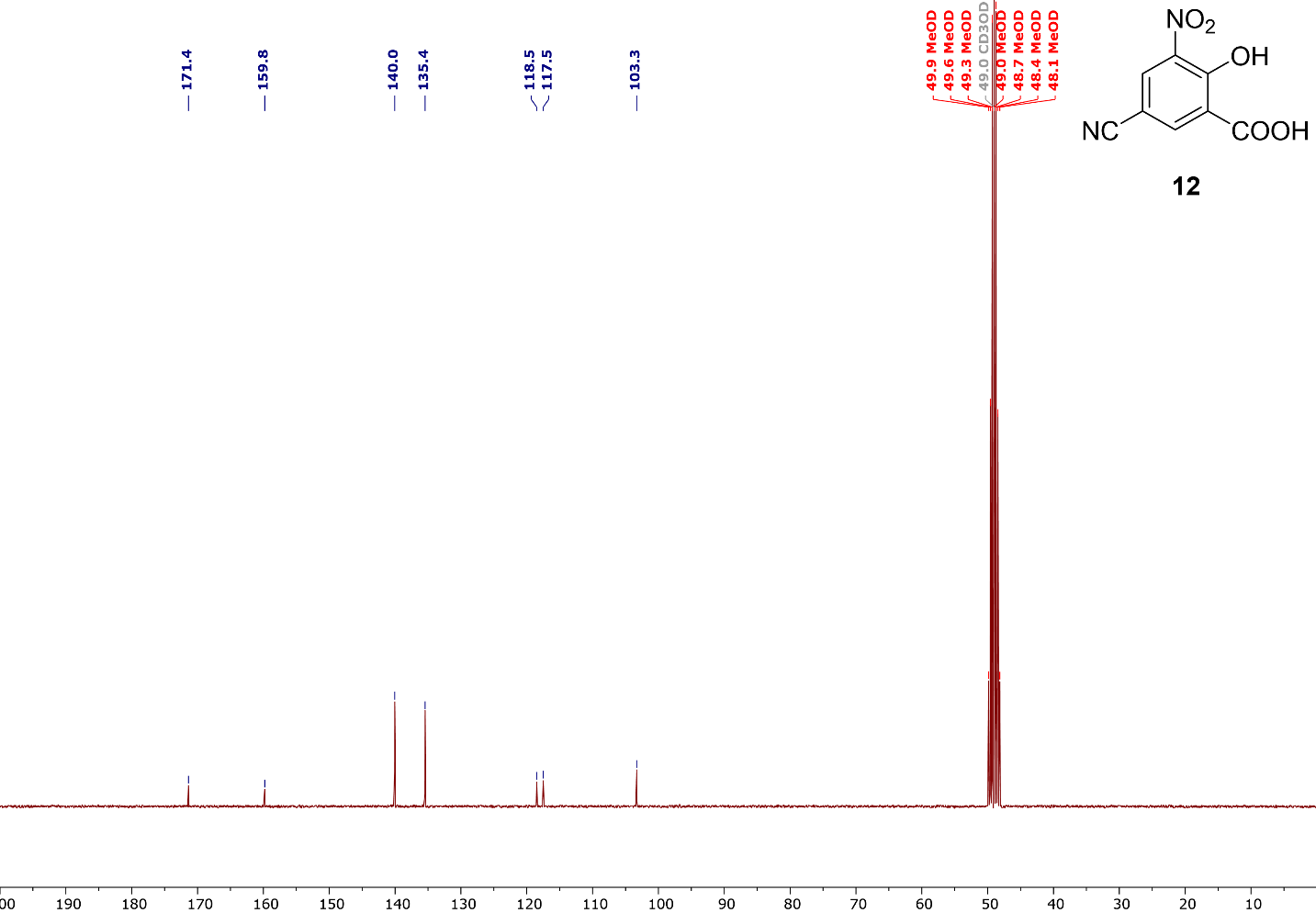


Supporting Figure DS4. ^13^C NMR of compound 12 (75 MHz, 300K, CD_3_OD).


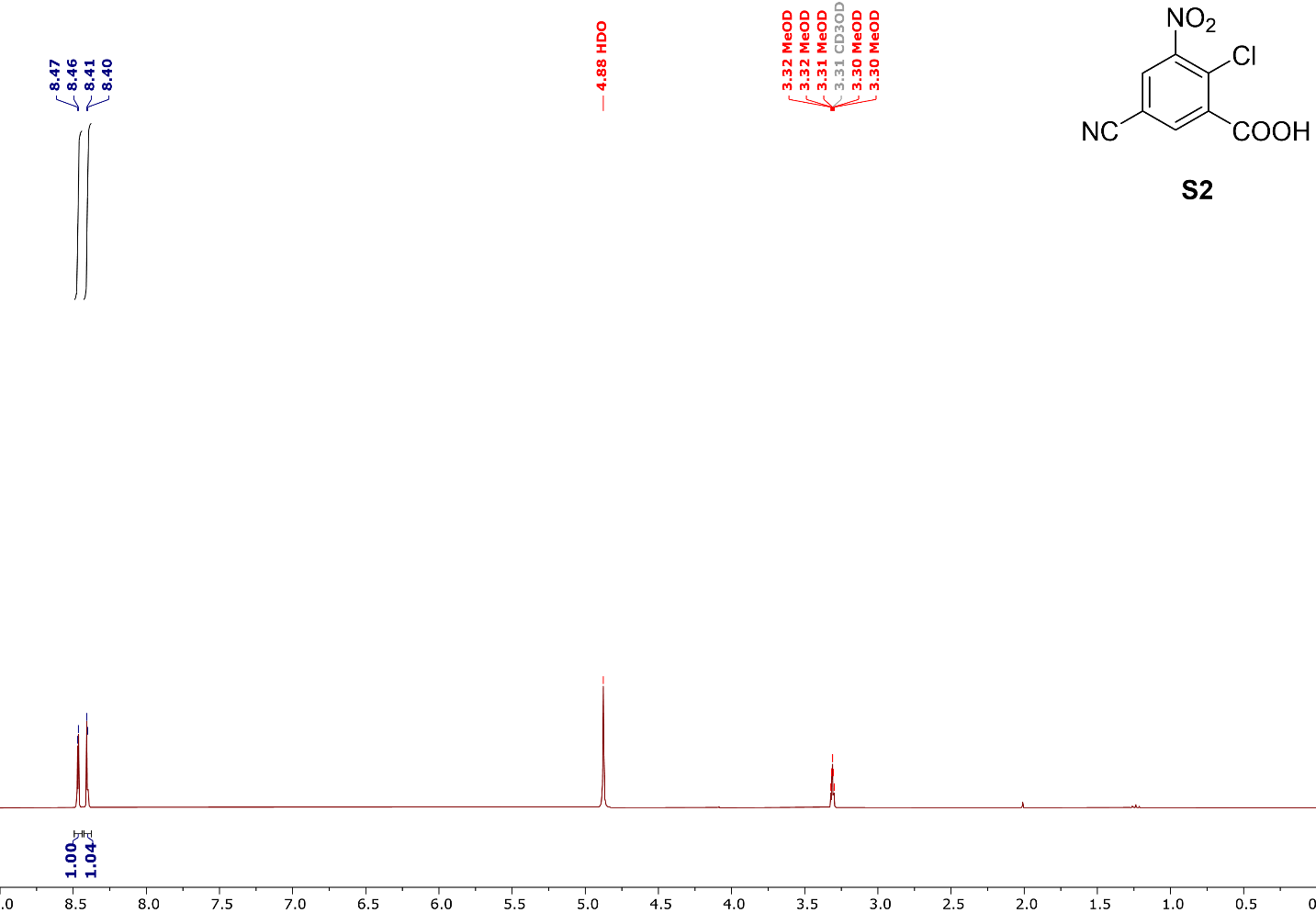


**Supporting Figure DS5.** ^1^H NMR of compound S2 (300 MHz, 300K, CD_3_OD).


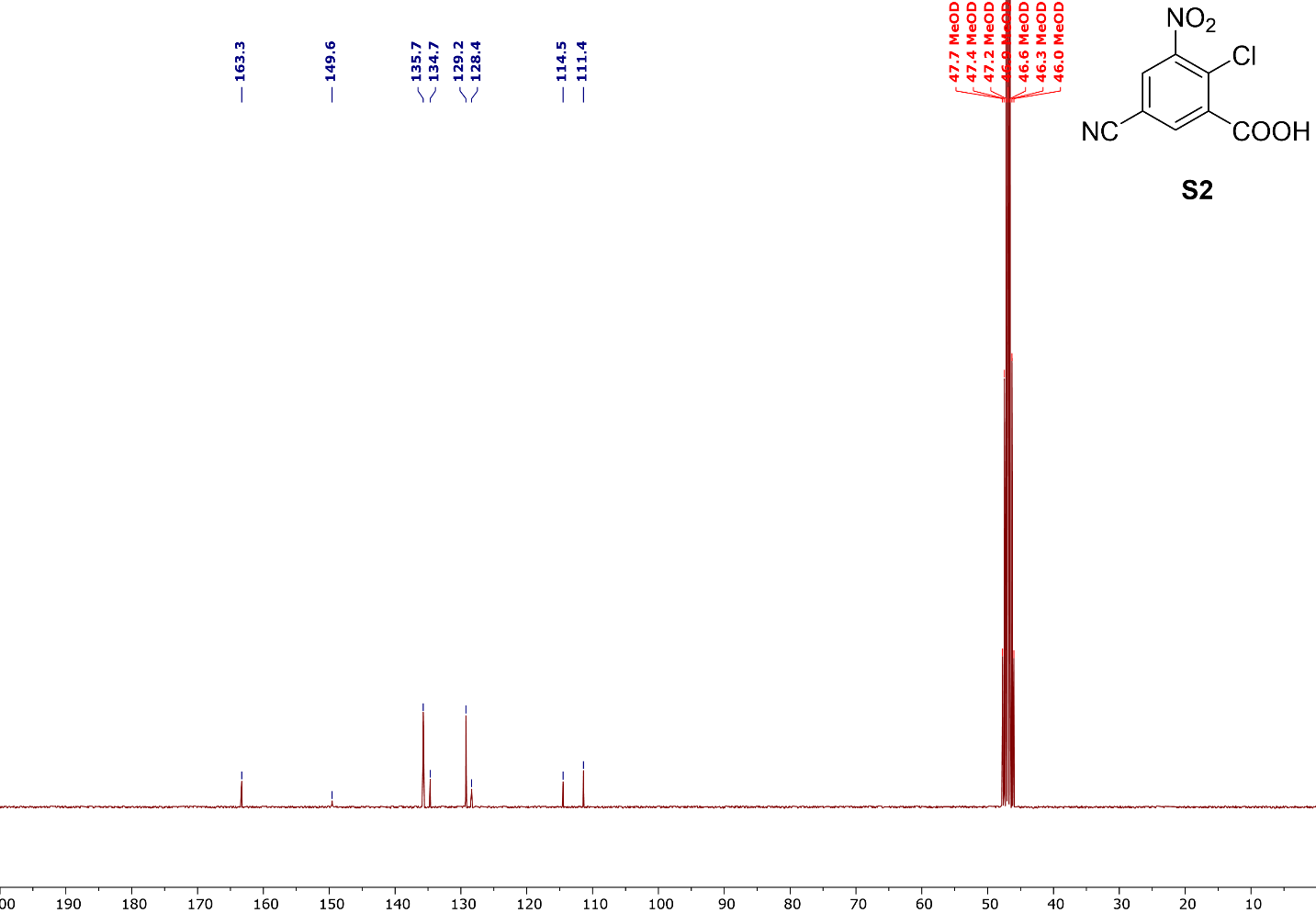


Supporting Figure DS6. ^13^C NMR of compound S2 (75 MHz, 300K, CD_3_OD).


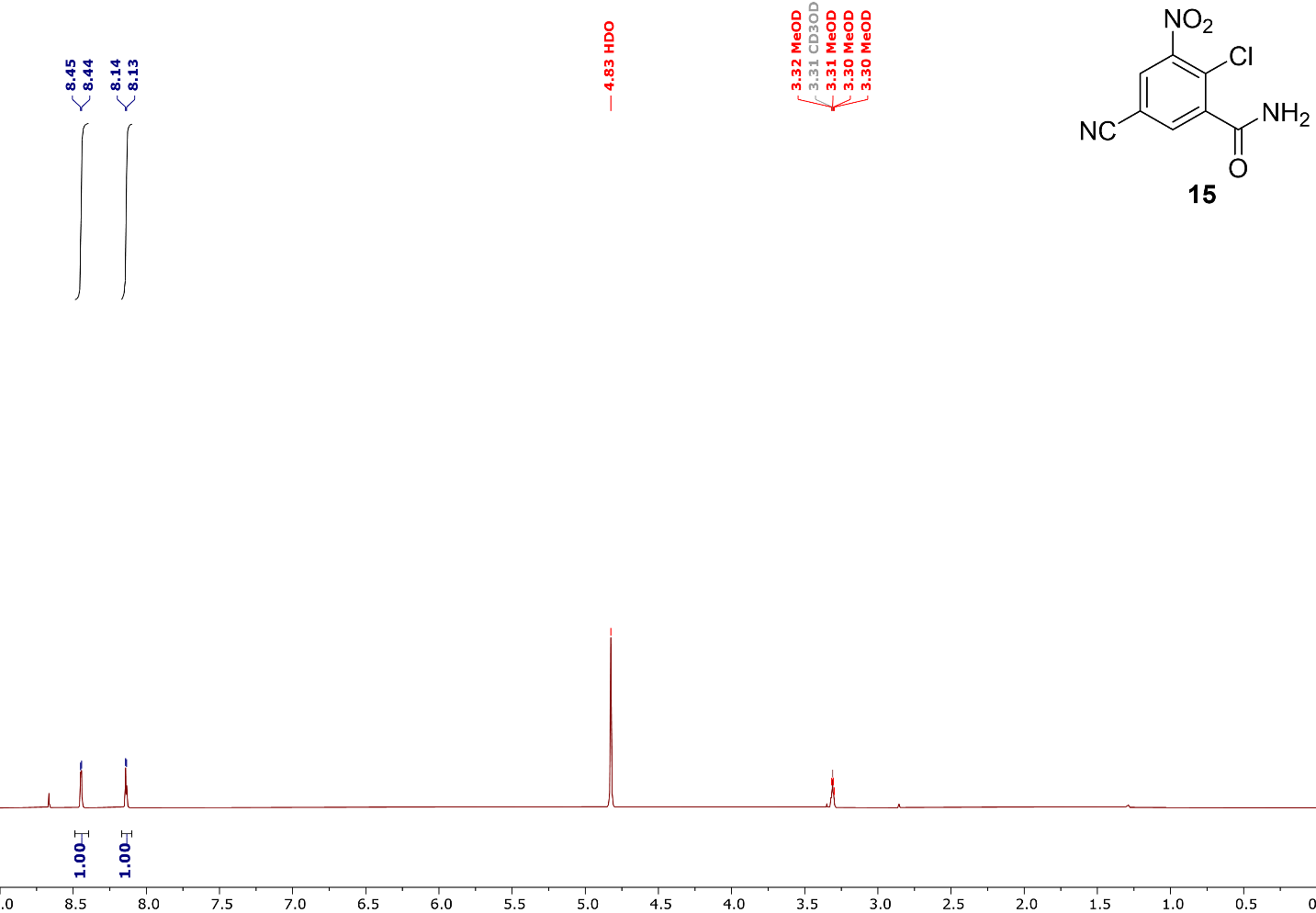


Supporting Figure DS7. ^1^H NMR of compound 15 (300 MHz, 300K, CD_3_OD).


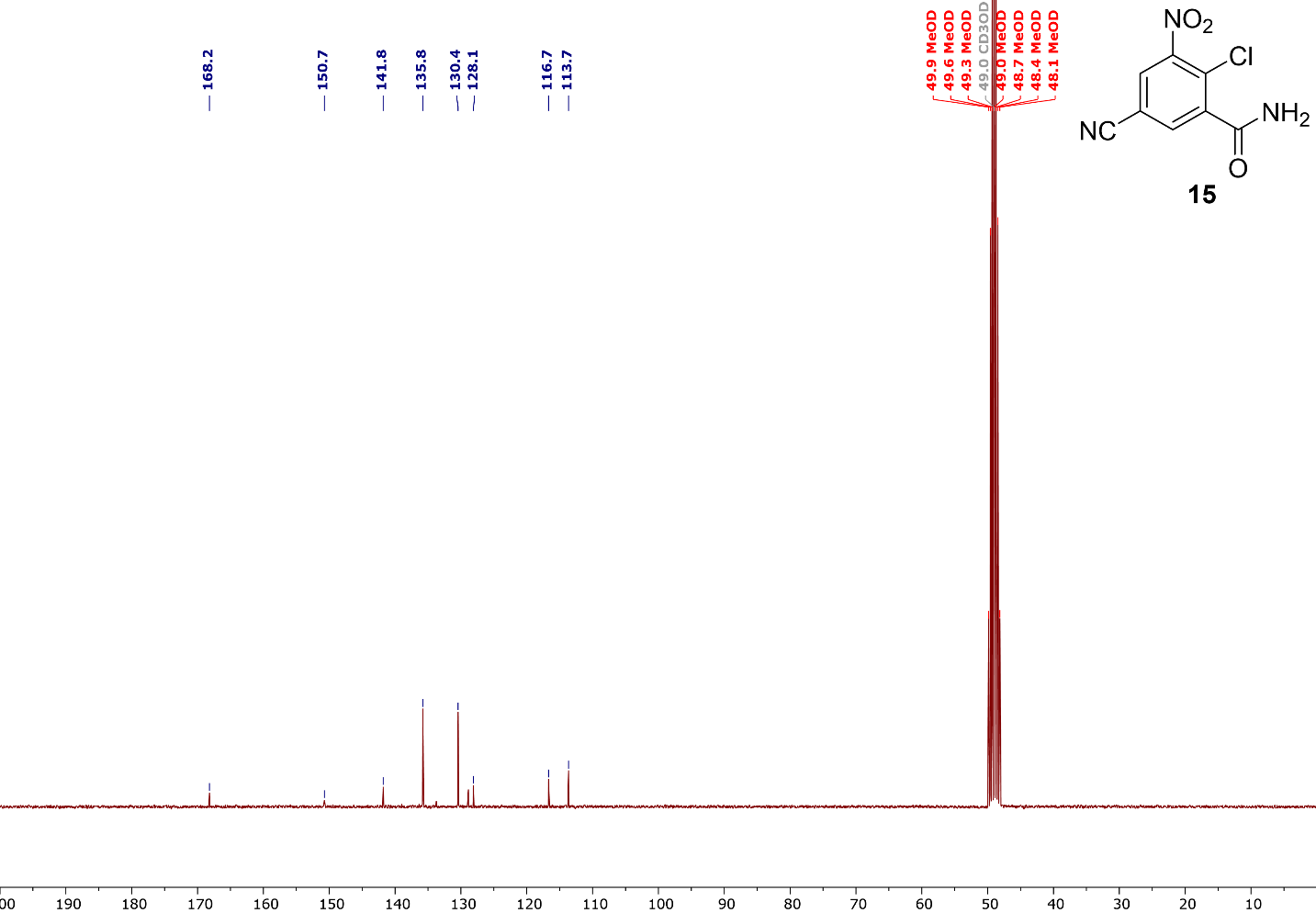


Supporting Figure DS8. ^13^C NMR of compound 15 (75 MHz, 300K, CD_3_OD).


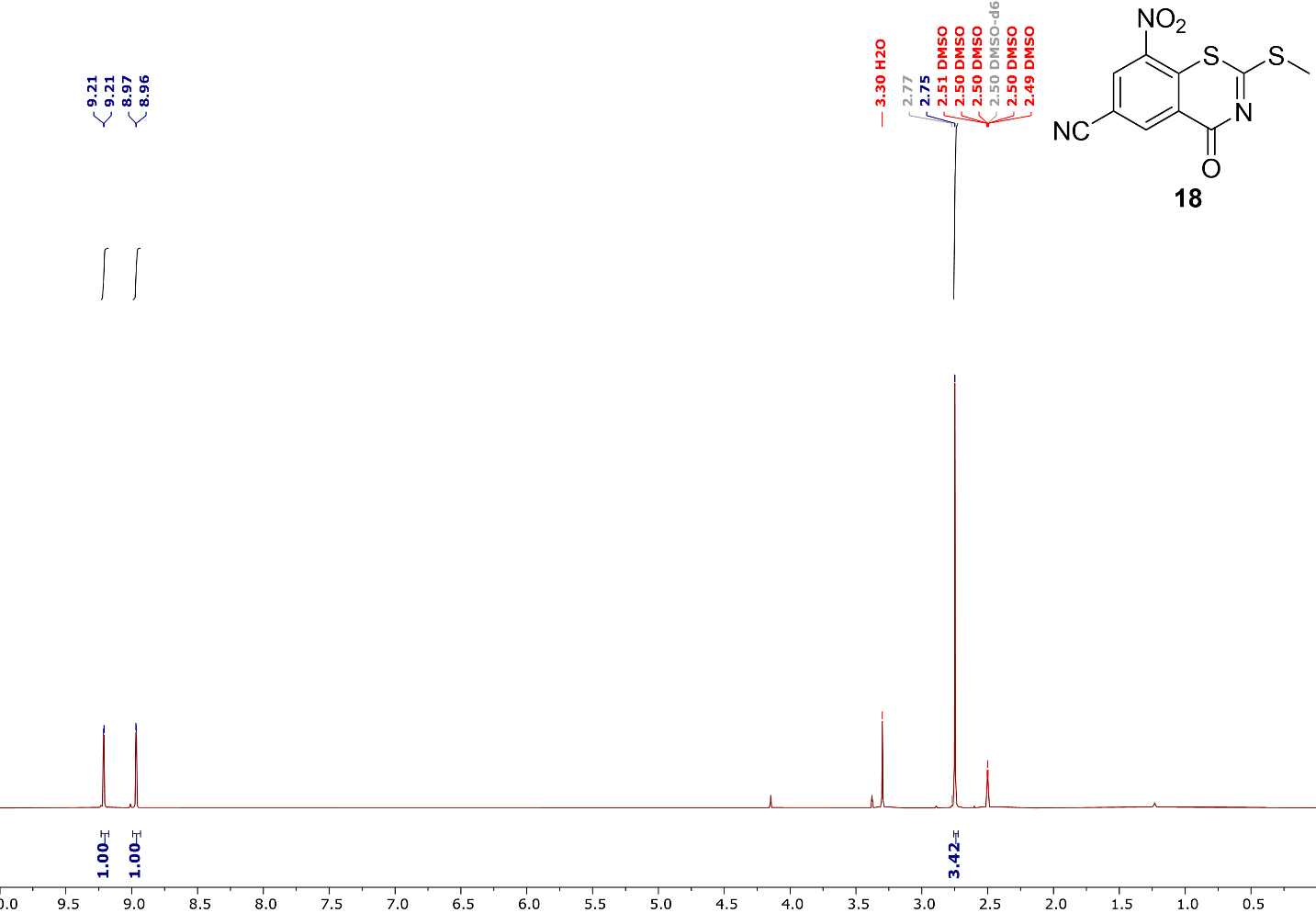


Supporting Figure DS9. ^1^H NMR of compound 18 (500 MHz, 300K, DMSO-*d_6_*).


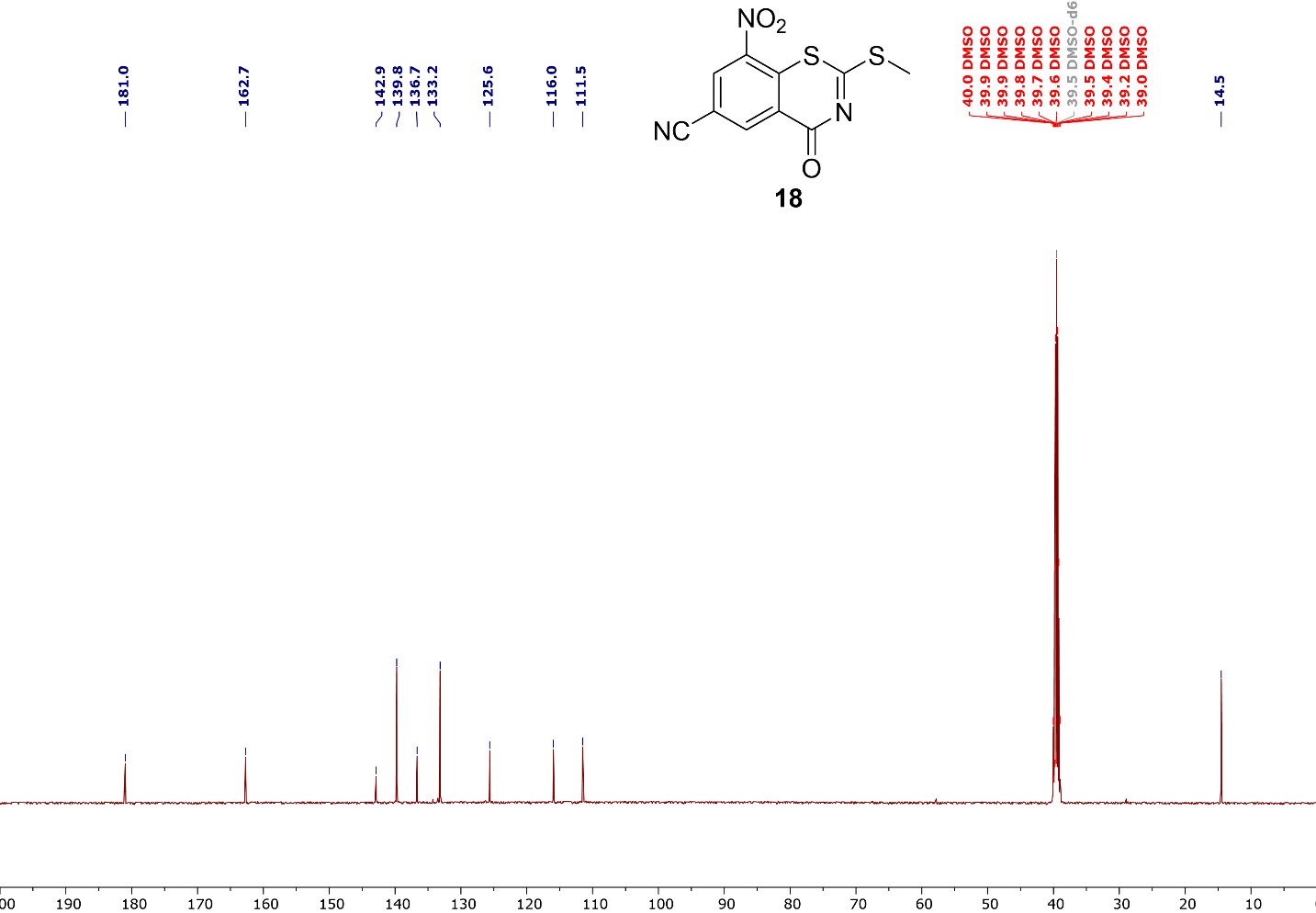


Supporting Figure DS10. ^13^C NMR of compound 18 (126 MHz, 300K, DMSO-*d_6_*).


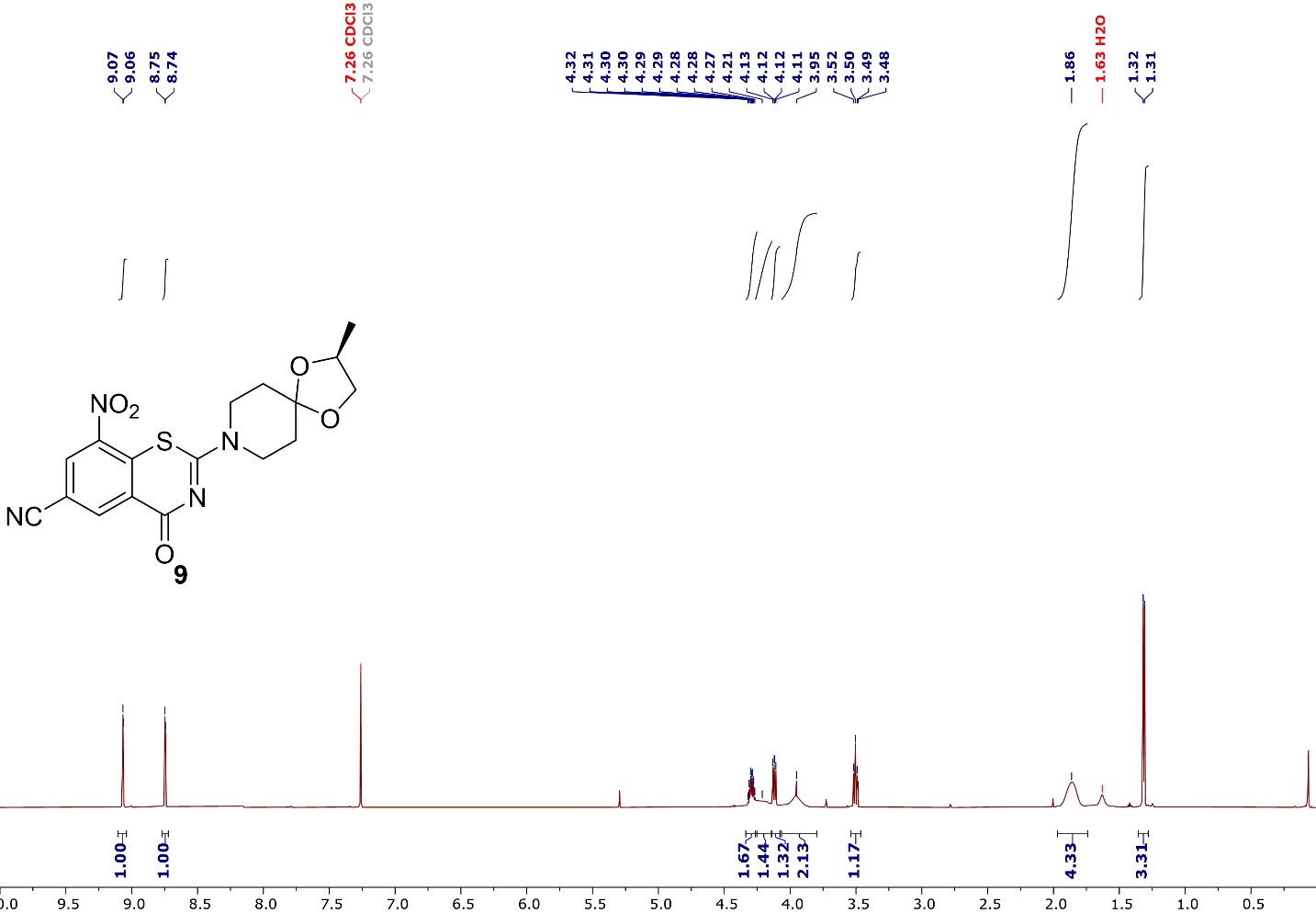


Supporting Figure DS11. ^1^H NMR of compound 9 (600 MHz, 300K, CDCl_3_ + 1% TMS).


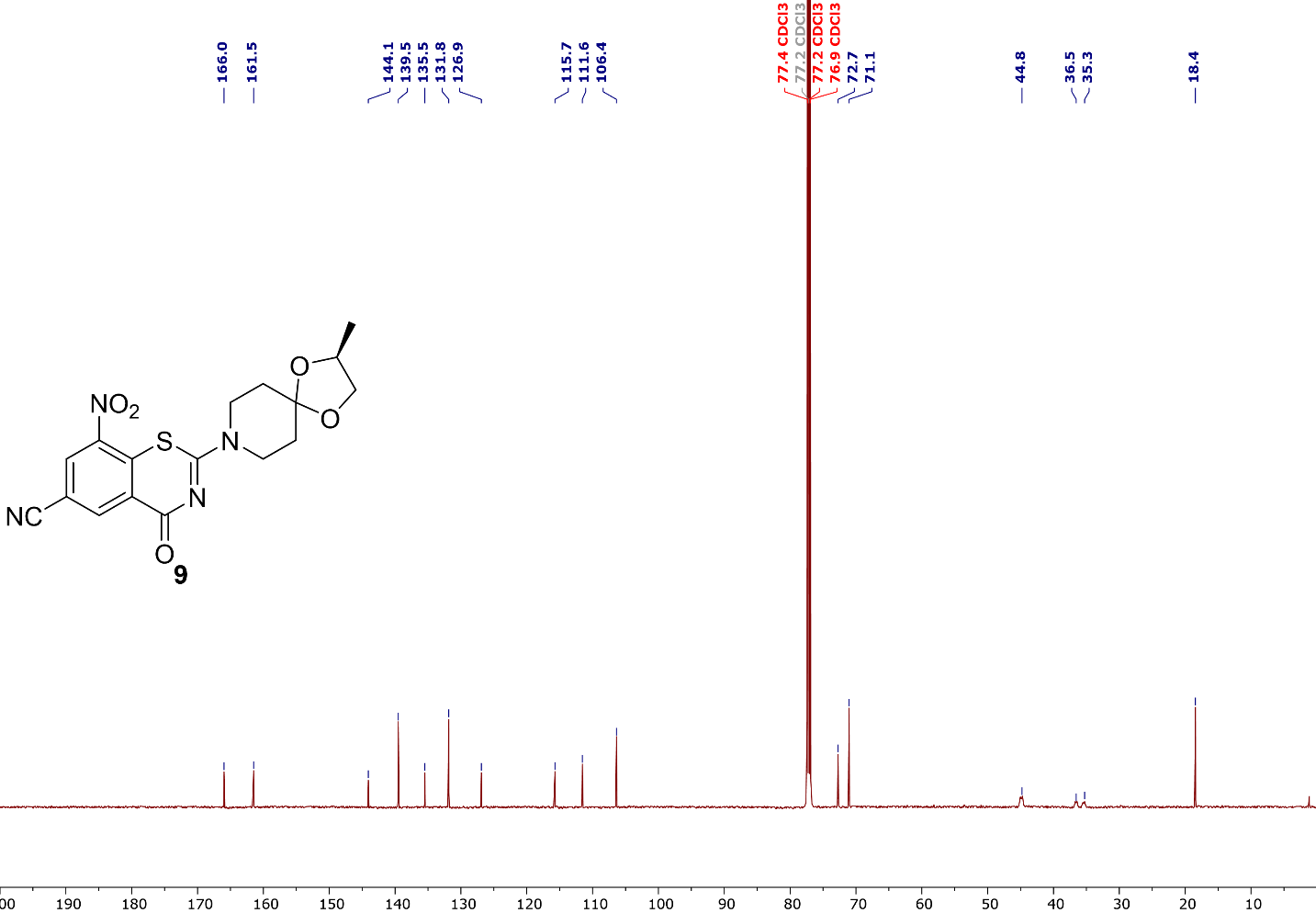


Supporting Figure DS12. ^13^C NMR of compound 9 (151 MHz, 300K, CDCl_3_ + 1% TMS).


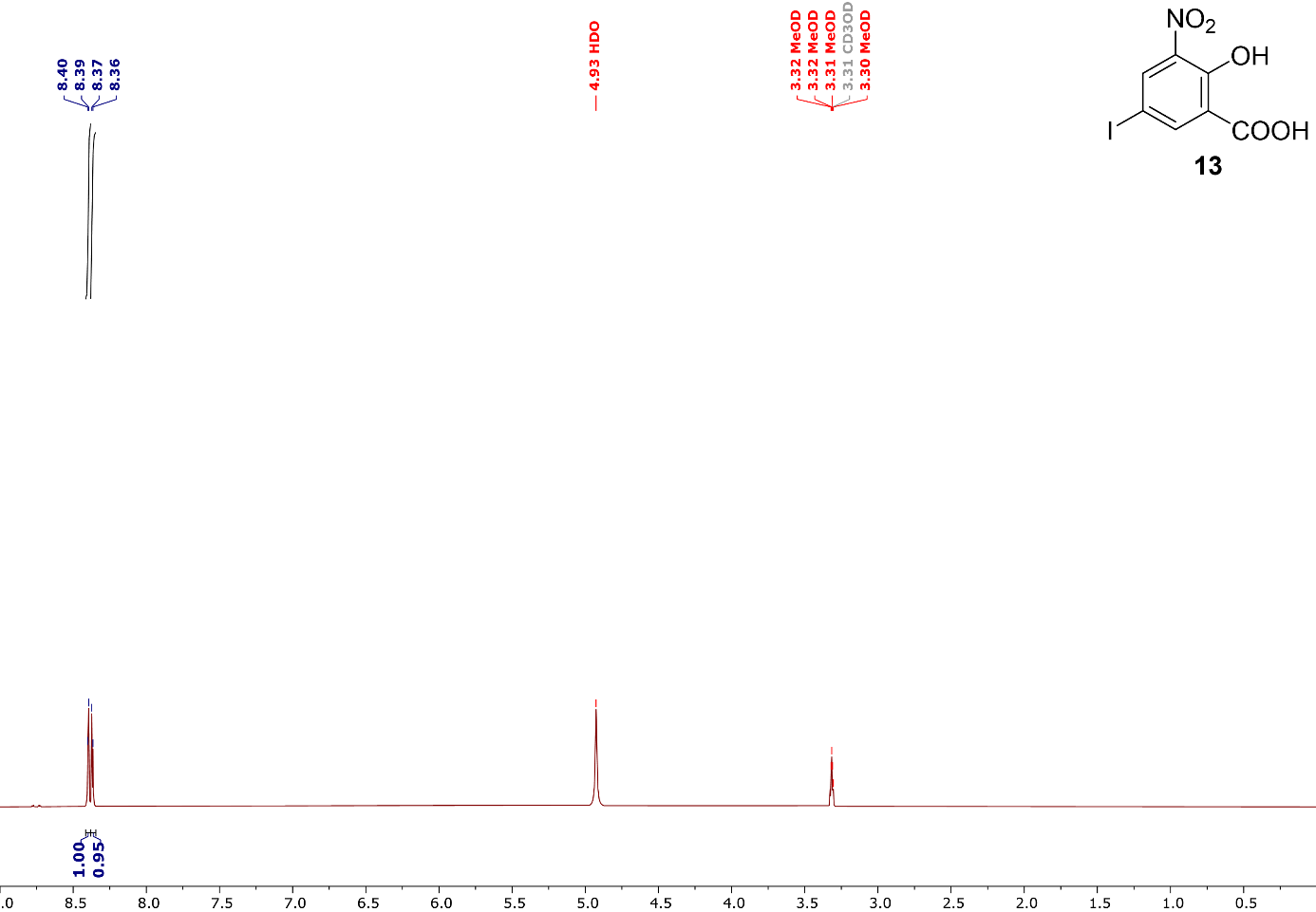


Supporting Figure DS13. ^1^H NMR of compound 13 (300 MHz, 300K, CD_3_OD).


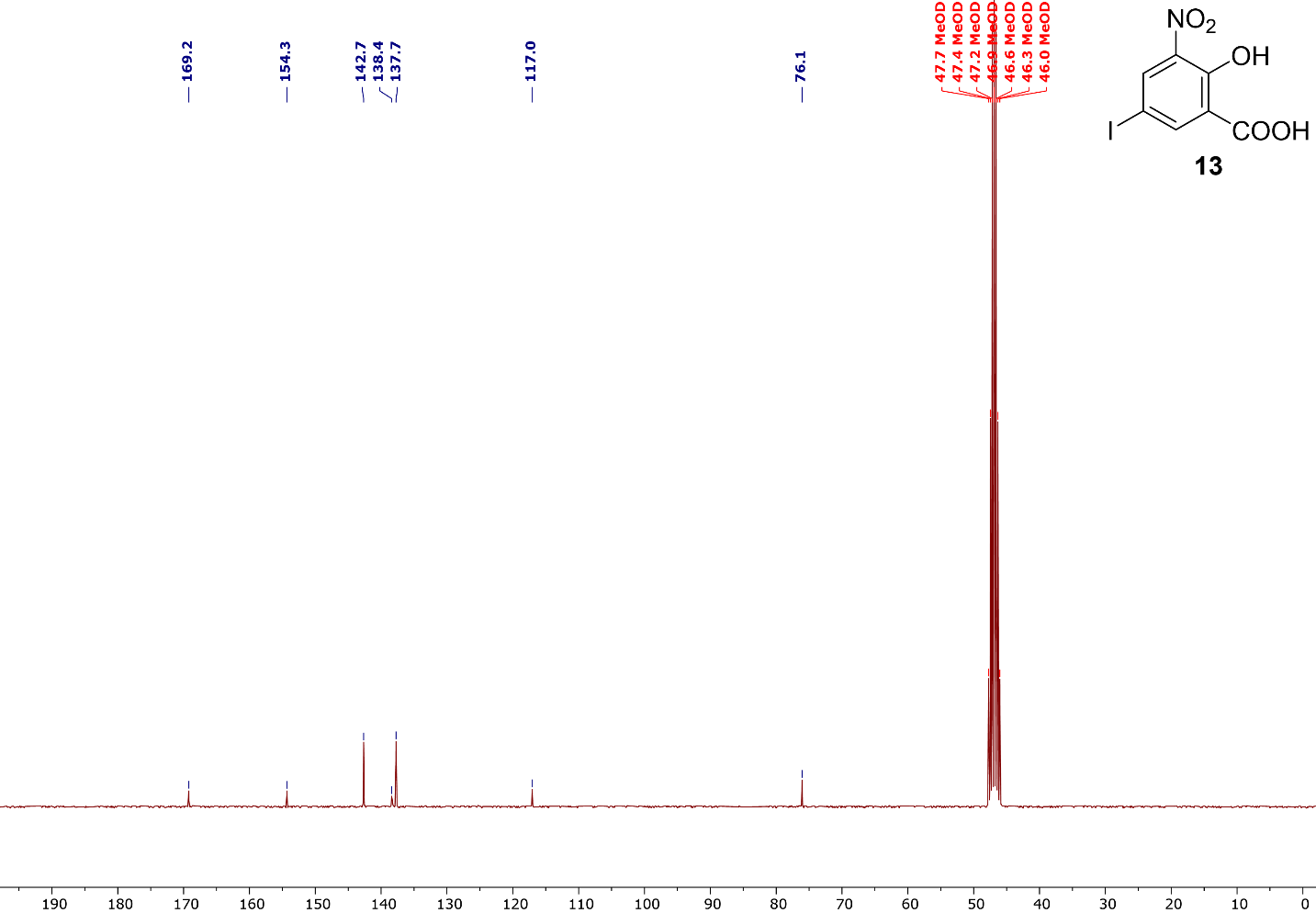


Supporting Figure DS14. ^13^C NMR of compound 13 (75 MHz, 300K, CD_3_OD).


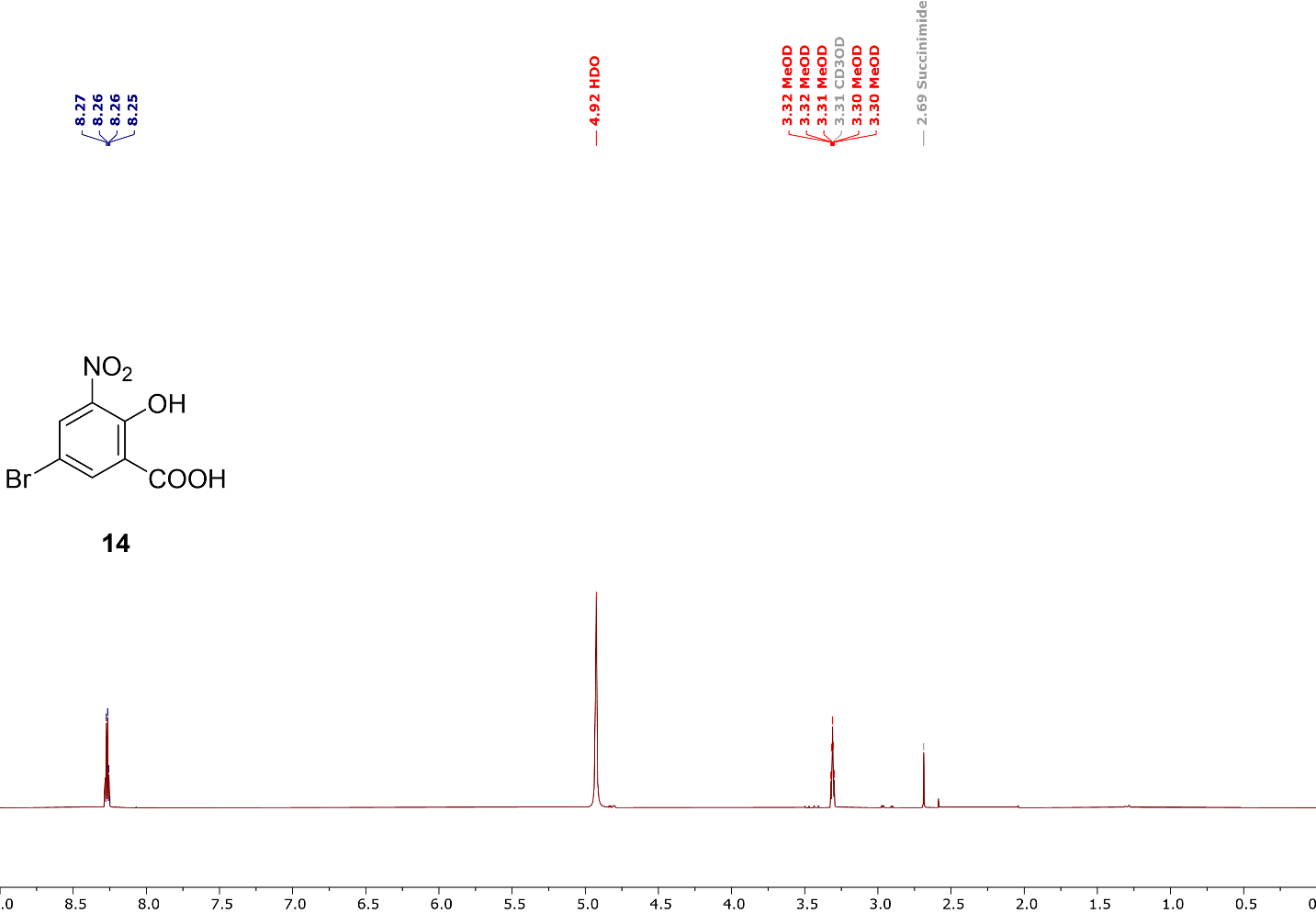


Supporting Figure DS15. ^1^H NMR of compound 14 (300 MHz, 300K, CD_3_OD).


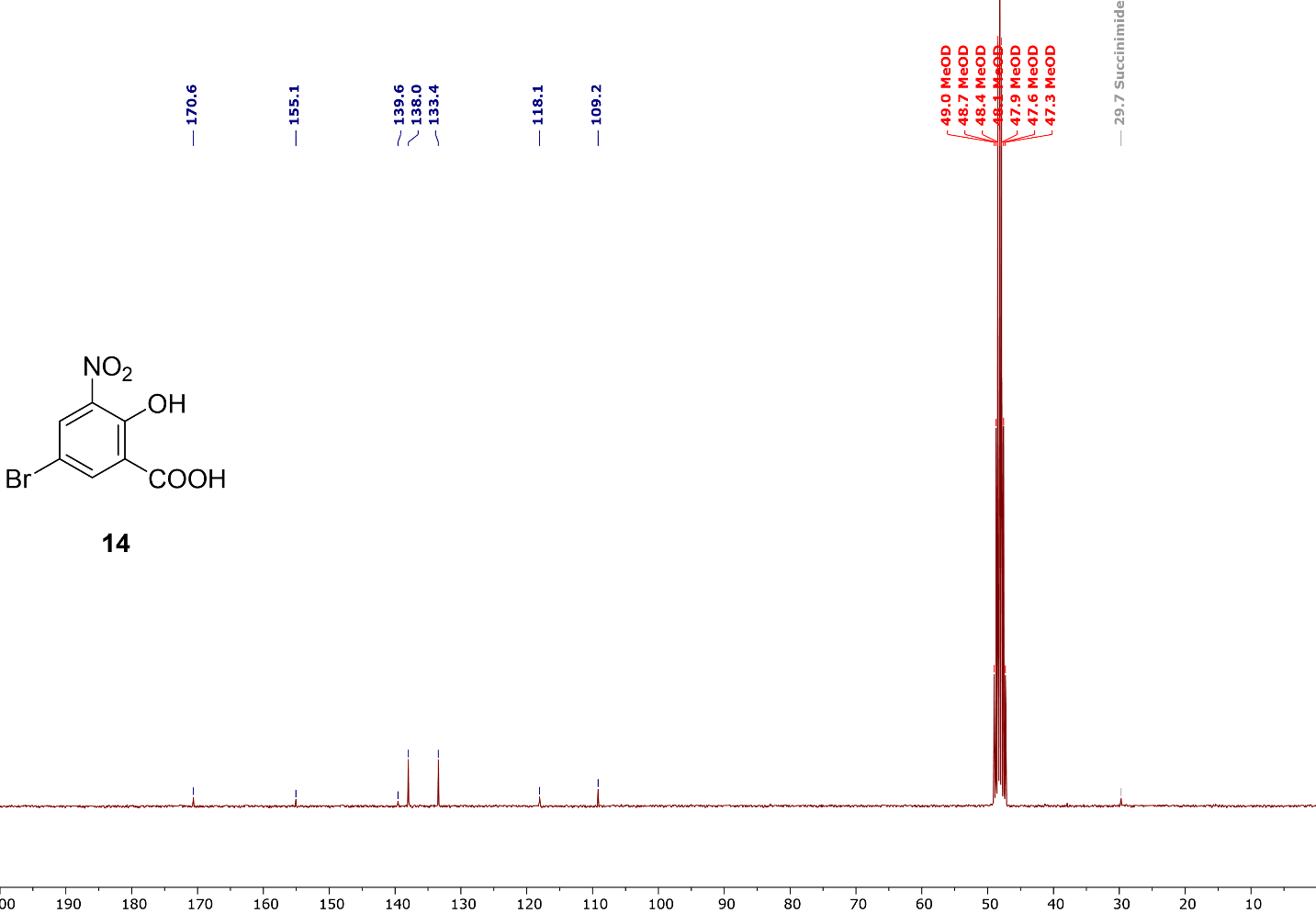


Supporting Figure DS16. ^13^C NMR of compound 14 (75 MHz, 300K, CD_3_OD).


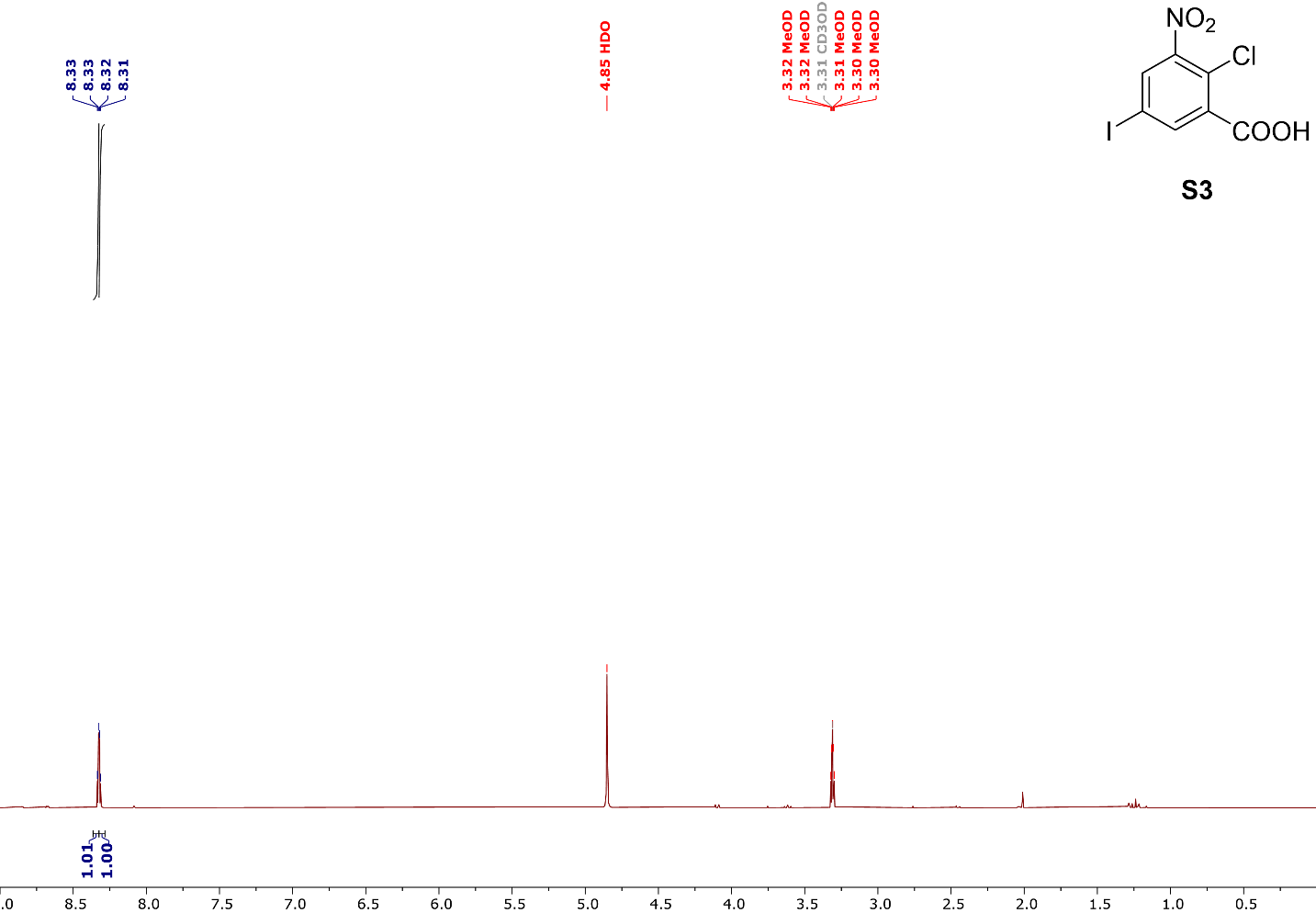


Supporting Figure DS17. ^1^H NMR of compound S3 (300 MHz, 300K, CD_3_OD).


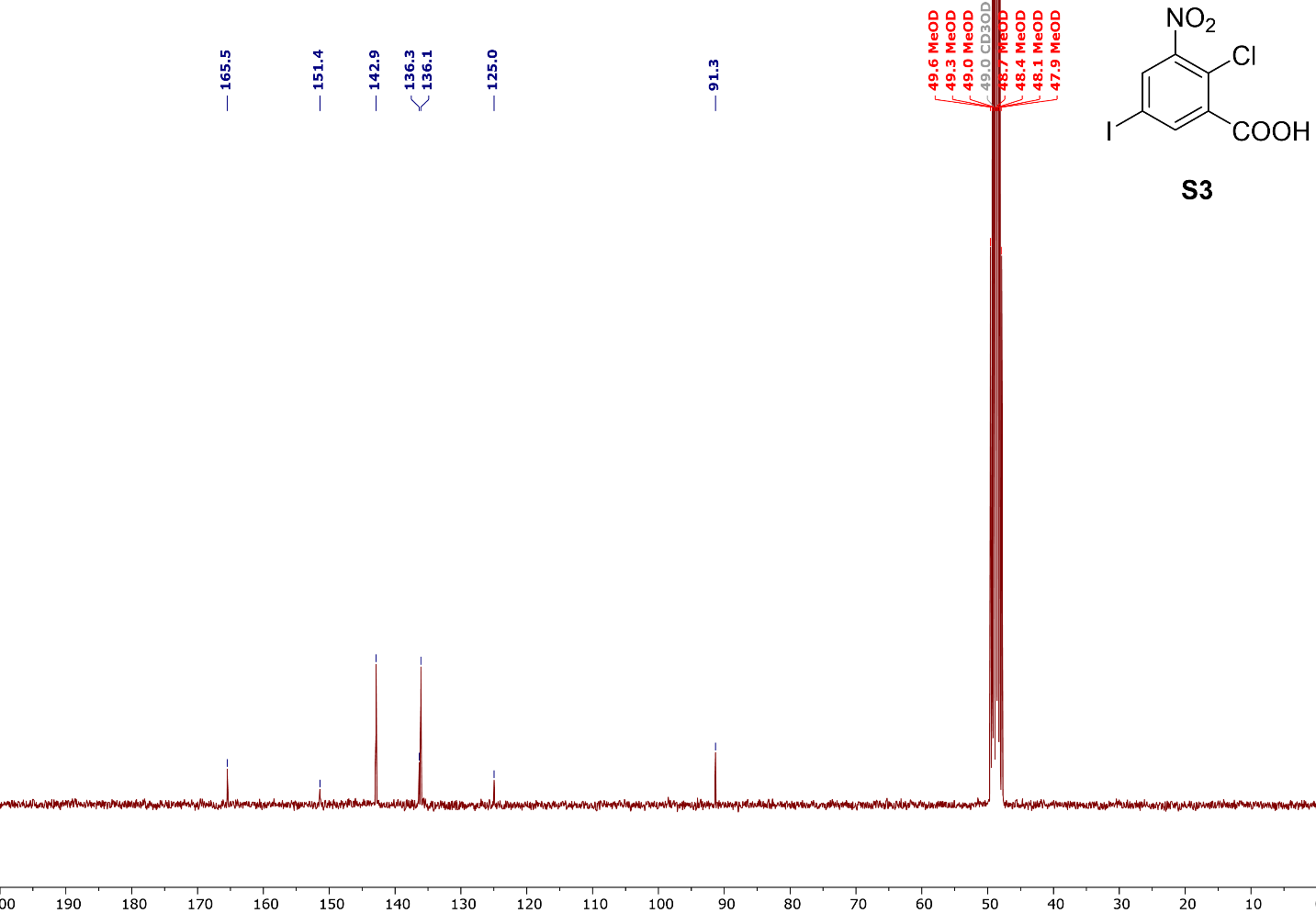


Supporting Figure DS18. ^13^C NMR of compound S3 (75 MHz, 300K, CD_3_OD).


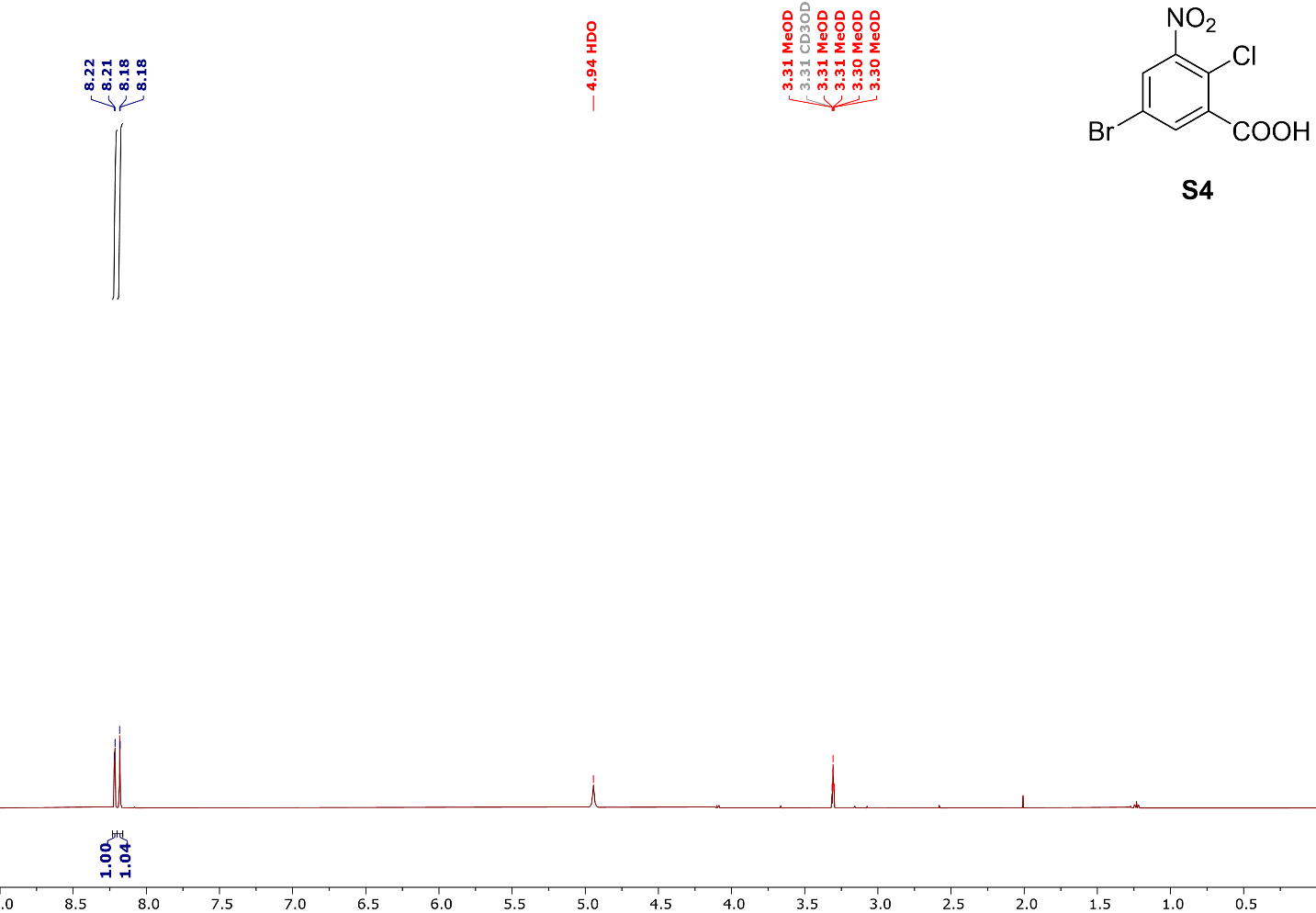


Supporting Figure DS19. ^1^H NMR of compound S4 (500 MHz, 300K, CD_3_OD).


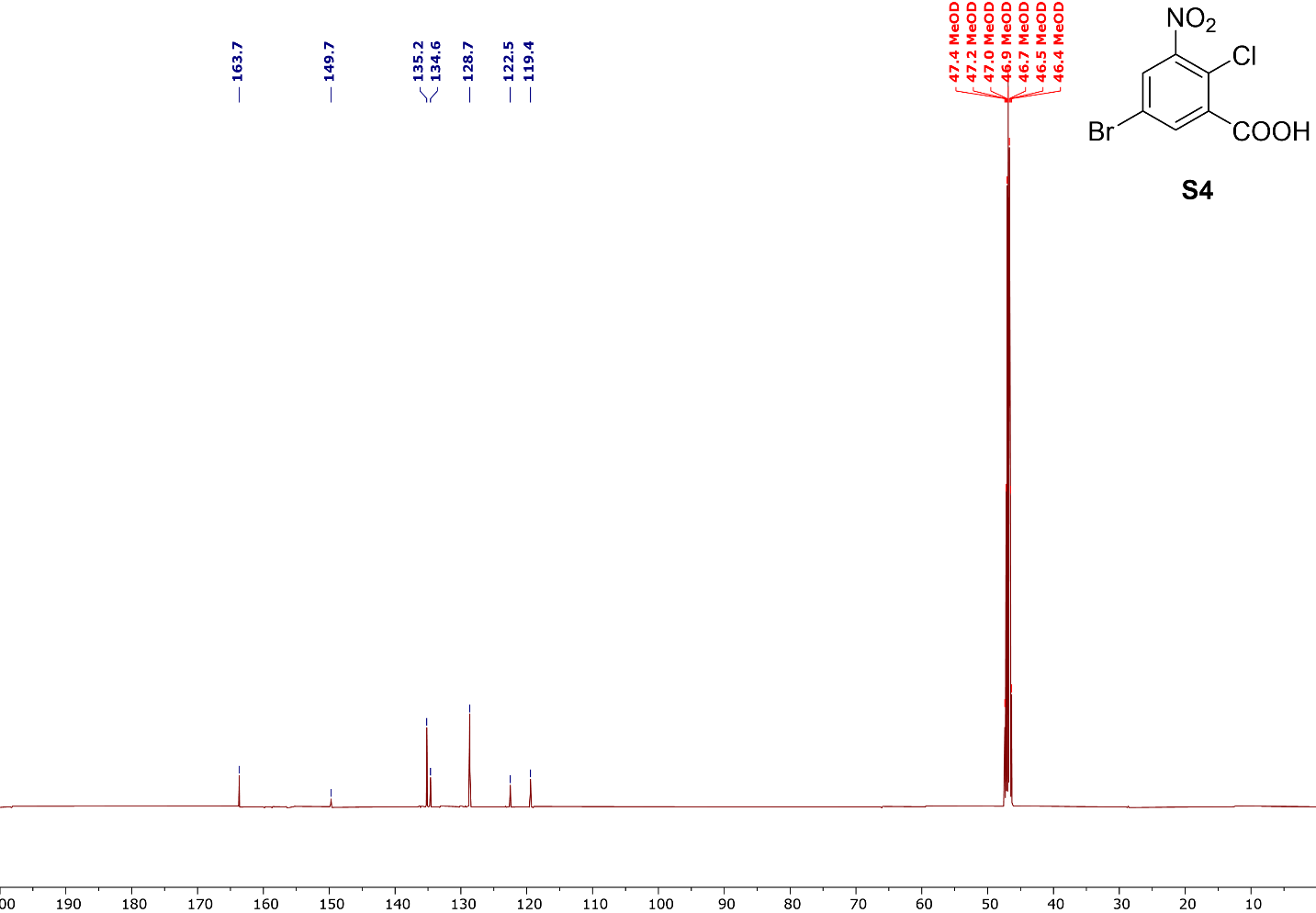


Supporting Figure DS20. ^13^C NMR of compound S4 (126 MHz, 300K, CD_3_OD).


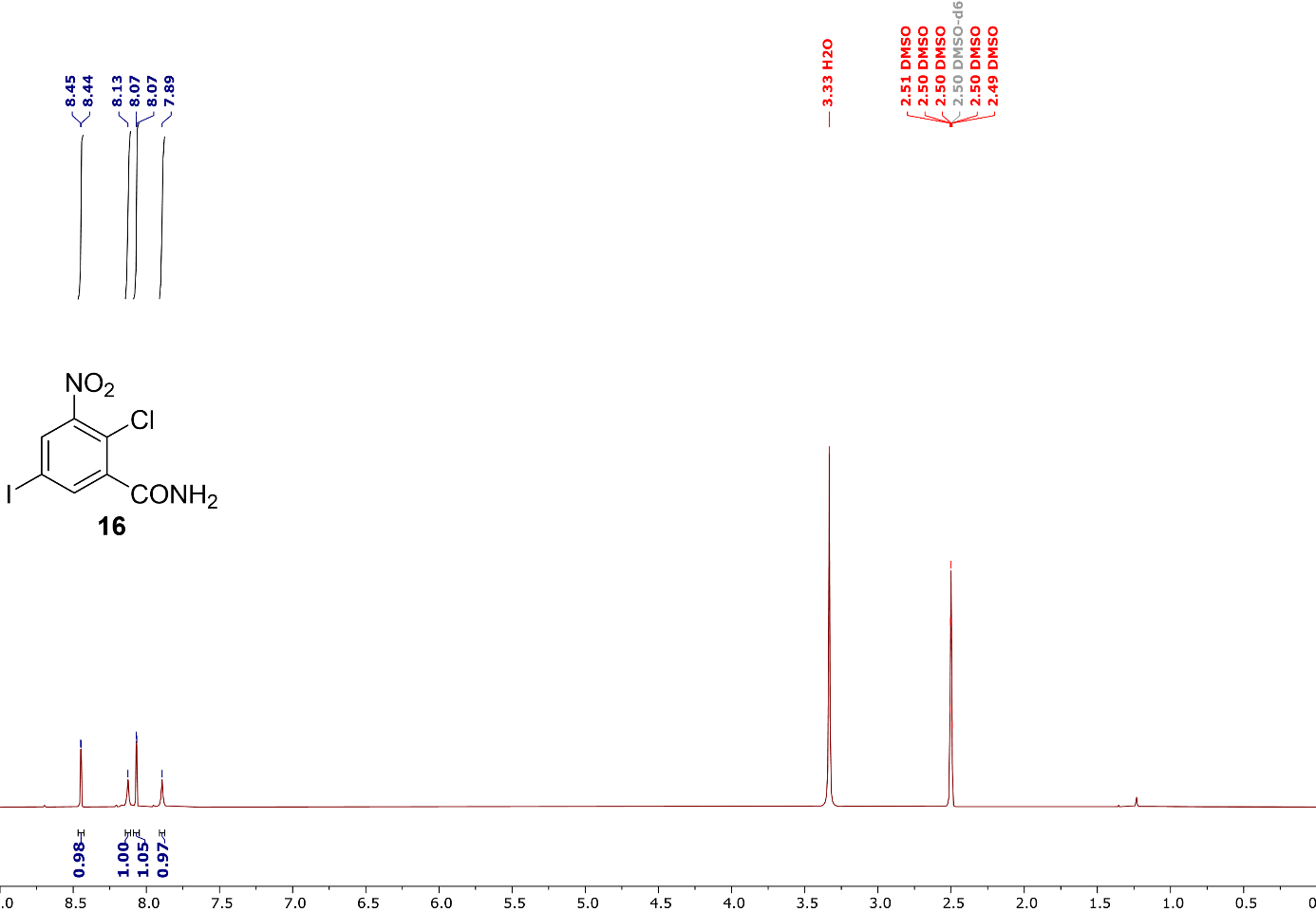


Supporting Figure DS21. ^1^H NMR of compound 16 (500 MHz, 300K, DMSO-*d_6_*).


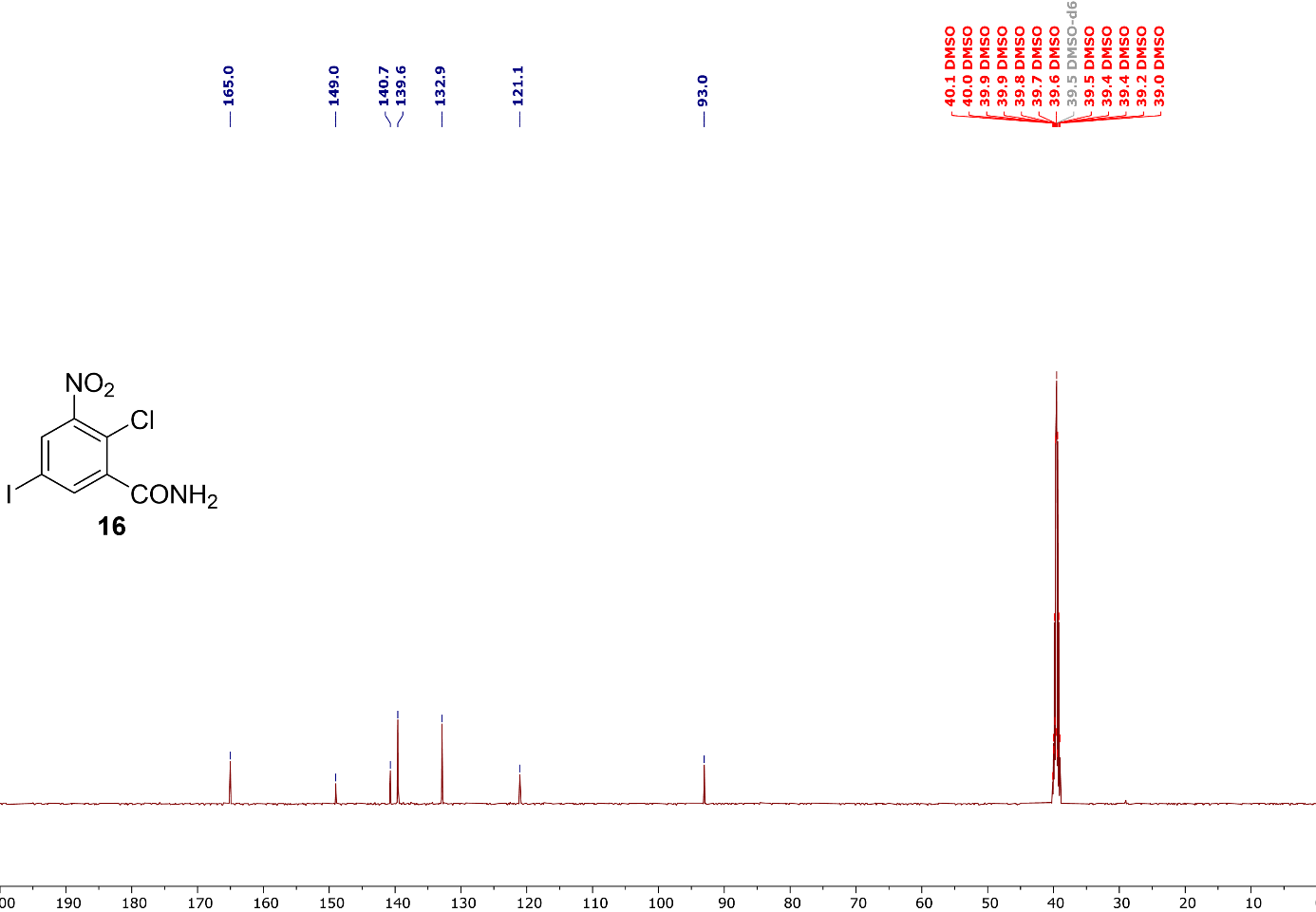


Supporting Figure DS22. ^13^C NMR of compound 16 (126 MHz, 300K, DMSO-*d_6_*).


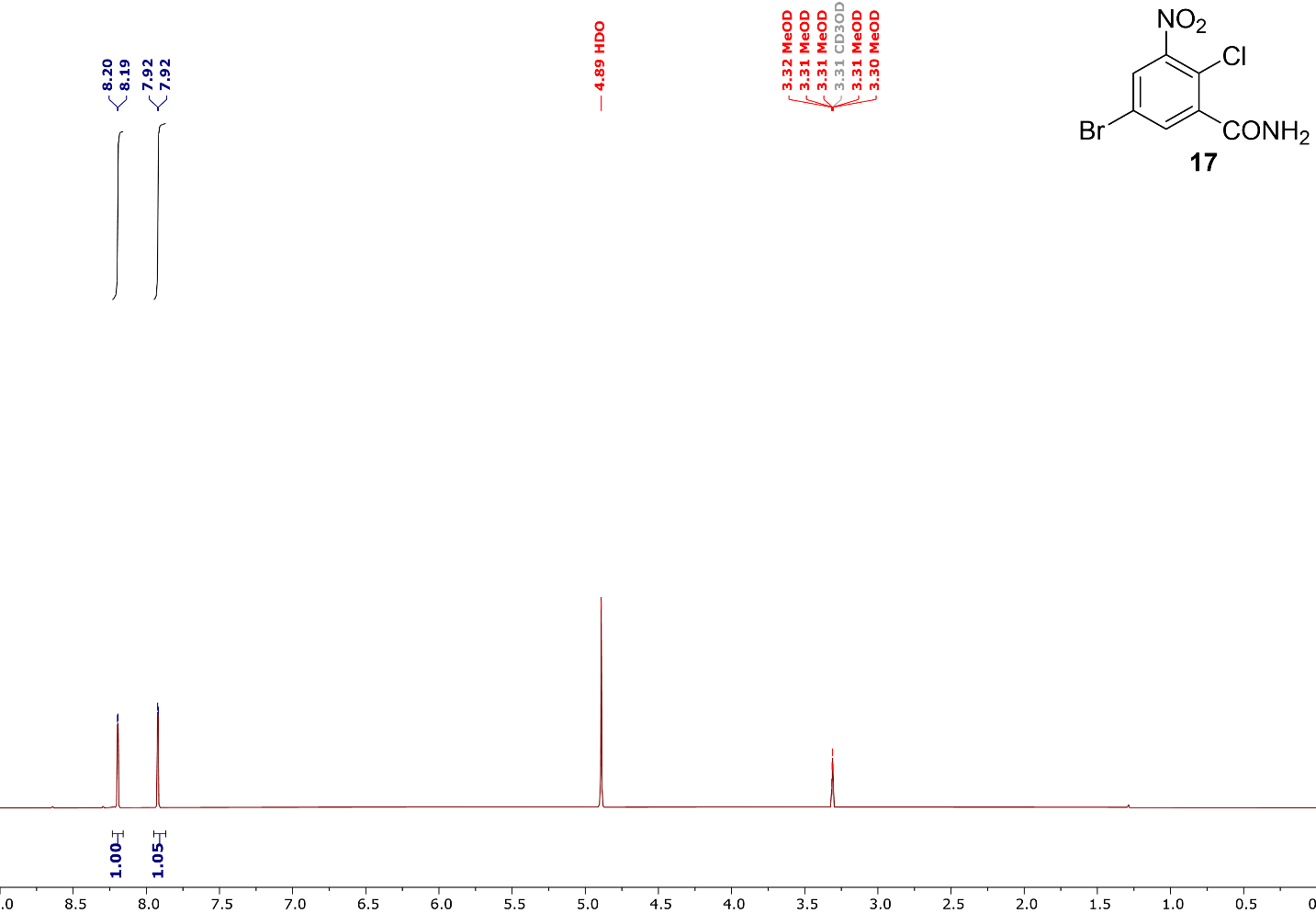


Supporting Figure DS23. ^1^H NMR of compound 17 (500 MHz, 300K, CD_3_OD).


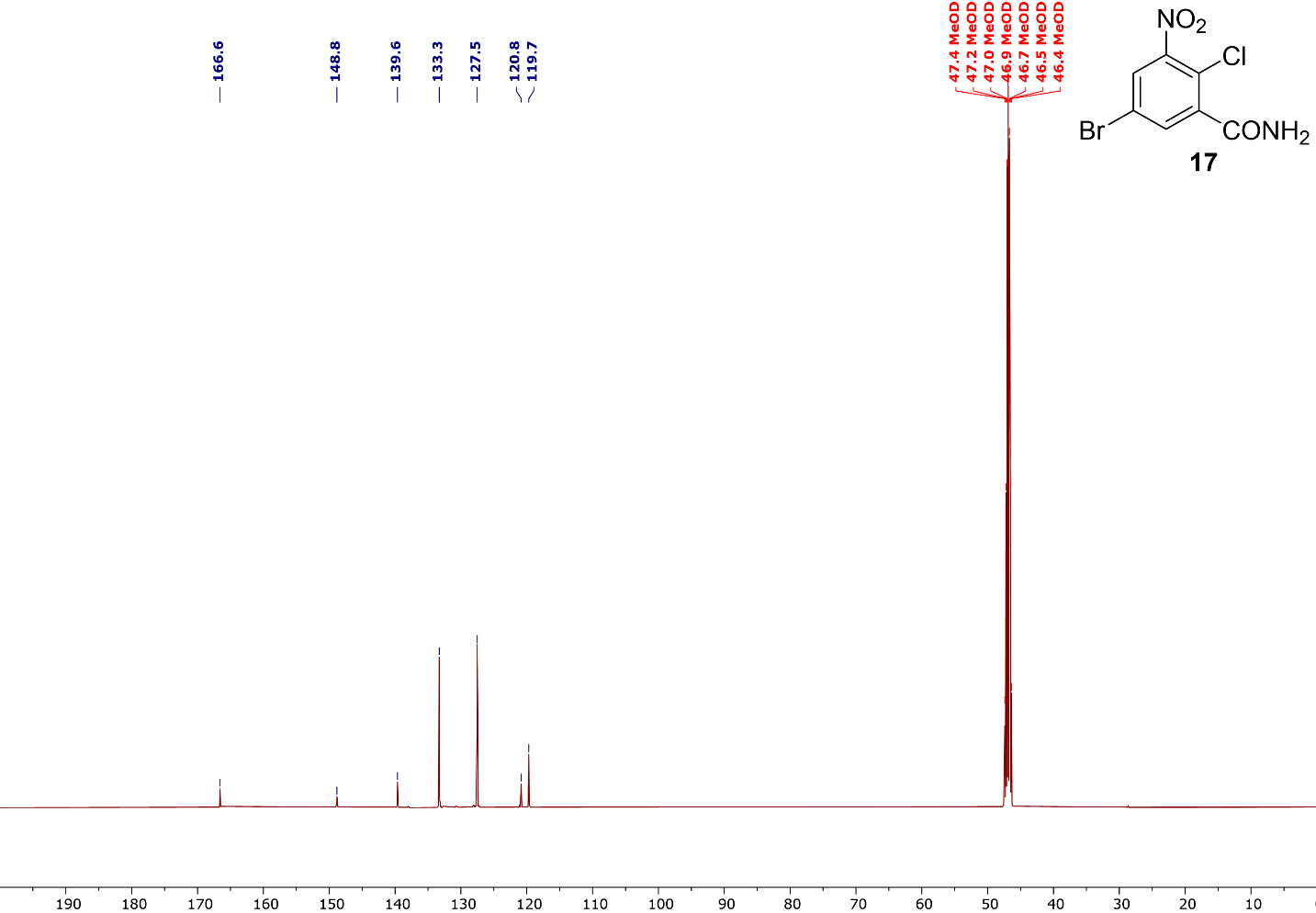


Supporting Figure DS24. ^13^C NMR of compound 17 (126 MHz, 300K, CD_3_OD).


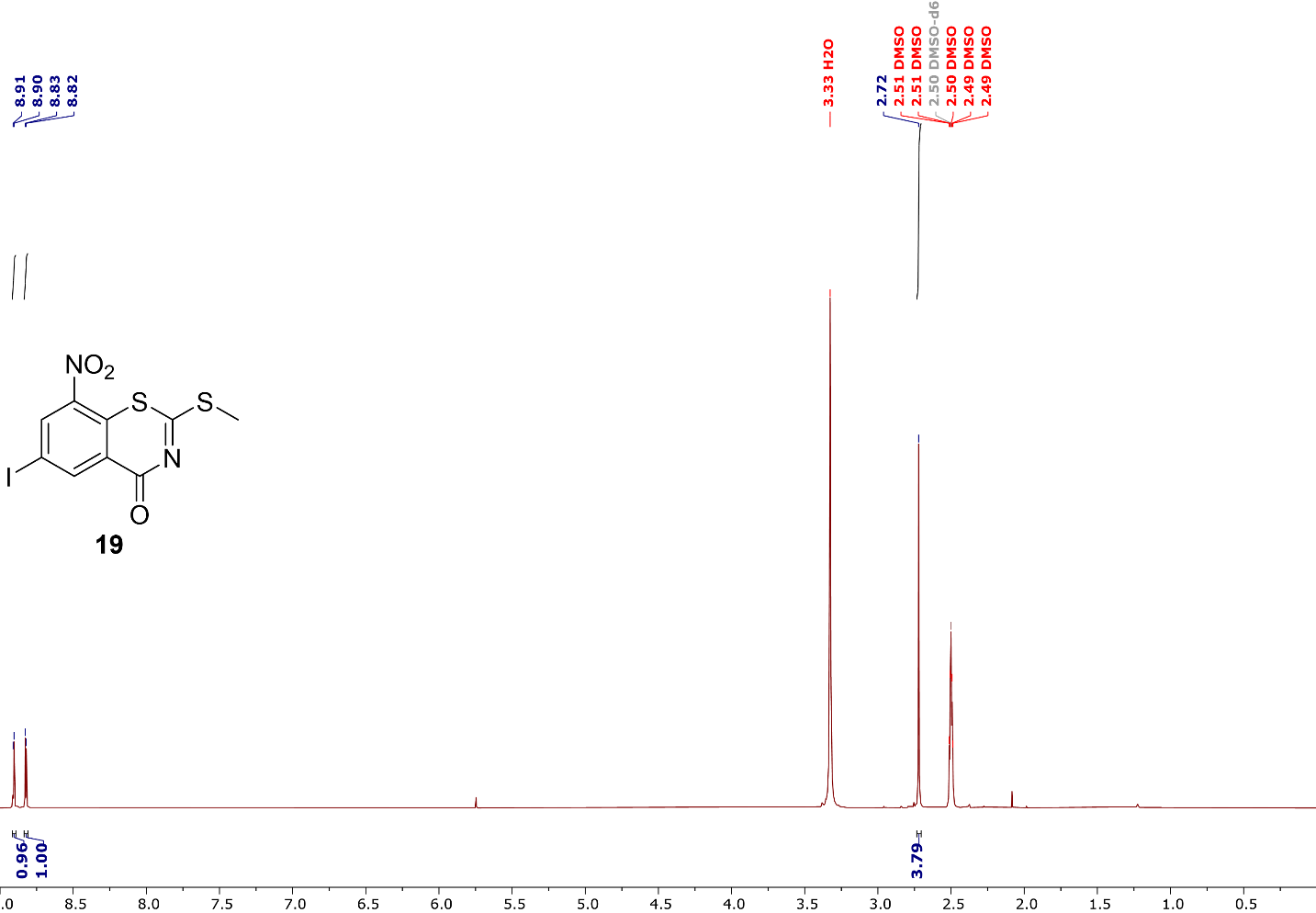


Supporting Figure DS25. ^1^H NMR of compound 19 (300 MHz, 300K, DMSO-*d_6_*).


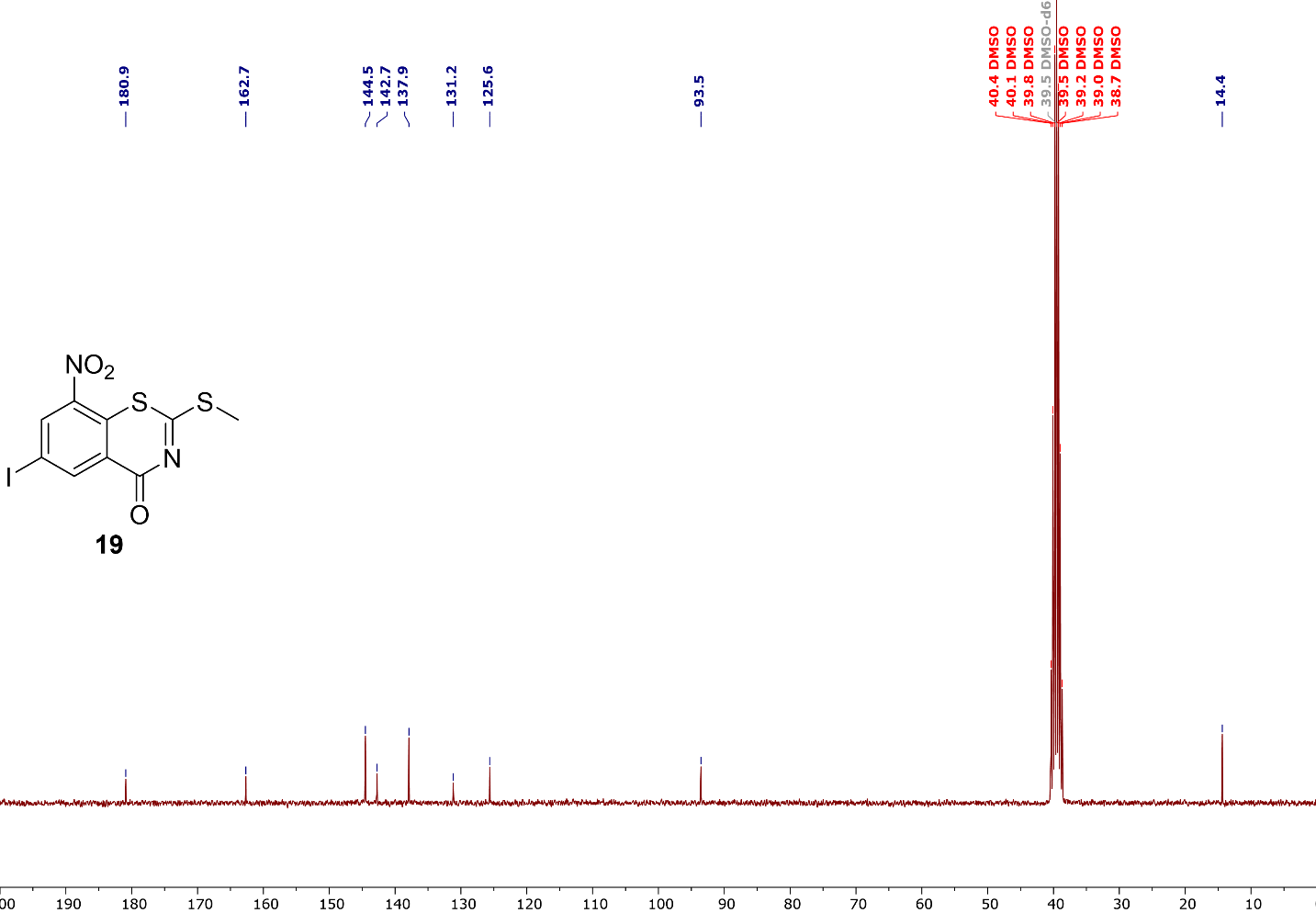


Supporting Figure DS26. ^13^C NMR of compound 19 (75 MHz, 300K, DMSO-*d_6_*).


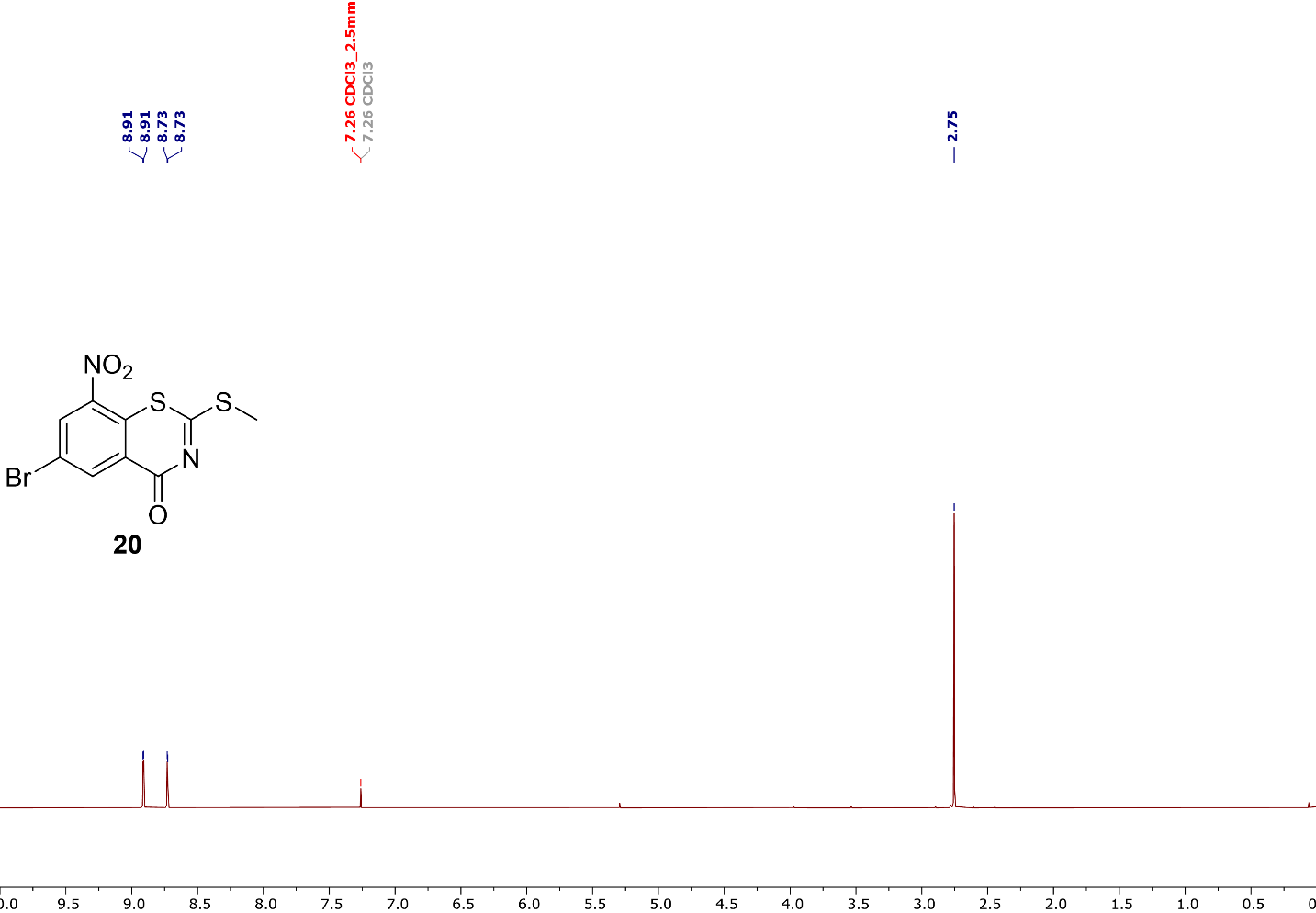


Supporting Figure DS27. ^1^H NMR of compound 20 (500 MHz, 300K, CDCl_3_ + 1% TMS).


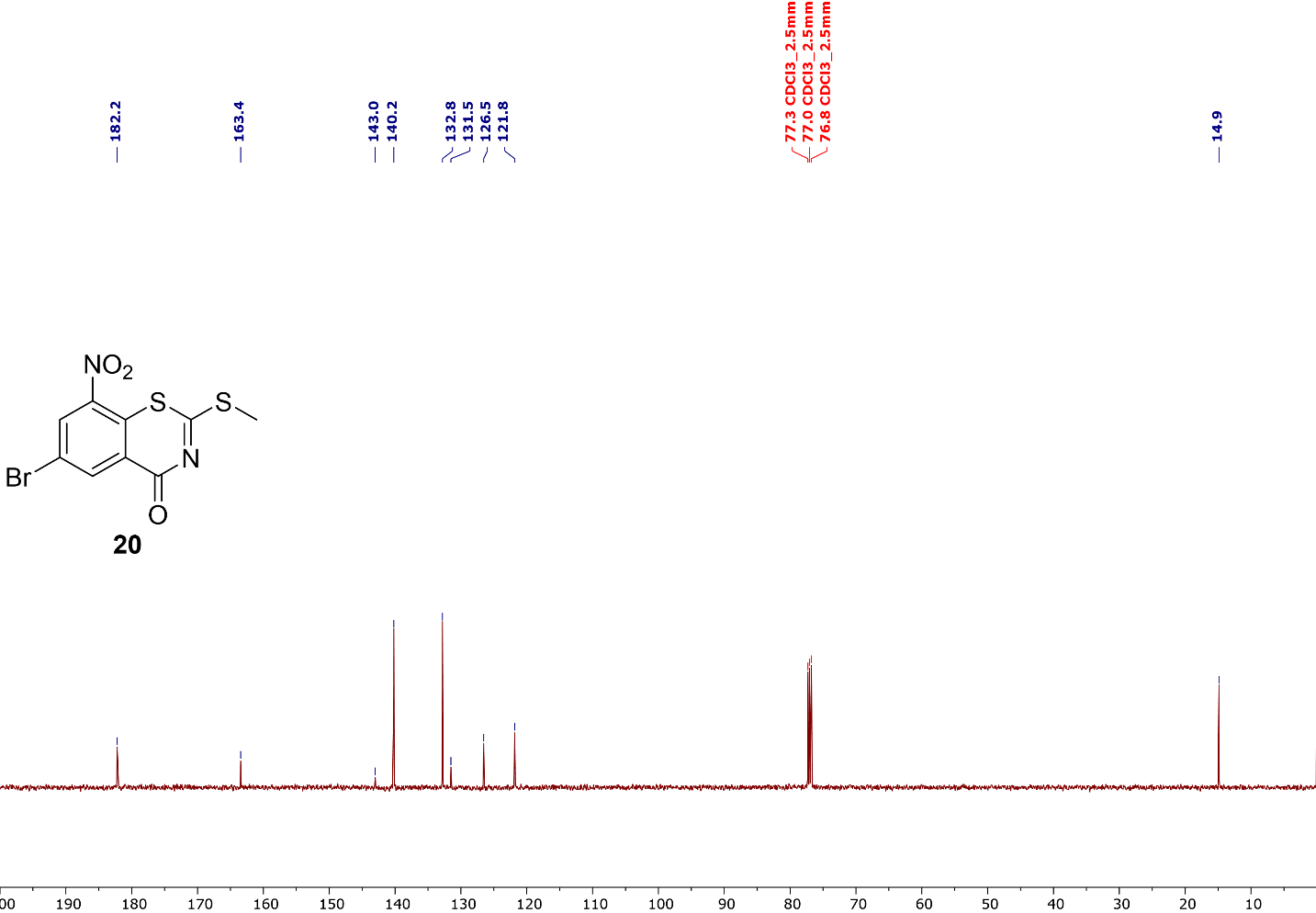


Supporting Figure DS28. ^13^C NMR of compound 20 (126 MHz, 300K, CDCl3 + 1% TMS).


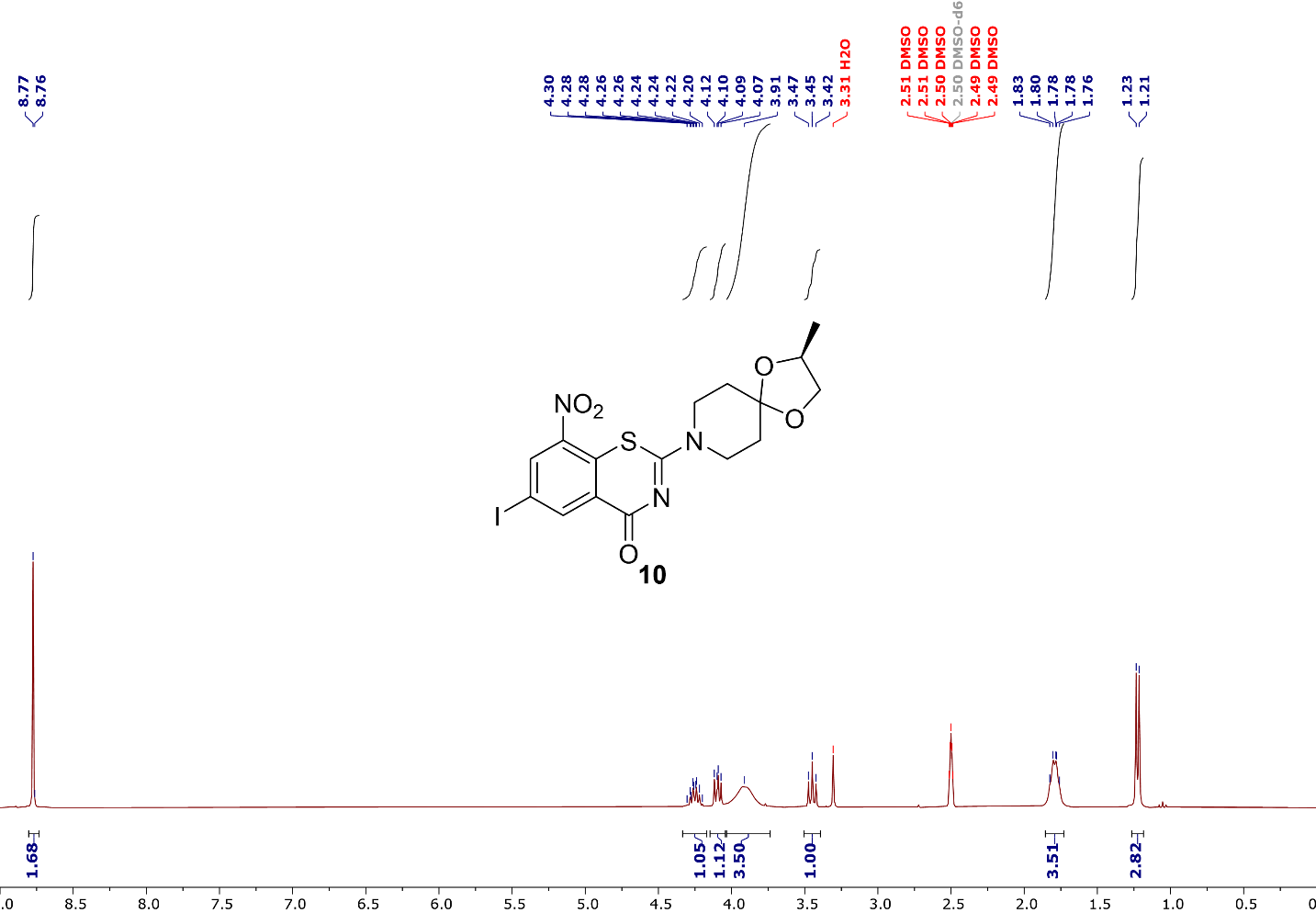


Supporting Figure DS29. ^1^H NMR of compound 10 (300 MHz, 300K, DMSO-*d_6_*).


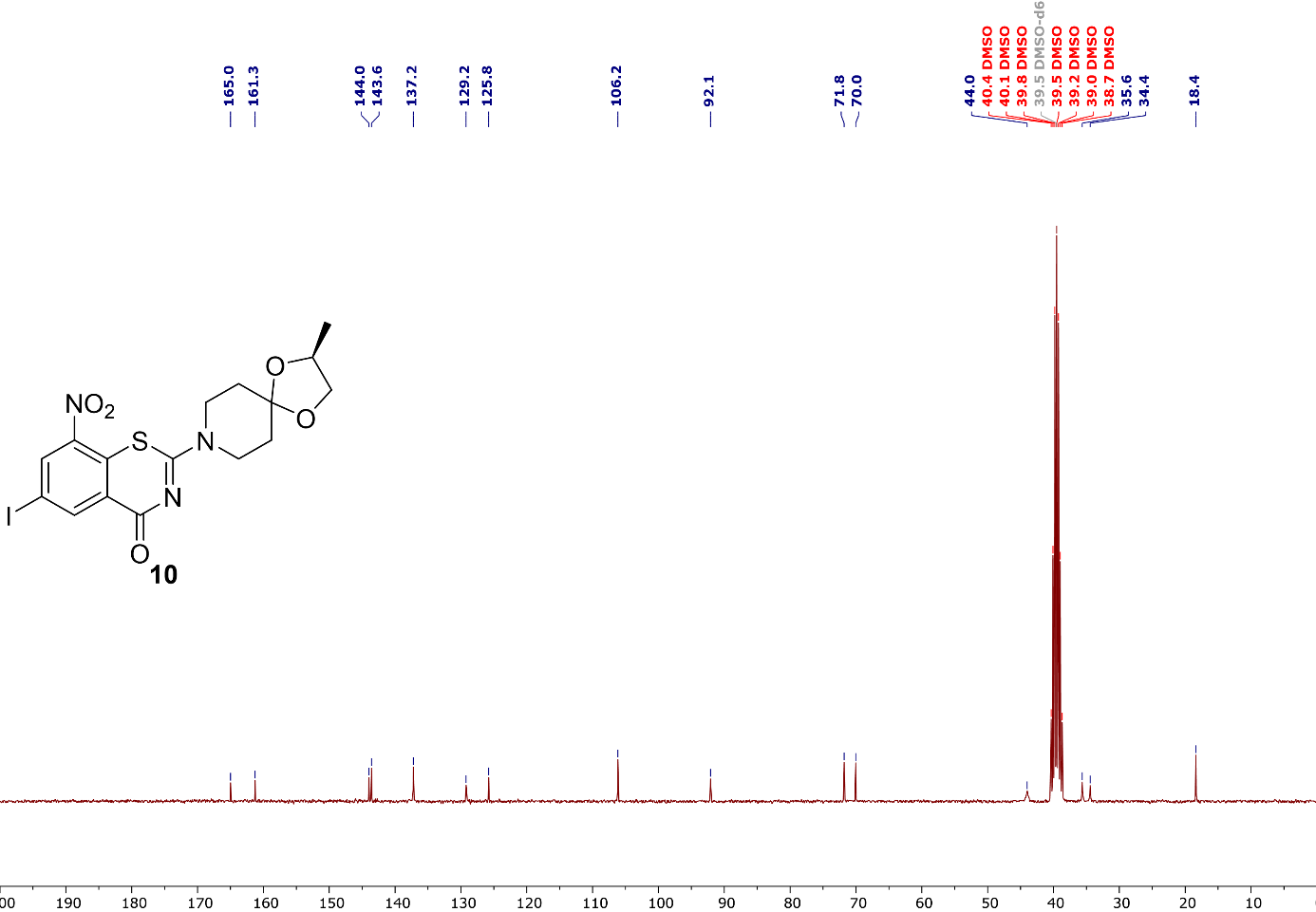


Supporting Figure DS30. ^13^C NMR of compound 10 (75 MHz, 300K, DMSO-*d_6_*).


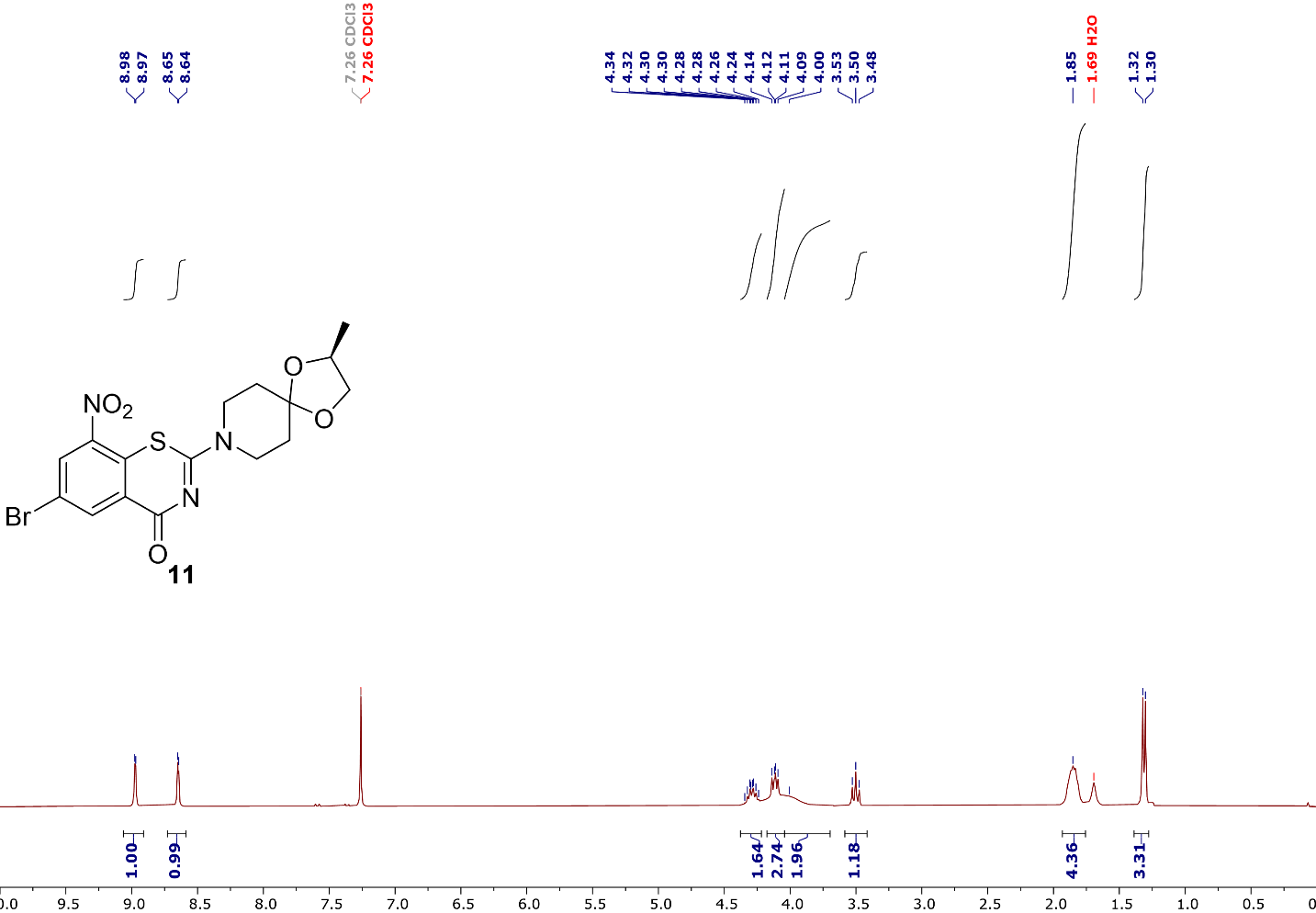


Supporting Figure DS31. ^1^H NMR of compound 11 (300 MHz, 300K, CDCl_3_).


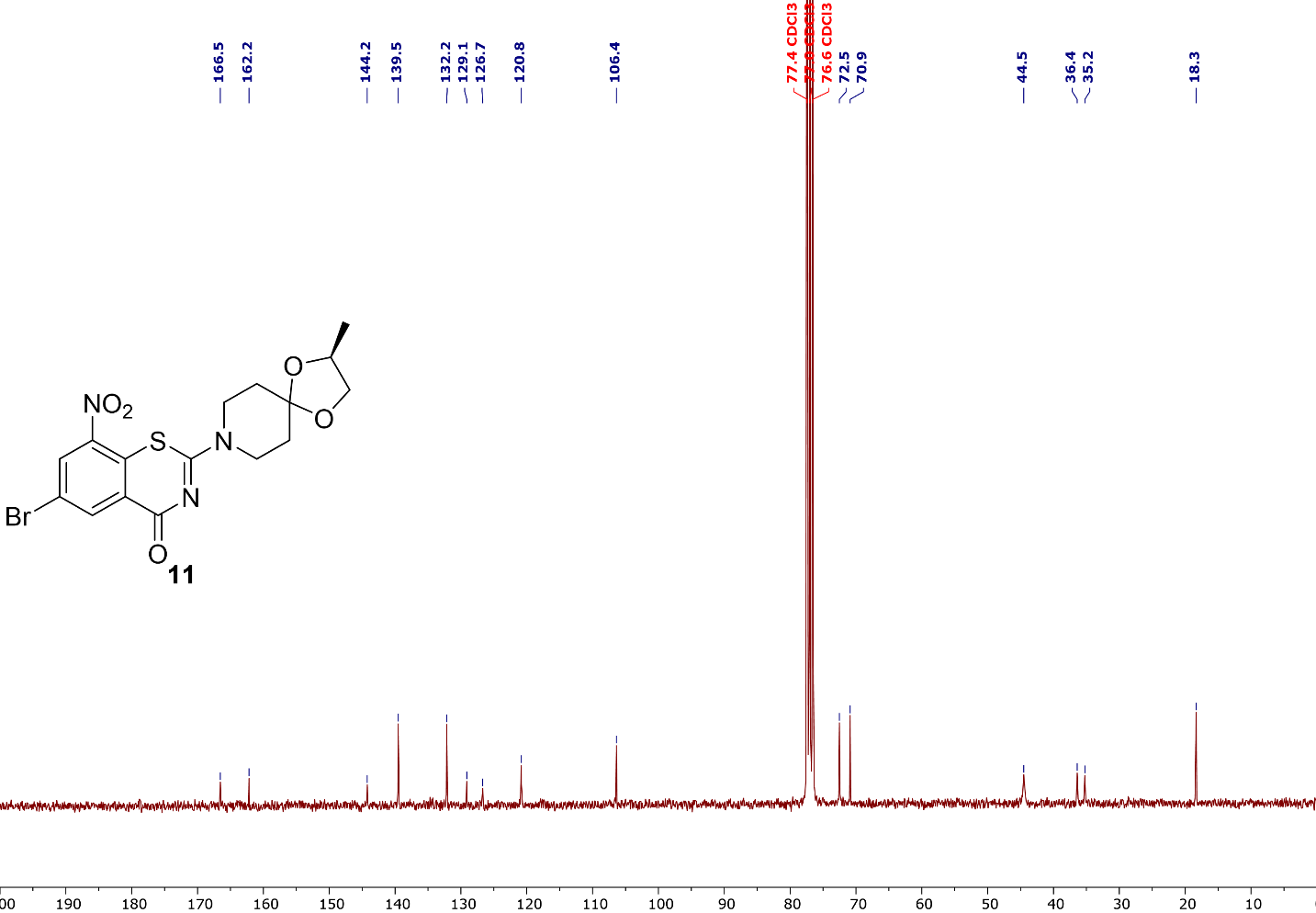


Supporting Figure DS32. ^13^C NMR of compound 11 (75 MHz, 300K, CDCl_3_).


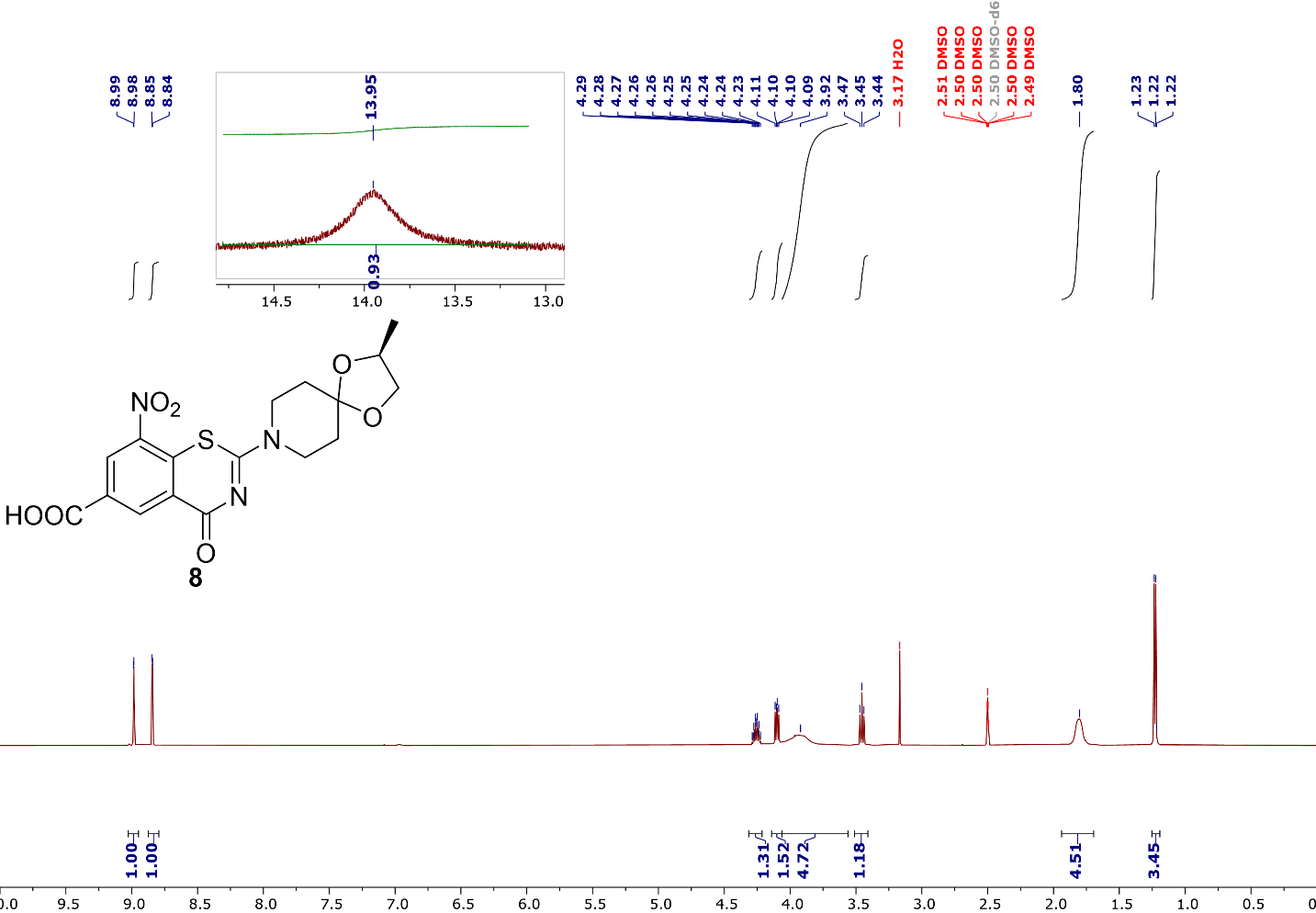


Supporting Figure DS33. ^1^H NMR of compound 8 (500 MHz, 300K, DMSO-*d_6_*).


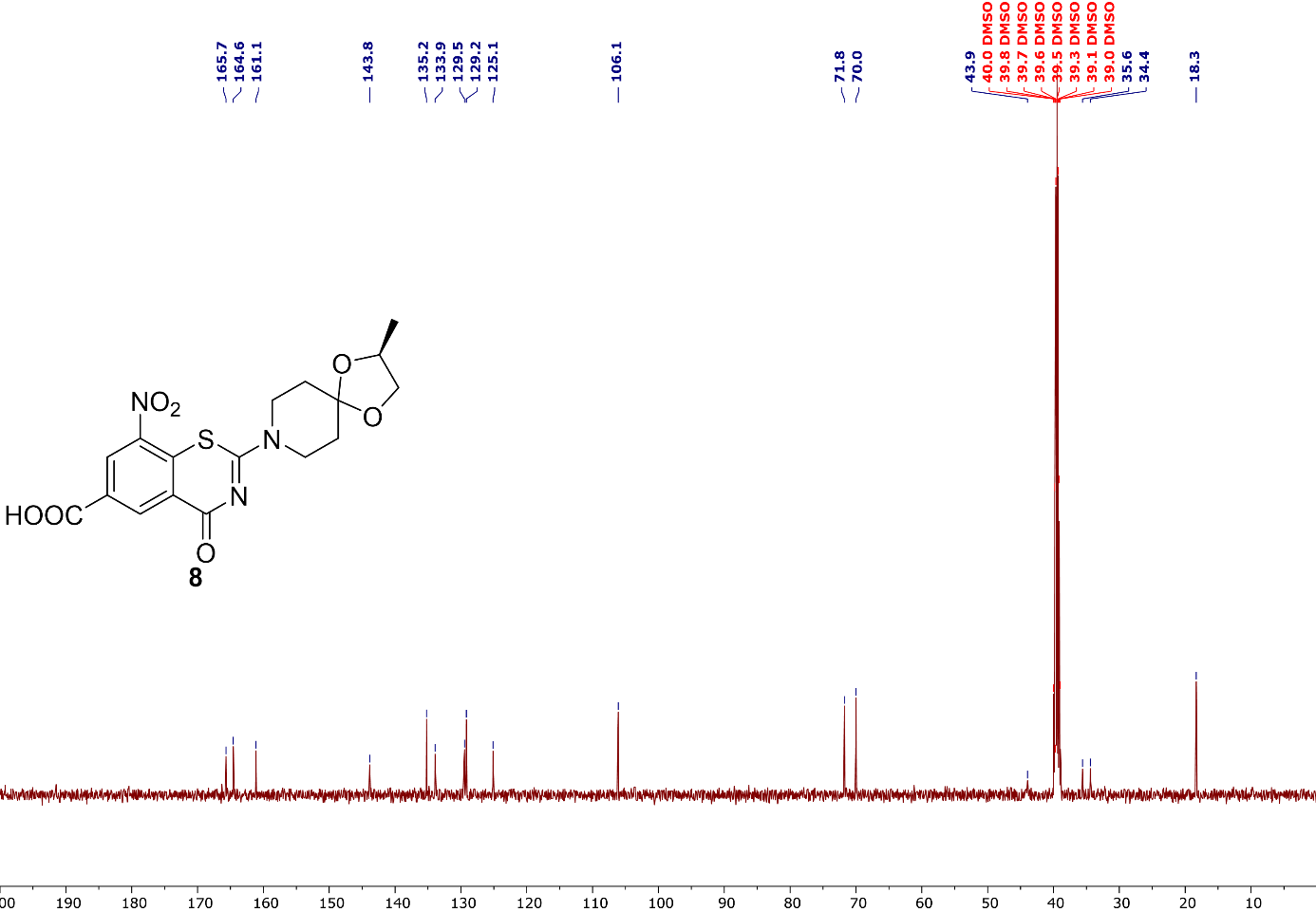


Supporting Figure DS34. ^13^C NMR of compound 8 (126 MHz, 300K, DMSO-*d_6_*).


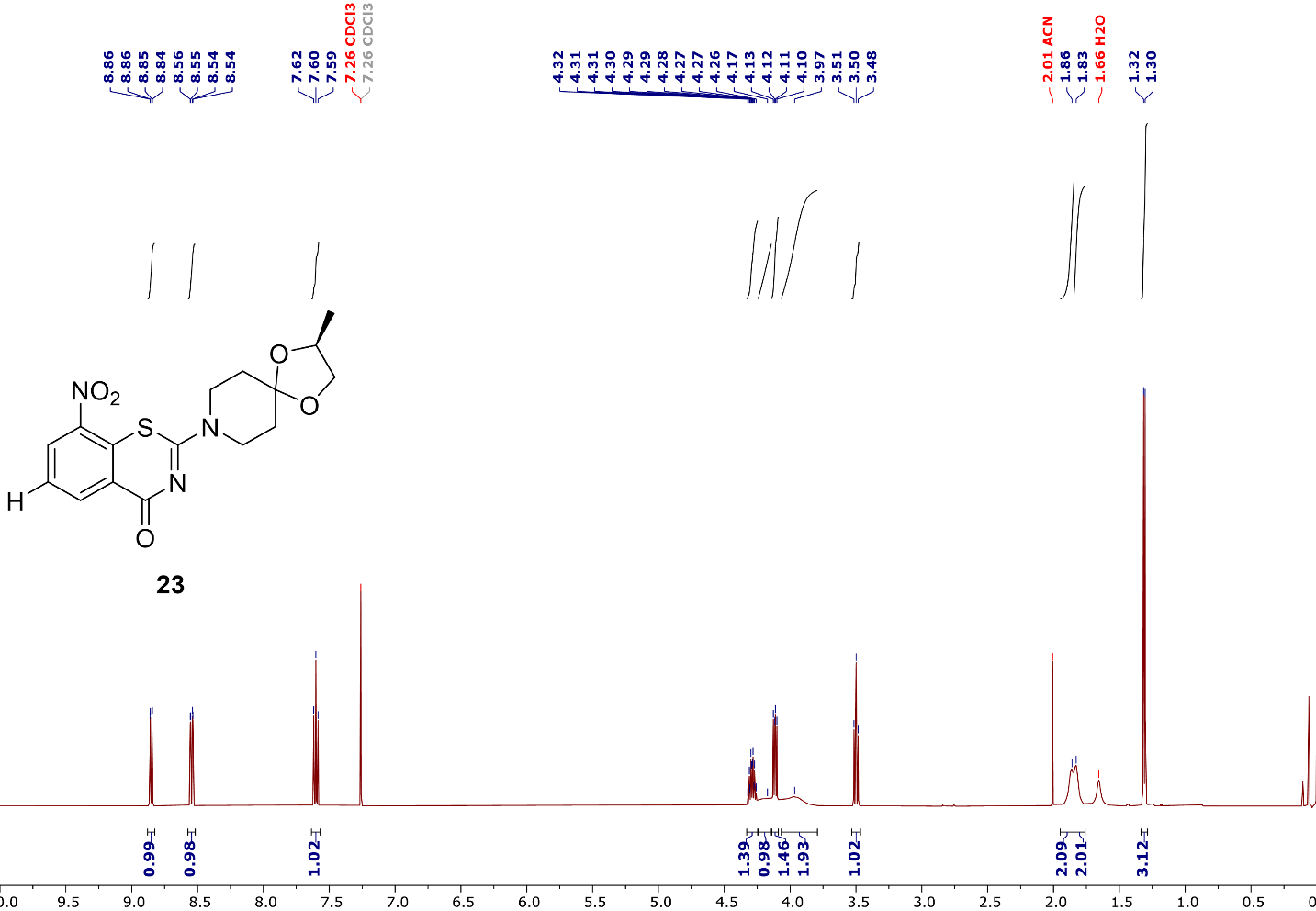


Supporting Figure DS35. ^1^H NMR of compound 24 (500 MHz, 300K, CDCl_3_).


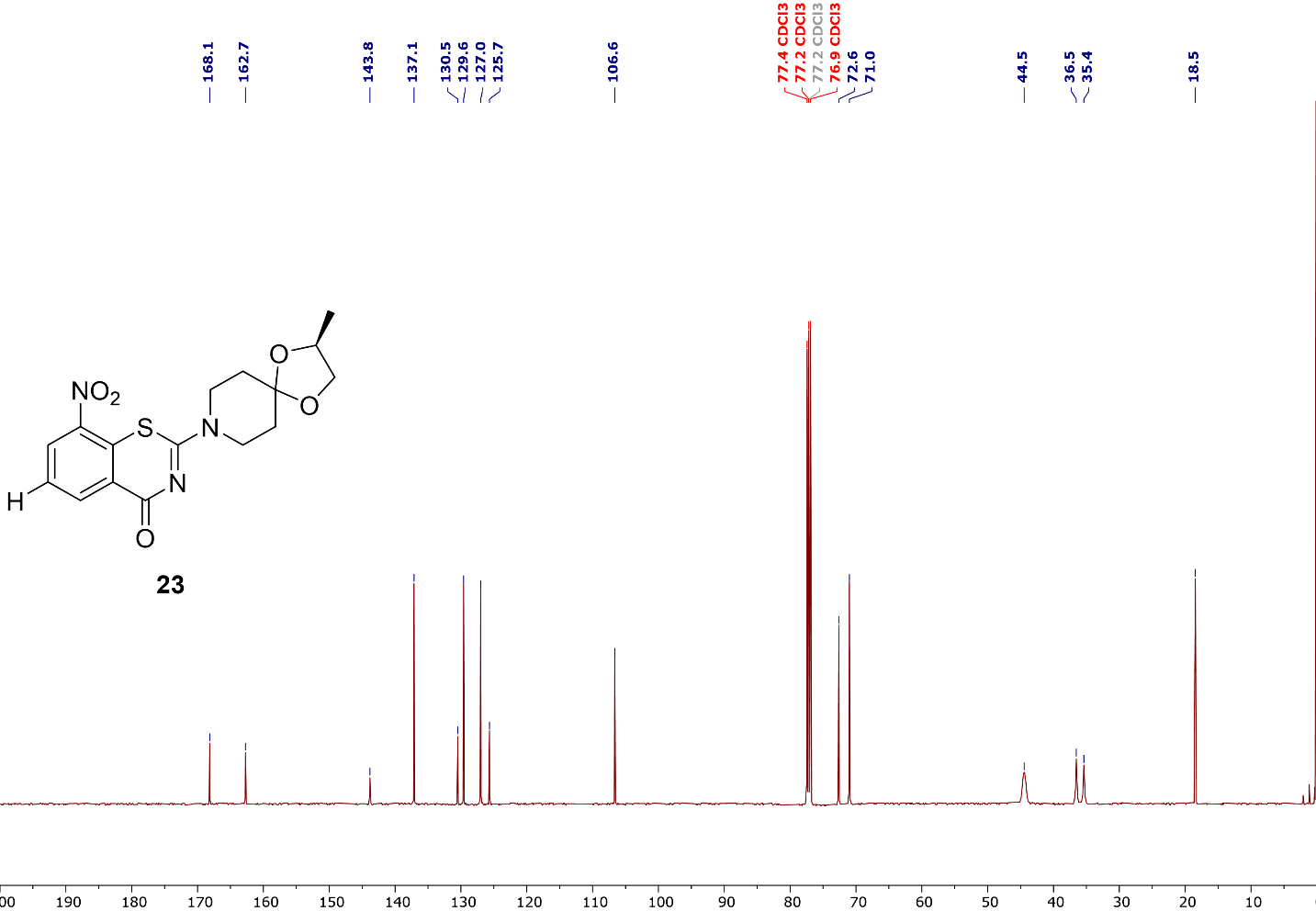


Supporting Figure DS36. ^13^C NMR of compound 24 (126 MHz, 300K, CDCl_3_).


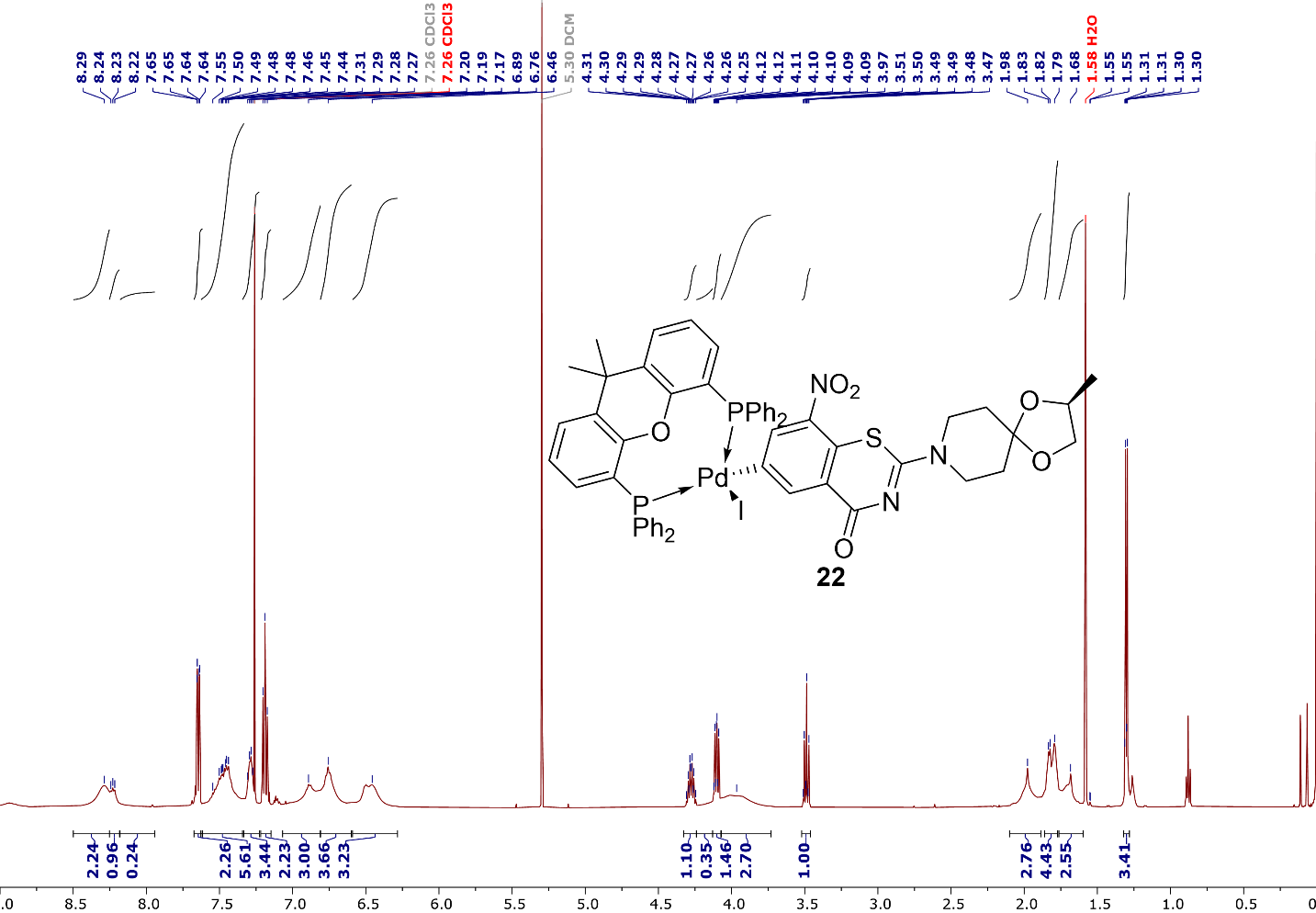


Supporting Figure DS37. ^1^H NMR of compound 22 (500 MHz, 300K, CDCl_3_ + 1% TMS).


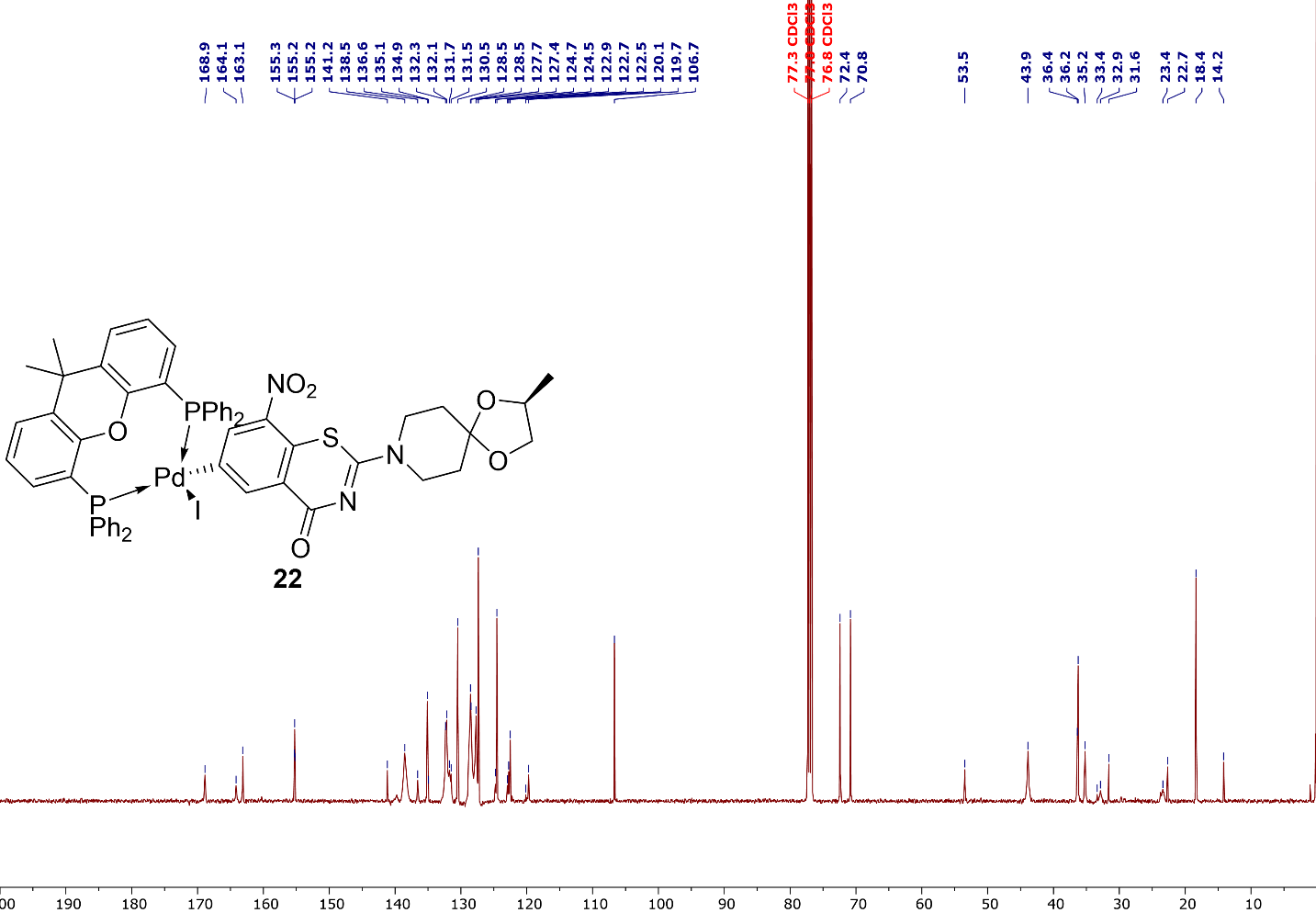


Supporting Figure DS38. ^13^C NMR of compound 22 (126 MHz, 300K, CDCl_3_ + 1% TMS).


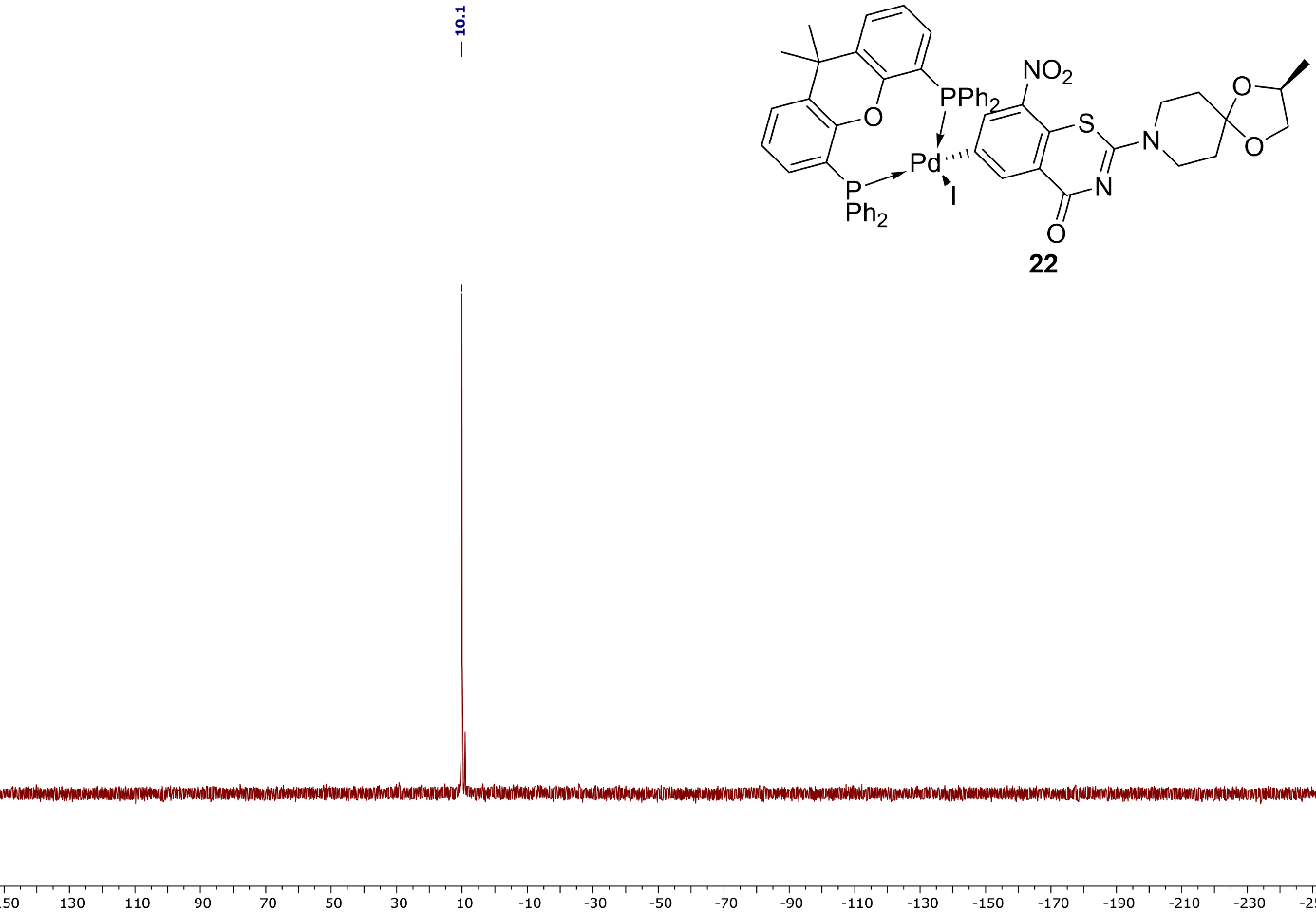


Supporting Figure DS39. ^31^P NMR of compound 22 (202 MHz, 300K, CDCl_3_ + 1% TMS).


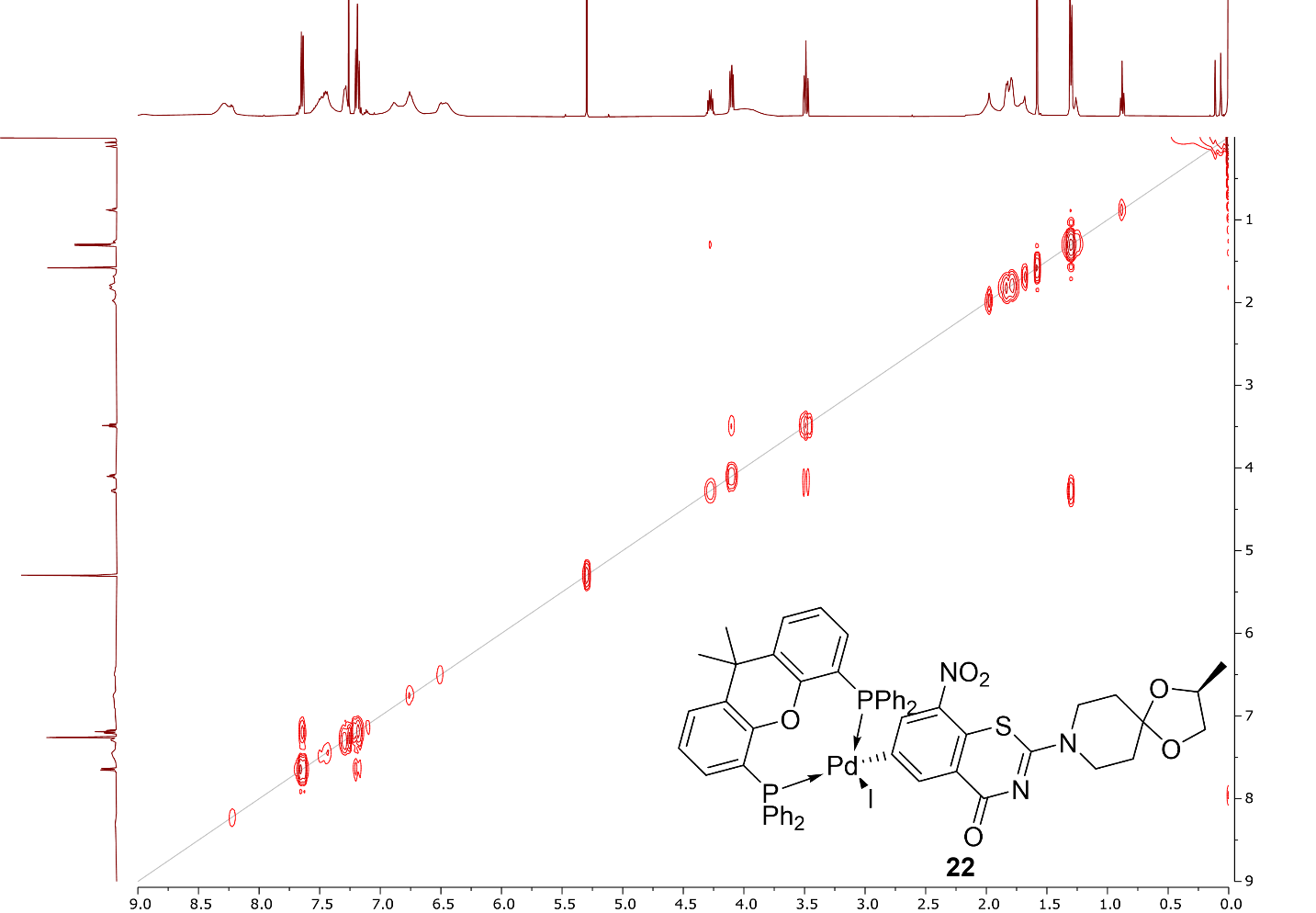


Supporting Figure DS40. COSY experiment of compound 22 (300K, CDCl3 + 1% TMS).


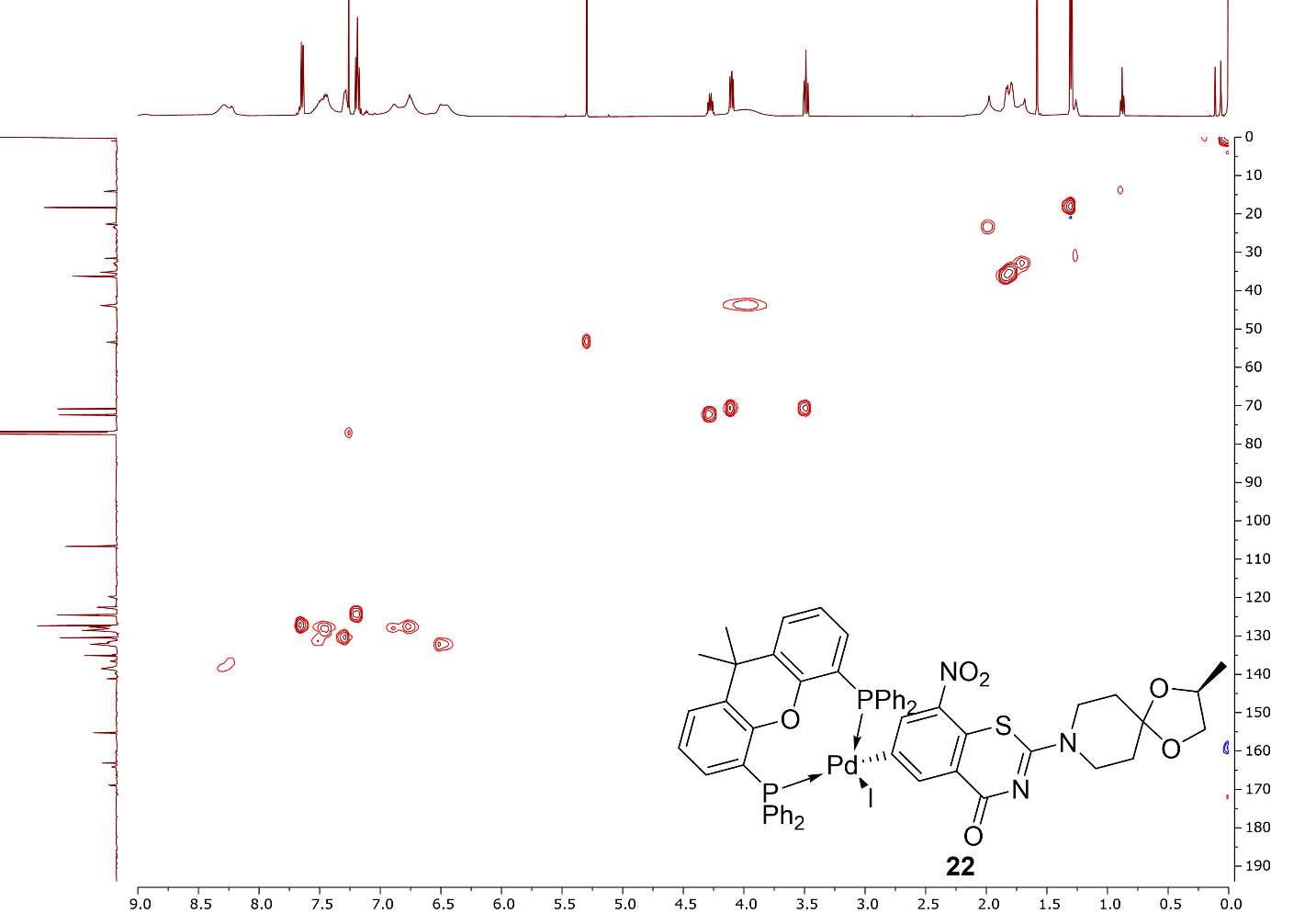


Supporting Figure DS41. HSQC experiment of compound 22 (300K, CDCl_3_ + 1% TMS).


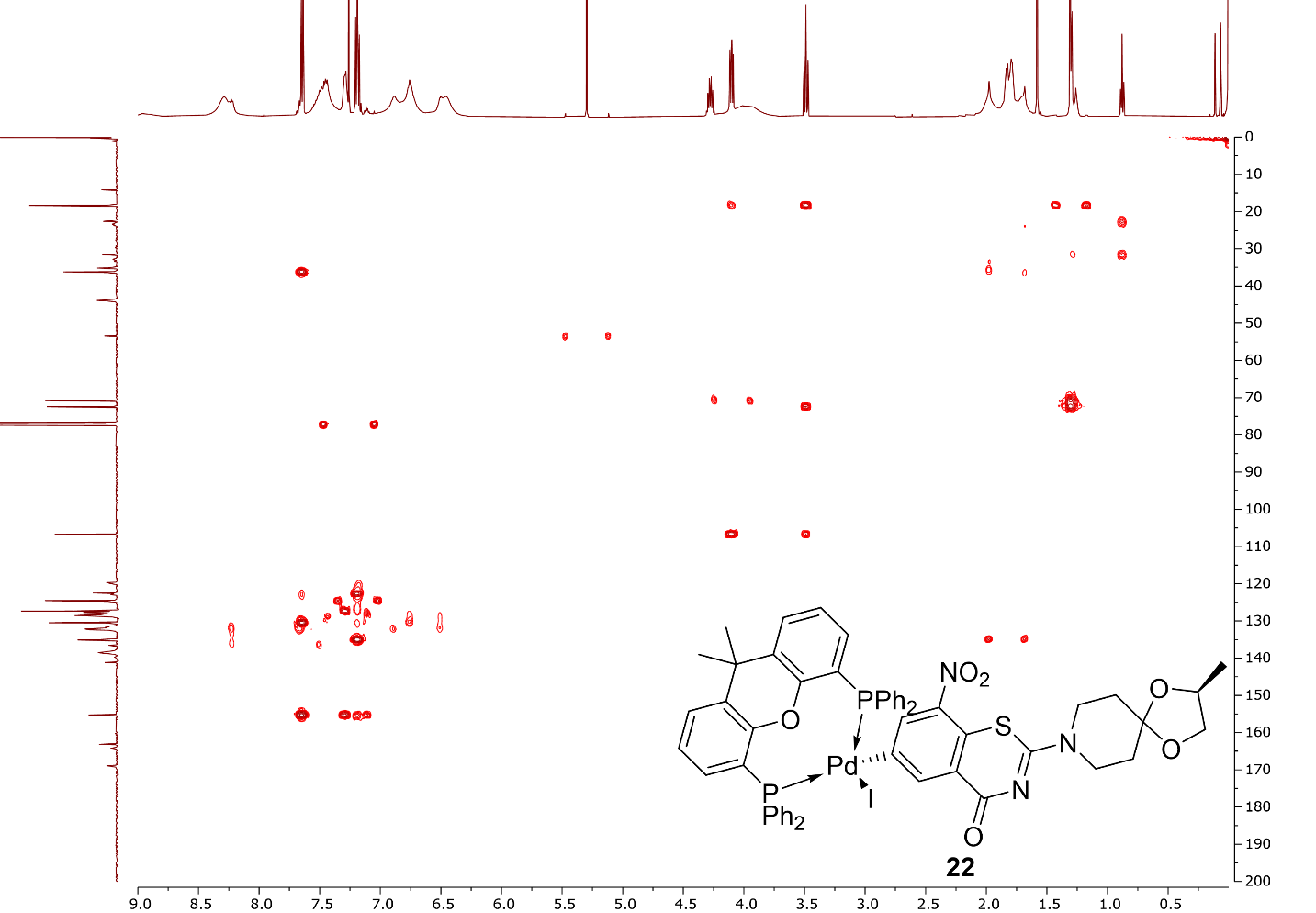


Supporting Figure DS42. HMBC experiment of compound 22 (300K, CDCl_3_ + 1% TMS).


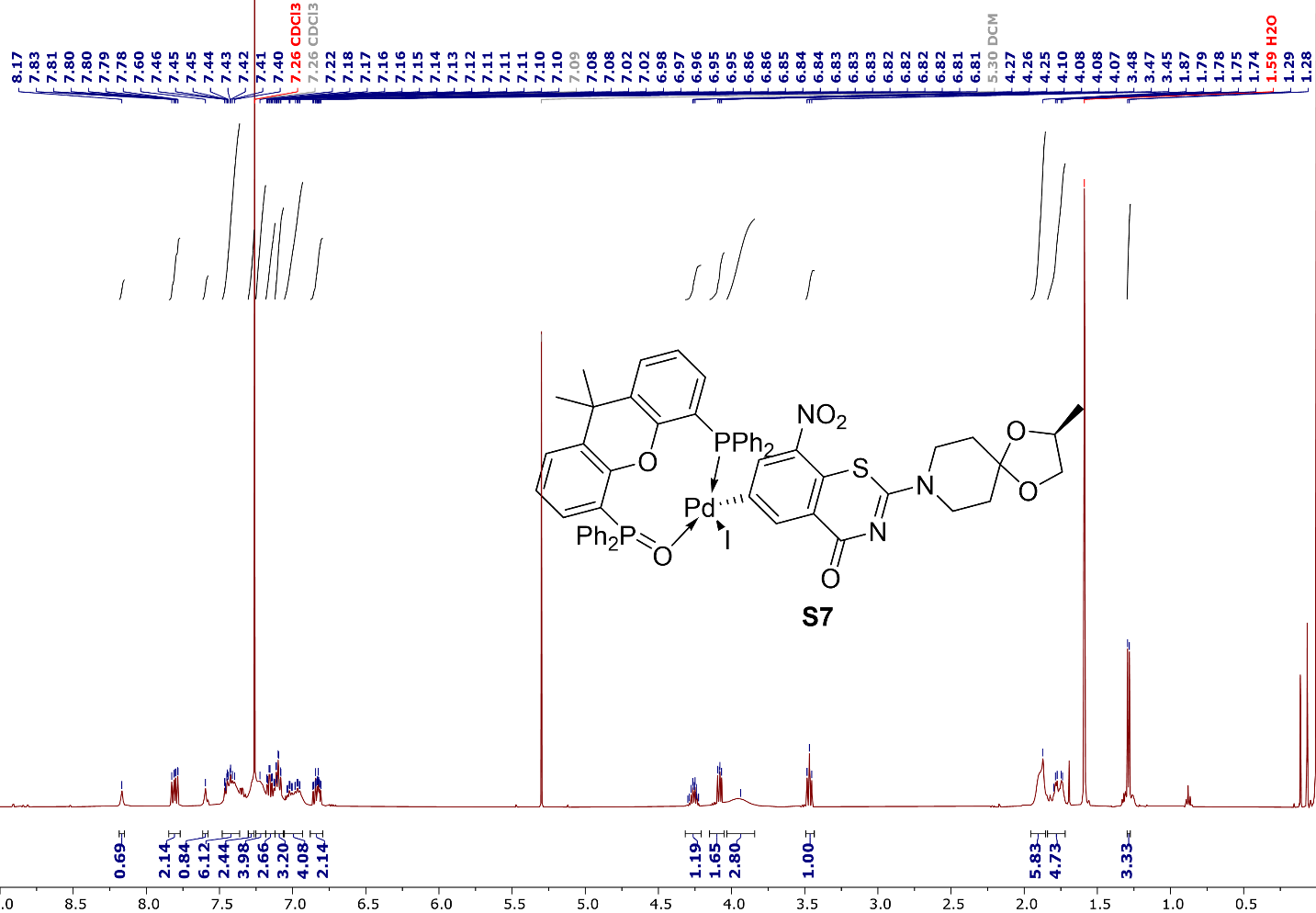


Supporting Figure DS43. ^1^H NMR of compound S7 (500 MHz, 300K, CDCl_3_ + 1% TMS).


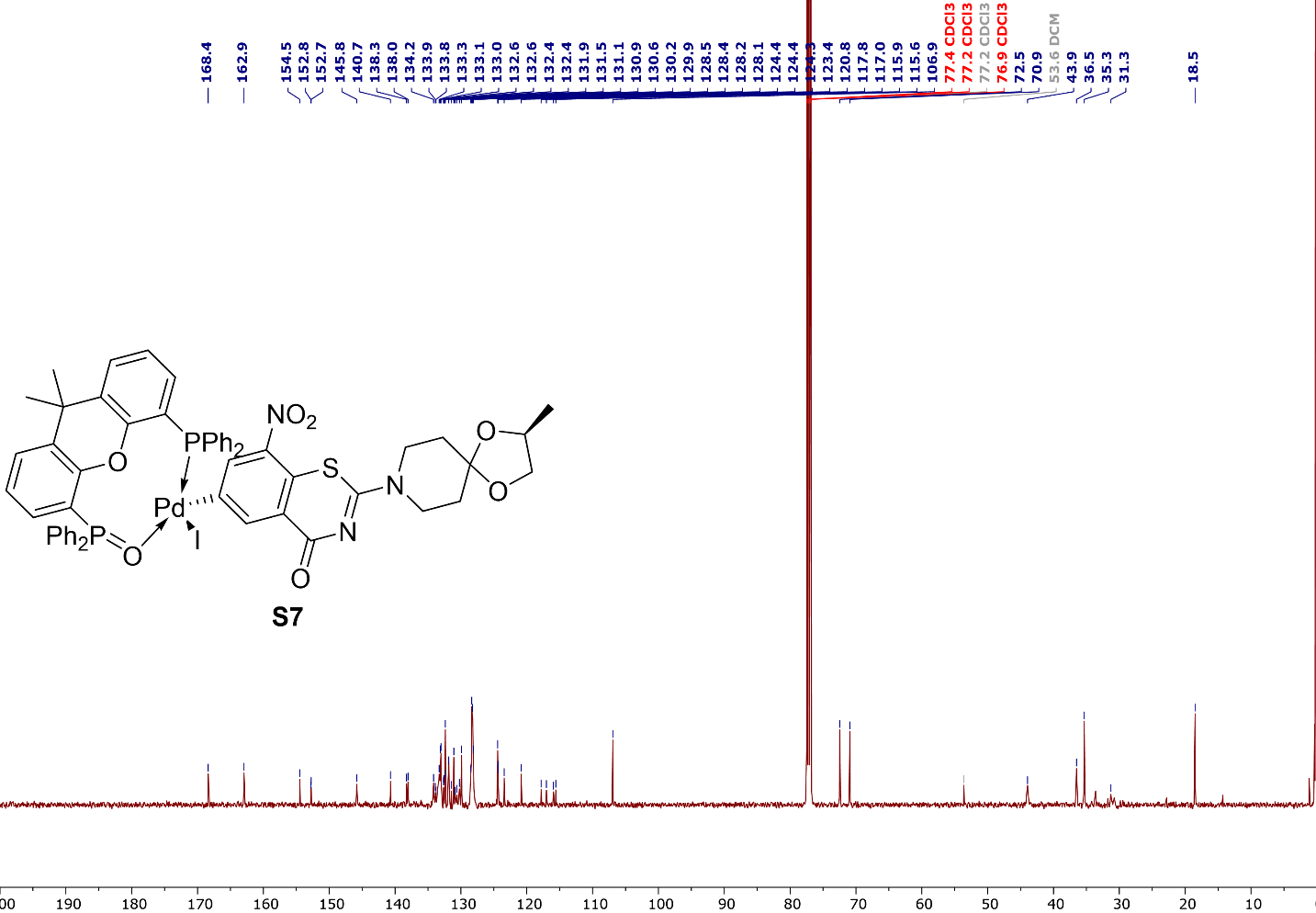


Supporting Figure DS44. ^13^C NMR of compound S7 (126 MHz, 300K, CDCl_3_ + 1% TMS).


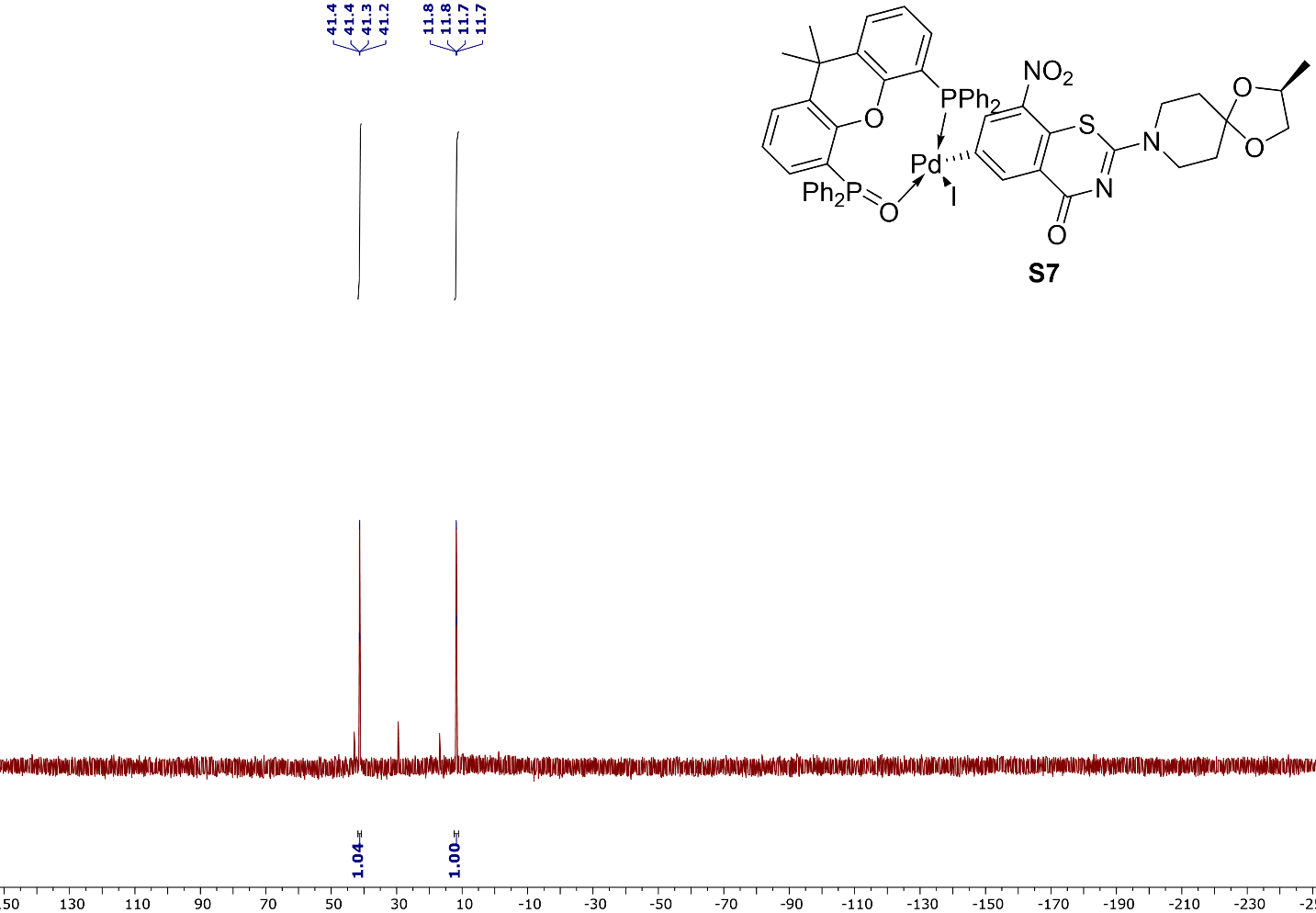


Supporting Figure DS45. ^31^P NMR of compound S7 (202 MHz, 300K, CDCl_3_ + 1% TMS).


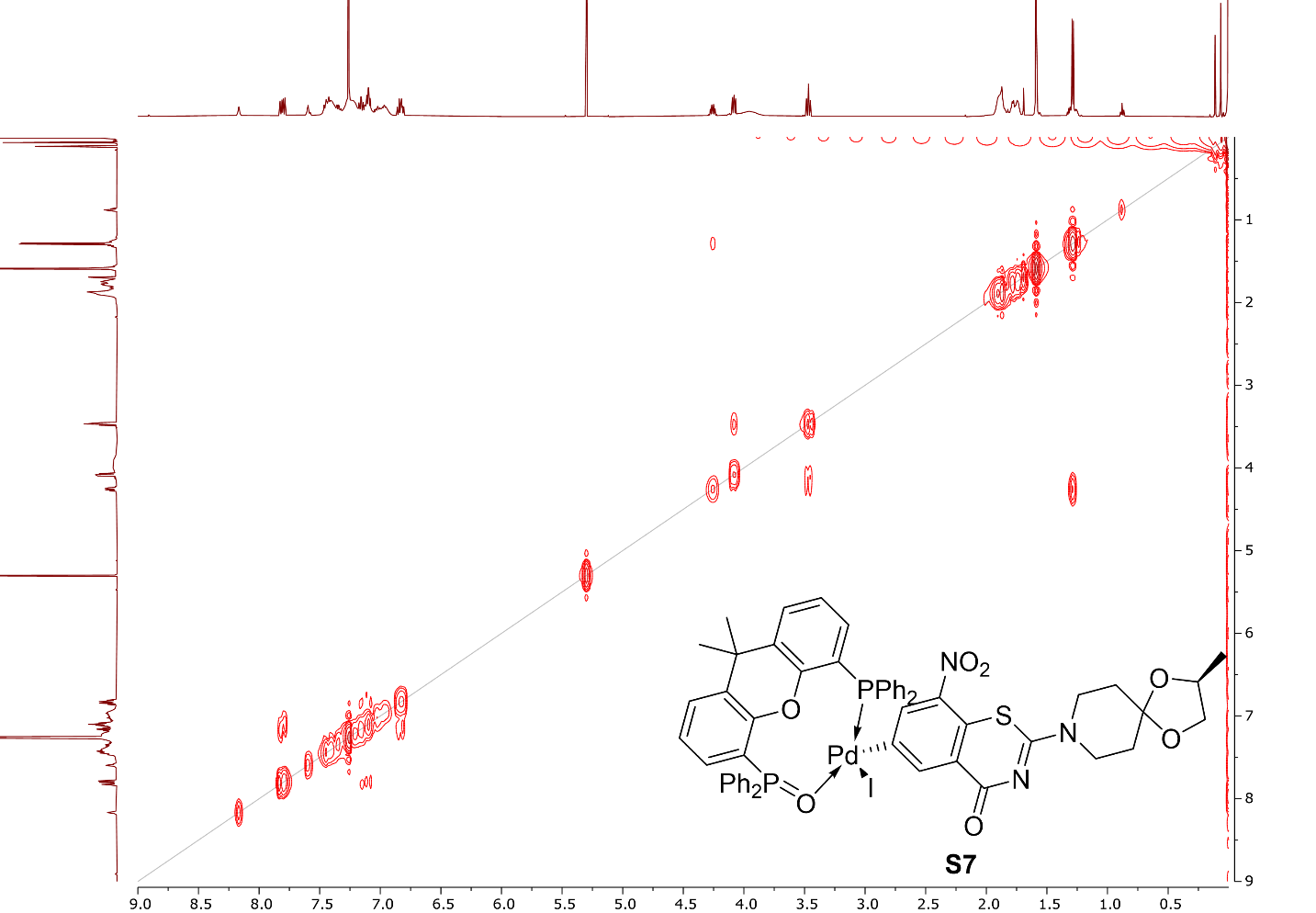


Supporting Figure DS46. COSY experiment of compound S7 (300K, CDCl_3_ + 1% TMS).


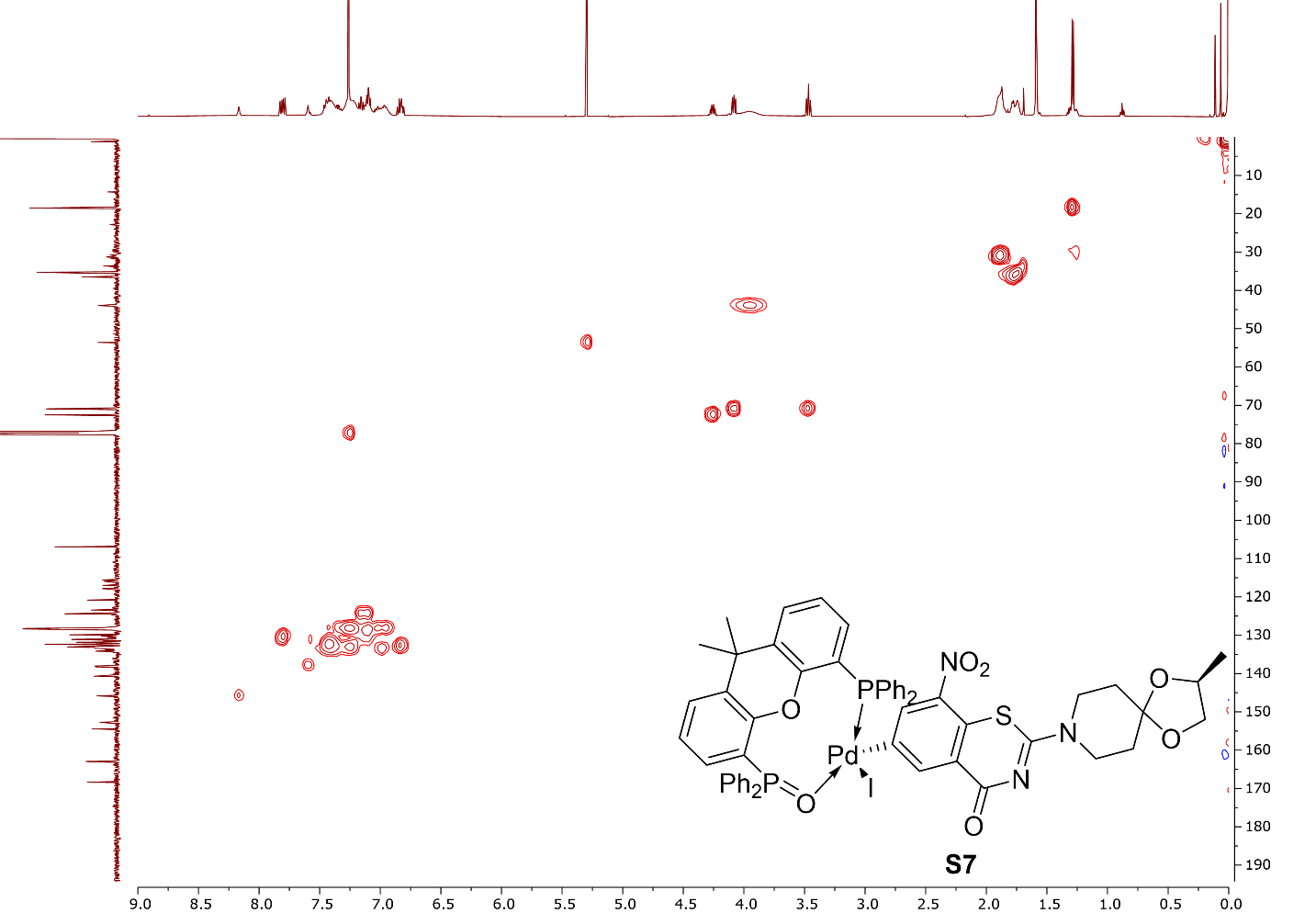


Supporting Figure DS47. HSQC experiment of compound S7 (300K, CDCl_3_ + 1% TMS).


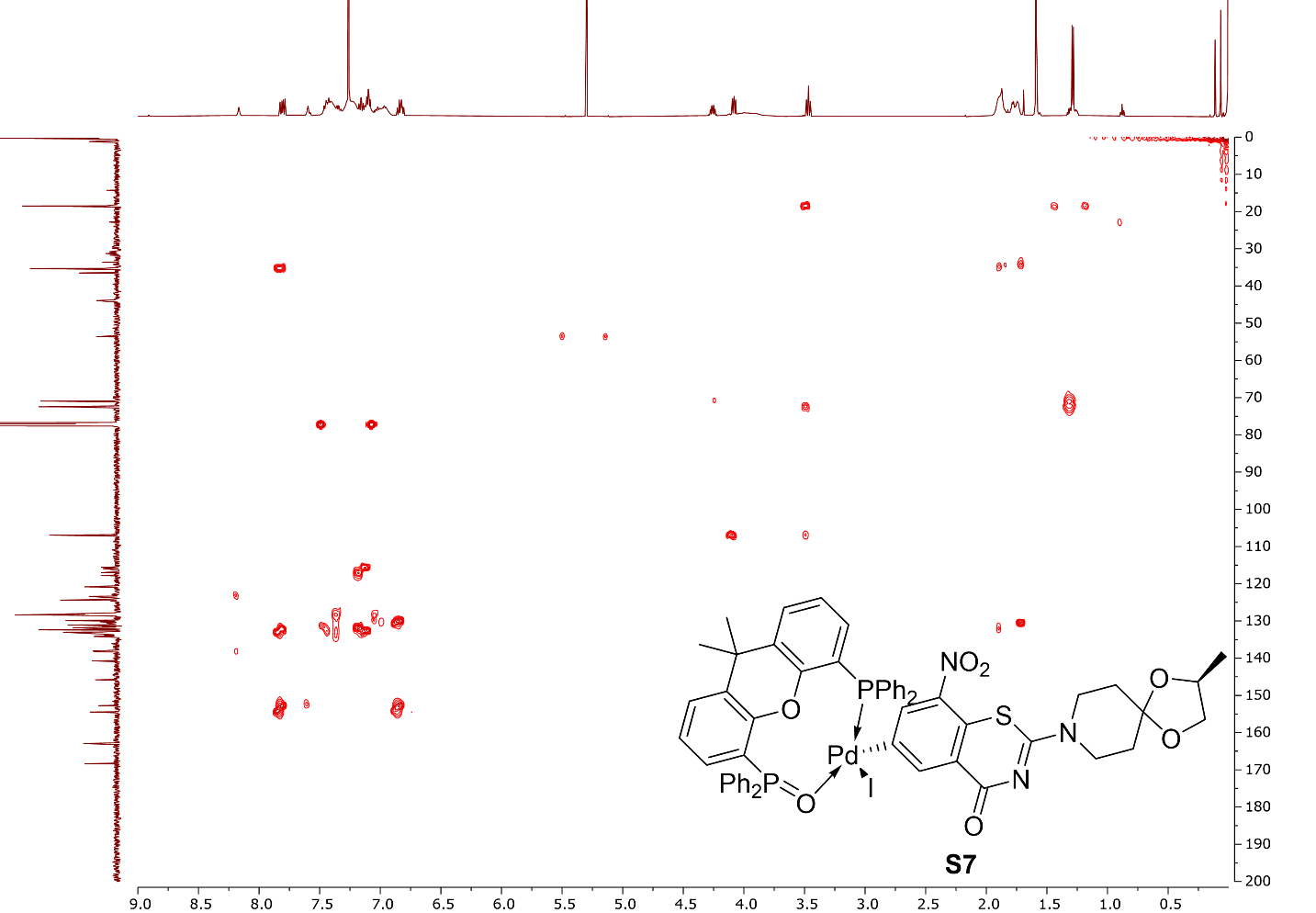


Supporting Figure DS48. HMBC experiment of compound S7 (300K, CDCl_3_ + 1% TMS).


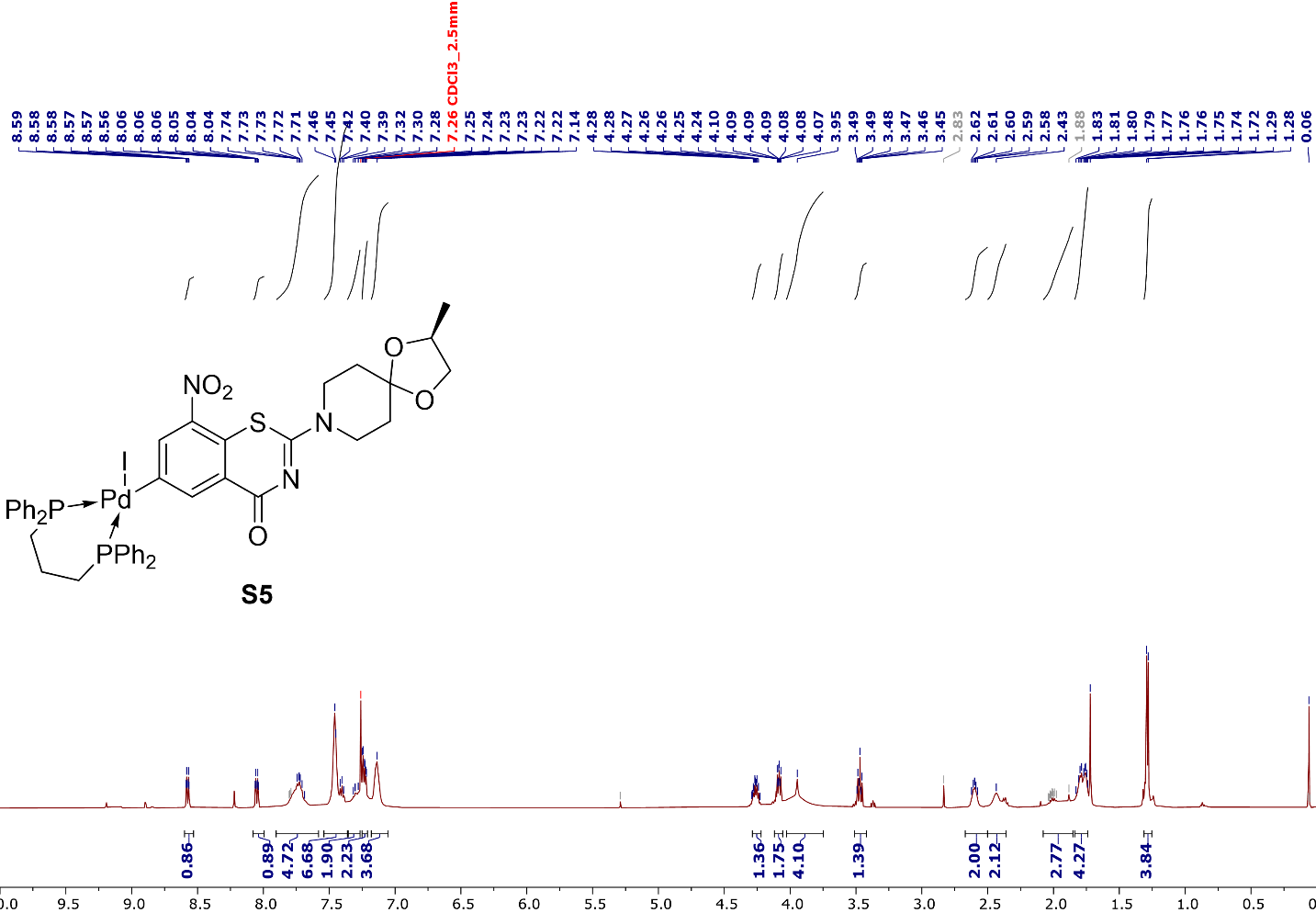


Supporting Figure DS49. ^1^H NMR of compound S5 (500 MHz, 300K, CDCl_3_ + 1% TMS).


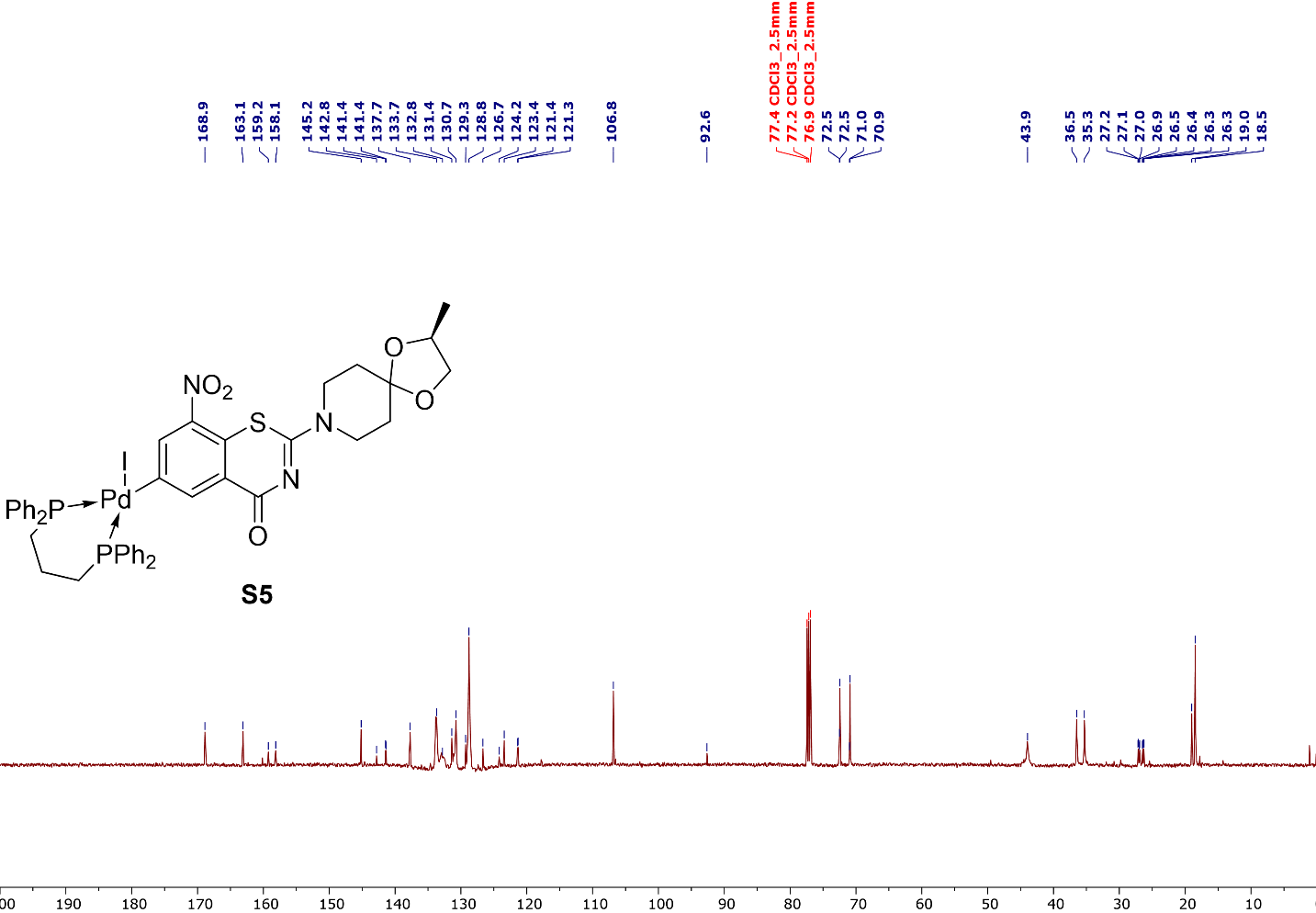


Supporting Figure DS50. ^13^C NMR of compound S5 (126 MHz, 300K, CDCl_3_ + 1% TMS).


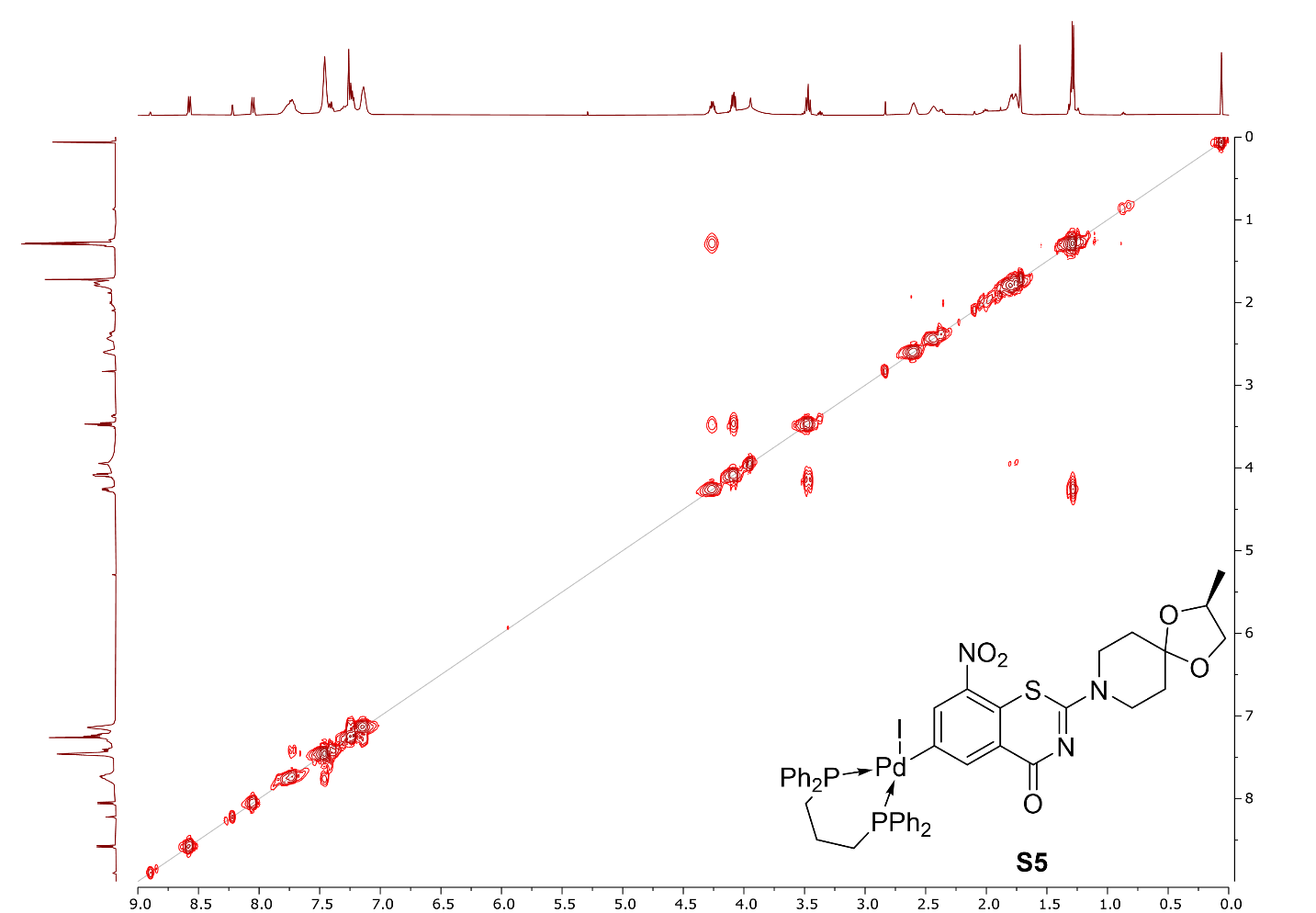


Supporting Figure DS51. COSY experiment of compound S5 (300K, CDCl_3_ + 1% TMS).


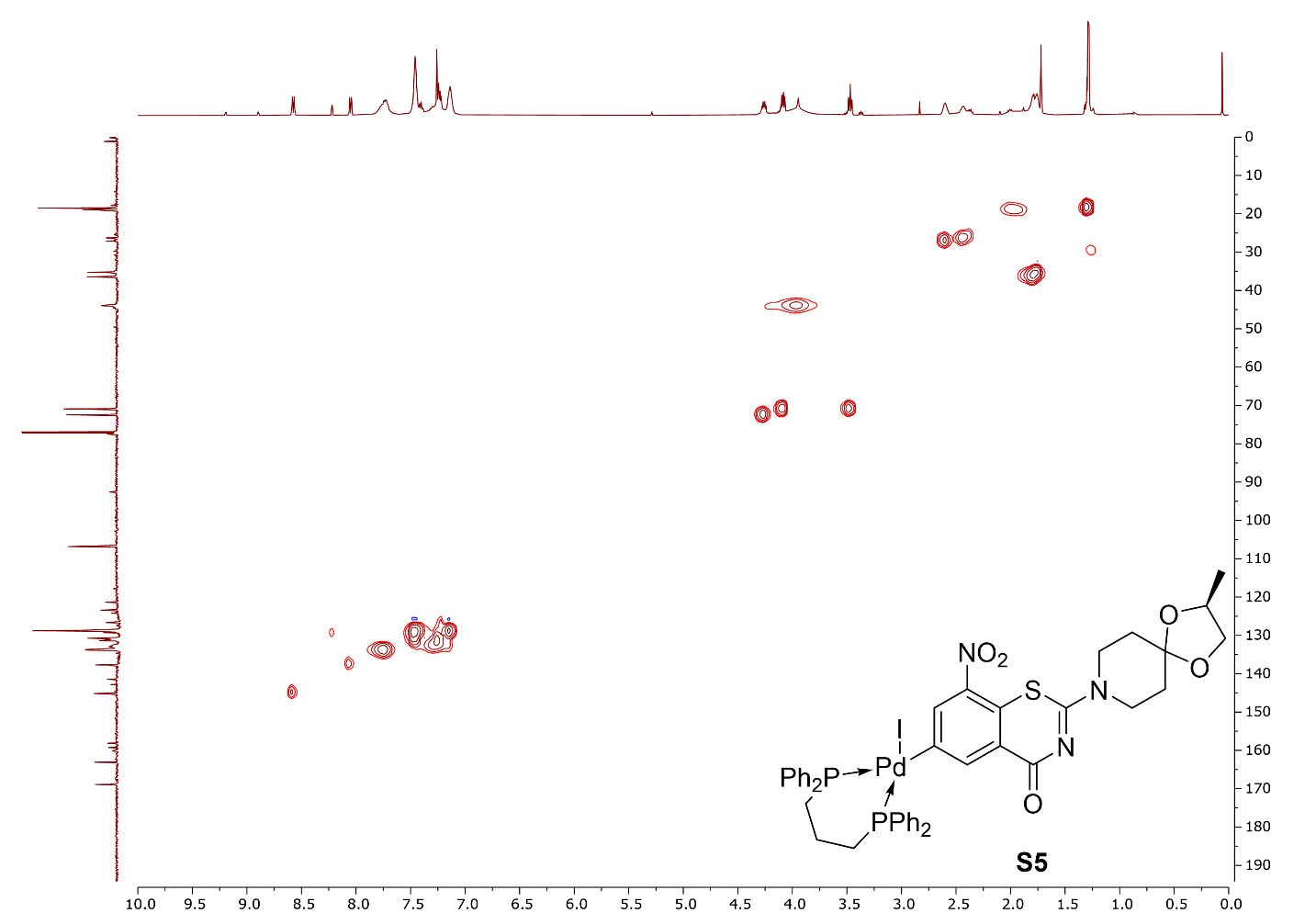


Supporting Figure DS52. HSQC experiment of compound S5 (300K, CDCl_3_ + 1% TMS).


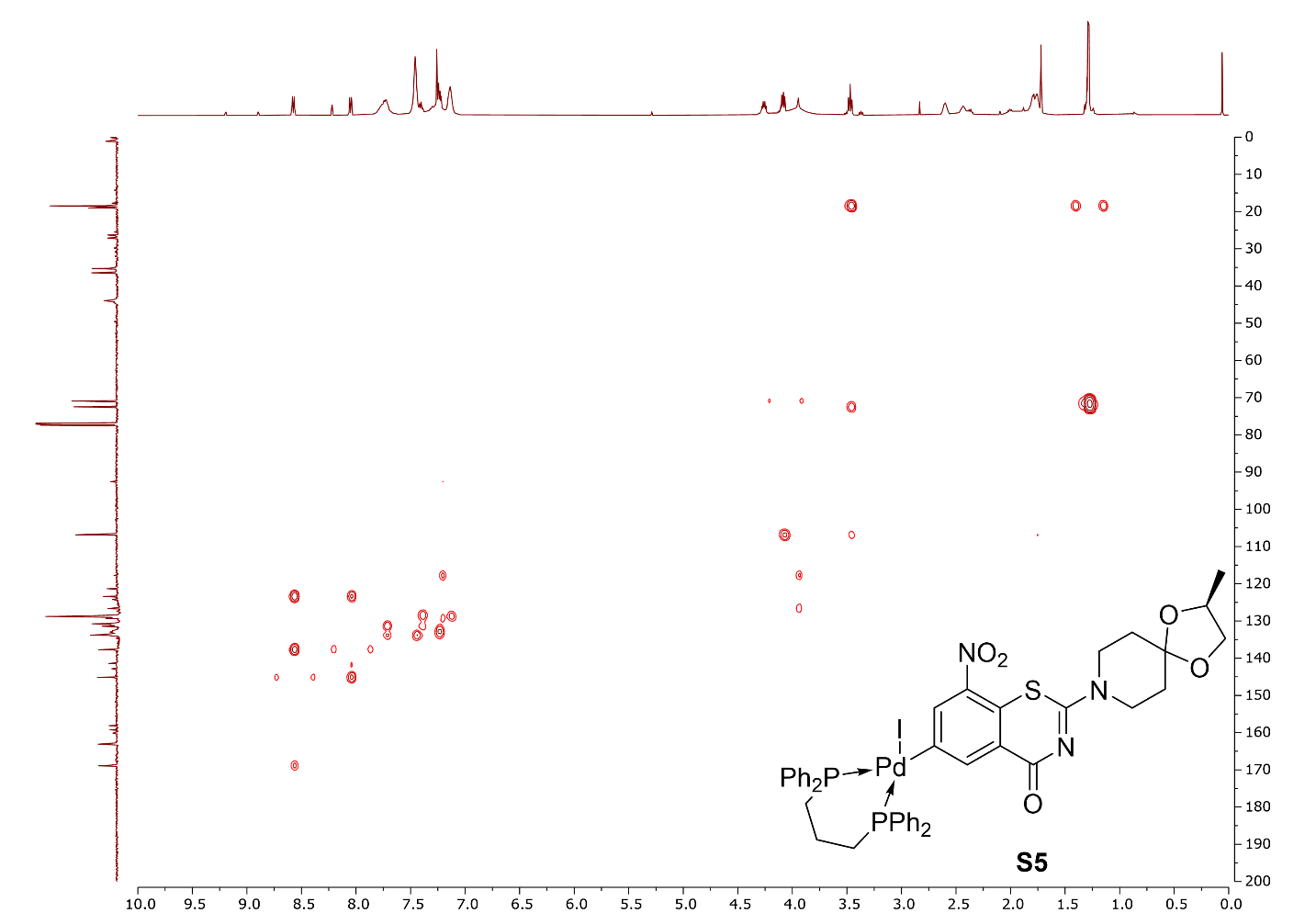


Supporting Figure DS53. HMBC experiment of compound S5 (300K, CDCl_3_ + 1% TMS).


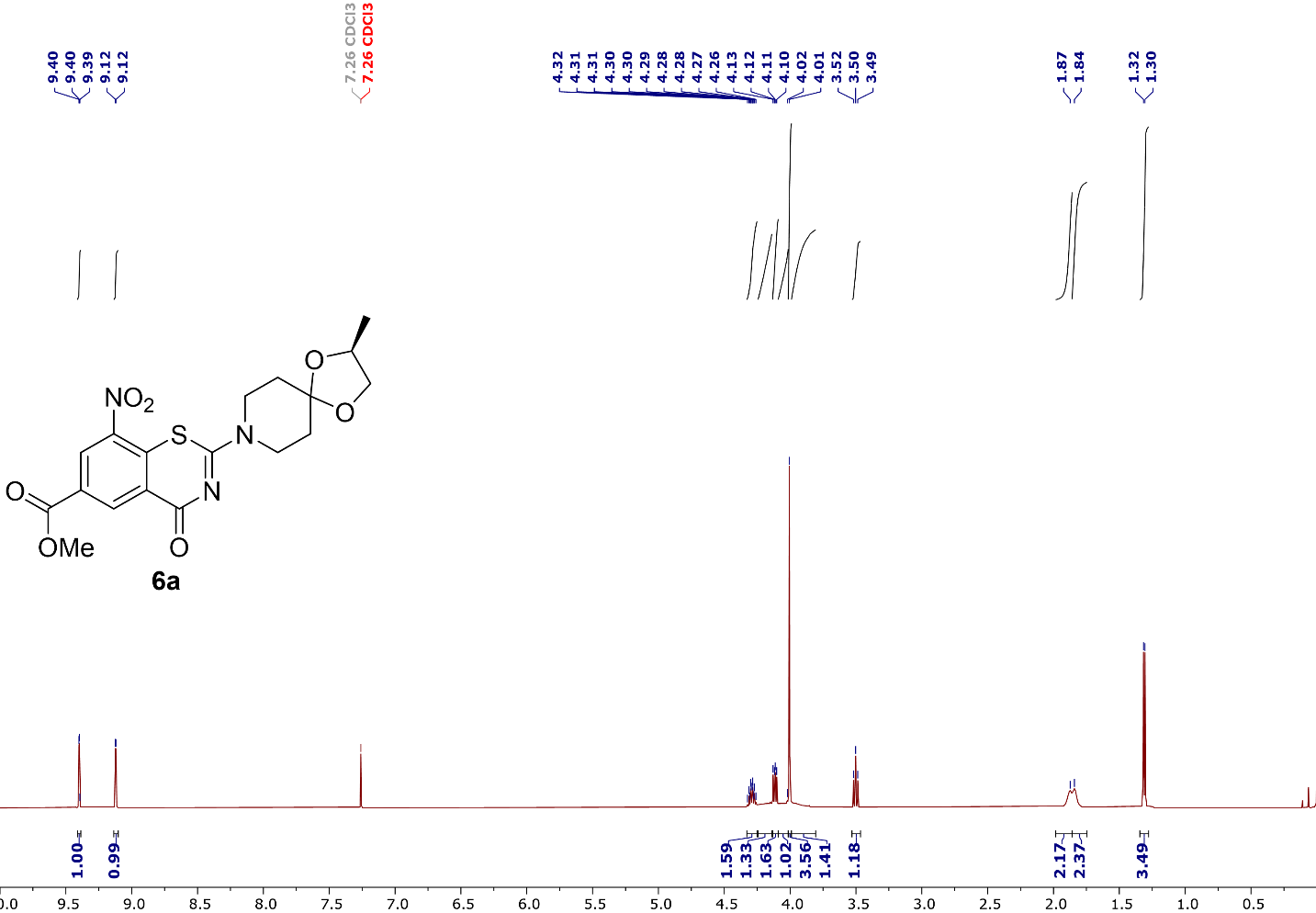


Supporting Figure DS54. ^1^H NMR of compound 6a (500 MHz, 300K, CDCl_3_ + 1% TMS).


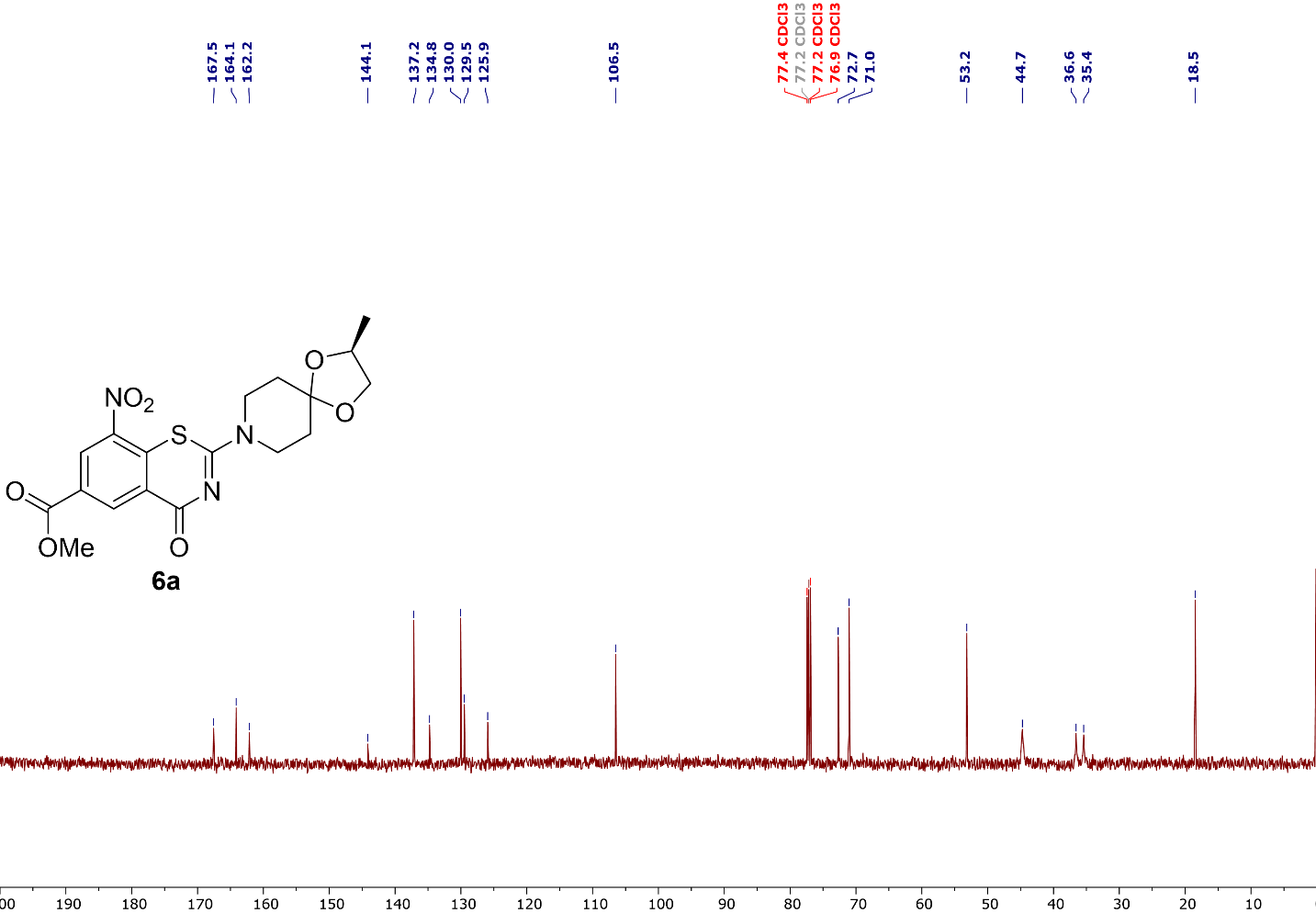


Supporting Figure DS55. ^13^C NMR of compound 6a (126 MHz, 300K, CDCl_3_ + 1% TMS).


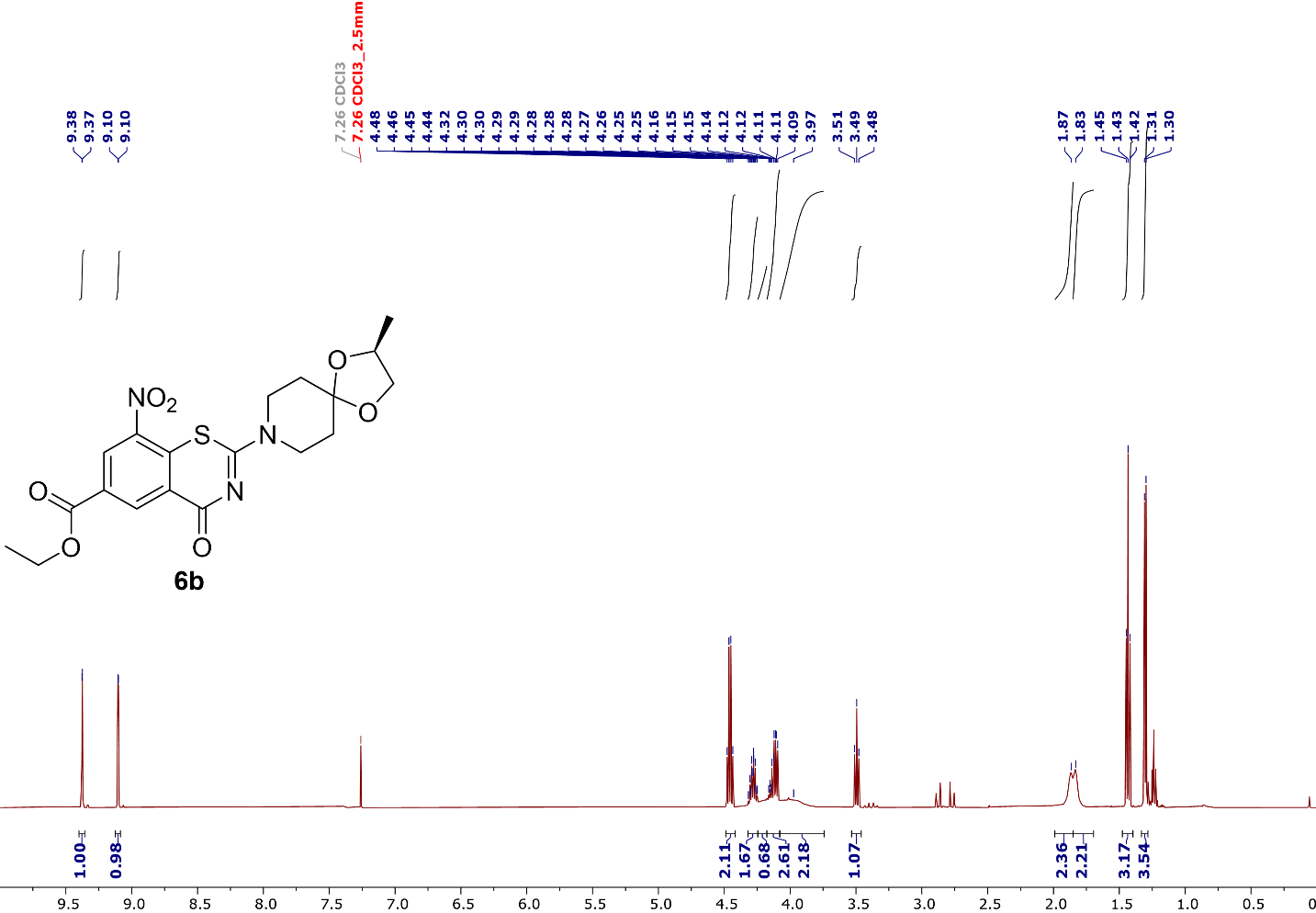


Supporting Figure DS56. ^1^H NMR of compound 6b (500 MHz, 300K, CDCl_3_).


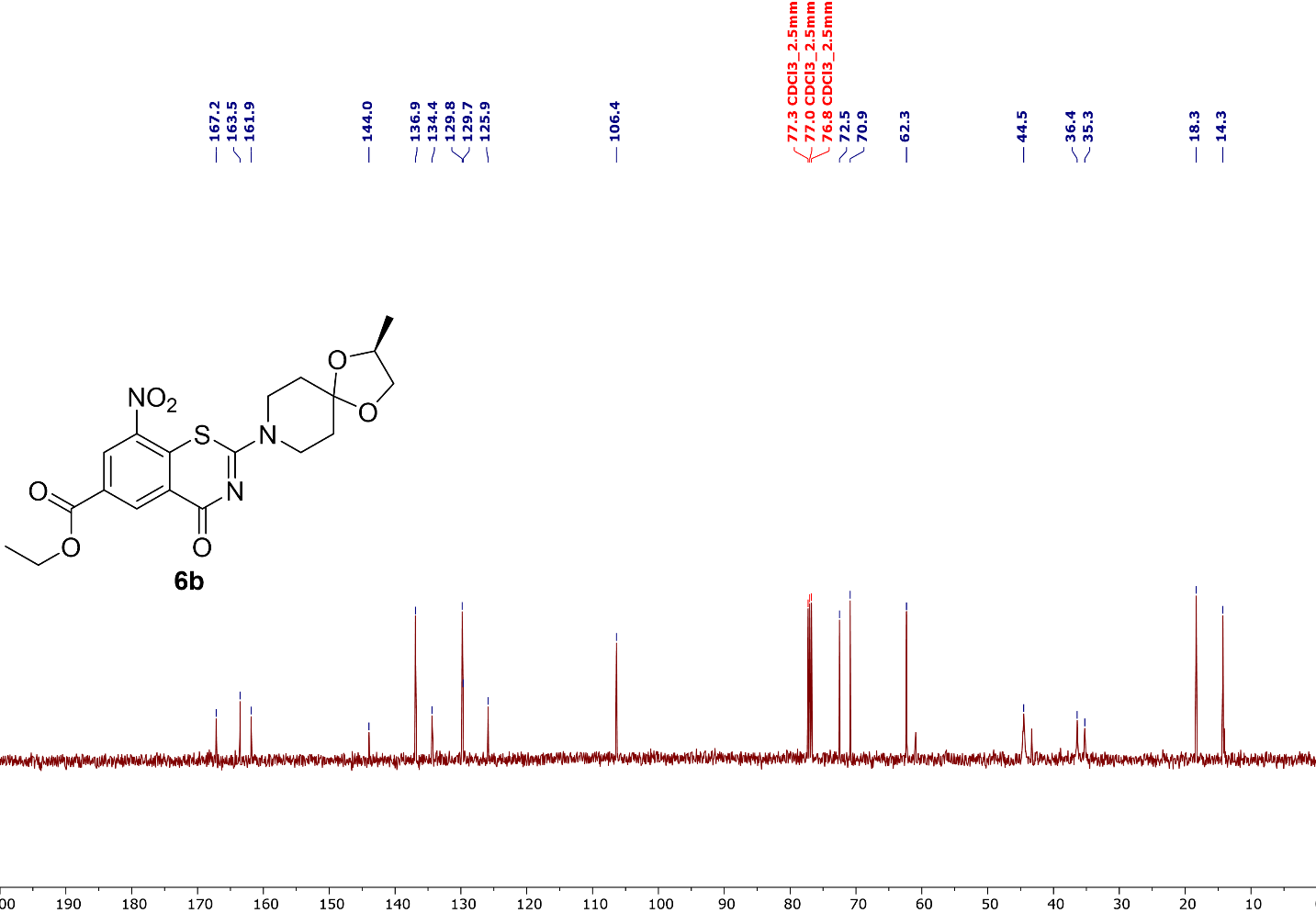


Supporting Figure DS57. ^1^H NMR of compound 6b (126 MHz, 300K, CDCl_3_).


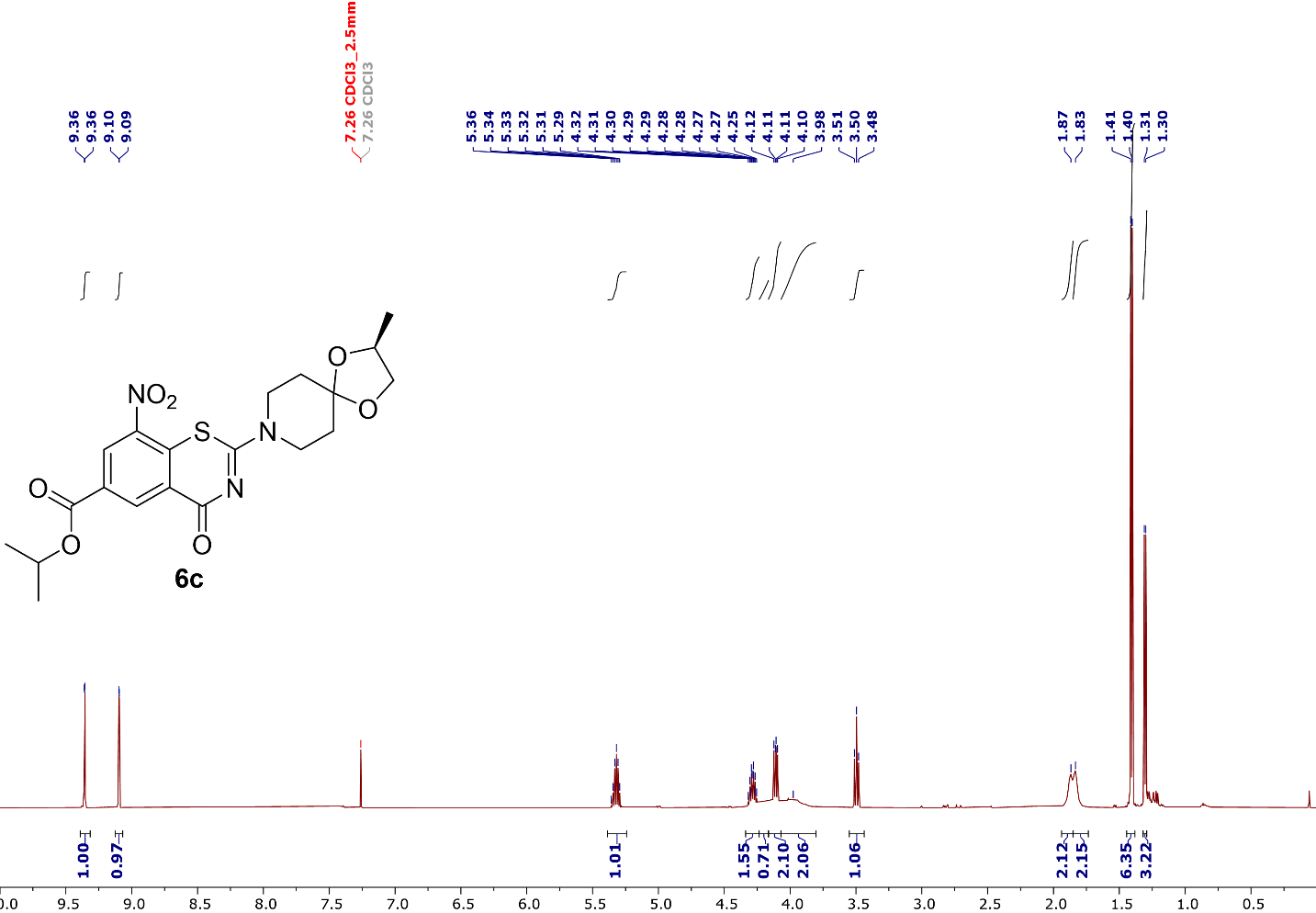


Supporting Figure DS58. ^1^H NMR of compound 6c (500 MHz, 300K, CDCl_3_).


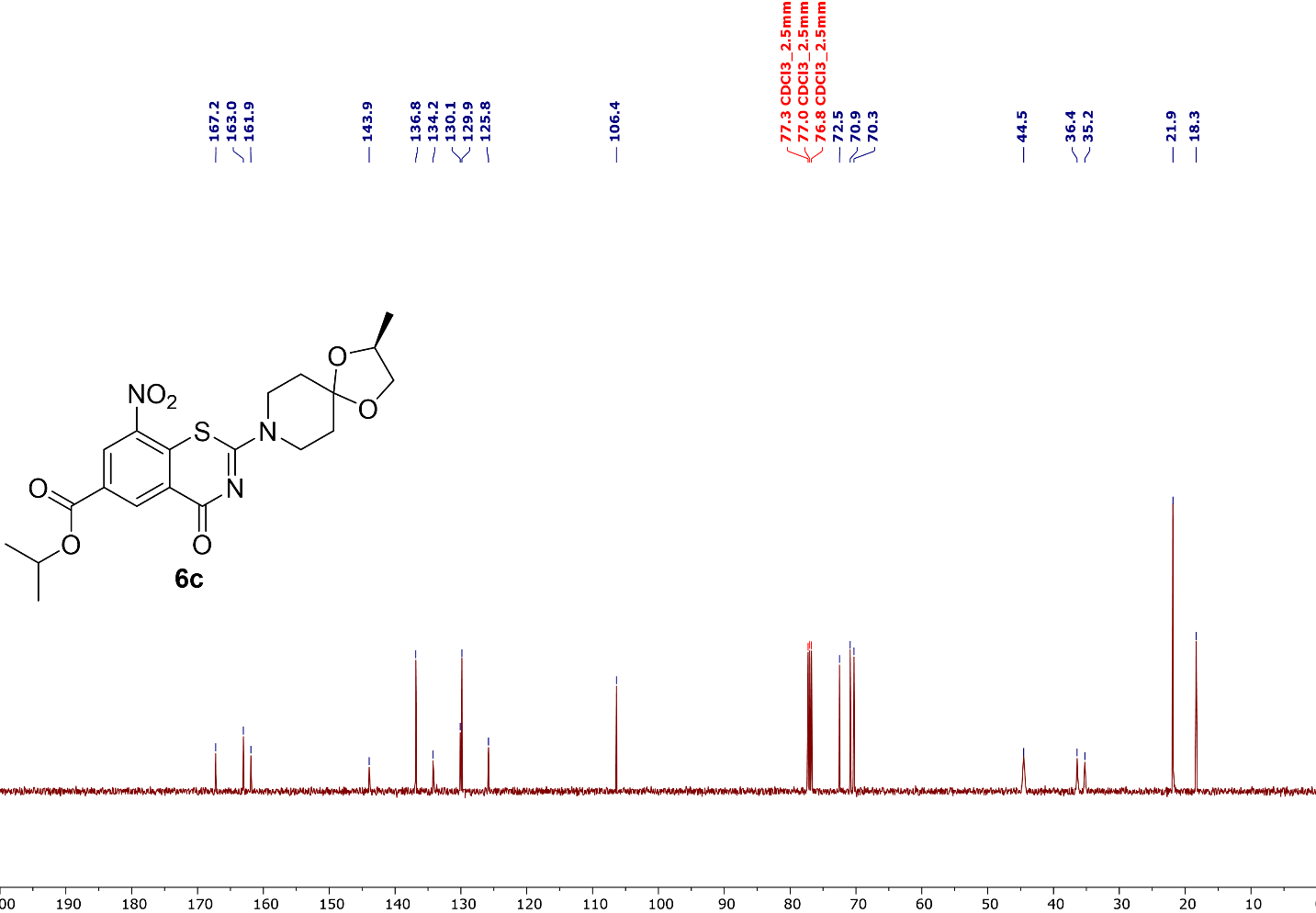


Supporting Figure DS59. ^13^C NMR of compound 6c (126 MHz, 300K, CDCl_3_).


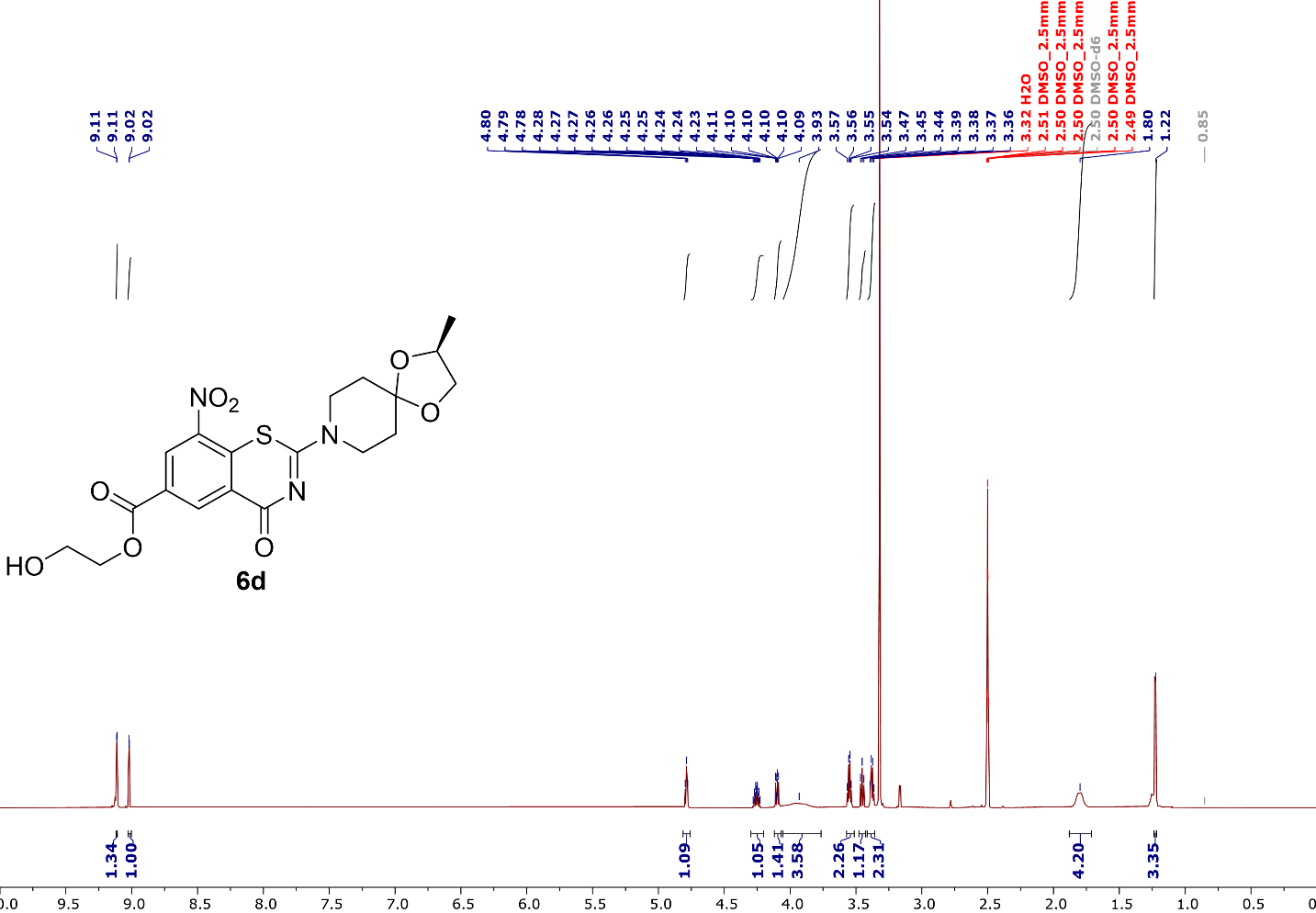


Supporting Figure DS60. ^1^H NMR of compound 6d (600 MHz, 300K, DMSO-*d_6_*).


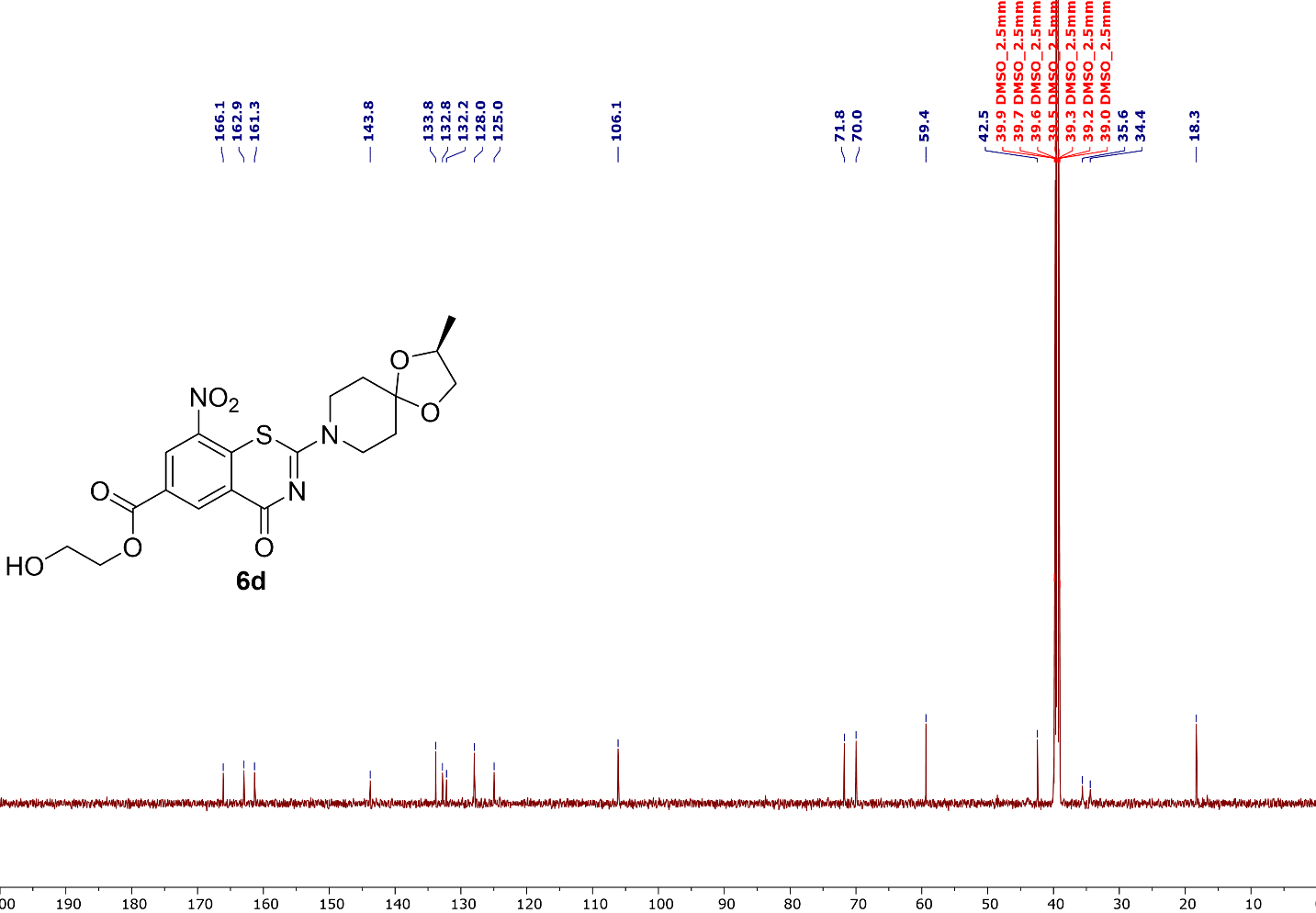


Supporting Figure DS61. ^13^C NMR of compound 6d (151 MHz, 300K, DMSO-*d_6_*).


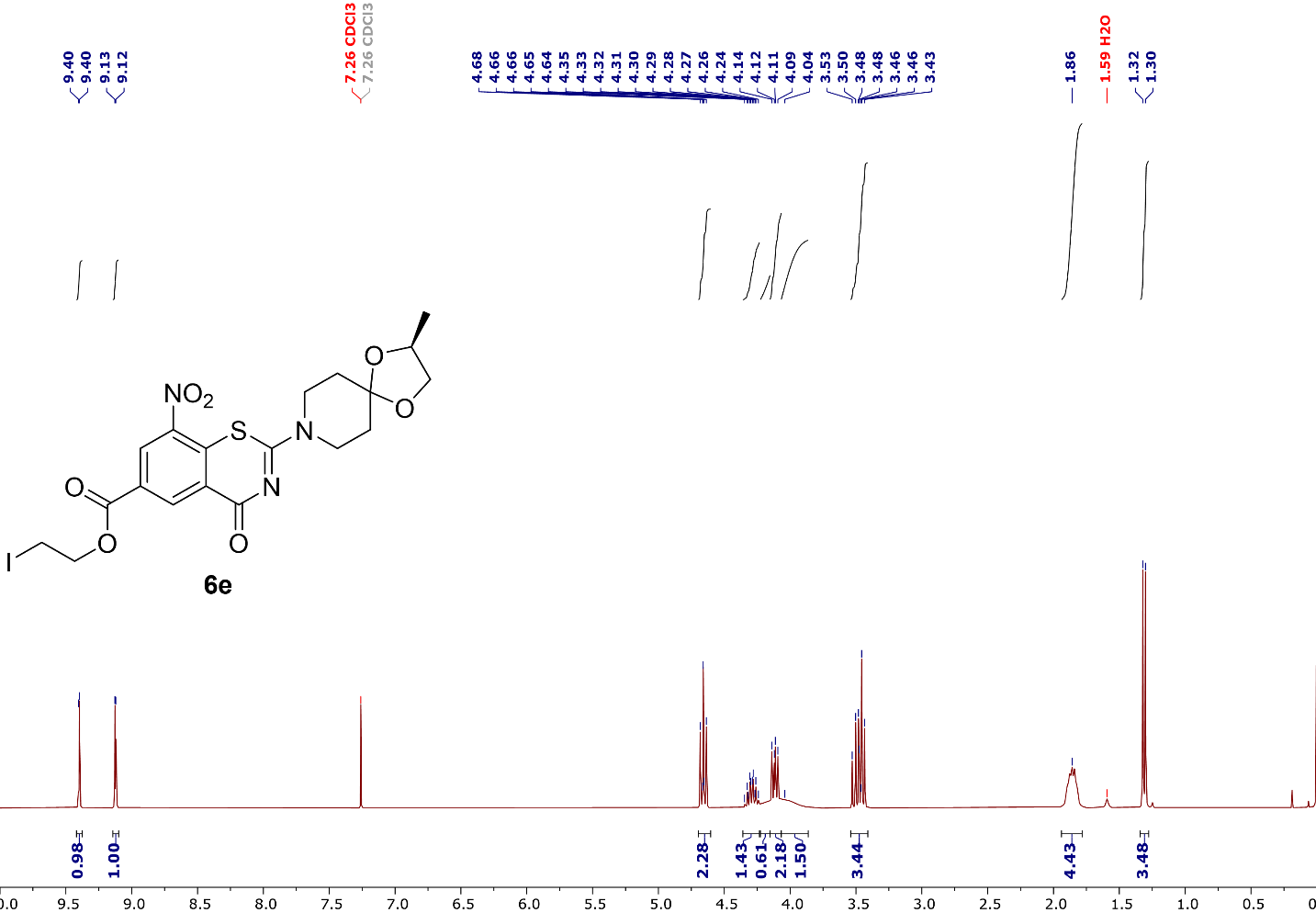


Supporting Figure DS62. ^1^H NMR of compound 6e (300 MHz, 300K, CDCl_3_ + 1% TMS).


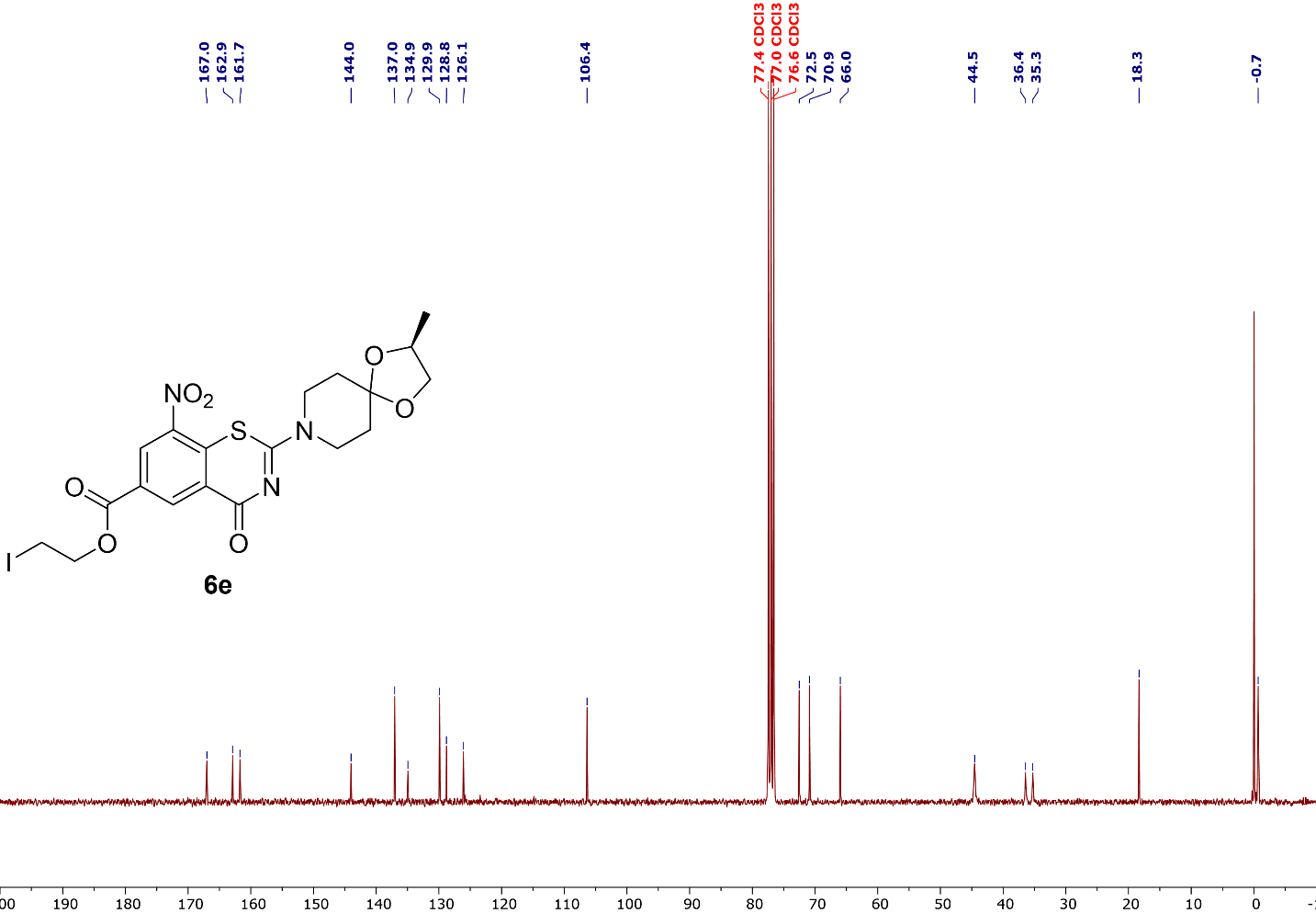


Supporting Figure DS63. ^13^C NMR of compound 6e (75 MHz, 300K, CDCl_3_ + 1% TMS).


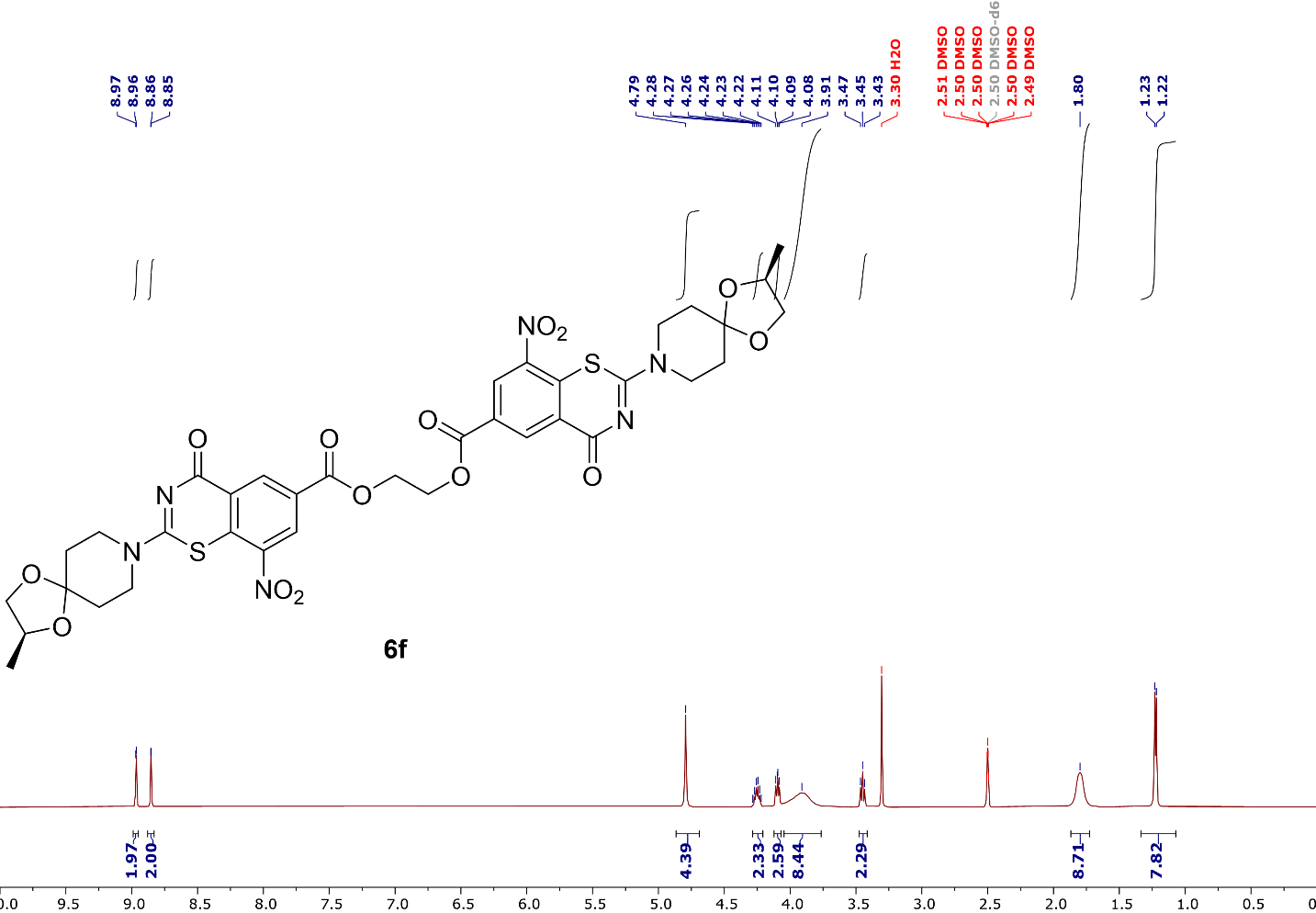


Supporting Figure DS64. ^1^H NMR of compound 6f (500 MHz, 300K, DMSO-*d_6_*).


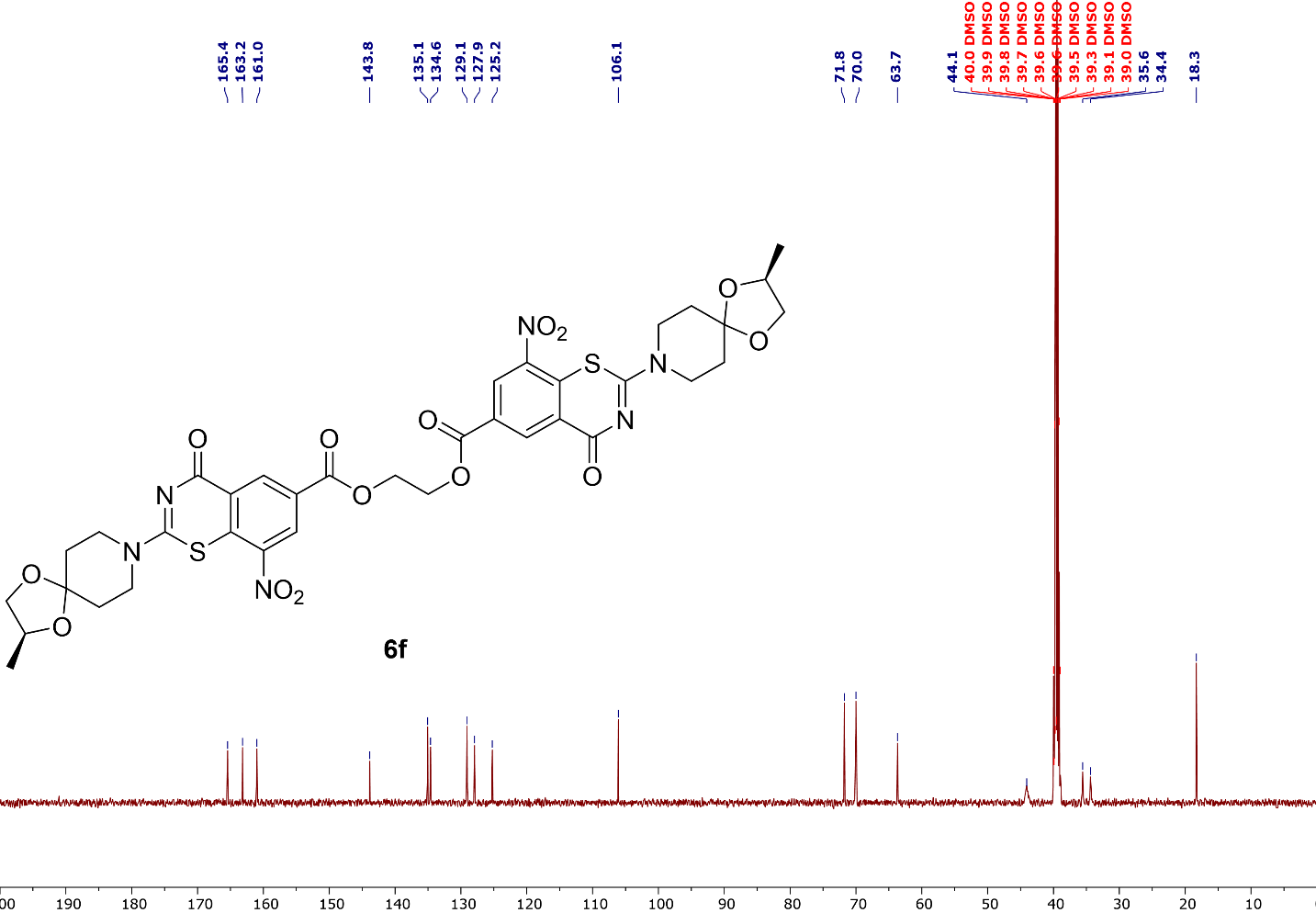


Supporting Figure DS65. ^13^C NMR of compound 6f (126 MHz, DMSO-*d_6_*).


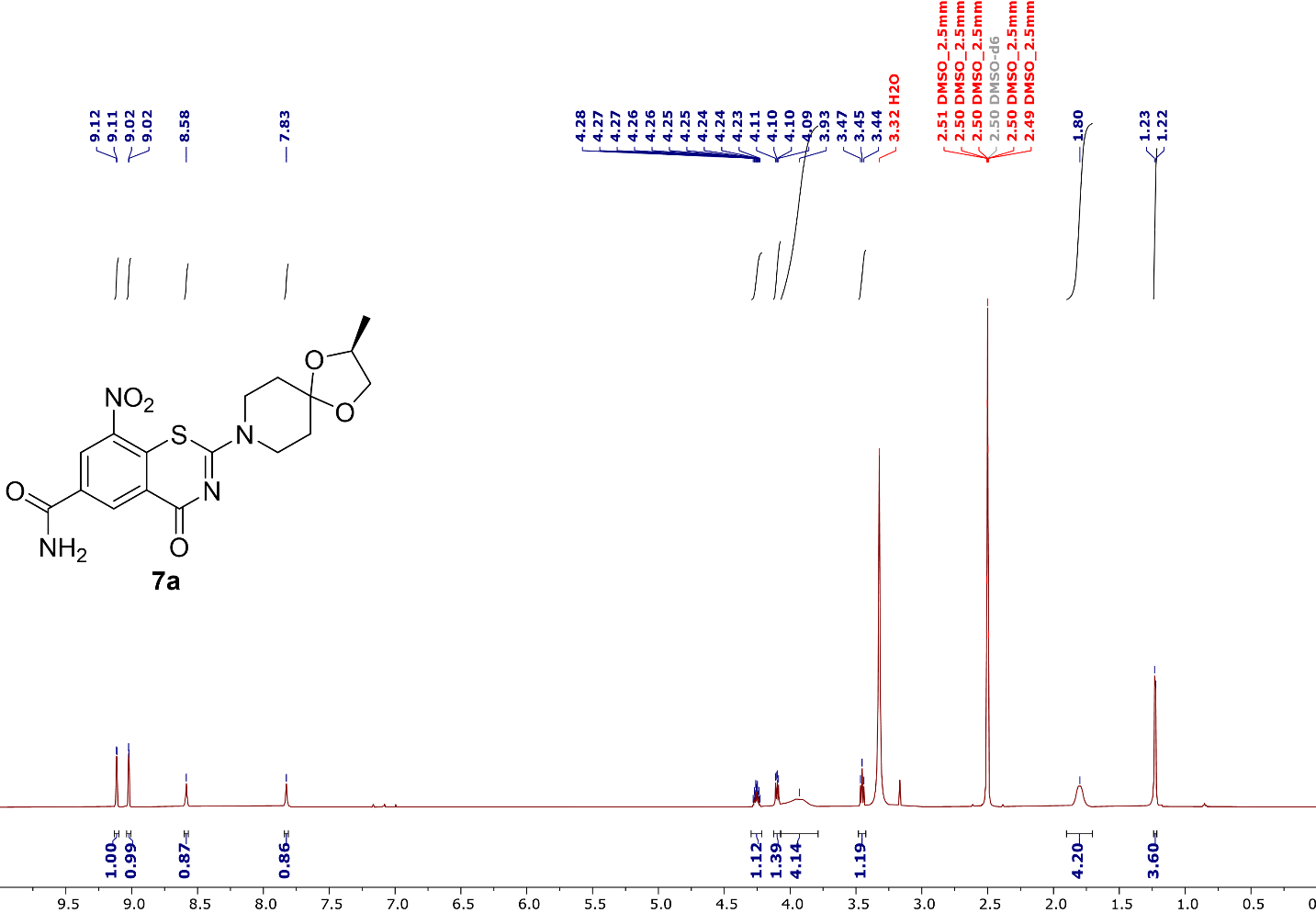


Supporting Figure DS66. ^1^H NMR of compound 7a (600 MHz, 300K, DMSO-*d_6_*).


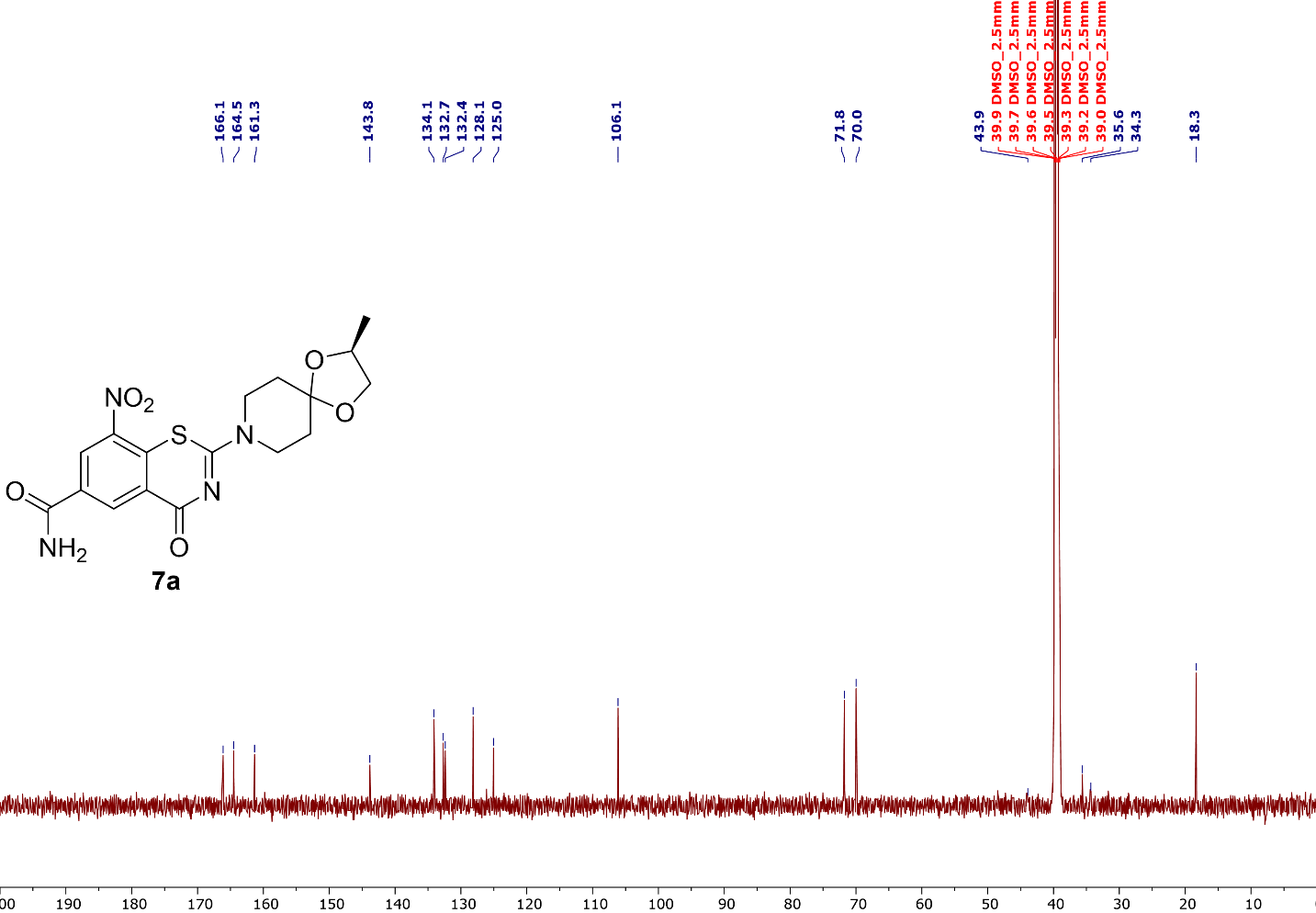


Supporting Figure DS67. ^13^C NMR of compound 7a (151 MHz, 300K, DMSO-*d_6_*).


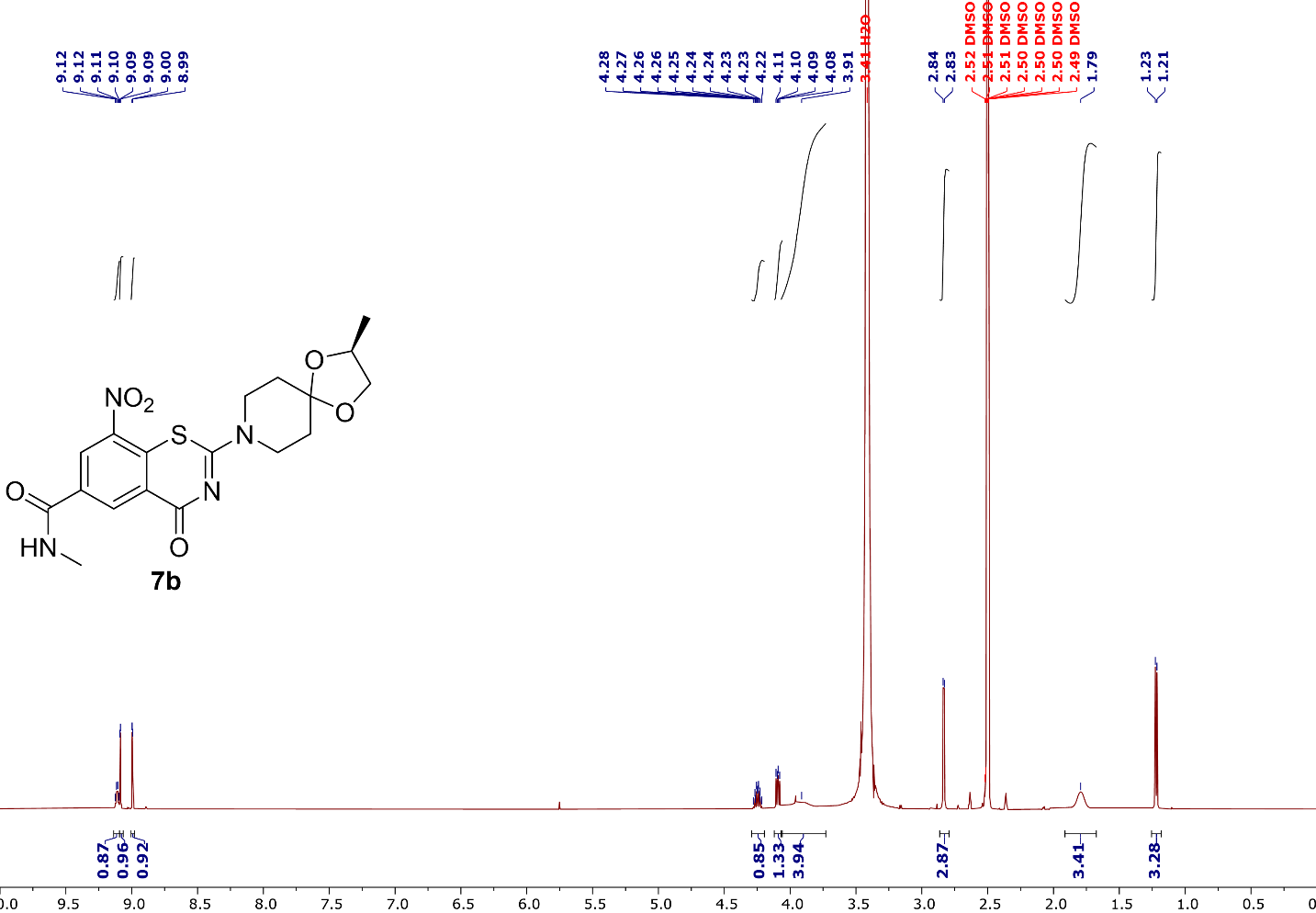


Supporting Figure DS68. ^1^H NMR of compound 7b (500 MHz, 300K, DMSO-*d_6_*).


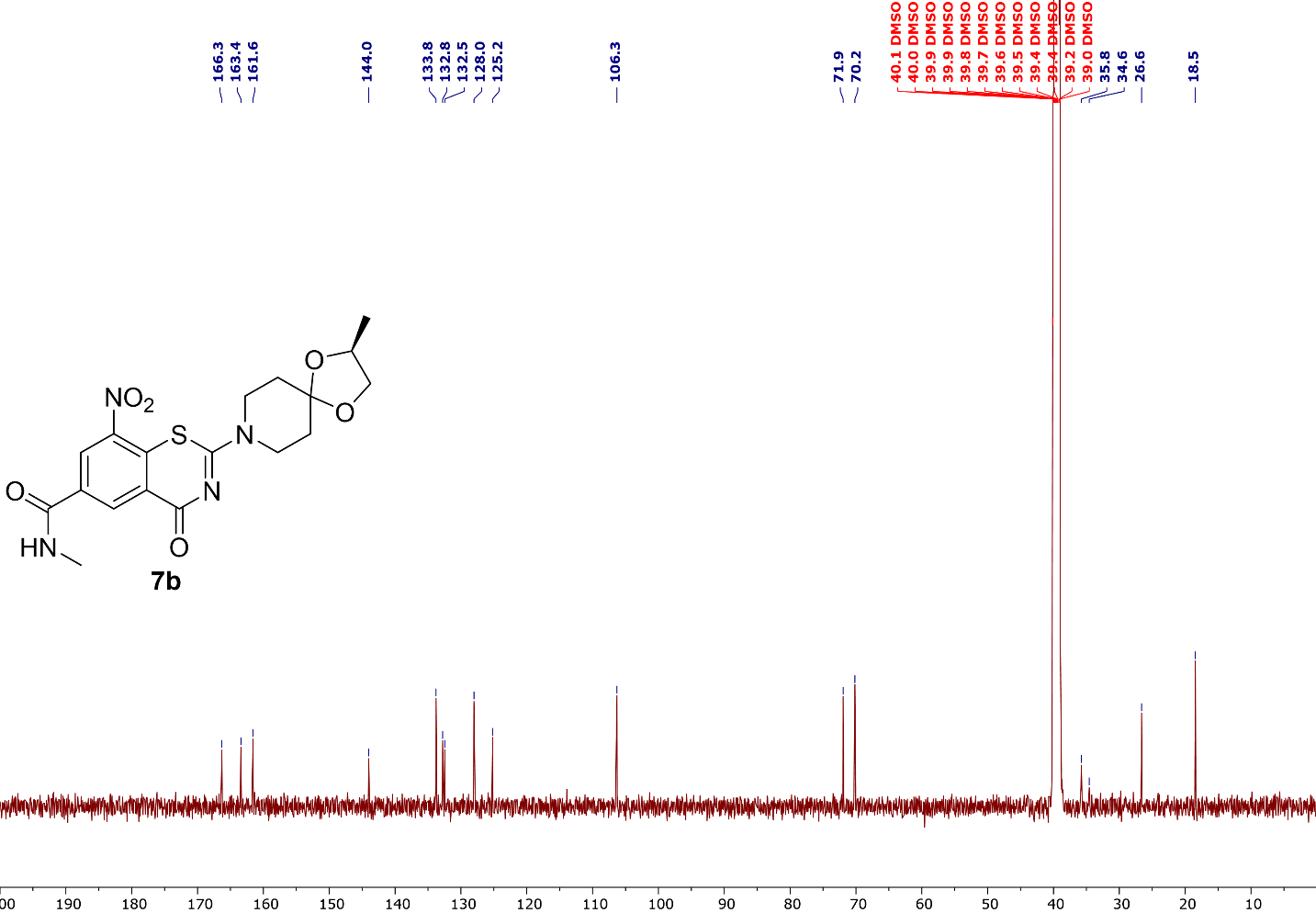


Supporting Figure DS69. ^13^C NMR of compound 7b (126 MHz, 300K, DMSO-*d_6_*).


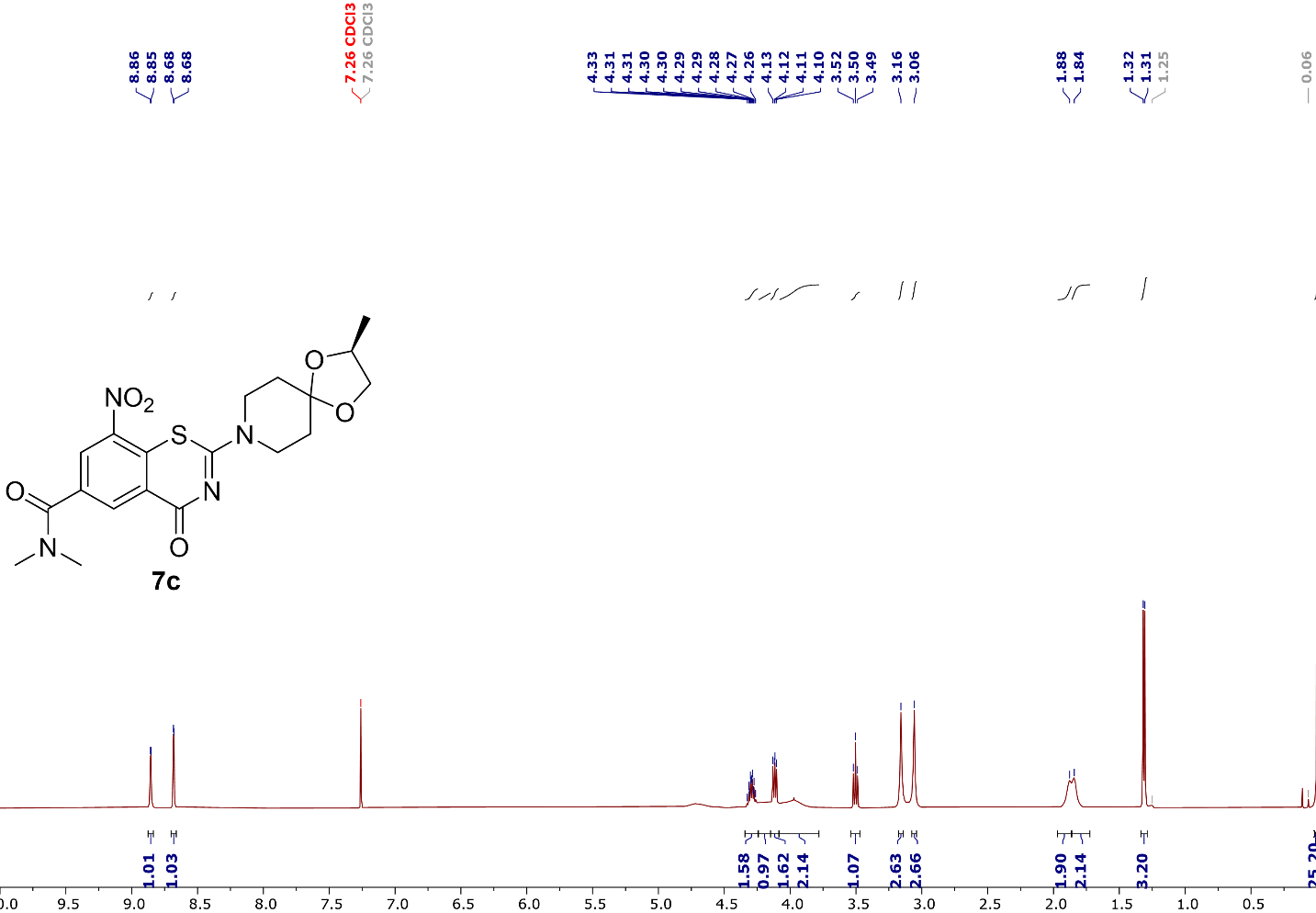


Supporting Figure DS70. ^1^H NMR of compound 7c (500 MHz, 300K, CDCl_3_ + 1% TMS).


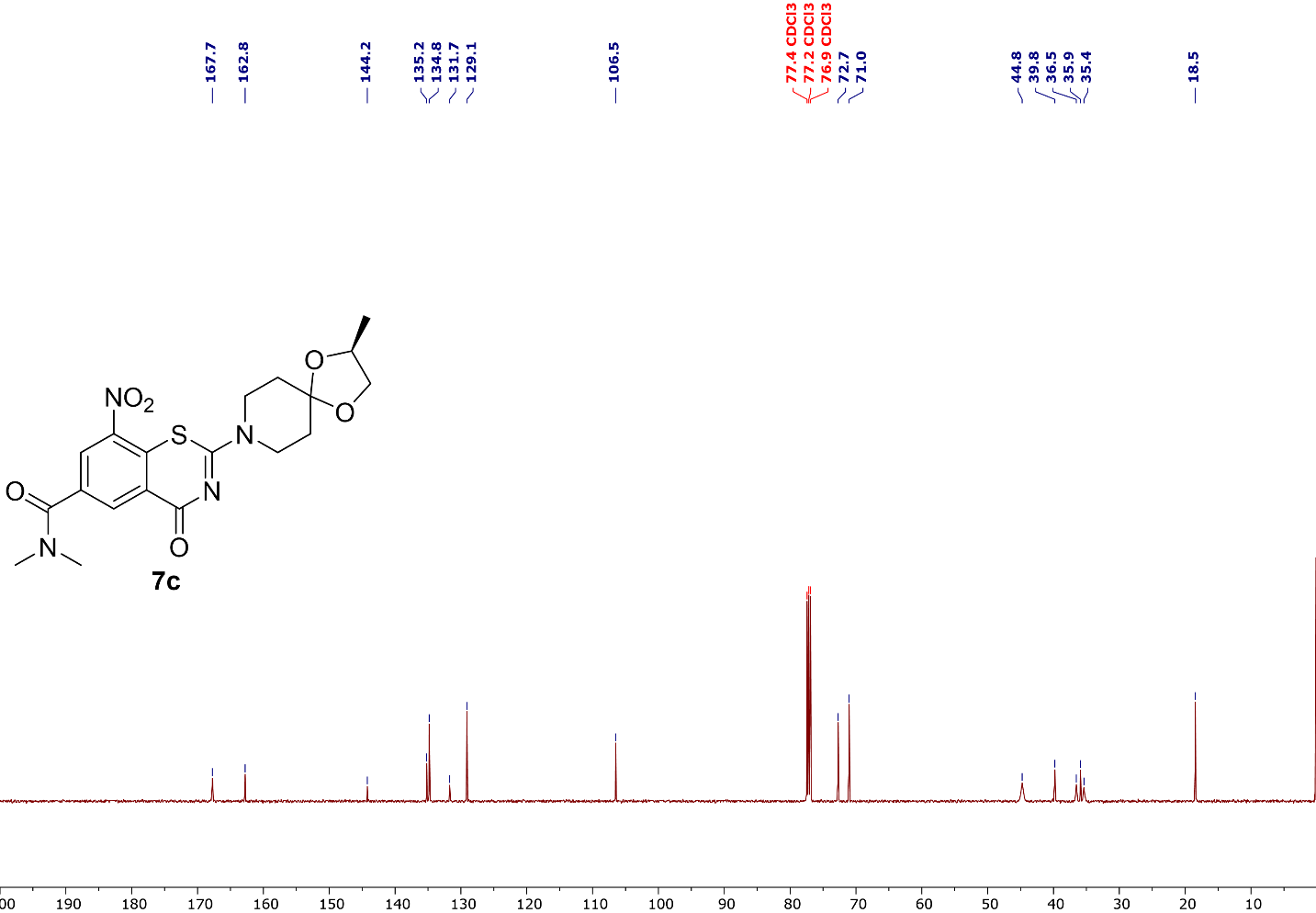


Supporting Figure DS71. ^13^C NMR of compound 7c (126 MHz, 300K, CDCl_3_ + 1% TMS).


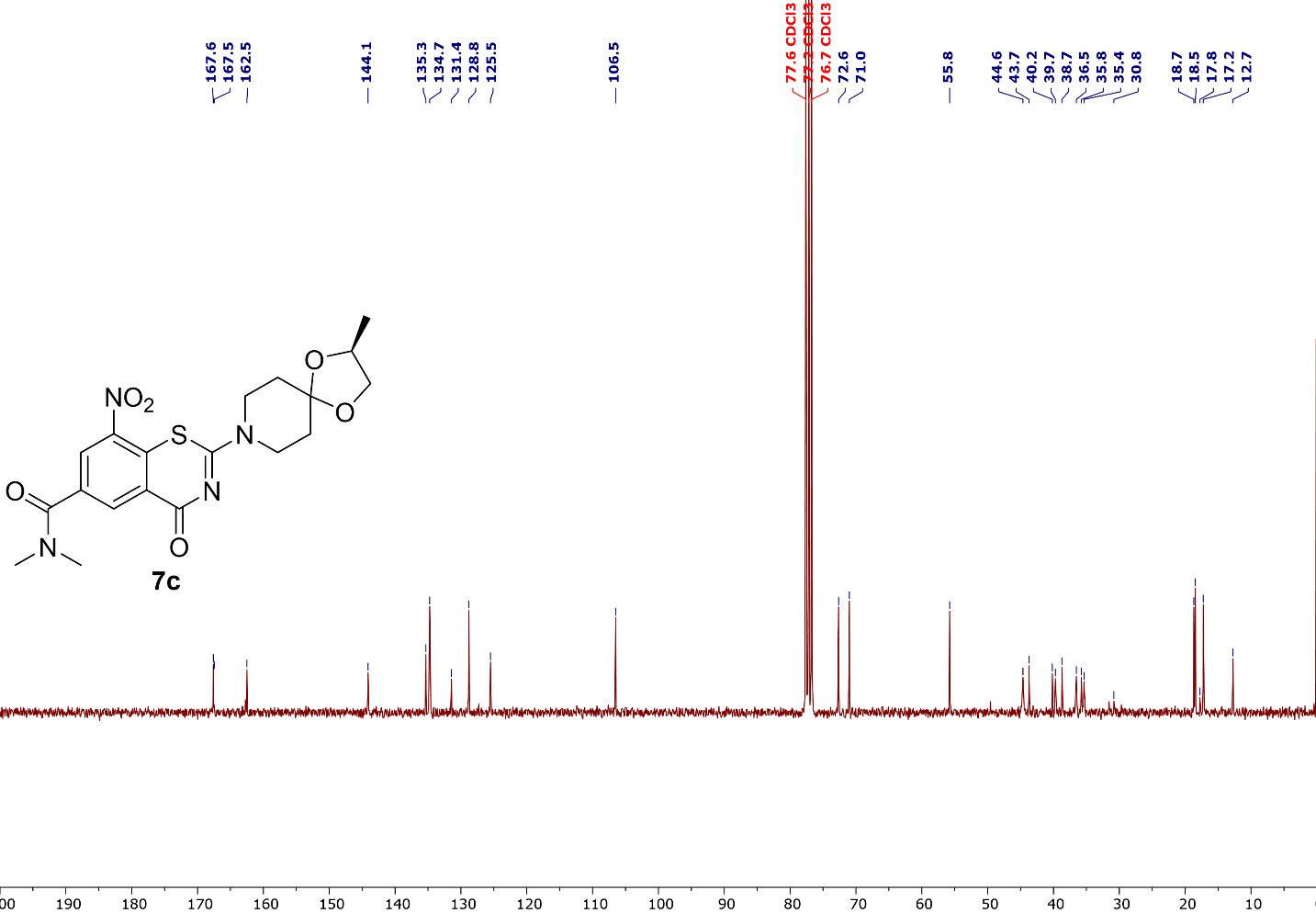


Supporting Figure DS72. ^13^C NMR of unpurified compound 7c (75 MHz, 300K, CDCl_3_ + 1% TMS).


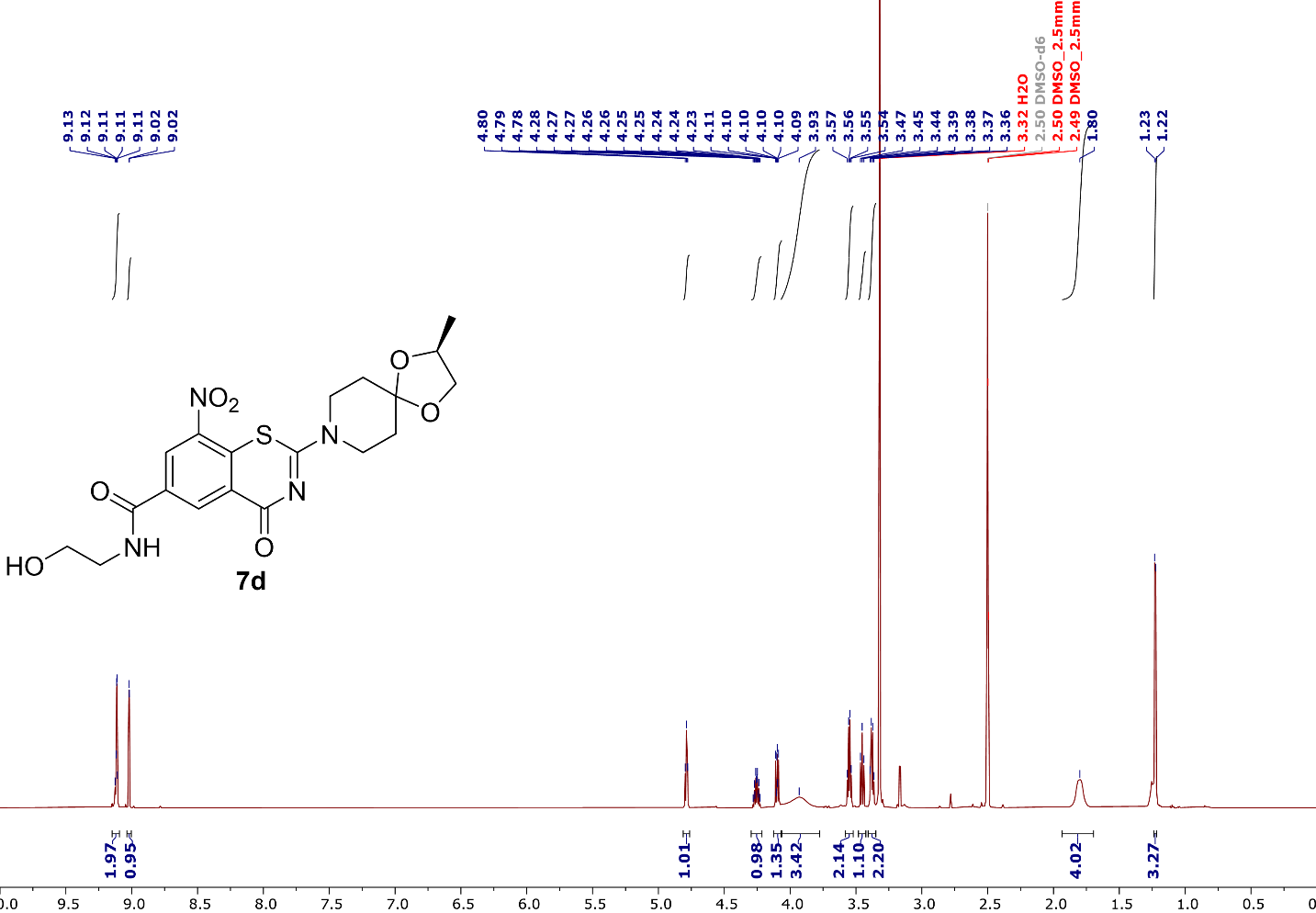


Supporting Figure DS73. ^1^H NMR of compound 7d (600 MHz, 300K, DMSO-*d_6_*).


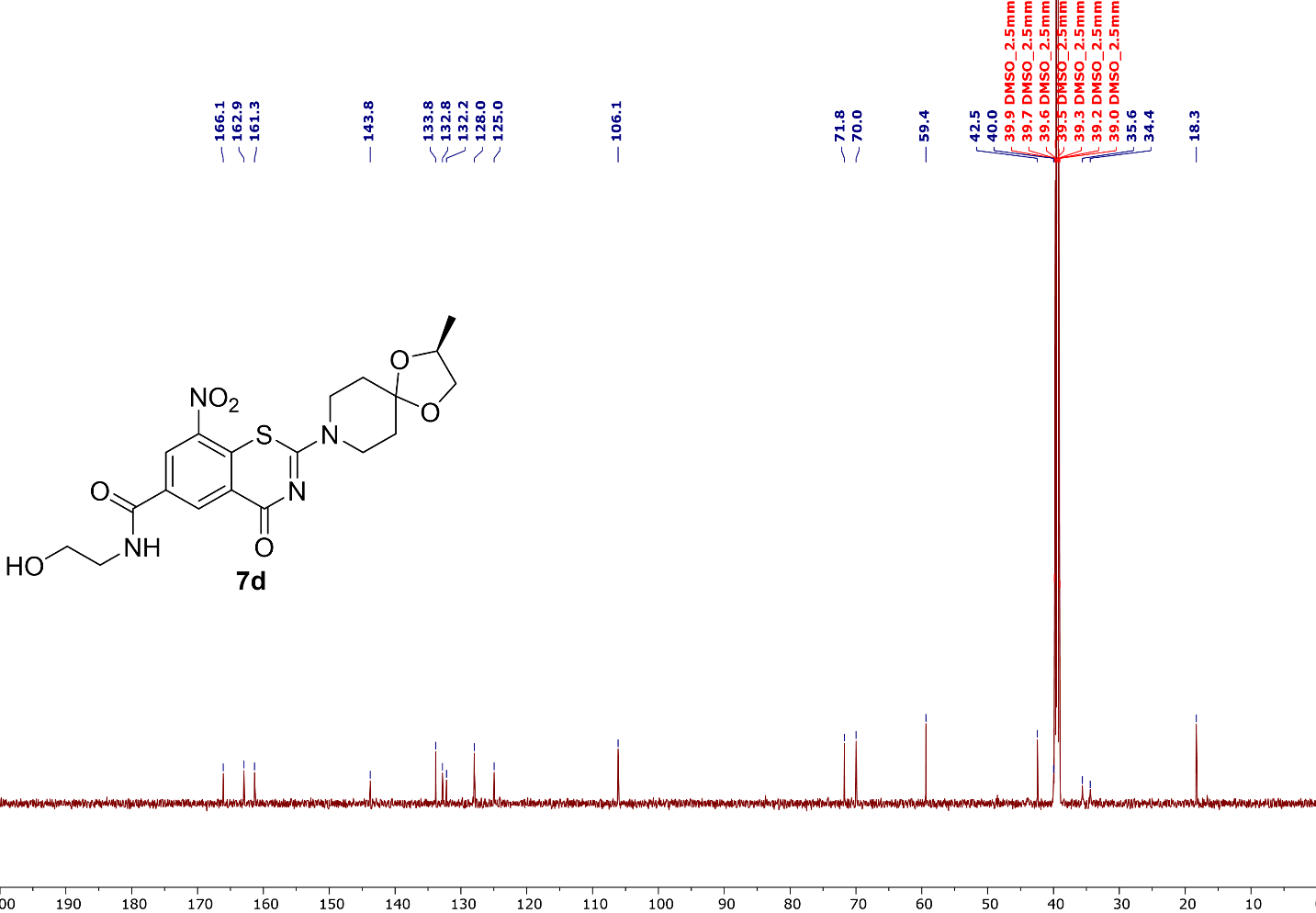


Supporting Figure DS74. ^13^C NMR of compound 7d (151 MHz, 300K, DMSO-*d_6_*).


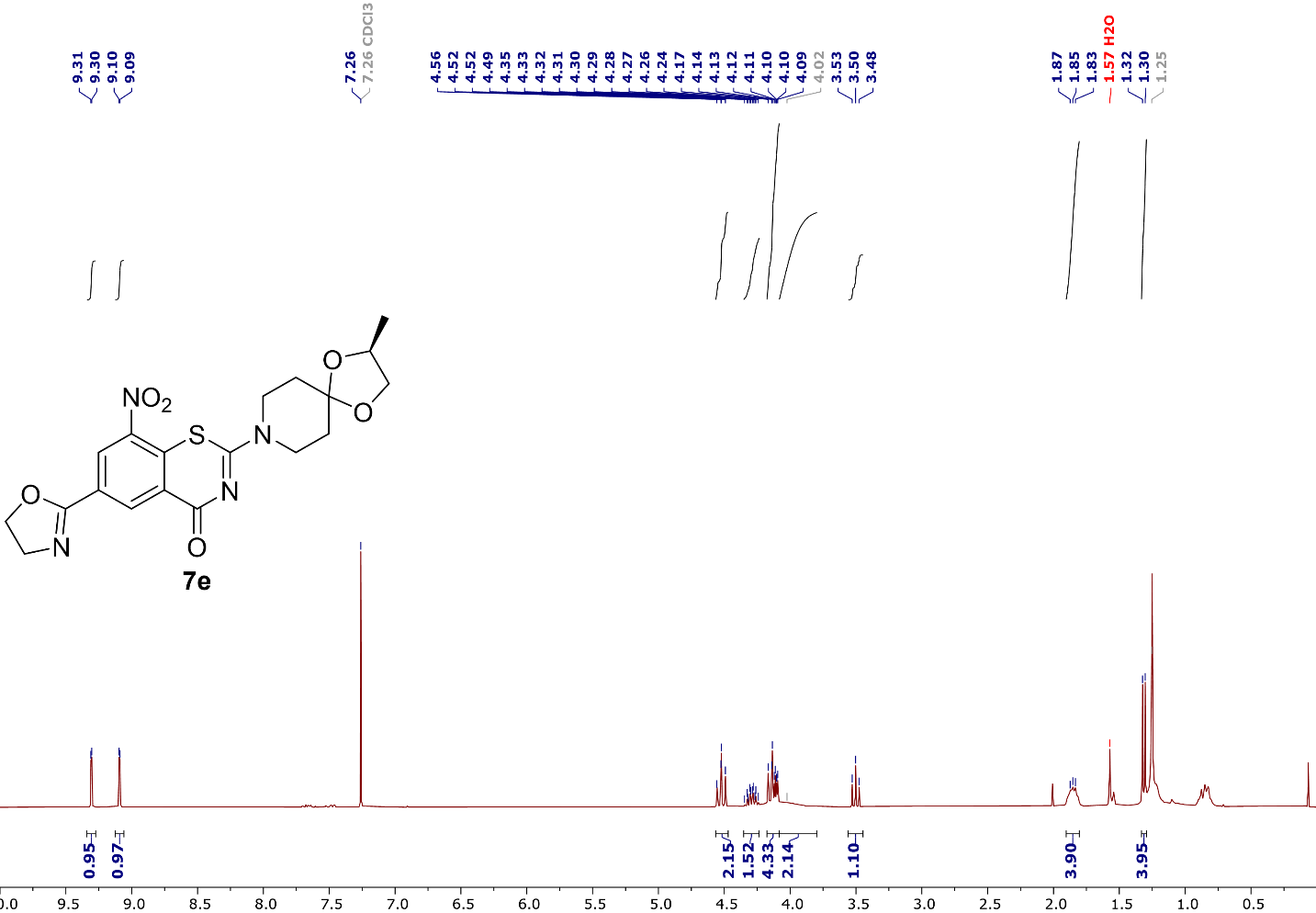


Supporting Figure DS75. ^1^H NMR of compound 7e (300 MHz, 300K, CDCl_3_).


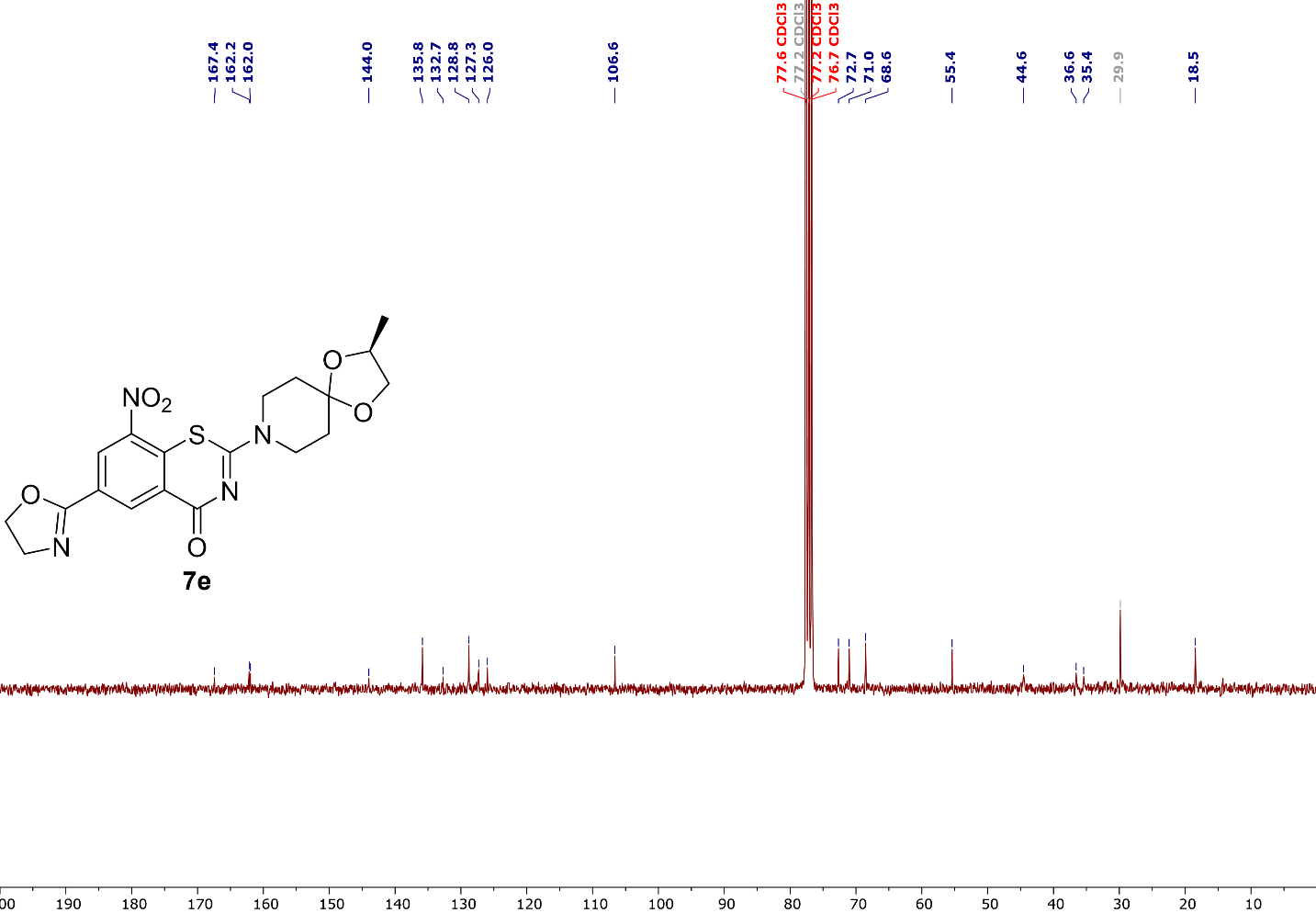


Supporting Figure DS76. ^13^C NMR of compound 7e (75 MHz, 300K, CDCl_3_).


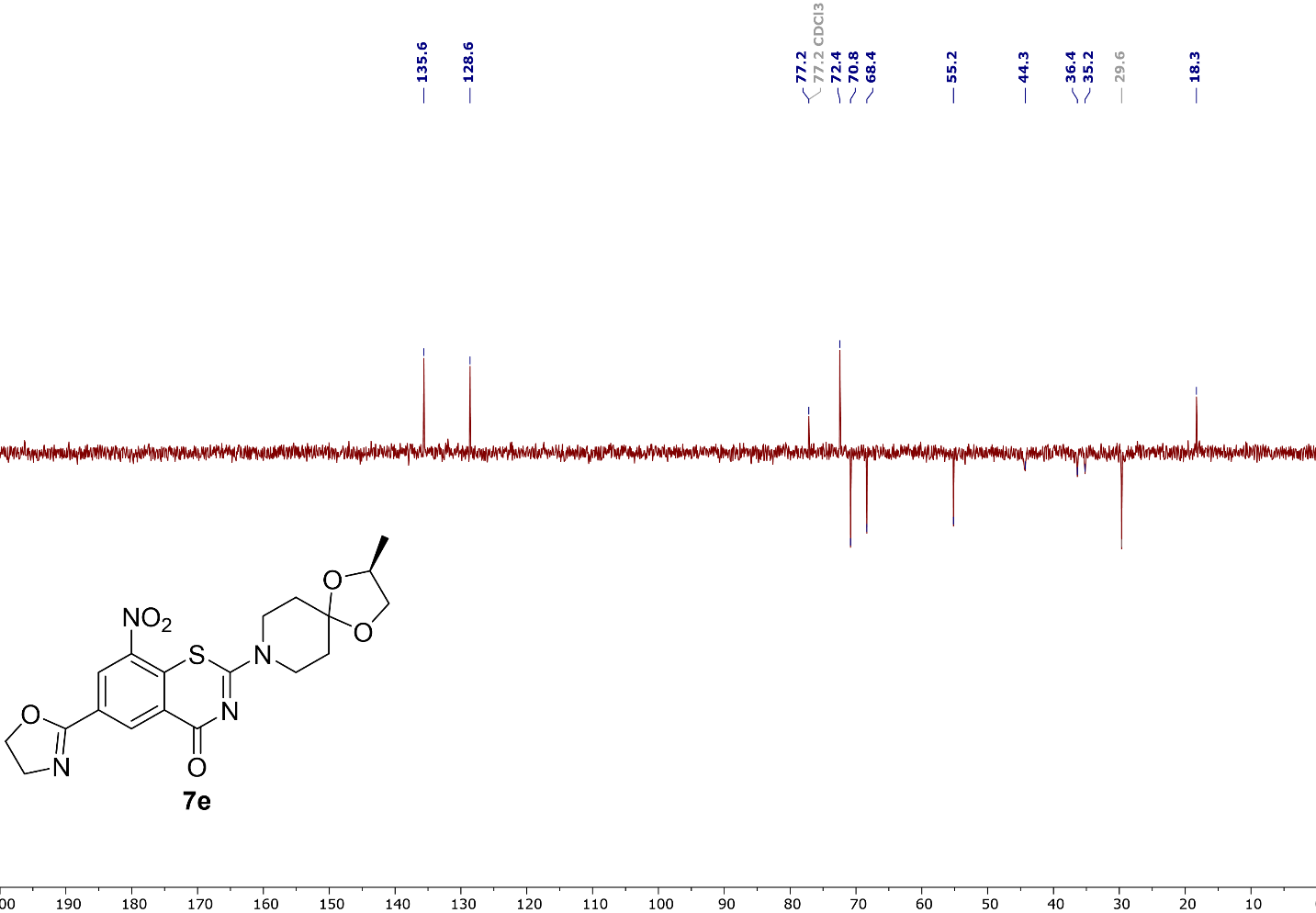


Supporting Figure DS77. DEPT 135 NMR of compound 7e (75 MHz, 300K, CDCl_3_).


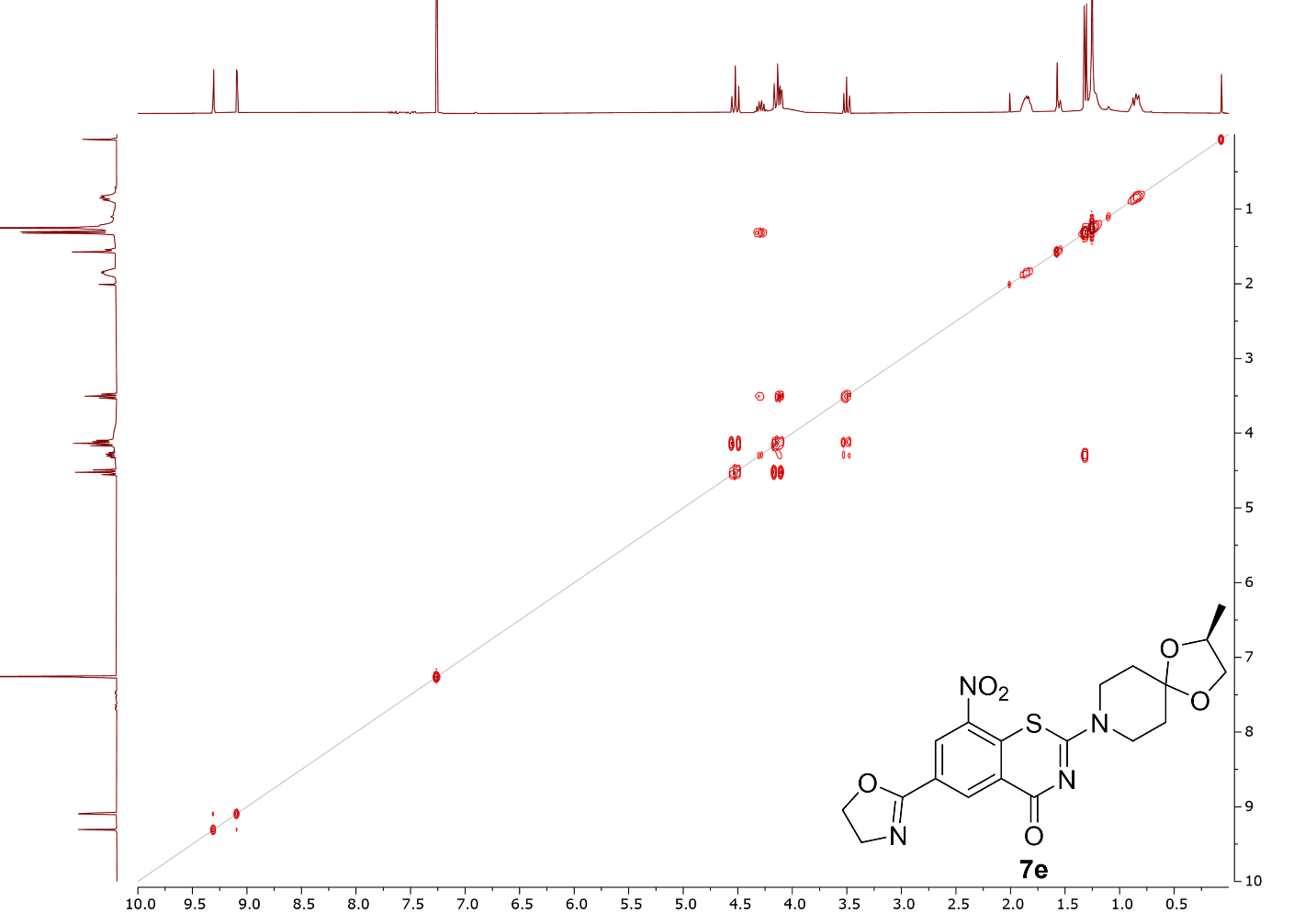


Supporting Figure DS78. COSY experiment of compound 7e (300K, CDCl_3_).


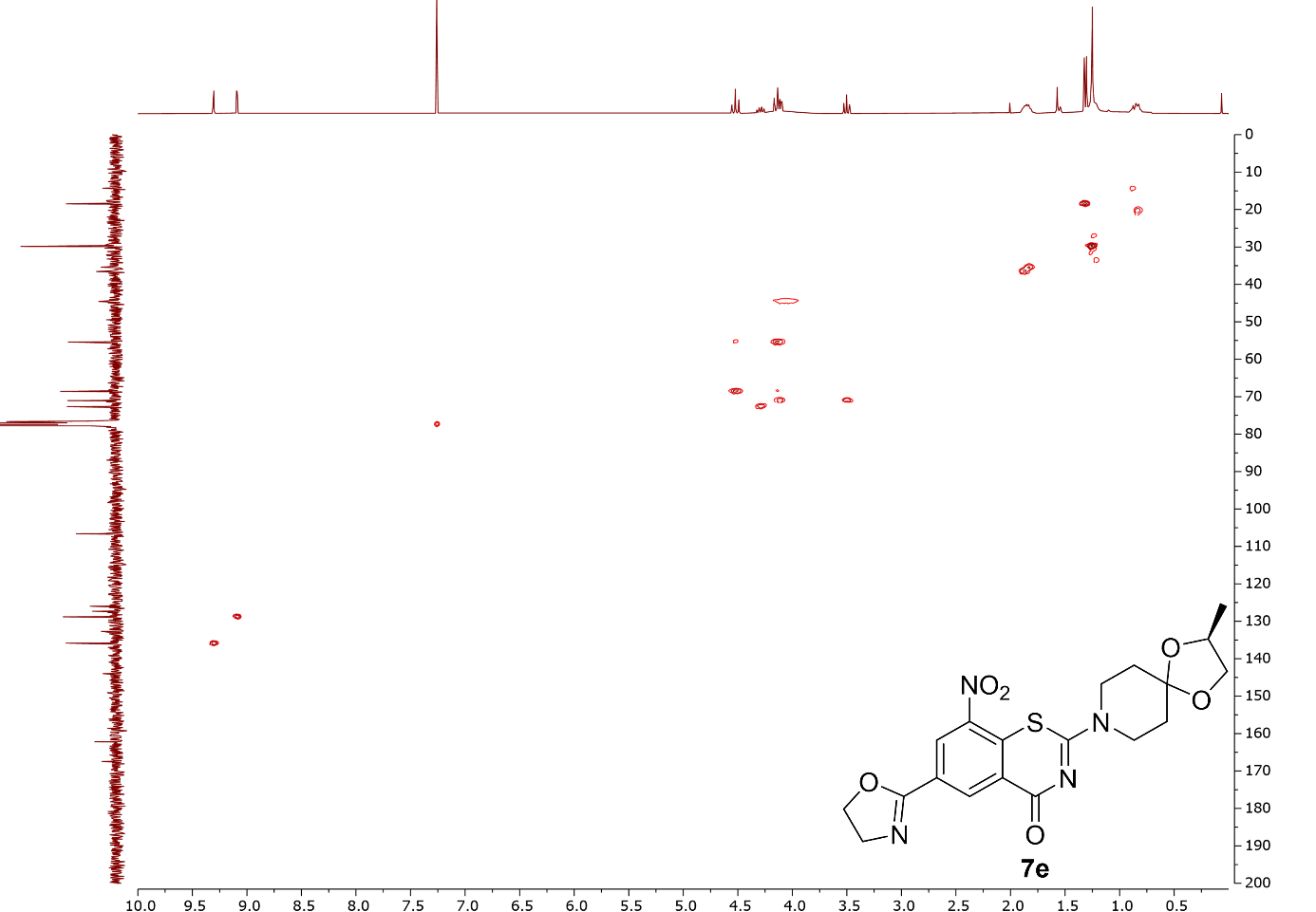


Supporting Figure DS79. HSQC experiment of compound 7e (300K, CDCl_3_).


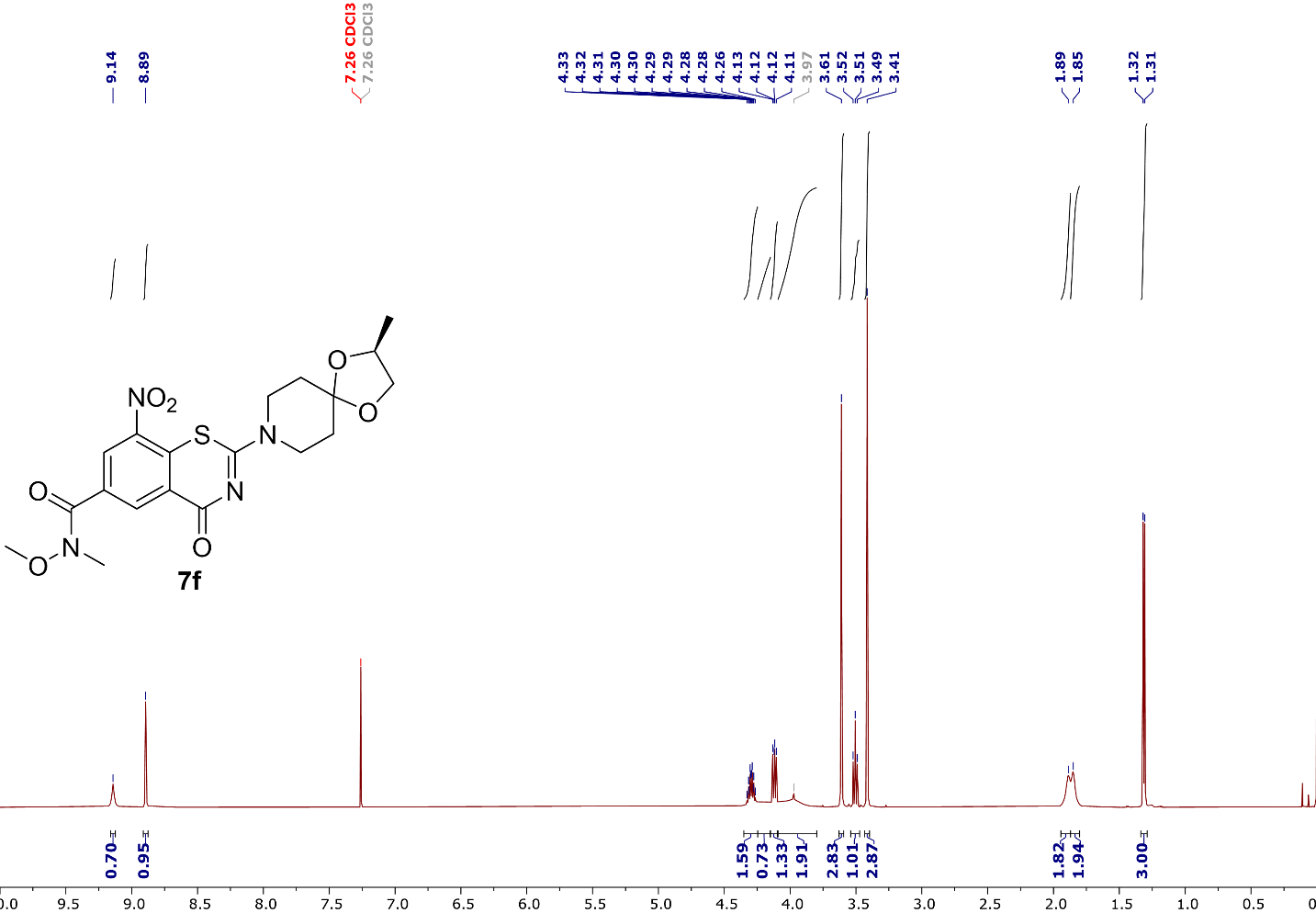


Supporting Figure DS80. ^1^H NMR of compound 7f (500 MHz, 300K, CDCl_3_ + 1% TMS).


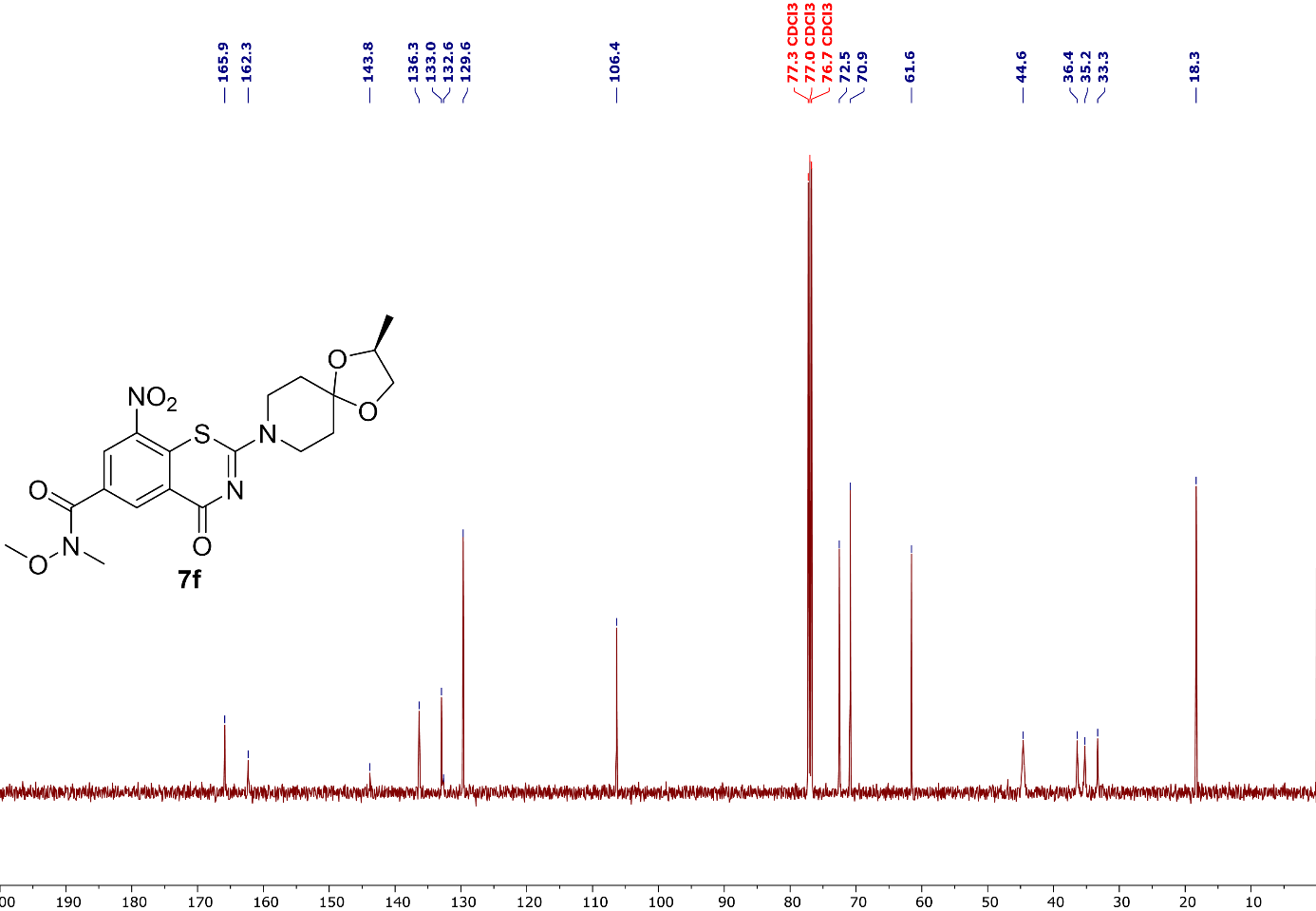


Supporting Figure DS81. ^13^C NMR of compound 7f (126 MHz, 300K, CDCl_3_ + 1% TMS).


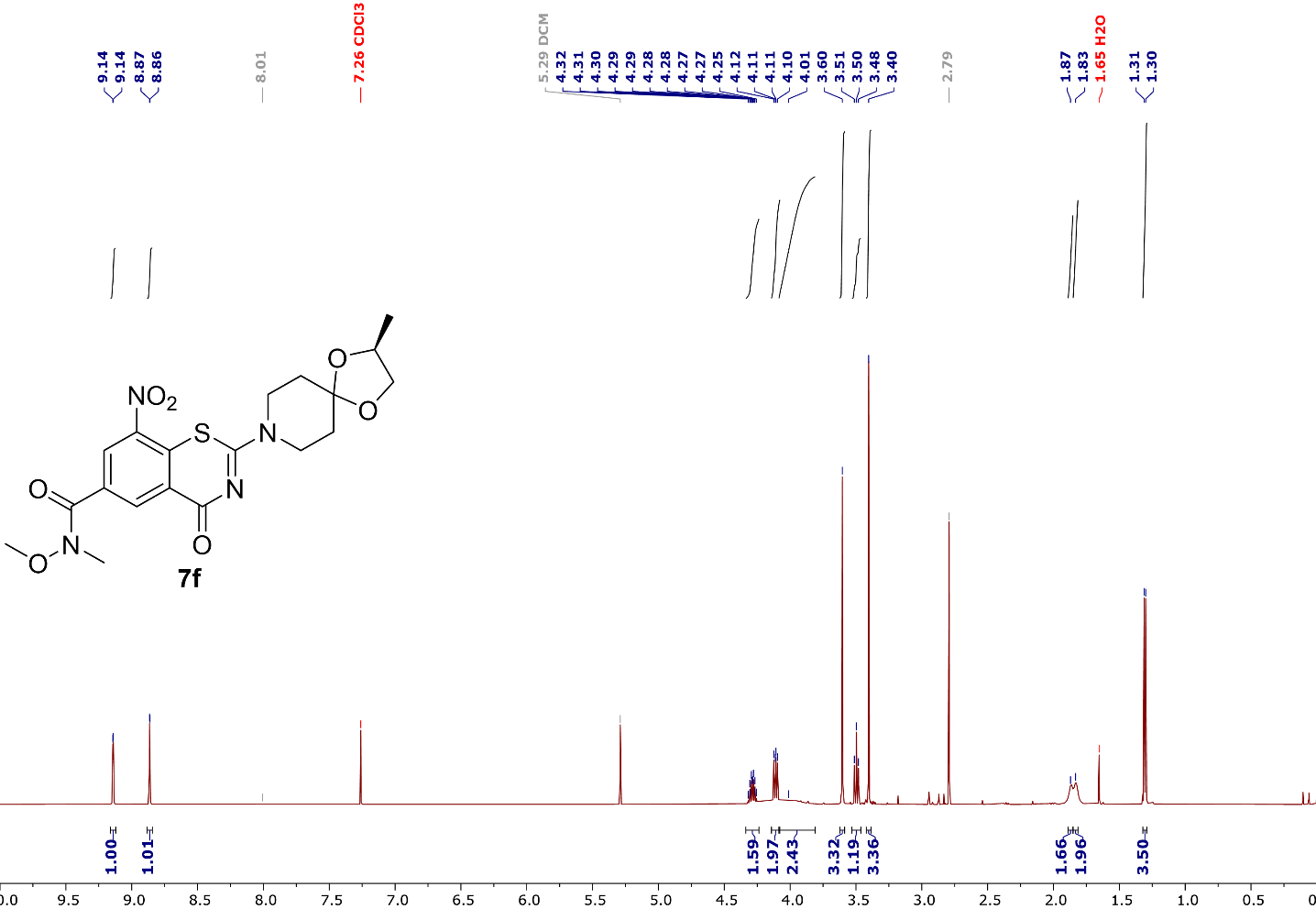


Supporting Figure DS82. ^1^H NMR of unpurified compound 7f (500 MHz, 300K, CDCl_3_ + 1% TMS).


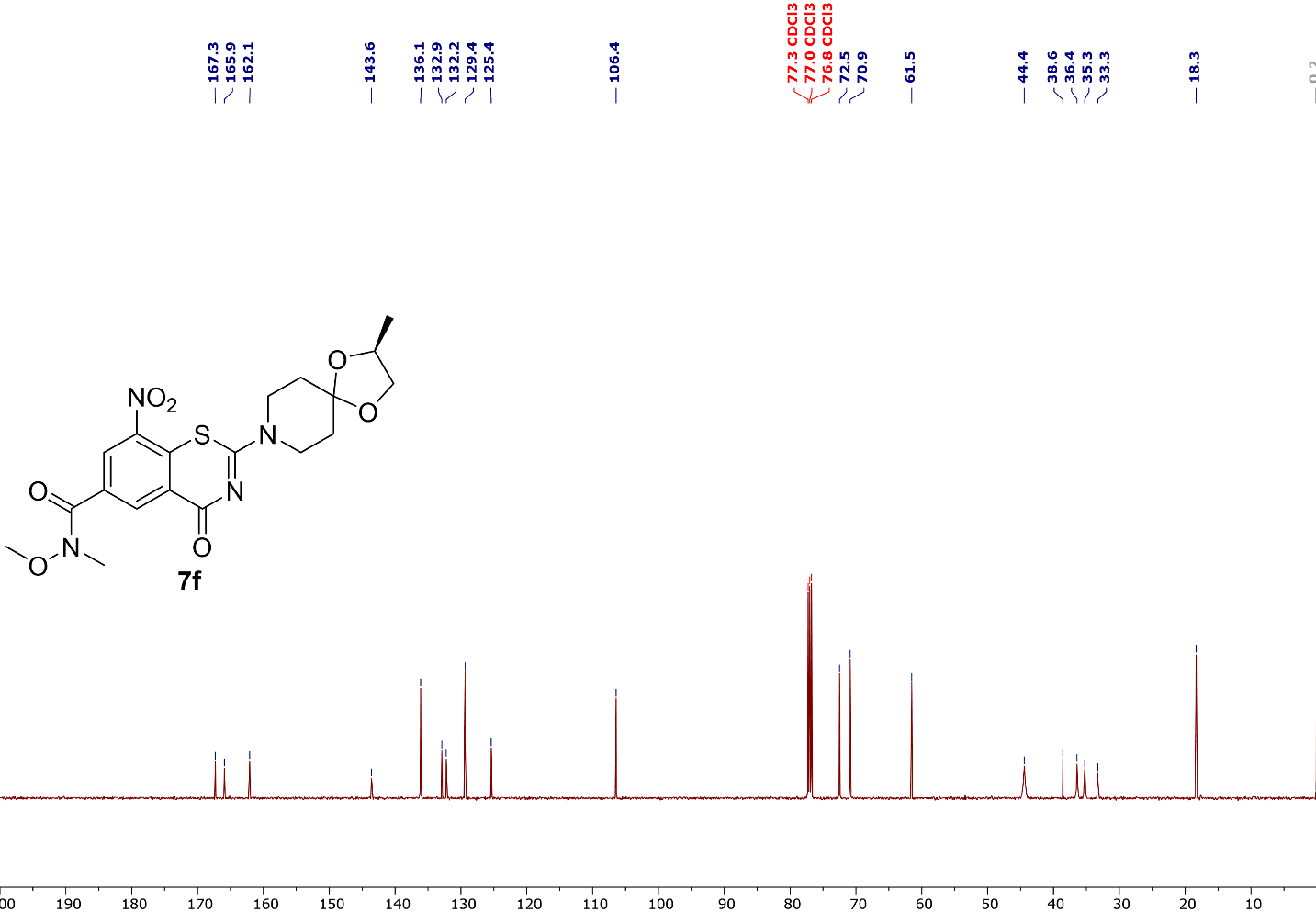


Supporting Figure DS83. ^13^C NMR of unpurified compound 7f (126 MHz, 300K, CDCl_3_ + 1% TMS).
