# Supplementary material for: Modulation of the Meisenheimer complex metabolism of nitro-benzothiazinones by targeted C-6 substitution
Source: Commun Chem. 2024 Jul 6;7:153. doi: 10.1038/s42004-024-01235-x (PMC11227536; doi:10.1038/s42004-024-01235-x)
Supplement: Supplementary file 7 — Supplementary Data 5 [file 42004_2024_1235_MOESM7_ESM.docx]

**Supplementary Information**

**Modulation of the Meisenheimer complex metabolism of nitro-benzothiazinones by targeted C-6 substitution**

François Keiff,^1^ Freddy A. Bernal,^1^ Melanie Joch,^1^ Thibault Joseph William Jacques-dit-Lapierre, ^1^ Yan Li,^1^ Phil Liebing,^2^ Hans-Martin Dahse,^3^ Ivan Vilotijevic,^4^ Florian Kloss*^1^

^1^ Transfer Group Anti-infectives and 3Infection Biology, Leibniz Institute for Natural Products Research and Infection Biology – Leibniz-HKI, Beutenbergstr. 11a, 07745 Jena, Germany.

^2^ Institute for Inorganic and Analytical Chemistry, Friedrich-Schiller-Universität Jena, Humboldtstr. 8, 07743 Jena, Germany.

^3^ Department of Infection Biology, Leibniz Institute for Natural Product Research and Infection Biology – Leibniz-HKI, Beutenbergstr. 11a, 07745 Jena, Germany.

^4^ Institute of Organic Chemistry and Macromolecular Chemistry, Friedrich Schiller University Jena, Humboldtstr. 10, Jena 07743, Germany.

10, Jena 07743, Germany.

**Supplementary Data 5**

# Cartesian Coordinates

# Section A. Calculations at CPCM(water)-B3LYP-D3/6-311+G(d,p)//B3LYP-D3/6-31+G(d,p) level

**Compound 1**

O 0.14845 -0.08197 0.41403

C 1.37546 -0.08471 0.42358

C 2.10855 1.21360 0.16691

C 1.32062 2.29415 -0.23844

C 1.88097 3.54241 -0.49891

C 0.99534 4.67212 -0.96187

F -0.11794 4.77785 -0.19726

F 1.62988 5.86751 -0.92596

F 0.57538 4.48009 -2.23948

C 3.24865 3.72720 -0.35246

C 4.04342 2.65997 0.06071

N 5.47772 2.94384 0.20566

O 5.90395 4.02619 -0.19290

O 6.18162 2.07174 0.72653

C 3.50170 1.37672 0.32693

S 4.54248 0.04201 0.84241

C 3.36044 -1.32955 0.79713

N 3.93185 -2.53748 1.02554

C 5.37574 -2.81741 1.01138

C 5.75384 -3.53318 -0.29279

C 4.92331 -4.81431 -0.46518

O 5.17804 -5.43523 -1.71638

C 6.18892 -6.42156 -1.49374

C 5.85421 -6.93535 -0.08806

C 4.83446 -8.06914 -0.07253

O 5.32408 -5.75446 0.54719

C 3.42788 -4.52057 -0.34690

C 3.11911 -3.76769 0.95368

N 2.07585 -1.24779 0.61945

H 0.25308 2.12470 -0.33040

H 3.71523 4.68431 -0.54567

H 5.95549 -1.90351 1.13523

H 5.59594 -3.46370 1.86764

H 5.56463 -2.87490 -1.14747

H 6.81733 -3.79060 -0.28199

H 6.09790 -7.17927 -2.27517

H 7.19114 -5.97236 -1.53493

H 6.75529 -7.22096 0.46574

H 4.49494 -8.25646 0.95004

H 5.28072 -8.99019 -0.46479

H 3.96660 -7.81292 -0.68823

H 3.11491 -3.92024 -1.20764

H 2.88361 -5.46859 -0.37230

H 2.06842 -3.49427 1.01609

H 3.38706 -4.38689 1.81659

**Compound 6a**

O 0.17729 -0.14766 -0.55866

C 1.40269 -0.13389 -0.52662

C 2.11657 1.17284 -0.27670

C 1.33192 2.23135 0.19024

C 1.87797 3.49034 0.44322

C 0.98122 4.57356 0.94693

O 1.63653 5.74043 1.11877

C 0.83896 6.83969 1.60690

O -0.20249 4.42394 1.17744

C 3.23688 3.69920 0.21334

C 4.02642 2.65554 -0.26050

N 5.44225 2.96644 -0.49098

O 5.89423 4.01753 -0.03837

O 6.10727 2.14851 -1.13711

C 3.49537 1.36325 -0.50728

S 4.53136 0.04683 -1.07949

C 3.40460 -1.35607 -0.86612

N 4.00425 -2.56287 -1.02483

C 3.21580 -3.80004 -0.86780

C 3.51760 -4.44361 0.49142

C 5.02001 -4.69076 0.65107

O 5.27407 -5.19359 1.96460

C 6.05360 -6.38454 1.83718

C 5.64099 -6.91961 0.46773

C 6.65538 -7.79494 -0.24210

O 5.46332 -5.69718 -0.26787

C 5.82823 -3.41785 0.38415

C 5.45291 -2.80864 -0.97464

N 2.12547 -1.29556 -0.65504

H 0.27275 2.05655 0.34466

H 0.02989 7.05926 0.90605

H 1.52577 7.68173 1.68422

H 0.41268 6.59286 2.58230

H 3.69323 4.66305 0.39492

H 2.16087 -3.55513 -0.96853

H 3.51201 -4.47847 -1.67524

H 3.17441 -3.79045 1.30035

H 2.99249 -5.39964 0.58257

H 7.12918 -6.15452 1.86062

H 5.80395 -7.04910 2.66802

H 4.67328 -7.43879 0.54915

H 7.61168 -7.27106 -0.33573

H 6.30244 -8.05348 -1.24431

H 6.81300 -8.72375 0.31650

H 5.61878 -2.69939 1.18348

H 6.89394 -3.66401 0.40883

H 5.69973 -3.50834 -1.78006

H 6.01272 -1.89193 -1.15487

**Compound 6b**

O 0.14387 -0.12166 0.26252

C 1.34212 -0.13069 0.00124

C 2.09601 1.17795 -0.03441

C 1.42997 2.29408 0.48147

C 2.02056 3.55801 0.49441

C 1.25199 4.71074 1.05659

O 1.96265 5.85564 1.01297

C 1.31103 7.06155 1.49906

C 0.44593 7.68292 0.41395

O 0.12303 4.61943 1.49945

C 3.30320 3.71181 -0.02822

C 3.97242 2.61226 -0.55563

N 5.31020 2.87193 -1.10258

O 5.83909 3.95825 -0.87060

O 5.83527 1.97787 -1.77631

C 3.39502 1.31562 -0.56841

S 4.28209 -0.06569 -1.22998

C 3.20806 -1.42145 -0.69116

N 3.74420 -2.65083 -0.90042

C 3.01171 -3.85862 -0.47391

C 3.64895 -4.42213 0.80294

C 5.14317 -4.70288 0.58946

O 5.77920 -5.11940 1.78924

C 5.75658 -6.54875 1.79305

C 5.94369 -6.89214 0.30971

C 7.40360 -6.98448 -0.12190

O 5.28178 -5.79045 -0.34320

C 5.86571 -3.46738 0.05175

C 5.15698 -2.92813 -1.19887

N 2.00987 -1.31488 -0.20328

H 0.42611 2.15928 0.86887

H 0.72501 6.81007 2.38663

H 2.14160 7.71265 1.77949

H -0.37479 7.01273 0.14576

H 1.04067 7.89636 -0.47954

H 0.01845 8.62367 0.77743

H 3.79129 4.67690 -0.03521

H 1.96824 -3.59466 -0.31792

H 3.08693 -4.59165 -1.28440

H 3.53977 -3.70047 1.61932

H 3.14588 -5.34978 1.09255

H 6.57022 -6.89660 2.43336

H 4.79954 -6.92476 2.18050

H 5.40288 -7.80386 0.03283

H 7.47190 -7.03765 -1.21215

H 7.87144 -7.88181 0.29942

H 7.96103 -6.10725 0.22144

H 5.88254 -2.69970 0.83284

H 6.89709 -3.74223 -0.18607

H 5.18499 -3.68076 -1.99408

H 5.66175 -2.03862 -1.57329

**Compound 6c**

O 0.34835 -0.13259 0.82376

C 1.48704 -0.10209 0.37001

C 2.16785 1.23236 0.18368

C 1.35128 2.36517 0.25410

C 1.87241 3.65216 0.11637

C 0.94030 4.81988 0.19502

O 1.59033 5.99201 0.07344

C 0.79388 7.22162 0.12094

C 0.16059 7.46191 -1.24418

C 1.75956 8.31529 0.54877

O -0.26099 4.71011 0.35252

C 3.24291 3.81091 -0.08191

C 4.06764 2.69152 -0.14211

N 5.49958 2.94972 -0.33185

O 5.85250 4.07560 -0.68104

O 6.27974 2.01329 -0.12296

C 3.55562 1.37393 -0.02453

S 4.62471 -0.03267 -0.14623

C 3.39040 -1.35146 -0.28868

N 3.90480 -2.54103 -0.68866

C 5.33278 -2.83605 -0.86682

C 5.85321 -3.67299 0.30991

C 5.02308 -4.94879 0.45905

O 5.48303 -5.66650 1.59444

C 5.41591 -7.05510 1.26102

C 5.77902 -7.06149 -0.22939

C 7.27831 -7.13638 -0.49953

O 5.23855 -5.80358 -0.67843

C 3.52599 -4.62806 0.55286

C 3.07661 -3.76109 -0.63086

N 2.12528 -1.25712 -0.01152

H 0.29057 2.22428 0.43070

H 0.01419 7.07251 0.87422

H 0.93572 7.55983 -2.01155

H -0.42451 8.38722 -1.22286

H -0.50721 6.63925 -1.51005

H 1.22670 9.26781 0.63140

H 2.56241 8.43060 -0.18673

H 2.20721 8.08229 1.51915

H 3.68116 4.79424 -0.18758

H 5.43713 -3.40581 -1.79660

H 5.90840 -1.91833 -0.98404

H 6.89745 -3.95238 0.14512

H 5.79073 -3.09811 1.24004

H 6.12974 -7.58818 1.89281

H 4.40606 -7.45170 1.43512

H 5.25026 -7.85212 -0.77312

H 7.81279 -6.39290 0.10014

H 7.66449 -8.13071 -0.24795

H 7.48025 -6.94324 -1.55694

H 2.95715 -5.56279 0.56657

H 3.33706 -4.09665 1.49159

H 2.03323 -3.46888 -0.54002

H 3.22105 -4.30302 -1.57156

**Compound 6d**

O 0.25769 -0.13991 -0.51067

C 1.43599 -0.09683 -0.17357

C 2.12984 1.24194 -0.10094

C 1.30870 2.37308 -0.13154

C 1.83907 3.66270 -0.08882

C 0.90329 4.82652 -0.12137

O 1.55741 6.00270 -0.06239

C 0.75214 7.20444 -0.11620

C 0.32711 7.54375 -1.53189

O 1.49493 7.88062 -2.27844

O -0.30700 4.72010 -0.19130

C 3.22256 3.82742 -0.03162

C 4.04992 2.70885 -0.01209

N 5.49386 2.96946 0.03136

O 5.87771 4.10288 0.31490

O 6.25023 2.02541 -0.22627

C 3.53079 1.38880 -0.03264

S 4.60909 -0.01390 0.03778

C 3.39639 -1.32522 0.34102

N 3.94868 -2.49955 0.73716

C 5.38761 -2.79482 0.76342

C 5.76957 -3.64624 -0.45543

C 4.92606 -4.93021 -0.49037

O 5.18505 -5.69444 -1.65842

C 6.18277 -6.65809 -1.31264

C 5.83089 -7.00197 0.13978

C 4.79809 -8.11546 0.27717

O 5.30674 -5.75174 0.62774

C 3.43376 -4.61216 -0.41962

C 3.12048 -3.72016 0.78925

N 2.11081 -1.24006 0.17931

H 0.23617 2.22762 -0.19986

H -0.12793 7.07944 0.52060

H 1.40371 7.98452 0.28127

H -0.20537 6.69065 -1.96990

H -0.37008 8.39462 -1.47532

H 1.26493 7.96314 -3.21194

H 3.66691 4.81366 -0.00738

H 5.97261 -1.87643 0.80810

H 5.59153 -3.35489 1.68225

H 5.59169 -3.08322 -1.37789

H 6.83041 -3.91104 -0.41036

H 6.09061 -7.50049 -2.00167

H 7.19037 -6.22701 -1.39561

H 6.72412 -7.22993 0.73182

H 4.44869 -8.17946 1.31151

H 5.23530 -9.08069 -0.00259

H 3.93769 -7.92140 -0.37076

H 3.13364 -4.10490 -1.34260

H 2.87996 -5.55232 -0.34906

H 3.36842 -4.24504 1.71785

H 2.07287 -3.42987 0.80864

**Compound 6e**

O 0.47958 0.07207 1.01174

C 1.61137 0.10326 0.54007

C 2.28881 1.43845 0.34599

C 1.46939 2.56923 0.40750

C 1.98874 3.85594 0.25628

C 1.05386 5.01654 0.31191

O 1.69874 6.19649 0.17230

C 0.88563 7.38684 0.16451

C 0.09968 7.56358 -1.12034

I 1.38005 7.67381 -2.90456

O -0.14898 4.91802 0.46228

C 3.36023 4.01842 0.06145

C 4.18759 2.90024 0.01751

N 5.62198 3.16001 -0.15972

O 5.97762 4.28791 -0.49737

O 6.39963 2.22152 0.05038

C 3.67678 1.58163 0.13746

S 4.74435 0.17525 0.01285

C 3.50741 -1.13952 -0.15023

N 4.01518 -2.32012 -0.58247

C 5.43723 -2.61277 -0.80727

C 5.97384 -3.53365 0.29750

C 5.13694 -4.81150 0.37380

O 5.61672 -5.61315 1.44273

C 5.53173 -6.97287 1.01027

C 5.86250 -6.87118 -0.48444

C 7.35582 -6.92825 -0.79146

O 5.31603 -5.58288 -0.82676

C 3.64595 -4.48438 0.52796

C 3.17616 -3.53428 -0.58166

N 2.24470 -1.04801 0.14162

H 0.40874 2.42618 0.58398

H 0.17536 7.34110 0.99889

H 1.59307 8.20179 0.32315

H -0.58516 6.73548 -1.29143

H -0.45064 8.50476 -1.09417

H 3.79613 5.00151 -0.05770

H 5.52077 -3.11701 -1.77663

H 6.01894 -1.69356 -0.86964

H 7.01236 -3.80671 0.09074

H 5.93524 -3.02560 1.26709

H 6.25449 -7.55567 1.58557

H 4.52242 -7.37500 1.17620

H 5.31949 -7.61846 -1.07356

H 7.53627 -6.65713 -1.83566

H 7.74398 -7.93938 -0.62305

H 7.90550 -6.23259 -0.14947

H 3.06714 -5.41245 0.49072

H 3.48682 -4.01860 1.50633

H 2.13880 -3.23868 -0.44340

H 3.28875 -4.01415 -1.56010

**Compound 7a**

O 0.36773 -0.24178 0.96173

C 1.48568 -0.17676 0.46300

C 2.13034 1.17699 0.28243

C 1.29079 2.28763 0.40324

C 1.77720 3.59281 0.30375

C 0.79695 4.72974 0.42288

N 1.31165 5.94732 0.78652

O -0.39253 4.56907 0.17801

C 3.13751 3.78260 0.07309

C 3.98718 2.68451 -0.05269

N 5.40418 2.98539 -0.28080

O 5.72517 4.14570 -0.54142

O 6.20813 2.05104 -0.18868

C 3.50824 1.35350 0.03389

S 4.59897 -0.02548 -0.17868

C 3.38708 -1.36763 -0.29926

N 3.90751 -2.53445 -0.75521

C 5.33712 -2.80006 -0.96683

C 5.90040 -3.62719 0.19700

C 5.10029 -4.92032 0.36147

O 5.59899 -5.62895 1.48580

C 5.55043 -7.01854 1.15334

C 5.88001 -7.01874 -0.34511

C 7.37370 -7.07030 -0.64900

O 5.31178 -5.76848 -0.78159

C 3.59846 -4.63260 0.48637

C 3.10747 -3.77328 -0.68630

N 2.13389 -1.30886 0.03421

H 0.23552 2.11741 0.58628

H 2.20373 6.03159 1.24908

H 0.64003 6.68680 0.93859

H 3.56928 4.76847 -0.04670

H 5.43027 -3.36682 -1.89929

H 5.89087 -1.87062 -1.09772

H 6.94646 -3.88493 0.00989

H 5.84578 -3.05489 1.12925

H 6.28852 -7.53788 1.76844

H 4.55266 -7.43433 1.35057

H 5.35120 -7.81729 -0.87714

H 7.90954 -6.31765 -0.06203

H 7.54895 -6.87528 -1.71081

H 7.78103 -8.05815 -0.40526

H 3.05058 -5.57955 0.50942

H 3.41634 -4.10681 1.42958

H 2.05946 -3.50562 -0.57540

H 3.24781 -4.30797 -1.63143

**Compound 7b**

O 0.28268 -0.14320 -0.79072

C 1.43378 -0.11204 -0.36876

C 2.12995 1.22089 -0.23407

C 1.31659 2.35694 -0.24535

C 1.85105 3.64571 -0.18736

C 0.89706 4.81185 -0.17209

N 1.36953 5.98736 -0.68070

C 0.51427 7.16556 -0.75276

O -0.23578 4.69687 0.29030

C 3.23435 3.79227 -0.11737

C 4.05860 2.66788 -0.10043

N 5.50191 2.91982 -0.04222

O 5.89161 4.06669 0.18237

O 6.25748 1.96026 -0.23481

C 3.53112 1.35350 -0.13584

S 4.59167 -0.06193 -0.03813

C 3.35378 -1.35419 0.24811

N 3.87781 -2.52411 0.69256

C 5.31017 -2.81569 0.84185

C 5.78679 -3.71674 -0.30530

C 4.94833 -5.00316 -0.35596

O 5.29707 -5.80859 -1.47258

C 6.26752 -6.75387 -1.01632

C 5.80317 -7.04830 0.41541

C 4.76535 -8.16186 0.50987

O 5.24148 -5.78320 0.81752

C 3.45495 -4.68394 -0.41403

C 3.04600 -3.74268 0.72732

N 2.07791 -1.25960 0.02574

H 0.24274 2.22089 -0.30999

H 2.21086 5.97500 -1.23807

H 1.13660 8.04598 -0.92632

H -0.01932 7.27788 0.19273

H -0.22815 7.07872 -1.55483

H 3.70635 4.76435 -0.04343

H 5.89055 -1.89466 0.89165

H 5.44186 -3.33639 1.79673

H 5.68383 -3.19333 -1.26191

H 6.84035 -3.97654 -0.16364

H 6.23164 -7.62063 -1.68009

H 7.27731 -6.32024 -1.03535

H 6.64769 -7.25220 1.08296

H 5.22703 -9.13401 0.30195

H 3.95953 -7.99717 -0.21224

H 4.33405 -8.19043 1.51449

H 3.23029 -4.21656 -1.37869

H 2.89536 -5.62111 -0.34805

H 3.21453 -4.22905 1.69417

H 2.00095 -3.45273 0.64834

**Compound 7c**

O 0.44054 -0.03453 -0.99287

C 1.57216 -0.03514 -0.51951

C 2.23527 1.28561 -0.20326

C 1.43670 2.42614 -0.34153

C 1.91674 3.70284 -0.04874

C 1.03109 4.89458 -0.31082

N 1.01702 5.90766 0.61257

C 0.27502 7.12276 0.28679

C 1.37952 5.76493 2.02024

O 0.39852 4.94528 -1.36557

C 3.24282 3.84121 0.34981

C 4.06346 2.71950 0.45937

N 5.44877 2.97145 0.86839

O 5.74863 4.09803 1.26667

O 6.24909 2.03231 0.79274

C 3.58241 1.41146 0.19870

S 4.64328 0.00334 0.37321

C 3.44606 -1.33344 0.14361

N 3.95215 -2.56542 0.40749

C 5.37043 -2.86386 0.64832

C 6.01323 -3.44530 -0.61892

C 5.23694 -4.67983 -1.08948

O 5.79938 -5.13513 -2.32055

C 6.03544 -6.53927 -2.19999

C 6.29923 -6.71267 -0.70594

C 6.00281 -8.08351 -0.12936

O 5.39130 -5.74912 -0.14537

C 3.74321 -4.38139 -1.23432

C 3.17561 -3.76974 0.05377

N 2.21450 -1.21929 -0.25152

H 0.42131 2.29204 -0.69801

H 0.78197 7.98064 0.73971

H -0.75209 7.07411 0.67232

H 0.23397 7.24535 -0.79471

H 1.70598 4.75074 2.24341

H 0.50645 5.98424 2.64779

H 2.18172 6.46246 2.28938

H 3.66865 4.81343 0.56383

H 5.90350 -1.97791 0.99300

H 5.41580 -3.60293 1.45543

H 6.01531 -2.69934 -1.42074

H 7.04983 -3.73544 -0.42093

H 6.88519 -6.80006 -2.83548

H 5.15152 -7.11492 -2.51238

H 7.33395 -6.41351 -0.47619

H 6.67913 -8.83067 -0.55838

H 4.97018 -8.37354 -0.34691

H 6.14000 -8.08004 0.95554

H 3.60488 -3.68170 -2.06501

H 3.21936 -5.31104 -1.47596

H 3.27061 -4.47785 0.88370

H 2.13164 -3.48914 -0.06347

**Compound 7d**

O 0.37027 -0.11238 0.80967

C 1.52511 -0.06657 0.40090

C 2.23236 1.26673 0.34960

C 1.42834 2.40401 0.46240

C 1.97002 3.69134 0.47549

C 1.02431 4.85916 0.57691

N 1.53059 6.01917 1.09253

C 0.68331 7.19997 1.27015

C 0.51614 7.98380 -0.03252

O 1.76148 8.47727 -0.52332

O -0.14219 4.76221 0.20281

C 3.35060 3.83398 0.36145

C 4.16549 2.70897 0.24331

N 5.60598 2.96146 0.13766

O 6.36590 1.99664 0.27494

O 5.98704 4.11417 -0.07205

C 3.63176 1.39693 0.22248

S 4.68319 -0.01477 0.02764

C 3.43496 -1.29167 -0.28234

N 3.94629 -2.45053 -0.76691

C 5.37661 -2.75890 -0.90023

C 5.82813 -3.68579 0.23676

C 4.97200 -4.95302 0.25615

O 5.36582 -5.75671 1.35818

C 5.29306 -7.11702 0.92397

C 5.72983 -7.02527 -0.54365

C 7.23943 -7.10625 -0.74518

O 5.22839 -5.72985 -0.92770

C 3.47684 -4.61265 0.30173

C 3.09837 -3.65711 -0.83770

N 2.16131 -1.19772 -0.04754

H 0.35591 2.26952 0.55004

H 2.43601 6.01139 1.53667

H -0.29862 6.88100 1.63652

H 1.15799 7.83616 2.02174

H 0.01173 7.35571 -0.77755

H -0.10870 8.86238 0.15527

H 2.26897 7.73098 -0.86809

H 3.83160 4.80403 0.33116

H 5.51434 -3.26432 -1.86211

H 5.97063 -1.84572 -0.92764

H 6.87444 -3.97272 0.10089

H 5.73085 -3.17692 1.20173

H 5.96536 -7.70711 1.55047

H 4.26937 -7.50478 1.01927

H 5.21813 -7.76639 -1.16748

H 7.49670 -6.84227 -1.77490

H 7.59866 -8.12255 -0.54715

H 7.75382 -6.41688 -0.06830

H 2.89487 -5.53586 0.22248

H 3.25024 -4.14478 1.26557

H 2.05598 -3.35403 -0.77741

H 3.28296 -4.13275 -1.80653

**Compound 7e**

O 0.47780 0.20913 1.76079

C 1.60455 0.01876 1.31745

C 2.31583 1.14412 0.61038

C 1.52864 2.22807 0.21567

C 2.08163 3.33387 -0.43578

C 1.22455 4.45879 -0.84513

N -0.04042 4.52551 -0.69192

C -0.46881 5.81933 -1.24089

O 1.86752 5.51005 -1.44026

C 3.45346 3.35617 -0.68027

C 4.24818 2.28528 -0.27777

N 5.68205 2.39921 -0.55675

O 6.06069 3.26939 -1.33992

O 6.44010 1.60941 0.01916

C 3.70500 1.14600 0.36354

S 4.73265 -0.21848 0.83366

C 3.45899 -1.45001 1.20453

N 3.94698 -2.70726 1.36518

C 3.02602 -3.81385 1.68307

C 2.76034 -4.64982 0.42546

C 4.07396 -5.15314 -0.16941

O 3.80060 -5.86005 -1.36987

C 4.69971 -6.97002 -1.41471

C 4.81845 -7.36551 0.06157

C 3.73637 -8.33362 0.52846

O 4.69301 -6.09388 0.72658

C 5.05431 -3.99672 -0.40870

C 5.24998 -3.18066 0.87676

N 2.19644 -1.22007 1.39766

C 0.83566 6.48406 -1.75954

H 0.46759 2.19831 0.43656

H -1.20397 5.65621 -2.03621

H -0.95815 6.40643 -0.45619

H 3.91685 4.19791 -1.17766

H 3.50973 -4.43240 2.44670

H 2.10532 -3.39619 2.08388

H 2.13408 -5.51276 0.66768

H 2.23837 -4.04254 -0.32123

H 4.25209 -7.74181 -2.04494

H 5.67018 -6.67228 -1.83599

H 5.81379 -7.75621 0.29997

H 3.75684 -8.42332 1.61822

H 2.74732 -7.97582 0.22601

H 3.89622 -9.32721 0.09452

H 4.65164 -3.35403 -1.19884

H 6.01407 -4.40051 -0.74548

H 5.92988 -2.34735 0.70572

H 5.68906 -3.81192 1.65621

H 0.84956 6.64342 -2.84094

H 1.08832 7.41764 -1.25039

**Compound 7f**

O 0.38304 -0.07610 1.08850

C 1.48399 -0.07304 0.54490

C 2.14904 1.24609 0.24311

C 1.33048 2.38146 0.30760

C 1.83575 3.66339 0.08552

C 1.03189 4.93941 0.04916

N 0.00280 5.11635 0.93936

O -0.46628 4.03250 1.69114

C 0.06488 4.06486 3.02717

C -0.95913 6.20150 0.83211

O 1.34945 5.84192 -0.72590

C 3.19325 3.79887 -0.21565

C 4.02004 2.68197 -0.25676

N 5.44081 2.92958 -0.53082

O 5.77856 4.04094 -0.93324

O 6.22808 1.99600 -0.33131

C 3.52044 1.37156 -0.04689

S 4.57962 -0.04445 -0.16319

C 3.33441 -1.35854 -0.18367

N 3.81422 -2.56495 -0.57721

C 5.22539 -2.87455 -0.84318

C 5.82444 -3.67014 0.32463

C 5.00312 -4.93512 0.57800

O 5.53844 -5.61384 1.70455

C 5.44788 -7.01314 1.42635

C 5.70702 -7.07388 -0.08436

C 7.18404 -7.16132 -0.45475

O 5.13578 -5.83193 -0.53951

C 3.51731 -4.60323 0.76363

C 2.98868 -3.77726 -0.41677

N 2.09008 -1.24750 0.17466

H 0.28794 2.22094 0.54454

H -0.38437 3.20664 3.53034

H -0.21755 4.99260 3.53735

H 1.15514 3.96077 3.01119

H -0.50552 6.99987 0.24621

H -1.20319 6.56775 1.83268

H -1.87369 5.85014 0.34119

H 3.60854 4.77644 -0.42705

H 5.26205 -3.47933 -1.75580

H 5.79355 -1.96490 -1.03520

H 6.85391 -3.96089 0.09817

H 5.82855 -3.06118 1.23498

H 6.20350 -7.52561 2.02584

H 4.45229 -7.40049 1.68376

H 5.14222 -7.88234 -0.56158

H 7.58678 -8.14725 -0.19609

H 7.75874 -6.39883 0.08040

H 7.31239 -7.00538 -1.52963

H 2.94832 -5.53389 0.85112

H 3.39642 -4.03699 1.69320

H 1.95507 -3.47637 -0.26356

H 3.06453 -4.35407 -1.34466

**Compound 8**

O 0.12120 -0.12530 -0.44210

C 1.34740 -0.14950 -0.45460

C 2.14280 1.06530 -0.08370

C 1.48840 2.06870 0.65410

C 2.13070 3.24150 1.04660

C 1.39760 4.33870 1.87070

O 0.23330 4.03720 2.22720

O 2.07360 5.37890 2.07120

C 3.46230 3.41630 0.67620

C 4.13110 2.43650 -0.05970

N 5.51770 2.70610 -0.42010

O 6.12310 3.60770 0.16210

O 6.03280 2.00920 -1.31290

C 3.49250 1.23120 -0.44770

S 4.37530 -0.02560 -1.34810

C 3.24520 -1.40420 -1.11860

N 3.77700 -2.62330 -1.47380

C 2.96110 -3.82850 -1.26170

C 3.25150 -4.42990 0.12130

C 4.74610 -4.71050 0.28730

O 4.99350 -5.15340 1.62960

C 5.72800 -6.37260 1.56550

C 5.28880 -6.96730 0.22900

C 6.26520 -7.92400 -0.42840

O 5.15670 -5.78820 -0.57390

C 5.59470 -3.48400 -0.05070

C 5.21470 -2.92000 -1.42940

N 2.00930 -1.33770 -0.74530

H 0.44830 1.93080 0.92930

H 3.96330 4.33340 0.96390

H 1.91050 -3.55990 -1.35630

H 3.22830 -4.54720 -2.04470

H 2.93210 -3.72920 0.89930

H 2.70070 -5.36640 0.26020

H 6.81260 -6.18290 1.57040

H 5.46110 -6.98340 2.43250

H 4.30290 -7.44600 0.34550

H 6.38750 -8.82410 0.18460

H 7.24130 -7.44470 -0.55380

H 5.89940 -8.22620 -1.41390

H 5.42030 -2.72160 0.71580

H 6.65200 -3.76720 -0.02430

H 5.42430 -3.66200 -2.20740

H 5.80180 -2.03130 -1.65660

**Compound 9**

O 0.14313 0.21442 -0.18953

C 1.35698 0.19829 -0.01702

N 2.04944 1.34989 0.25478

C 3.28591 1.40312 0.64944

N 3.85122 2.60193 0.93005

C 3.09624 3.84581 0.67745

C 3.60268 4.49804 -0.61488

C 5.11408 4.75151 -0.53534

O 5.63008 5.26384 -1.75422

C 5.63445 6.68900 -1.63463

C 5.97316 6.90209 -0.15372

C 7.46943 6.93955 0.13880

O 5.35775 5.75492 0.46575

C 5.87187 3.47217 -0.17638

C 5.29235 2.84475 1.09914

S 4.37935 -0.00556 0.97510

C 2.09811 -1.11080 -0.17128

C 3.42976 -1.31037 0.25051

C 3.98473 -2.60312 0.06711

N 5.35581 -2.92597 0.48872

O 5.85157 -3.98404 0.10669

O 5.93319 -2.10852 1.21280

C 3.26884 -3.63946 -0.52205

C 1.95256 -3.41975 -0.93718

C 1.20280 -4.48337 -1.53984

N 0.59789 -5.34697 -2.03011

C 1.37815 -2.15763 -0.75119

H 5.47892 7.79128 0.25265

H 2.03758 3.60456 0.61730

H 3.27197 4.51228 1.52820

H 3.39732 3.84057 -1.46613

H 3.08582 5.44808 -0.78064

H 6.38685 7.08227 -2.32159

H 4.65110 7.10650 -1.89129

H 7.91288 7.86373 -0.24884

H 7.97287 6.08827 -0.33017

H 7.64337 6.89777 1.21775

H 5.79368 2.76685 -1.01074

H 6.92676 3.72016 -0.03081

H 5.41293 3.53460 1.94059

H 5.81868 1.92556 1.35309

H 3.74589 -4.60325 -0.64742

H 0.35274 -1.96029 -1.04395

**Compound 10**

O 0.24665 -0.17136 -0.37365

C 1.43622 -0.12571 -0.07391

C 2.14160 1.21155 -0.07831

C 1.31525 2.33670 -0.19949

C 1.85136 3.61964 -0.22620

I 0.57316 5.31349 -0.39939

C 3.22714 3.79705 -0.14662

C 4.05495 2.67805 -0.03697

N 5.49695 2.95150 0.02537

O 5.86958 4.11502 0.17246

O 6.26422 1.98758 -0.07846

C 3.54041 1.35894 0.01219

S 4.61605 -0.03862 0.18771

C 3.39739 -1.34370 0.47299

N 3.94022 -2.51775 0.88228

C 5.37764 -2.81278 0.95616

C 5.78923 -3.71006 -0.21968

C 4.94480 -4.99406 -0.22885

O 5.23445 -5.80227 -1.35940

C 6.21640 -6.75615 -0.94868

C 5.81881 -7.04398 0.50444

C 4.77201 -8.14399 0.65074

O 5.29212 -5.77240 0.92943

C 3.45176 -4.67030 -0.21240

C 3.10748 -3.73429 0.95425

N 2.11173 -1.25994 0.29751

H 0.24889 2.15827 -0.27947

H 3.68440 4.77779 -0.16837

H 5.96390 -1.89436 0.97918

H 5.55836 -3.33754 1.90070

H 5.63645 -3.18173 -1.16686

H 6.84804 -3.97395 -0.13667

H 7.22832 -6.33221 -1.01679

H 6.14096 -7.62326 -1.60873

H 6.69179 -7.25690 1.13139

H 3.93376 -7.96772 -0.03068

H 4.39030 -8.16660 1.67552

H 5.20898 -9.12258 0.42078

H 3.17957 -4.19685 -1.16175

H 2.89290 -5.60600 -0.12273

H 3.32755 -4.22671 1.90766

H 2.06061 -3.44082 0.93405

**Compound 11**

O 0.36251 -0.25476 0.86129

C 1.48932 -0.17676 0.38057

C 2.13214 1.18483 0.24135

C 1.29141 2.28106 0.47305

C 1.78037 3.57640 0.38247

Br 0.59249 5.08803 0.70302

C 3.11159 3.81058 0.07410

C 3.95388 2.71955 -0.14591

N 5.35362 3.04399 -0.45212

O 5.64829 4.21649 -0.68147

O 6.16563 2.11206 -0.45837

C 3.49087 1.38167 -0.08042

S 4.58045 0.02011 -0.39694

C 3.38881 -1.33904 -0.43764

N 3.90768 -2.50617 -0.89441

C 5.32952 -2.75300 -1.17157

C 5.95765 -3.56914 -0.03312

C 5.18430 -4.87325 0.17096

O 5.74509 -5.57281 1.27095

C 5.70357 -6.96319 0.94195

C 5.96401 -6.95900 -0.57019

C 7.44352 -6.98181 -0.94178

O 5.35143 -5.72088 -0.97956

C 3.68619 -4.60461 0.36656

C 3.12861 -3.75505 -0.78369

N 2.14572 -1.29761 -0.05799

H 0.25915 2.07545 0.73230

H 3.51727 4.81123 0.00266

H 5.38732 -3.32104 -2.10640

H 5.86497 -1.81700 -1.32956

H 6.99747 -3.81235 -0.26863

H 5.93864 -2.99463 0.89919

H 6.47697 -7.47043 1.52307

H 4.72227 -7.39448 1.18443

H 5.42702 -7.76801 -1.07785

H 7.88104 -7.96123 -0.71663

H 7.99080 -6.21828 -0.37985

H 7.56653 -6.78453 -2.01064

H 3.15230 -5.55858 0.41733

H 3.54253 -4.07894 1.31654

H 2.08355 -3.50048 -0.62397

H 3.23125 -4.29178 -1.73278

**Compound 23**

O 2.15205 0.41659 3.24497

C 2.55674 0.25735 2.09597

C 2.85613 1.47214 1.25067

C 2.42754 2.70505 1.76108

C 2.66079 3.89703 1.08317

C 3.34837 3.86874 -0.12382

C 3.78992 2.65083 -0.64242

N 4.52409 2.71167 -1.91087

O 5.08406 1.67937 -2.29946

O 4.55387 3.77914 -2.52370

C 3.54461 1.42164 0.02140

S 4.08150 -0.11516 -0.68231

C 3.26532 -1.27918 0.43471

N 3.30792 -2.56615 0.00316

C 2.92123 -3.66240 0.91178

C 4.17990 -4.35997 1.44625

C 5.07617 -4.83758 0.30054

O 6.28220 -5.37522 0.84671

C 6.49898 -6.65667 0.25349

C 5.08155 -7.13430 -0.05417

C 4.94544 -8.13681 -1.18344

O 4.44215 -5.90081 -0.42449

C 5.37656 -3.70517 -0.68630

C 4.07302 -3.04476 -1.15662

N 2.70164 -1.00370 1.57288

H 1.91229 2.68961 2.71529

H 2.31905 4.84128 1.49283

H 3.55851 4.77312 -0.68101

H 2.32095 -3.25054 1.71962

H 2.31605 -4.36899 0.33371

H 4.74901 -3.66884 2.07688

H 3.90209 -5.22443 2.05749

H 7.09262 -6.56825 -0.66869

H 7.03257 -7.28216 0.97341

H 4.61513 -7.52306 0.86450

H 5.44229 -9.07642 -0.91850

H 3.89131 -8.35040 -1.38068

H 5.39703 -7.74354 -2.09961

H 6.01049 -2.96595 -0.18565

H 5.92684 -4.11462 -1.53873

H 3.45190 -3.78072 -1.67844

H 4.26822 -2.23290 -1.85706

#

# Section B. Calculations at CPCM(water)-PBE0-D3BJ/ma-def2-TZVP//PBE0-D3BJ/ma-def2-SVP level

**Compound 1**

O -1.59361 2.39744 1.82988

C -1.13815 1.58962 1.02410

C -2.03638 0.51003 0.50055

C -3.39304 0.62705 0.80025

C -4.31376 -0.32396 0.37129

C -5.77242 -0.14410 0.69532

F -6.28769 0.92006 0.05975

F -5.96403 0.06165 2.00553

F -6.50292 -1.20519 0.34630

C -3.87731 -1.41532 -0.36276

C -2.52439 -1.54642 -0.65630

N -2.14631 -2.72271 -1.42289

O -0.96057 -2.99161 -1.49257

O -3.02104 -3.37576 -1.95041

C -1.56836 -0.59086 -0.24268

S 0.12141 -0.77477 -0.65863

C 0.75827 0.81554 -0.15406

N 2.00768 1.04631 -0.56921

C 2.75349 2.21451 -0.11064

C 3.88779 1.77142 0.80447

C 4.78936 0.74949 0.12702

O 5.51429 1.39901 -0.90261

C 6.81693 0.85138 -0.90094

C 7.06113 0.58802 0.57560

C 8.04178 -0.51290 0.89067

O 5.75237 0.24085 1.02946

C 3.96860 -0.40736 -0.44141

C 2.83623 0.11020 -1.31750

N 0.13767 1.69168 0.58666

H -3.70475 1.49235 1.38887

H -4.56730 -2.18222 -0.71378

H 3.14907 2.72694 -1.00160

H 2.06718 2.89227 0.40457

H 3.46912 1.31364 1.71399

H 4.49281 2.63992 1.10369

H 7.51013 1.58090 -1.34045

H 6.85754 -0.08846 -1.48290

H 7.37164 1.52737 1.07050

H 9.04445 -0.23548 0.53115

H 8.10509 -0.68055 1.97562

H 7.74165 -1.45347 0.40400

H 4.62453 -1.06615 -1.02920

H 3.55939 -0.99502 0.39502

H 3.24303 0.65243 -2.18572

H 2.23617 -0.71662 -1.71519

**Compound 1 HMC**

O -1.36969 3.00682 -0.69186

C -0.98547 1.89825 -0.28368

C -1.94190 0.79184 -0.22580

C -3.34863 1.18743 -0.59523

C -4.34418 0.08988 -0.39316

C -5.77817 0.44395 -0.56880

F -6.60703 -0.60151 -0.46310

F -6.19583 1.35754 0.33906

F -6.01524 1.00699 -1.77489

C -3.98391 -1.16681 -0.07505

C -2.61190 -1.53526 0.13006

N -2.33422 -2.85162 0.35879

O -1.14379 -3.21612 0.52902

O -3.25529 -3.69897 0.39422

C -1.60105 -0.49814 0.10217

S 0.04616 -0.94485 0.54666

C 0.84364 0.64027 0.52274

N 2.12279 0.61761 0.95155

C 2.99453 1.77138 0.78027

C 4.04570 1.47897 -0.28437

C 4.82331 0.20670 0.03906

O 5.64159 0.39995 1.18270

C 6.95344 0.65395 0.72462

C 7.03303 -0.19672 -0.53102

C 8.02405 0.25662 -1.57247

O 5.70094 -0.09007 -1.03005

C 3.87943 -0.95591 0.31281

C 2.84254 -0.57669 1.36221

N 0.31392 1.75596 0.11365

H -3.36753 1.53534 -1.64633

H -3.63052 2.08324 -0.01334

H -4.72848 -1.95336 0.04246

H 2.38400 2.63774 0.50942

H 3.47907 1.97757 1.74839

H 3.56018 1.35330 -1.26491

H 4.75037 2.32089 -0.36130

H 7.66726 0.35912 1.50559

H 7.09428 1.72487 0.48509

H 7.22958 -1.24896 -0.25003

H 9.04736 0.18266 -1.17419

H 7.83382 1.30122 -1.86227

H 7.96281 -0.37592 -2.46993

H 4.46620 -1.82399 0.64780

H 3.37456 -1.22166 -0.62895

H 2.15040 -1.40633 1.54837

H 3.33695 -0.35676 2.32249

**Compound 1 TS**

O -2.29814 -2.32642 -0.46092

C -1.35573 -1.53011 -0.42906

C -0.00993 -2.02535 -0.06456

C 0.08598 -3.43313 0.15574

C 1.26957 -3.94875 0.75488

C 1.26391 -5.35151 1.27240

F 0.44546 -5.48700 2.33637

F 2.47418 -5.76401 1.66291

F 0.82260 -6.22894 0.35453

C 2.39305 -3.16819 0.82297

C 2.35864 -1.82815 0.39995

N 3.57989 -1.10737 0.45997

O 3.57468 0.07876 0.13696

O 4.59914 -1.68191 0.82150

C 1.13047 -1.22624 -0.00600

S 1.07639 0.48859 -0.37186

C -0.65444 0.70587 -0.74050

N -0.97707 1.96457 -1.07668

C -2.34696 2.32818 -1.42076

C -2.90405 3.30295 -0.39355

C -2.00861 4.52874 -0.25239

O -2.08400 5.28949 -1.45384

C -2.38021 6.62889 -1.11102

C -3.13991 6.47957 0.19456

C -3.11456 7.66763 1.11995

O -2.45140 5.37767 0.78197

C -0.56894 4.11330 0.02093

C -0.08309 3.11221 -1.01988

N -1.56919 -0.22117 -0.72729

H -0.86212 -3.95497 0.29700

H 3.32932 -3.55490 1.22462

H -2.32818 2.79344 -2.41994

H -2.94954 1.41733 -1.47352

H -2.97047 2.80797 0.58770

H -3.91442 3.62290 -0.68833

H -2.97220 7.08336 -1.91754

H -1.45429 7.21522 -0.96132

H -4.18501 6.18433 -0.01979

H -3.62889 8.51934 0.65007

H -3.63126 7.43705 2.06288

H -2.07891 7.96368 1.34644

H 0.07130 5.00777 0.00620

H -0.51380 3.66633 1.02580

H -0.06758 3.57877 -2.01822

H 0.94432 2.80233 -0.79719

B 0.46371 -4.03132 -2.56481

H 0.06580 -4.05738 -1.32637

H 1.68406 -3.88747 -2.47382

H -0.11599 -3.04974 -3.03348

H 0.11975 -5.10952 -3.05417

**Compound 1 radical anion**

O 1.10342 2.86820 -1.44345

C 0.81281 1.94659 -0.69154

C 1.83028 0.90364 -0.36956

C 3.07357 1.03782 -1.02318

C 4.08403 0.10563 -0.79061

C 5.38722 0.20630 -1.52433

F 5.45188 -0.64835 -2.56784

F 5.60401 1.43072 -2.02813

F 6.44398 -0.08555 -0.74573

C 3.88968 -0.95397 0.08047

C 2.65554 -1.10620 0.75270

N 2.51037 -2.17264 1.62546

O 3.46128 -2.97108 1.79140

O 1.40591 -2.29724 2.23400

C 1.60685 -0.15971 0.51359

S 0.07291 -0.38344 1.35034

C -0.84480 0.97213 0.68080

N -2.12993 1.05070 1.15980

C -3.05633 1.97392 0.53209

C -3.86249 1.24920 -0.54231

C -4.56717 0.02701 0.02916

O -5.61131 0.47248 0.89589

C -6.76972 -0.25356 0.58450

C -6.59798 -0.53680 -0.90006

C -7.32338 -1.74585 -1.43665

O -5.20178 -0.72932 -0.98576

C -3.59793 -0.86874 0.79073

C -2.80224 -0.05640 1.80898

N -0.45197 1.86837 -0.16611

H 3.20081 1.88476 -1.69527

H 4.66015 -1.69518 0.28872

H -3.72855 2.37145 1.31007

H -2.47817 2.79677 0.09734

H -3.18013 0.91567 -1.33912

H -4.61443 1.91658 -0.99092

H -6.82499 -1.20162 1.15676

H -7.65209 0.35870 0.82293

H -6.88853 0.36469 -1.47778

H -8.41310 -1.60951 -1.35736

H -7.03610 -2.64795 -0.87574

H -7.07386 -1.90638 -2.49516

H -2.90973 -1.33991 0.07176

H -4.16755 -1.67134 1.28455

H -2.08260 -0.69160 2.33994

H -3.48562 0.35908 2.56784

**Compound 6a**

O 1.74241 -2.64987 -1.25619

C 1.25021 -1.58860 -0.88029

C 2.15272 -0.52884 -0.32877

C 3.52110 -0.68967 -0.54969

C 4.45173 0.22242 -0.05827

C 5.91606 0.08085 -0.29212

O 6.21471 -1.00021 -0.99442

C 7.59227 -1.23297 -1.27974

O 6.73717 0.87207 0.11715

C 4.00218 1.30738 0.68641

C 2.64480 1.47191 0.92253

N 2.26148 2.62042 1.72672

O 1.11618 2.66015 2.14009

O 3.09172 3.47473 1.95309

C 1.67915 0.57569 0.40342

S -0.03042 0.85386 0.65127

C -0.71766 -0.34740 -0.47844

N -2.01994 -0.17332 -0.72457

C -2.78848 -1.17057 -1.46300

C -3.79385 -1.83308 -0.53008

C -4.69384 -0.80682 0.14547

O -5.52540 -1.42008 1.11743

C -6.86877 -1.33954 0.67748

C -6.85324 -0.13308 -0.24473

C -7.90957 -0.10327 -1.31799

O -5.55877 -0.25565 -0.82682

C -3.86440 0.28455 0.81661

C -2.85724 0.87603 -0.15942

N -0.07772 -1.35757 -0.99888

H 3.84463 -1.56171 -1.11811

H 7.62710 -2.16060 -1.86032

H 8.16138 -1.34536 -0.34623

H 8.00656 -0.40010 -1.86503

H 4.70900 2.02978 1.09491

H -2.09883 -1.90295 -1.89163

H -3.30357 -0.65243 -2.28739

H -3.25722 -2.39190 0.25218

H -4.41916 -2.54232 -1.09209

H -7.52636 -1.21745 1.54948

H -7.15853 -2.25281 0.12548

H -6.89650 0.79398 0.35885

H -7.86518 -1.01426 -1.93416

H -8.90741 -0.03775 -0.85896

H -7.77658 0.77226 -1.96976

H -3.34154 -0.14897 1.68331

H -4.53422 1.07565 1.18474

H -2.24702 1.64728 0.32502

H -3.38037 1.36480 -0.99681

**Compound 6a HMC**

O 1.18455 2.58788 -1.99418

C 0.87805 1.53282 -1.41506

C 1.92527 0.76046 -0.74158

C 3.30124 1.35856 -0.87847

C 4.39060 0.52396 -0.28389

C 5.76185 1.00336 -0.46506

O 6.69974 0.22361 0.08691

C 8.05011 0.63950 -0.05425

O 6.04194 2.02854 -1.06666

C 4.11969 -0.61021 0.40686

C 2.79184 -1.09838 0.59277

N 2.62960 -2.22780 1.35171

O 1.47856 -2.69135 1.53386

O 3.61769 -2.79468 1.86503

C 1.68935 -0.39363 -0.03686

S 0.07389 -1.08355 0.11904

C -0.87746 0.05438 -0.85375

N -2.18215 -0.27428 -0.96173

C -3.14055 0.63919 -1.56789

C -4.03481 1.25132 -0.49571

C -4.71346 0.17054 0.33509

O -5.66079 -0.53829 -0.45938

C -6.95649 -0.25354 0.02966

C -6.69995 0.10550 1.48255

C -7.71942 1.00344 2.13357

O -5.44525 0.77367 1.37720

C -3.69073 -0.82550 0.87442

C -2.82267 -1.37013 -0.25295

N -0.42740 1.12999 -1.43085

H 3.50313 1.55055 -1.94714

H 3.30392 2.37013 -0.42927

H 8.20846 1.62020 0.41796

H 8.65709 -0.12060 0.45033

H 8.33138 0.70077 -1.11580

H 4.92768 -1.19335 0.84609

H -2.59501 1.41026 -2.11941

H -3.74888 0.06328 -2.28369

H -3.43513 1.88734 0.17442

H -4.80928 1.87959 -0.96105

H -7.40476 0.60035 -0.51186

H -7.59362 -1.14000 -0.09823

H -6.57252 -0.82067 2.07531

H -8.69181 0.49094 2.18391

H -7.41606 1.25765 3.15951

H -7.84227 1.93406 1.55890

H -3.05700 -0.30788 1.61145

H -4.21456 -1.64776 1.38385

H -3.44162 -1.92427 -0.97683

H -2.07848 -2.07778 0.13087

**Compound 6a TS**

O -1.30141 2.51970 3.19228

C -2.49766 2.48038 3.49322

C -3.23986 3.75031 3.65879

C -2.47858 4.94111 3.44938

C -3.04950 6.19763 3.79907

C -2.24845 7.43769 3.82477

O -0.97761 7.22405 3.49660

C -0.10748 8.34992 3.48827

O -2.68664 8.53506 4.11041

C -4.39935 6.27166 4.05404

C -5.19574 5.11992 4.07475

N -6.58798 5.30313 4.30207

O -7.30571 4.30748 4.36011

O -7.03118 6.43705 4.42386

C -4.60628 3.82700 3.92466

S -5.59254 2.39135 4.12294

C -4.36957 1.10845 3.93733

N -4.86746 -0.13023 4.08166

C -3.99002 -1.29482 4.07908

C -3.86388 -1.84390 5.49472

C -5.22798 -2.15897 6.09266

O -5.10954 -2.52117 7.46044

C -5.52592 -3.86568 7.61376

C -6.41921 -4.09150 6.40553

C -6.53063 -5.51335 5.92074

O -5.76378 -3.28587 5.43122

C -6.16774 -0.96054 5.97396

C -6.21246 -0.44408 4.54170

N -3.10665 1.27770 3.67155

H -1.39595 4.82546 3.46145

H 0.87756 7.96882 3.19815

H -0.05848 8.80668 4.48705

H -0.45357 9.09840 2.76133

H -4.86538 7.23469 4.26267

H -3.01588 -1.00330 3.67608

H -4.43577 -2.04778 3.41004

H -3.36993 -1.09750 6.13583

H -3.25330 -2.75896 5.49658

H -6.05175 -3.97477 8.57287

H -4.65746 -4.54968 7.59344

H -7.42597 -3.67753 6.60641

H -5.53491 -5.92875 5.70308

H -7.00979 -6.13534 6.69153

H -7.14427 -5.56709 5.00993

H -5.81304 -0.16566 6.64854

H -7.17658 -1.25521 6.29914

H -6.87311 0.42692 4.45992

H -6.62196 -1.21506 3.87020

B -2.58479 5.20551 0.59420

H -2.31817 5.00273 1.84716

H -1.50870 5.12572 -0.00330

H -3.08142 6.33483 0.58298

H -3.38291 4.30852 0.30985

**Compound 6a radical anion**

O 1.22987 0.38586 -3.15189

C 0.92221 0.00800 -2.02972

C 1.93949 0.02465 -0.93487

C 3.23543 0.42507 -1.30442

C 4.25904 0.47627 -0.34692

C 5.61233 0.88449 -0.78356

O 6.51835 0.86690 0.21473

C 7.82809 1.25039 -0.13372

O 5.91809 1.20676 -1.90965

C 3.99396 0.15338 0.97656

C 2.69867 -0.23292 1.37889

N 2.48553 -0.51942 2.71912

O 1.31715 -0.85835 3.07398

O 3.43396 -0.43518 3.53426

C 1.65553 -0.31461 0.39671

S 0.06058 -0.85610 0.91144

C -0.79033 -0.83868 -0.63737

N -2.08020 -1.30715 -0.56461

C -2.78192 -1.52610 0.68269

C -3.61034 -0.29730 1.04953

C -4.55610 0.07612 -0.08236

O -5.58279 -0.91039 -0.15653

C -6.79677 -0.24018 -0.36031

C -6.59105 1.05976 0.40335

C -7.41029 2.23900 -0.06163

O -5.21694 1.30240 0.18570

C -3.81740 0.18114 -1.41119

C -2.97904 -1.06607 -1.67684

N -0.35410 -0.43334 -1.78654

H 3.42171 0.68707 -2.34475

H 7.84897 2.27778 -0.52956

H 8.42186 1.18843 0.78677

H 8.24740 0.58008 -0.90034

H 4.76272 0.18899 1.74566

H -3.44483 -2.39774 0.55284

H -2.07593 -1.77721 1.48424

H -4.19886 -0.47335 1.96281

H -2.93782 0.55452 1.23382

H -6.97725 -0.03639 -1.43524

H -7.61780 -0.86107 0.02703

H -6.77112 0.87907 1.48291

H -7.24035 2.42923 -1.13216

H -7.13149 3.14479 0.49546

H -8.48337 2.05286 0.10112

H -4.55146 0.34177 -2.21668

H -3.16045 1.06411 -1.37959

H -2.37618 -0.94791 -2.58419

H -3.63467 -1.94573 -1.78799

**Compound 6b**

O 1.66286 -2.93083 -0.66906

C 1.28479 -1.84237 -0.24226

C 2.29280 -0.75167 -0.05052

C 3.63739 -1.11726 -0.10669

C 4.65893 -0.18250 0.03736

C 6.07529 -0.64274 -0.02977

O 6.93348 0.35170 0.13277

C 8.34024 0.06049 0.06325

C 8.83900 0.08916 -1.36178

O 6.38869 -1.79964 -0.20706

C 4.32607 1.15503 0.22742

C 2.99093 1.53318 0.27739

N 2.72578 2.94962 0.46525

O 3.63241 3.66721 0.83089

O 1.59527 3.34445 0.24067

C 1.93794 0.59494 0.15625

S 0.26777 1.10014 0.28572

C -0.54268 -0.48792 0.39539

N -1.82316 -0.41408 0.77190

C -2.52858 0.80508 1.14233

C -3.60031 1.13442 0.11398

C -4.55723 -0.04258 -0.07235

O -5.34900 -0.23698 1.08143

C -6.54614 0.48971 0.88215

C -6.79281 0.31155 -0.61189

C -7.57739 -0.93545 -0.96613

O -5.46024 0.24683 -1.12360

C -3.78634 -1.32333 -0.35429

C -2.70521 -1.57428 0.68979

N -0.01236 -1.63803 0.08492

H 3.87915 -2.16777 -0.27408

H 8.81161 0.84459 0.66956

H 8.52115 -0.91378 0.53917

H 8.36594 -0.69549 -1.96971

H 8.64365 1.06633 -1.82736

H 9.92595 -0.08249 -1.36812

H 5.09704 1.91709 0.33409

H -1.82919 1.64042 1.26343

H -2.98900 0.63040 2.12817

H -3.13575 1.36732 -0.85664

H -4.16547 2.02078 0.43804

H -7.33146 0.05743 1.51623

H -6.41205 1.55419 1.14472

H -7.27304 1.20228 -1.04664

H -7.13735 -1.82541 -0.49087

H -7.58373 -1.08791 -2.05557

H -8.62012 -0.83653 -0.62706

H -3.32484 -1.23631 -1.34992

H -4.49225 -2.16639 -0.37525

H -3.15148 -1.72853 1.68511

H -2.10675 -2.45475 0.43956

**Compound 6b HMC**

O -2.08608 1.52241 -1.66320

C -1.50795 0.57476 -1.10645

C -2.28168 -0.32210 -0.24443

C -3.74735 0.02239 -0.17643

C -4.56017 -0.93276 0.63903

C -6.01605 -0.76849 0.73117

O -6.44287 0.28388 0.02524

C -7.84550 0.56367 0.02582

C -8.08019 1.78756 -0.82066

O -6.77576 -1.47593 1.37398

C -3.98493 -1.95599 1.31459

C -2.58198 -2.21312 1.27934

N -2.10496 -3.25296 2.03248

O -0.87715 -3.51130 2.02496

O -2.88055 -3.93552 2.73577

C -1.73692 -1.37600 0.44617

S -0.02147 -1.77329 0.34863

C 0.54742 -0.54751 -0.79848

N 1.85715 -0.65383 -1.11175

C 2.53107 0.39360 -1.86566

C 3.40312 1.22359 -0.93154

C 4.38931 0.33849 -0.16236

O 5.37821 -0.18292 -1.02916

C 6.39481 0.79592 -1.13279

C 6.23169 1.66463 0.13450

C 7.42205 1.66501 1.06311

O 5.10989 1.07796 0.79085

C 3.67159 -0.81248 0.51842

C 2.78911 -1.56604 -0.47007

N -0.16973 0.40240 -1.32298

H -4.14414 0.08756 -1.20482

H -3.85916 1.05366 0.20766

H -8.17754 0.71598 1.06502

H -8.38238 -0.31306 -0.37021

H -9.15481 2.02117 -0.83939

H -7.74728 1.62311 -1.85638

H -7.54644 2.65987 -0.41483

H -4.60424 -2.62519 1.91168

H 1.77865 1.01292 -2.36275

H 3.14995 -0.09011 -2.63822

H 2.76885 1.75013 -0.20132

H 3.94738 1.98516 -1.51025

H 6.27677 1.39588 -2.05112

H 7.36642 0.28181 -1.17073

H 5.98216 2.70218 -0.15120

H 7.69673 0.63584 1.34174

H 8.28528 2.13600 0.56899

H 7.20139 2.23413 1.97837

H 3.05337 -0.40509 1.33321

H 4.42099 -1.48730 0.95755

H 3.40578 -2.02478 -1.25917

H 2.25835 -2.38601 0.02836

**Compound 6b TS**

O -0.08240 5.23902 4.73070

C -0.51277 4.11133 4.98869

C -1.94390 3.81338 4.75900

C -2.67582 4.81059 4.04323

C -4.09662 4.72210 3.98706

C -4.84617 5.86273 3.42799

O -6.16393 5.67830 3.43851

C -6.99663 6.70287 2.87740

C -7.13673 6.54779 1.38134

O -4.30900 6.86581 2.99842

C -4.71186 3.55592 4.38057

C -3.97054 2.49246 4.91405

N -4.69126 1.32048 5.26657

O -5.88584 1.24890 5.00754

O -4.08589 0.40690 5.82406

C -2.56349 2.62314 5.13142

S -1.67068 1.31169 5.87752

C -0.01187 1.95896 5.82240

N 0.91891 1.08414 6.23747

C 0.68102 -0.32663 6.50560

C 1.21974 -1.17300 5.35928

C 2.69768 -0.87539 5.10211

O 3.49813 -1.36170 6.16047

C 3.91716 -2.66183 5.79389

C 4.11681 -2.53148 4.28788

C 5.49975 -2.06205 3.88350

O 3.12216 -1.56681 3.94098

C 2.93832 0.62056 4.95893

C 2.33841 1.39658 6.12495

N 0.33810 3.15637 5.45139

H -2.21151 5.79436 3.97488

H -7.96316 6.58025 3.38352

H -6.57985 7.68453 3.14527

H -6.16933 6.67061 0.87342

H -7.54878 5.56050 1.12524

H -7.82493 7.31643 0.99817

H -5.79254 3.44475 4.30327

H -0.38416 -0.52486 6.67307

H 1.19991 -0.57721 7.44404

H 0.65273 -0.96075 4.43940

H 1.09634 -2.24153 5.59057

H 4.83716 -2.90373 6.34261

H 3.14308 -3.41172 6.03565

H 3.86518 -3.47073 3.77037

H 5.78536 -1.15586 4.43922

H 5.52813 -1.83540 2.80742

H 6.24291 -2.84780 4.08923

H 2.47662 0.95632 4.01761

H 4.02141 0.80071 4.89423

H 2.82087 1.10857 7.07214

H 2.45534 2.47529 5.98723

B -1.89983 3.83244 1.54755

H -2.20596 4.63735 2.52400

H -2.76492 2.95850 1.61144

H -1.94931 4.51587 0.52227

H -0.77180 3.43224 1.84028

**Compound 6b radical anion**

O 2.05841 2.24539 -0.53909

C 1.55445 1.26244 -0.01077

C 2.36567 0.02224 0.17530

C 3.72751 0.12230 -0.16057

C 4.56984 -0.99264 -0.02940

C 6.01211 -0.92395 -0.35566

O 6.38996 0.30525 -0.75582

C 7.75743 0.47492 -1.08238

C 7.97552 1.92603 -1.43391

O 6.80058 -1.84000 -0.28257

C 4.05992 -2.20558 0.40957

C 2.69451 -2.33838 0.73669

N 2.22902 -3.57957 1.14511

O 0.99327 -3.69075 1.40548

O 3.02103 -4.54483 1.23533

C 1.83806 -1.19076 0.64088

S 0.15464 -1.35580 1.13139

C -0.39033 0.31385 0.93636

N -1.67060 0.53219 1.38447

C -2.59342 -0.53342 1.71439

C -3.48545 -0.86071 0.51970

C -4.20198 0.39052 0.03088

O -5.16600 0.81655 0.99722

C -6.44347 0.53118 0.49603

C -6.23997 0.60803 -1.00971

C -7.20695 -0.18349 -1.85423

O -4.93664 0.08006 -1.13766

C -3.22363 1.53358 -0.20315

C -2.34382 1.76484 1.02264

N 0.25345 1.31185 0.41993

H 4.09307 1.08139 -0.52029

H 8.01914 -0.19285 -1.92069

H 8.37938 0.16393 -0.22632

H 9.03037 2.09833 -1.69685

H 7.35100 2.22181 -2.28983

H 7.71775 2.57762 -0.58589

H 4.69517 -3.08466 0.50953

H -3.21756 -0.19375 2.55775

H -2.05030 -1.42370 2.05527

H -2.87476 -1.26474 -0.30263

H -4.23788 -1.61972 0.78447

H -7.15765 1.27129 0.88732

H -6.78065 -0.48329 0.79027

H -6.24774 1.67211 -1.32387

H -8.22873 0.21126 -1.74280

H -7.20045 -1.24295 -1.55694

H -6.92967 -0.12320 -2.91627

H -3.78973 2.44265 -0.45872

H -2.58426 1.27442 -1.06100

H -2.95951 2.08916 1.87779

H -1.57999 2.52353 0.81911

**Compound 6c**

O 1.74987 1.55286 1.63596

C 1.18303 0.66241 1.00677

C 1.97235 -0.13669 0.01680

C 3.35915 0.01603 0.05260

C 4.19279 -0.65993 -0.83469

C 5.67763 -0.51375 -0.81286

O 6.08314 0.31922 0.12842

C 7.50199 0.58087 0.27529

C 7.73192 0.87711 1.73891

C 7.89271 1.71598 -0.64790

O 6.40940 -1.10450 -1.57824

C 3.62221 -1.49937 -1.78484

C 2.24378 -1.65341 -1.83687

N 1.73062 -2.53941 -2.86905

O 2.51275 -3.23946 -3.47609

O 0.53029 -2.52539 -3.07756

C 1.37904 -0.99014 -0.93252

S -0.35133 -1.24398 -0.99507

C -0.85684 -0.41836 0.50601

N -2.12711 -0.66055 0.84811

C -3.08262 -1.42568 0.05771

C -4.11699 -0.49330 -0.55816

C -4.81093 0.33987 0.52036

O -5.63602 -0.47512 1.33021

C -6.93123 -0.43080 0.76824

C -7.02511 0.99966 0.25765

C -7.93602 1.20641 -0.92678

O -5.66940 1.28371 -0.09084

C -3.78918 1.03478 1.40693

C -2.75919 0.05449 1.95274

N -0.12473 0.38779 1.22324

H 3.77582 0.69173 0.79932

H 8.03143 -0.33895 -0.01579

H 7.41376 0.03174 2.36613

H 8.80305 1.05356 1.91394

H 7.17845 1.77711 2.04839

H 7.69483 1.46149 -1.69899

H 8.96801 1.92281 -0.54236

H 7.33683 2.63095 -0.39182

H 4.24935 -2.03520 -2.49735

H -2.57326 -2.01699 -0.71227

H -3.57121 -2.14301 0.73591

H -4.86599 -1.07930 -1.11080

H -3.62952 0.18906 -1.27171

H -7.03453 -1.15697 -0.05962

H -7.66591 -0.66747 1.54922

H -7.32223 1.66965 1.08570

H -7.64299 0.55175 -1.76172

H -7.90241 2.25133 -1.26798

H -8.97502 0.97454 -0.64645

H -3.28193 1.80716 0.80839

H -4.31483 1.53220 2.23531

H -1.98631 0.56781 2.53161

H -3.24099 -0.69595 2.59887

**Compound 6c HMC**

O -0.97848 -0.15999 -3.15846

C -0.63128 -0.26266 -1.97043

C -1.65143 -0.18465 -0.92174

C -3.05380 -0.03090 -1.45133

C -4.10503 0.03330 -0.38985

C -5.49702 0.13515 -0.84026

O -6.38873 0.20708 0.15262

C -7.79129 0.26891 -0.17307

C -8.32515 -1.13396 -0.38536

C -8.46518 1.00089 0.96630

O -5.81449 0.15798 -2.01972

C -3.78759 -0.00157 0.92666

C -2.44311 -0.11031 1.39331

N -2.23990 -0.11360 2.74779

O -3.20764 -0.04141 3.53557

O -1.07136 -0.18516 3.19920

C -1.36888 -0.21866 0.42169

S 0.27614 -0.41197 1.02875

C 1.18281 -0.56878 -0.48775

N 2.50066 -0.80880 -0.31723

C 3.17817 -0.83892 0.96871

C 4.01651 0.41818 1.16228

C 4.99798 0.59258 0.00790

O 5.66928 1.83284 0.15676

C 7.06147 1.60008 0.06362

C 7.19429 0.17276 0.56286

C 8.40029 -0.59079 0.08105

O 6.00054 -0.40768 0.04636

C 4.27980 0.51325 -1.33393

C 3.42797 -0.74698 -1.43859

N 0.69165 -0.45661 -1.68701

H -3.26625 -0.85853 -2.15269

H -3.10067 0.86810 -2.09405

H -7.88995 0.84761 -1.10451

H -8.20411 -1.73469 0.52939

H -9.39656 -1.09223 -0.63282

H -7.80256 -1.63695 -1.21160

H -8.03902 2.00676 1.09404

H -9.53993 1.10478 0.75763

H -8.34878 0.44624 1.91044

H -4.57090 0.05304 1.68092

H 2.46052 -0.96316 1.78833

H 3.82762 -1.72920 0.98204

H 3.36317 1.30373 1.20857

H 4.57821 0.35807 2.10662

H 7.59009 2.33631 0.68479

H 7.40966 1.68526 -0.98273

H 7.15480 0.16568 1.66930

H 8.44256 -0.59806 -1.01881

H 9.31876 -0.12218 0.46527

H 8.37242 -1.62946 0.44099

H 5.02643 0.53855 -2.14165

H 3.63538 1.40078 -1.43227

H 4.06483 -1.64552 -1.40297

H 2.85700 -0.76468 -2.37159

**Compound 6c TS**

O -0.20059 1.60425 2.42965

C -0.96713 0.80539 1.88349

C -2.42611 0.96199 2.07484

C -2.82748 2.04525 2.91771

C -4.19157 2.14279 3.31684

C -4.63222 3.10104 4.35280

O -3.61597 3.79404 4.85130

C -3.86812 4.77899 5.87820

C -2.80894 5.84520 5.71361

C -3.84703 4.10359 7.23441

O -5.78922 3.24133 4.70224

C -5.13432 1.38260 2.66617

C -4.76357 0.44169 1.69680

N -5.81425 -0.26481 1.05152

O -6.97583 -0.00368 1.33508

O -5.51853 -1.11704 0.21661

C -3.38457 0.18645 1.42453

S -2.94145 -1.10812 0.32886

C -1.16382 -1.05147 0.43424

N -0.55513 -1.99727 -0.29919

C -1.23169 -3.07754 -1.00204

C -1.00131 -4.39983 -0.28239

C 0.49000 -4.67272 -0.11684

O 1.08783 -4.93598 -1.37882

C 1.30959 -6.32906 -1.46898

C 1.56218 -6.71583 -0.02315

C 1.27684 -8.15000 0.33968

O 0.66285 -5.84185 0.65606

C 1.19754 -3.48012 0.51571

C 0.88883 -2.18908 -0.23191

N -0.45800 -0.20081 1.12215

H -2.05567 2.46239 3.56253

H -4.86639 5.20167 5.68753

H -2.85803 6.29462 4.71102

H -2.96387 6.63988 6.45768

H -1.80317 5.42281 5.86311

H -2.86185 3.65044 7.42454

H -4.61652 3.32110 7.30041

H -4.04457 4.84598 8.02190

H -6.19005 1.49048 2.91517

H -2.30302 -2.87159 -1.10817

H -0.81606 -3.12139 -2.02114

H -1.45489 -5.22393 -0.85303

H -1.47170 -4.37377 0.71298

H 0.41834 -6.85072 -1.86583

H 2.16501 -6.51223 -2.13333

H 2.60153 -6.45219 0.25140

H 0.24179 -8.41988 0.08002

H 1.42612 -8.31627 1.41640

H 1.96150 -8.81726 -0.20532

H 0.85690 -3.39041 1.55893

H 2.28159 -3.66720 0.52161

H 1.26974 -2.23809 -1.26433

H 1.33759 -1.32357 0.26376

B -2.91499 4.20370 1.10188

H -2.56647 3.34498 2.01131

H -1.87528 4.76752 0.75281

H -3.42567 3.50750 0.22242

H -3.71931 4.93711 1.68106

**Compound 6c radical anion**

O 1.45512 2.39955 0.82189

C 0.98228 1.27060 0.84648

C 1.86121 0.08914 0.59754

C 3.18304 0.37360 0.20767

C 4.08233 -0.67230 -0.05336

C 5.47423 -0.41864 -0.49224

O 5.73056 0.89386 -0.64856

C 7.03942 1.26940 -1.07636

C 6.89285 2.59497 -1.79435

C 7.97528 1.33170 0.11701

O 6.31026 -1.26986 -0.70280

C 3.67862 -1.99166 0.08746

C 2.36356 -2.30567 0.48820

N 2.01746 -3.64128 0.63268

O 2.86155 -4.53437 0.39384

O 0.83896 -3.90900 1.01503

C 1.43173 -1.24008 0.72619

S -0.22249 -1.65728 1.15841

C -0.94421 -0.04584 1.21971

N -2.29828 -0.05922 1.46428

C -3.05412 1.16098 1.25173

C -3.60220 1.19168 -0.17337

C -4.42172 -0.05205 -0.47960

O -4.79315 -0.09548 -1.85296

C -6.18707 0.02294 -1.95223

C -6.67462 -0.45080 -0.59045

C -8.00332 0.09618 -0.13227

O -5.64017 0.01651 0.24773

C -3.65560 -1.32311 -0.12848

C -3.11036 -1.24722 1.29507

N -0.36061 1.09995 1.07404

H 3.47353 1.41796 0.11862

H 7.40006 0.49490 -1.77326

H 7.86770 2.93227 -2.17810

H 6.50001 3.36483 -1.11239

H 6.19696 2.50196 -2.64067

H 7.61417 2.06998 0.84991

H 8.98680 1.62505 -0.20479

H 8.04063 0.34935 0.60500

H 4.36368 -2.81616 -0.10479

H -3.88190 1.18611 1.97927

H -2.38895 2.01317 1.43137

H -4.23279 2.07882 -0.33859

H -2.75961 1.23401 -0.88036

H -6.54631 -0.59584 -2.78884

H -6.49123 1.07410 -2.12833

H -6.69935 -1.56007 -0.57662

H -8.81050 -0.24465 -0.79876

H -8.23174 -0.24702 0.88683

H -7.98602 1.19651 -0.13057

H -4.32048 -2.19252 -0.25011

H -2.82255 -1.44097 -0.83855

H -3.94575 -1.19052 2.01239

H -2.53686 -2.14868 1.54414

**Compound 6d**

O 1.44905 3.08572 -1.04122

C 1.09546 2.03330 -0.51429

C 2.11382 0.95652 -0.29718

C 3.44625 1.30110 -0.52256

C 4.47990 0.38300 -0.36183

C 5.88333 0.82194 -0.59756

O 6.75864 -0.14798 -0.37994

C 8.14705 0.14627 -0.57476

C 8.91741 -1.12592 -0.33075

O 8.64113 -2.12915 -1.27952

O 6.18581 1.94227 -0.94270

C 4.17031 -0.91682 0.02405

C 2.84686 -1.27676 0.24145

N 2.61310 -2.65911 0.62721

O 3.56515 -3.34591 0.93140

O 1.46282 -3.05959 0.61988

C 1.78186 -0.35378 0.09802

S 0.13077 -0.83268 0.42417

C -0.69351 0.75079 0.34750

N -1.96143 0.71583 0.77094

C -2.67550 -0.47651 1.20804

C -3.74238 -0.85620 0.19005

C -4.68773 0.31317 -0.06838

O -5.47951 0.57417 1.08159

C -6.76465 0.03394 0.84759

C -6.92114 0.19826 -0.65338

C -7.87555 -0.75334 -1.32756

O -5.58637 -0.03709 -1.09916

C -3.90947 1.57550 -0.41642

C -2.83789 1.87449 0.62378

N -0.18324 1.86161 -0.10628

H 3.66616 2.32355 -0.83290

H 8.29474 0.51376 -1.60185

H 8.45610 0.93136 0.13313

H 8.72472 -1.48384 0.69700

H 9.98986 -0.89169 -0.40497

H 7.72312 -2.40888 -1.17368

H 4.94975 -1.66581 0.15718

H -3.14164 -0.24394 2.17868

H -1.98329 -1.30872 1.38150

H -4.32422 -1.71318 0.56032

H -3.27037 -1.14833 -0.76095

H -7.50143 0.59955 1.43391

H -6.80838 -1.03369 1.13398

H -7.20032 1.24442 -0.88203

H -7.60472 -1.79752 -1.10910

H -7.86728 -0.60615 -2.41734

H -8.90007 -0.57288 -0.96856

H -4.60909 2.42097 -0.49145

H -3.43656 1.42975 -1.39993

H -2.23372 2.74027 0.33926

H -3.29348 2.07355 1.60663

**Compound 6d HMC**

O 1.71196 -0.18552 -2.70806

C 1.24131 0.03215 -1.57999

C 2.10305 -0.13209 -0.40550

C 3.53830 -0.44322 -0.74487

C 4.44469 -0.49263 0.44563

C 5.88534 -0.64271 0.26636

O 6.23076 -0.72420 -1.01944

C 7.59430 -0.99553 -1.37307

C 8.55052 0.15561 -1.13083

O 9.05351 0.23298 0.18077

O 6.71458 -0.68834 1.17179

C 3.95994 -0.40395 1.71028

C 2.57653 -0.22943 1.99327

N 2.18606 -0.23853 3.30877

O 0.97269 -0.10818 3.59202

O 3.02818 -0.38231 4.21861

C 1.65277 -0.04089 0.88697

S -0.02260 0.35220 1.27033

C -0.67547 0.65901 -0.35020

N -1.93922 1.13248 -0.35272

C -2.71841 1.19289 -1.58166

C -3.78281 0.10086 -1.56955

C -4.66062 0.18628 -0.32787

O -5.47387 1.34537 -0.42800

C -6.76621 0.99388 0.02156

C -6.88611 -0.45231 -0.42598

C -7.84508 -1.30959 0.35961

O -5.54756 -0.91356 -0.25212

C -3.81269 0.23503 0.94029

C -2.75441 1.32528 0.83528

N -0.05814 0.44037 -1.47437

H 3.89666 0.30274 -1.47491

H 3.58111 -1.39644 -1.30500

H 7.54768 -1.20839 -2.44869

H 7.93278 -1.90300 -0.84671

H 8.05107 1.09750 -1.43164

H 9.40953 0.01692 -1.80736

H 8.32165 -0.02816 0.77271

H 4.64375 -0.46488 2.55694

H -3.18737 2.18831 -1.63783

H -2.04397 1.08077 -2.43550

H -4.42010 0.17618 -2.46327

H -3.29492 -0.88629 -1.57730

H -6.84375 1.07387 1.12230

H -7.50053 1.66464 -0.44444

H -7.14188 -0.48309 -1.50229

H -7.81285 -2.35150 0.00927

H -8.87316 -0.93924 0.22886

H -7.59698 -1.28917 1.43177

H -4.46706 0.42199 1.80489

H -3.33228 -0.74566 1.08259

H -2.13126 1.36064 1.73689

H -3.23933 2.31105 0.74790

**Compound 6d TS**

O -3.70955 0.26111 -0.73678

C -2.93843 0.34325 -1.69682

C -3.15560 1.40497 -2.70575

C -4.23382 2.30156 -2.43802

C -4.66127 3.19755 -3.45697

C -5.90474 3.95982 -3.24441

O -6.20634 4.75862 -4.26564

C -7.37308 5.57536 -4.15669

C -7.43811 6.42297 -5.40208

O -6.34953 7.30949 -5.51772

O -6.58513 3.87572 -2.24130

C -3.87180 3.37494 -4.57038

C -2.69950 2.62977 -4.74784

N -1.93352 2.90887 -5.91432

O -0.90998 2.25818 -6.10966

O -2.30824 3.78417 -6.68235

C -2.34544 1.59886 -3.82377

S -0.94428 0.59388 -4.13584

C -1.01201 -0.52870 -2.75324

N -0.01629 -1.42814 -2.74594

C 1.01842 -1.55210 -3.76219

C 0.86202 -2.85977 -4.52570

C 0.85428 -4.04882 -3.56977

O 2.13135 -4.21803 -2.97598

C 2.79481 -5.24091 -3.68977

C 1.65266 -6.16524 -4.07383

C 1.87680 -7.01343 -5.29937

O 0.59527 -5.23299 -4.29793

C -0.17064 -3.84555 -2.46278

C 0.02209 -2.50568 -1.76427

N -1.90820 -0.53727 -1.80862

H -4.95332 1.98495 -1.68359

H -8.26768 4.93819 -4.07179

H -7.29483 6.19881 -3.25237

H -8.35365 7.03135 -5.35760

H -7.51417 5.76901 -6.29016

H -5.54080 6.78836 -5.60139

H -4.15043 4.09063 -5.34240

H 1.99320 -1.52644 -3.24859

H 1.00301 -0.69814 -4.44916

H 1.68870 -2.97552 -5.24247

H -0.08174 -2.85505 -5.09296

H 3.54129 -5.70741 -3.03297

H 3.29803 -4.83924 -4.58948

H 1.39111 -6.80456 -3.20934

H 2.13337 -6.38540 -6.16603

H 0.97617 -7.59684 -5.53969

H 2.70136 -7.72000 -5.12021

H -0.08302 -4.66772 -1.73713

H -1.17524 -3.88608 -2.91138

H 1.00115 -2.46919 -1.25987

H -0.75796 -2.33045 -1.01803

B -2.92308 4.14473 -0.67127

H -3.65381 3.26284 -1.28043

H -2.76674 5.02487 -1.52124

H -1.88820 3.53607 -0.38877

H -3.57288 4.50951 0.31133

**Compound 6d radical anion**

O 1.45580 -3.02067 0.41672

C 1.04787 -2.00399 -0.12658

C 1.96525 -0.83871 -0.31118

C 3.22653 -0.94302 0.29998

C 4.14993 0.11019 0.19487

C 5.45303 -0.01961 0.86944

O 6.24510 1.04825 0.70920

C 7.47174 1.11107 1.42905

C 8.54851 0.14579 0.95657

O 8.51255 -1.11415 1.55493

O 5.80258 -0.99221 1.51609

C 3.83303 1.24800 -0.53388

C 2.58729 1.36481 -1.18028

N 2.33882 2.51212 -1.92060

O 3.20772 3.41170 -1.98635

O 1.22673 2.60147 -2.51792

C 1.62457 0.30771 -1.04494

S 0.04800 0.51097 -1.79822

C -0.76124 -0.95091 -1.22283

N -2.08556 -1.02312 -1.58433

C -2.92496 -2.01434 -0.93617

C -3.56678 -1.40766 0.30897

C -4.33471 -0.13638 -0.02796

O -5.50170 -0.49600 -0.76959

C -6.59534 0.17948 -0.20961

C -6.19511 0.31397 1.25201

C -6.82251 1.45540 2.01336

O -4.80167 0.51267 1.13997

C -3.48076 0.83692 -0.82971

C -2.83940 0.13436 -2.02325

N -0.26033 -1.92438 -0.53384

H 3.46179 -1.84930 0.85565

H 7.27943 0.95809 2.50499

H 7.80948 2.14534 1.27445

H 9.52762 0.59368 1.20047

H 8.49286 0.08633 -0.15058

H 7.57127 -1.36245 1.62189

H 4.52771 2.07842 -0.64086

H -2.30130 -2.87601 -0.67344

H -3.70163 -2.32948 -1.65163

H -2.77578 -1.15416 1.03138

H -4.25313 -2.12171 0.78973

H -6.73611 1.17959 -0.66708

H -7.50596 -0.41689 -0.36944

H -6.39723 -0.64385 1.77413

H -6.62365 2.41199 1.50715

H -7.91155 1.31440 2.09339

H -6.40849 1.51331 3.03006

H -4.11171 1.67765 -1.15745

H -2.69573 1.24418 -0.17374

H -3.62299 -0.21631 -2.71470

H -2.20006 0.82383 -2.58839

**Compound 6e**

O -1.60040 -3.02118 0.57564

C -1.24295 -1.85211 0.45058

C -2.25407 -0.82953 0.03350

C -3.59067 -1.22564 0.05307

C -4.61668 -0.36505 -0.32684

C -6.02346 -0.84517 -0.26185

O -6.88839 0.07879 -0.66680

C -8.28068 -0.22040 -0.59181

C -8.83141 -0.09050 0.80354

I -8.54730 1.88539 1.63618

O -6.33973 -1.94613 0.12727

C -4.29542 0.92005 -0.75101

C -2.96735 1.32354 -0.78589

N -2.71837 2.67678 -1.25663

O -3.66058 3.42857 -1.38746

O -1.56534 2.98474 -1.50048

C -1.91055 0.47197 -0.38046

S -0.25514 1.03675 -0.37619

C 0.55189 -0.31560 0.46634

N 1.81702 -0.05248 0.81023

C 2.53937 1.17361 0.49968

C 3.58924 0.90269 -0.56979

C 4.53416 -0.21159 -0.14128

O 5.34912 0.26942 0.90887

C 6.62754 -0.31131 0.73552

C 6.76662 -0.35191 -0.78194

C 7.34030 0.91331 -1.38678

O 5.41425 -0.55847 -1.19360

C 3.75243 -1.44847 0.29939

C 2.68957 -1.09944 1.33291

N 0.03491 -1.48828 0.70809

H -3.82083 -2.24221 0.37517

H -8.76396 0.47775 -1.28637

H -8.45473 -1.25141 -0.93995

H -8.35633 -0.77857 1.51262

H -9.91570 -0.25764 0.79604

H -5.06899 1.62084 -1.06197

H 3.01650 1.52041 1.42976

H 1.85176 1.96726 0.18427

H 4.17341 1.81314 -0.76635

H 3.09561 0.59913 -1.50572

H 6.66511 -1.32500 1.17247

H 7.37379 0.32373 1.23120

H 7.34908 -1.22863 -1.10607

H 6.80601 1.80345 -1.02123

H 7.26125 0.88411 -2.48359

H 8.40437 1.01128 -1.12188

H 4.44895 -2.18994 0.71879

H 3.27859 -1.89807 -0.58709

H 2.08157 -1.97050 1.59243

H 3.15661 -0.71155 2.25179

**Compound 6e HMC**

O -1.40584 -2.83674 1.75513

C -1.07008 -1.70394 1.37355

C -2.10574 -0.76423 0.93696

C -3.49510 -1.34728 0.94454

C -4.54156 -0.43128 0.39460

C -5.89146 -0.97405 0.25580

O -6.78320 -0.10651 -0.24683

C -8.10878 -0.57275 -0.43819

C -8.91261 0.51056 -1.09495

I -8.19010 1.00124 -3.07667

O -6.20152 -2.11348 0.56344

C -4.25512 0.84785 0.04779

C -2.94443 1.39809 0.15847

N -2.78126 2.72138 -0.16056

O -3.75593 3.40962 -0.53023

O -1.64390 3.24184 -0.07484

C -1.85321 0.53579 0.57671

S -0.22402 1.21056 0.58012

C 0.73067 -0.20978 1.04755

N 2.06160 0.01223 1.08410

C 2.99942 -1.07897 1.30678

C 3.72169 -1.41349 0.00652

C 4.40133 -0.18585 -0.58455

O 4.94483 -0.47011 -1.86543

C 6.35503 -0.37212 -1.79232

C 6.57456 0.55097 -0.60578

C 7.88657 0.39838 0.11845

O 5.49617 0.16408 0.23909

C 3.41957 0.97727 -0.70453

C 2.71367 1.22661 0.62211

N 0.25725 -1.37942 1.36357

H -3.48565 -2.29951 0.38537

H -3.75490 -1.65508 1.97555

H -8.10304 -1.49945 -1.03267

H -8.56788 -0.80228 0.53969

H -8.88185 1.45214 -0.53298

H -9.95212 0.18999 -1.23195

H -5.03682 1.50239 -0.33483

H 3.72019 -0.75596 2.07514

H 2.45093 -1.94471 1.68922

H 4.47937 -2.19267 0.17754

H 2.99598 -1.79391 -0.72911

H 6.73919 0.03219 -2.73946

H 6.80849 -1.36328 -1.60630

H 6.43334 1.60206 -0.92342

H 7.92637 1.06033 0.99560

H 8.02763 -0.64089 0.45221

H 8.71774 0.66864 -0.54994

H 3.96288 1.88040 -1.02049

H 2.67963 0.73583 -1.48371

H 3.44562 1.52613 1.38930

H 1.99620 2.05076 0.53393

**Compound 6e TS**

O -4.10493 7.34128 2.22351

C -2.92512 7.62808 2.44536

C -2.04707 6.61858 3.07831

C -2.66176 5.36305 3.37167

C -1.95519 4.41141 4.15959

C -2.67475 3.20693 4.60534

O -1.88352 2.33284 5.23449

C -2.43475 1.07393 5.60016

C -2.53654 0.12625 4.43347

I -0.62600 -0.24119 3.48712

O -3.85836 3.01560 4.41477

C -0.61181 4.59608 4.39234

C 0.04225 5.75546 3.95513

N 1.43594 5.84518 4.22243

O 2.00144 4.91385 4.77931

O 2.03244 6.86253 3.87627

C -0.68597 6.80532 3.31547

S 0.12966 8.29774 2.89440

C -1.22120 9.24973 2.22875

N -0.86046 10.48154 1.83232

C 0.44820 11.07918 2.05462

C 0.37550 12.07368 3.20644

C -0.69635 13.12699 2.96051

O -0.25071 13.98994 1.93369

C -0.72772 15.28300 2.25083

C -0.62175 15.30805 3.77180

C 0.72643 15.76491 4.29073

O -0.86784 13.94144 4.10685

C -2.03060 12.48016 2.58849

C -1.86498 11.47065 1.45921

N -2.45702 8.85966 2.10654

H -3.75137 5.34502 3.38071

H -1.77524 0.68180 6.38454

H -3.44410 1.21514 6.01974

H -3.18634 0.50837 3.63712

H -2.89749 -0.85412 4.76851

H -0.03433 3.85242 4.93968

H 0.74195 11.58688 1.12262

H 1.20793 10.31152 2.24456

H 1.34425 12.57627 3.34157

H 0.13114 11.54097 4.13854

H -1.77211 15.41438 1.91631

H -0.09214 16.02600 1.75100

H -1.42841 15.91118 4.21764

H 1.54270 15.20911 3.80447

H 0.79353 15.60550 5.37710

H 0.86629 16.83860 4.09149

H -2.74510 13.26311 2.29307

H -2.43513 11.97705 3.48052

H -2.80597 10.95796 1.23995

H -1.51570 11.97026 0.54221

B -2.31942 3.92255 0.98484

H -2.81562 4.66322 1.92943

H -3.08021 4.04624 0.02207

H -1.19674 4.39677 0.80109

H -2.30054 2.79191 1.47623

**Compound 6e radical anion**

O 1.89966 2.08440 -1.63451

C 1.40604 0.99367 -1.37860

C 2.26539 -0.09956 -0.83128

C 3.62062 0.22366 -0.63968

C 4.50993 -0.73904 -0.13343

C 5.93620 -0.43896 0.09425

O 6.24646 0.84740 -0.20241

C 7.58969 1.23979 -0.00360

C 7.81628 1.55304 1.46106

I 9.81280 2.30429 1.78348

O 6.77228 -1.21134 0.50540

C 4.05724 -2.01005 0.18761

C 2.70342 -2.35854 0.01119

N 2.30142 -3.63944 0.36312

O 3.13665 -4.45242 0.81652

O 1.08170 -3.93923 0.20510

C 1.79028 -1.37944 -0.51048

S 0.10673 -1.84143 -0.73402

C -0.54988 -0.33194 -1.37568

N -1.88901 -0.39945 -1.67537

C -2.75771 -1.44993 -1.18530

C -3.41302 -1.02159 0.12506

C -4.15528 0.29839 -0.05105

O -5.30430 0.06151 -0.86978

C -6.43192 0.56429 -0.20493

C -6.02869 0.48245 1.25992

C -6.71629 1.44287 2.19737

O -4.65183 0.78355 1.18001

C -3.26483 1.35650 -0.68463

C -2.60674 0.82939 -1.95665

N 0.06713 0.78607 -1.58883

H 3.94397 1.22936 -0.89835

H 8.27359 0.44474 -0.33756

H 7.73612 2.13422 -0.62453

H 7.13758 2.33685 1.81862

H 7.72772 0.65459 2.08261

H 4.73103 -2.76820 0.58451

H -2.20119 -2.38695 -1.05888

H -3.53160 -1.63662 -1.94791

H -2.63678 -0.88683 0.89420

H -4.11804 -1.78856 0.48058

H -6.63670 1.61537 -0.49160

H -7.30706 -0.05215 -0.46046

H -6.16703 -0.55826 1.61875

H -7.79487 1.22784 2.24716

H -6.30144 1.35409 3.21146

H -6.57569 2.47999 1.85741

H -2.48537 1.64270 0.03824

H -3.87449 2.24896 -0.89508

H -3.37524 0.61119 -2.71618

H -1.89504 1.55847 -2.35968

**Compound 7a**

O -1.10910 2.04370 2.68010

C -0.98599 1.19816 1.79657

C -2.12858 0.96011 0.85806

C -3.34067 1.56991 1.18106

C -4.46628 1.46109 0.36867

C -5.73226 2.14056 0.81023

N -6.62980 2.43011 -0.14472

O -5.90718 2.40372 1.99707

C -4.37262 0.69872 -0.79094

C -3.17376 0.07615 -1.12220

N -3.17025 -0.70713 -2.34647

O -2.09092 -1.06996 -2.78007

O -4.23114 -0.96673 -2.87419

C -2.01939 0.18525 -0.31201

S -0.53192 -0.62699 -0.75043

C 0.44552 -0.34127 0.71545

N 1.59348 -1.02822 0.73598

C 2.08378 -1.88069 -0.33847

C 3.25040 -1.20292 -1.04457

C 4.35518 -0.84705 -0.05647

O 4.96378 -2.05130 0.38787

C 6.36344 -1.85464 0.36829

C 6.54389 -0.89961 -0.79784

C 7.78019 -0.03869 -0.76451

O 5.37342 -0.09263 -0.67866

C 3.79959 -0.06120 1.12682

C 2.60798 -0.77300 1.75398

N 0.15551 0.47898 1.68687

H -3.38891 2.15903 2.09770

H -7.49474 2.88915 0.11882

H -6.44198 2.31139 -1.13178

H -5.22082 0.55386 -1.46085

H 2.40349 -2.83251 0.11394

H 1.28191 -2.12272 -1.04600

H 2.90121 -0.27544 -1.52445

H 3.66019 -1.86329 -1.82260

H 6.71546 -1.39550 1.31115

H 6.85841 -2.82510 0.22800

H 6.49733 -1.46641 -1.74727

H 7.82974 0.53651 0.17258

H 7.78874 0.66312 -1.61106

H 8.67876 -0.66979 -0.83744

H 3.49679 0.93836 0.77735

H 4.59546 0.06856 1.87558

H 2.16434 -0.17898 2.55751

H 2.91414 -1.74718 2.16615

**Compound 7a HMC**

O 1.66637 -3.20028 -0.32192

C 1.27858 -2.02162 -0.38228

C 2.26809 -0.94512 -0.31738

C 3.68503 -1.43929 -0.17642

C 4.70895 -0.35261 -0.06125

C 6.14272 -0.67849 0.13135

N 6.45200 -1.99263 0.19922

O 7.02086 0.18872 0.22305

C 4.35496 0.95037 -0.12606

C 3.00300 1.38652 -0.29057

N 2.76859 2.73251 -0.35396

O 1.59131 3.15295 -0.47752

O 3.71543 3.54739 -0.28811

C 1.95262 0.39025 -0.37398

S 0.28876 0.95139 -0.54121

C -0.58780 -0.59027 -0.59748

N -1.92350 -0.45759 -0.74091

C -2.63852 0.80807 -0.73148

C -3.38802 0.98740 0.58252

C -4.31621 -0.19658 0.84208

O -4.89170 -0.06179 2.13337

C -6.30108 -0.06455 2.00602

C -6.51766 0.39142 0.57336

C -7.79758 -0.06247 -0.07835

O -5.39469 -0.19961 -0.07202

C -3.56692 -1.51574 0.71543

C -2.81000 -1.60482 -0.60489

N -0.05952 -1.77573 -0.50955

H 3.91102 -2.10169 -1.03512

H 3.72803 -2.11561 0.69868

H 7.41962 -2.26314 0.32776

H 5.76058 -2.72657 0.12541

H 5.12573 1.71624 -0.04286

H -3.34994 0.79526 -1.57302

H -1.95601 1.64709 -0.91144

H -2.67286 1.06469 1.41652

H -3.98071 1.91416 0.55701

H -6.73316 0.61742 2.75202

H -6.70842 -1.08079 2.16121

H -6.43293 1.49397 0.51985

H -7.88407 -1.15921 -0.04368

H -7.83744 0.26326 -1.12783

H -8.66046 0.37448 0.44631

H -2.85490 -1.58910 1.55218

H -4.28677 -2.34330 0.80243

H -2.21438 -2.52091 -0.65939

H -3.51222 -1.59508 -1.45395

**Compound 7a TS**

O -4.39996 7.67326 3.00277

C -3.24963 7.72060 3.44814

C -2.56266 6.46152 3.81033

C -3.26953 5.25765 3.50501

C -2.82080 4.02148 4.05650

C -3.72231 2.84256 3.92733

N -3.39040 1.71899 4.59264

O -4.73271 2.90879 3.22438

C -1.55587 3.96633 4.59280

C -0.75839 5.11744 4.70063

N 0.54913 4.94484 5.22425

O 1.24937 5.94220 5.39124

O 0.94642 3.81709 5.49154

C -1.27959 6.39901 4.34906

S -0.32003 7.83890 4.62999

C -1.43339 9.10642 4.05763

N -0.92627 10.34615 4.14981

C 0.42823 10.67451 4.56885

C 1.22047 11.23213 3.39366

C 0.51429 12.43274 2.78067

O 0.57617 13.50850 3.70346

C 0.84856 14.68173 2.96521

C 1.73452 14.16597 1.84476

C 1.73170 14.97945 0.57581

O 1.17593 12.87379 1.61151

C -0.93452 12.09523 2.44250

C -1.65823 11.50092 3.64316

N -2.63478 8.92594 3.58908

H -4.32970 5.36416 3.27682

H -3.99943 0.91292 4.51706

H -2.62518 1.66934 5.25148

H -1.11899 3.03700 4.95870

H 0.35853 11.42321 5.37444

H 0.93388 9.80004 4.99426

H 1.32854 10.45783 2.61838

H 2.22510 11.53475 3.72362

H -0.08050 15.12683 2.56188

H 1.34452 15.40897 3.62205

H 2.76897 14.04797 2.21953

H 0.70706 15.09770 0.19154

H 2.34649 14.49768 -0.19820

H 2.15176 15.97795 0.77045

H -0.94205 11.37821 1.60674

H -1.44894 13.00918 2.10961

H -2.67292 11.18699 3.38253

H -1.72067 12.24130 4.45711

B -2.37460 5.12722 0.83518

H -3.07448 5.07910 1.93158

H -1.31501 4.57838 1.14253

H -2.25859 6.33352 0.61007

H -3.03098 4.51227 -0.00831

**Compound 7a radical anion**

O 1.58592 2.74345 1.56892

C 1.24285 1.83216 0.82296

C 2.23361 0.79112 0.42416

C 3.52530 0.93914 0.96361

C 4.53888 0.01786 0.65943

C 5.93367 0.16320 1.17791

N 6.24685 1.39044 1.68956

O 6.75812 -0.73696 1.15467

C 4.25900 -1.06115 -0.16346

C 2.97274 -1.23661 -0.72200

N 2.75002 -2.32694 -1.55057

O 1.59000 -2.46715 -2.04149

O 3.67842 -3.12825 -1.79171

C 1.94200 -0.28654 -0.42149

S 0.34612 -0.51535 -1.13178

C -0.50192 0.87455 -0.44680

N -1.81010 0.97975 -0.85740

C -2.67923 1.92055 -0.17700

C -3.42282 1.22253 0.95826

C -4.17963 0.00275 0.44603

O -4.75061 -0.69181 1.54764

C -6.12891 -0.82993 1.32899

C -6.44825 0.33741 0.40712

C -7.65564 0.17556 -0.48198

O -5.27325 0.39897 -0.37187

C -3.27598 -0.90969 -0.37083

C -2.54045 -0.12093 -1.45168

N -0.04767 1.77378 0.36819

H 3.65775 1.78123 1.64275

H 7.19415 1.53648 2.00687

H 5.61988 2.17660 1.60709

H 5.02803 -1.79262 -0.40823

H -3.39778 2.31867 -0.91236

H -2.06280 2.74098 0.20689

H -2.70083 0.89827 1.72336

H -4.13640 1.90774 1.44257

H -6.36768 -1.79254 0.83406

H -6.65439 -0.78748 2.29524

H -6.54809 1.26229 1.01222

H -7.56102 -0.73083 -1.09882

H -7.75974 1.04014 -1.15307

H -8.57198 0.09879 0.12335

H -2.54195 -1.36973 0.30845

H -3.88656 -1.71312 -0.81015

H -3.26458 0.29665 -2.17053

H -1.86508 -0.77277 -2.01973

**Compound 7b**

O -1.73361 -2.83317 0.79463

C -1.25356 -1.71005 0.65278

C -2.14878 -0.59444 0.21017

C -3.51576 -0.87811 0.15318

C -4.45258 0.07218 -0.24501

C -5.93536 -0.17121 -0.30622

N -6.32740 -1.45256 -0.38609

C -7.70901 -1.86224 -0.43661

O -6.71482 0.78090 -0.28457

C -3.99602 1.34071 -0.58818

C -2.63962 1.63335 -0.55564

N -2.25864 2.97779 -0.95574

O -1.07681 3.19086 -1.16187

O -3.12514 3.81881 -1.06756

C -1.67491 0.67949 -0.15111

S 0.02551 1.09207 -0.08630

C 0.68939 -0.36307 0.70629

N 1.97502 -0.24466 1.05652

C 2.72834 -1.38799 1.56390

C 3.77326 -1.80663 0.53825

C 4.68357 -0.64517 0.16204

O 5.50205 -0.33208 1.27999

C 6.82270 -0.15338 0.80836

C 6.88429 -1.11158 -0.36736

C 7.89543 -0.78708 -1.43678

O 5.56023 -0.99866 -0.88641

C 3.86791 0.57414 -0.25825

C 2.81976 0.91260 0.79334

N 0.05327 -1.48185 0.91852

H -3.80597 -1.88704 0.45319

H -5.62314 -2.17605 -0.46788

H -7.95382 -2.51380 0.41613

H -7.91799 -2.40897 -1.36827

H -8.34420 -0.96989 -0.39745

H -4.70345 2.11178 -0.89290

H 3.20867 -1.07844 2.50557

H 2.03137 -2.20212 1.78030

H 4.38533 -2.62918 0.93660

H 3.27010 -2.16089 -0.37475

H 6.99138 0.88867 0.47727

H 7.52573 -0.39750 1.61626

H 7.04056 -2.14198 0.00533

H 7.81371 -1.49328 -2.27570

H 8.91346 -0.86353 -1.02601

H 7.74547 0.23452 -1.81783

H 4.54559 1.42912 -0.39957

H 3.38046 0.36081 -1.22238

H 3.30810 1.18549 1.74228

H 2.21969 1.77752 0.48715

**Compound 7b HMC**

O 1.59981 -2.91401 0.51681

C 1.14551 -1.75833 0.47881

C 2.06584 -0.63925 0.27508

C 3.50730 -1.06181 0.14427

C 4.47567 0.07304 0.01111

C 5.93584 -0.17723 -0.07686

N 6.32140 -1.47183 -0.07147

C 7.70600 -1.85957 -0.16592

O 6.77199 0.73421 -0.15176

C 4.04437 1.35219 -0.04275

C 2.66199 1.71736 0.02668

N 2.34583 3.04384 -0.06644

O 1.14203 3.40154 -0.01726

O 3.24430 3.90456 -0.20188

C 1.67144 0.67444 0.20364

S -0.02208 1.15172 0.33871

C -0.79971 -0.42312 0.59646

N -2.13514 -0.35327 0.78405

C -2.94544 0.83415 0.56714

C -3.77002 0.67649 -0.70531

C -4.61894 -0.58770 -0.65925

O -5.26837 -0.80752 -1.90291

C -6.66258 -0.65172 -1.72012

C -6.84784 -0.95157 -0.24291

C -8.04869 -0.32557 0.41661

O -5.64731 -0.40696 0.29540

C -3.76595 -1.80583 -0.31873

C -2.93474 -1.56059 0.93425

N -0.20061 -1.57730 0.62903

H 3.76076 -1.69263 1.01742

H 3.58951 -1.75379 -0.71731

H 5.62294 -2.20241 -0.02723

H 8.29354 -1.44943 0.67075

H 8.15995 -1.50219 -1.10431

H 7.76979 -2.95384 -0.13788

H 4.77225 2.15635 -0.14588

H -3.60608 0.96012 1.44011

H -2.31980 1.73281 0.51279

H -4.42910 1.54593 -0.84606

H -3.09605 0.60850 -1.57338

H -6.97856 0.38195 -1.95368

H -7.19328 -1.35208 -2.38034

H -6.85472 -2.04773 -0.08722

H -8.06129 -0.54809 1.49341

H -8.97203 -0.72918 -0.02524

H -8.04204 0.76661 0.28044

H -4.42252 -2.67717 -0.17419

H -3.10234 -2.01926 -1.17140

H -2.26727 -2.40280 1.13881

H -3.59465 -1.41945 1.80562

**Compound 7b TS**

O -2.55562 2.47213 4.18442

C -3.72616 2.86317 4.13171

C -3.99774 4.30427 3.94713

C -2.83836 5.13170 3.75929

C -2.98967 6.55470 3.83073

C -1.81716 7.47349 3.84907

N -0.68418 6.97753 3.31929

C 0.51980 7.76458 3.22454

O -1.89121 8.61402 4.31719

C -4.25097 7.09073 3.80814

C -5.39760 6.27575 3.78462

N -6.65063 6.92867 3.70241

O -7.67401 6.24349 3.72601

O -6.68894 8.15059 3.60614

C -5.27562 4.85737 3.89224

S -6.71954 3.86655 4.00591

C -6.00671 2.24293 4.17465

N -6.91935 1.25956 4.24114

C -6.52221 -0.11627 4.51703

C -6.96338 -0.50582 5.92196

C -8.45975 -0.29725 6.11122

O -9.15206 -1.25686 5.32718

C -10.21185 -1.76370 6.11228

C -9.63233 -1.71488 7.51467

C -10.63021 -1.62655 8.64029

O -8.84493 -0.52675 7.45178

C -8.86991 1.11619 5.70827

C -8.36001 1.45305 4.31304

N -4.73780 1.95935 4.23598

H -1.90163 4.68449 4.09785

H -0.73883 6.12081 2.76888

H 0.40396 8.61172 2.52762

H 1.33482 7.12325 2.86779

H 0.79343 8.17004 4.20956

H -4.38181 8.17286 3.82037

H -7.00621 -0.76188 3.76695

H -5.43789 -0.20030 4.40156

H -6.71956 -1.56037 6.11881

H -6.43223 0.11533 6.65975

H -11.11030 -1.12284 6.03388

H -10.45683 -2.77779 5.76870

H -8.96352 -2.58367 7.66539

H -10.11765 -1.52679 9.60810

H -11.24065 -2.54175 8.67216

H -11.29813 -0.76256 8.50272

H -9.96693 1.19426 5.73751

H -8.45804 1.82705 6.44169

H -8.82256 0.78581 3.56835

H -8.63631 2.47631 4.03293

B -1.87868 4.77929 1.12753

H -2.42125 4.84822 2.32636

H -0.66216 4.80905 1.31885

H -2.33055 5.78172 0.57881

H -2.29262 3.70727 0.69555

**Compound 7b radical anion**

O 1.70985 2.82489 0.78804

C 1.23124 1.69602 0.75302

C 2.06895 0.55208 0.28942

C 3.40082 0.86250 -0.04693

C 4.27550 -0.13432 -0.50481

C 5.69029 0.15417 -0.89479

N 5.95209 1.46248 -1.18952

C 7.24786 1.91932 -1.59714

O 6.55458 -0.70822 -0.96012

C 3.82582 -1.43947 -0.62170

C 2.49057 -1.77995 -0.30835

N 2.08267 -3.09466 -0.48148

O 0.87827 -3.38078 -0.20889

O 2.89396 -3.95011 -0.89798

C 1.59943 -0.76185 0.16422

S -0.05136 -1.21123 0.57978

C -0.67234 0.34984 1.12476

N -1.96773 0.29531 1.58187

C -2.85045 -0.82088 1.31312

C -3.65356 -0.56994 0.03986

C -4.41018 0.75119 0.13370

O -5.46531 0.65384 1.08603

C -6.64732 0.40198 0.37622

C -6.42759 1.17026 -0.91931

C -7.18074 0.66270 -2.12501

O -5.03663 1.01939 -1.11114

C -3.49138 1.89048 0.54510

C -2.68945 1.53537 1.79459

N -0.06943 1.49652 1.13605

H 3.69963 1.89861 0.11489

H 5.17309 2.10662 -1.18885

H 7.62542 2.71862 -0.93699

H 7.93332 1.06280 -1.54248

H 7.24491 2.29716 -2.63420

H 4.48884 -2.23044 -0.96919

H -3.53574 -0.92504 2.17066

H -2.28269 -1.75703 1.24230

H -2.97449 -0.52718 -0.82576

H -4.37119 -1.38533 -0.14179

H -6.78086 -0.67956 0.17151

H -7.50273 0.75967 0.96764

H -6.66501 2.24073 -0.75130

H -8.26637 0.77718 -1.97989

H -6.95550 -0.39981 -2.30204

H -6.89369 1.22732 -3.02354

H -4.09963 2.79392 0.70527

H -2.79370 2.08887 -0.28287

H -3.36379 1.39967 2.65604

H -1.96119 2.32069 2.02591

**Compound 7c**

O 1.91157 2.14101 1.41210

C 1.44657 1.08315 0.99231

C 2.37757 0.06896 0.40357

C 3.74459 0.30119 0.57243

C 4.70755 -0.56415 0.06123

C 6.17081 -0.40853 0.36955

N 6.76062 0.76843 0.08500

C 8.12532 1.00448 0.50633

C 6.15916 1.82926 -0.69892

O 6.75802 -1.35695 0.89522

C 4.28568 -1.68495 -0.64483

C 2.92992 -1.91217 -0.85029

N 2.58155 -3.08325 -1.63578

O 3.45259 -3.88242 -1.90846

O 1.42028 -3.20301 -1.98621

C 1.93707 -1.05722 -0.31434

S 0.23481 -1.41235 -0.52409

C -0.48861 -0.20531 0.57259

N -1.79404 -0.39699 0.79526

C -2.60720 0.62508 1.44657

C -3.53956 1.26469 0.42490

C -4.38858 0.22150 -0.28694

O -5.14787 0.84951 -1.29054

C -6.39383 0.17811 -1.34395

C -6.65083 -0.15687 0.12156

C -7.36947 0.93437 0.88922

O -5.32567 -0.34829 0.61808

C -3.51243 -0.89290 -0.86261

C -2.59854 -1.46213 0.21280

N 0.12243 0.82187 1.09470

H 4.03114 1.19168 1.13477

H 8.79673 1.05021 -0.36656

H 8.45035 0.19558 1.16854

H 8.18486 1.96470 1.04178

H 5.26159 1.47766 -1.21826

H 6.88222 2.16500 -1.45753

H 5.89602 2.69186 -0.06525

H 5.00604 -2.39304 -1.05482

H -1.94631 1.36759 1.90240

H -3.18544 0.13409 2.24475

H -4.20624 1.98283 0.92393

H -2.94684 1.80844 -0.32733

H -7.13993 0.85525 -1.78023

H -6.32479 -0.73496 -1.96108

H -7.18760 -1.11337 0.22329

H -7.37924 0.70304 1.96459

H -8.41194 1.01654 0.54517

H -6.87458 1.90674 0.74451

H -2.90768 -0.47764 -1.68402

H -4.14919 -1.69065 -1.27149

H -3.19565 -1.90353 1.02595

H -1.96300 -2.26230 -0.18459

**Compound 7c HMC**

O -1.79812 0.92083 -2.45649

C -1.35761 0.57482 -1.34716

C -2.23812 -0.13381 -0.42016

C -3.63659 -0.33604 -0.94377

C -4.59178 -0.88887 0.06880

C -6.03461 -1.01994 -0.30385

N -6.71236 0.11910 -0.57782

C -8.06901 0.04147 -1.07178

C -6.24501 1.43869 -0.21212

O -6.56371 -2.13421 -0.39347

C -4.16346 -1.37047 1.24988

C -2.78456 -1.29540 1.66265

N -2.44949 -1.83681 2.86513

O -3.31816 -2.40321 3.57278

O -1.25871 -1.77526 3.27304

C -1.83926 -0.61144 0.80707

S -0.19109 -0.39810 1.40047

C 0.52649 0.54149 0.07746

N 1.80512 0.92131 0.30338

C 2.62667 0.41718 1.39230

C 3.53244 -0.69981 0.88558

C 4.38347 -0.22140 -0.28416

O 5.10329 -1.30265 -0.86002

C 6.48737 -1.08671 -0.65993

C 6.57779 0.41836 -0.47576

C 7.74644 0.91822 0.33291

O 5.35544 0.68140 0.20565

C 3.52345 0.43566 -1.36185

C 2.60486 1.49789 -0.76883

N -0.05905 0.87085 -1.03549

H -3.60371 -1.01217 -1.82270

H -3.99781 0.61623 -1.36753

H -8.25371 0.88503 -1.75239

H -8.21516 -0.90045 -1.61290

H -8.80247 0.09179 -0.24803

H -5.30481 1.37256 0.34700

H -6.09571 2.06949 -1.10341

H -6.99061 1.92693 0.43641

H -4.86525 -1.81351 1.95713

H 3.22978 1.25695 1.77129

H 2.00528 0.07478 2.22879

H 4.19532 -1.05627 1.68783

H 2.91634 -1.54627 0.54429

H 6.84164 -1.61512 0.24443

H 7.04092 -1.45453 -1.53552

H 6.55934 0.91390 -1.46557

H 7.76686 0.43920 1.32358

H 8.68774 0.68825 -0.18833

H 7.68886 2.00805 0.46673

H 4.18009 0.88224 -2.12386

H 2.92013 -0.34437 -1.85223

H 1.93684 1.91425 -1.52860

H 3.19828 2.31952 -0.33785

**Compound 7c TS**

O 0.63816 3.73079 4.00277

C -0.33425 2.99386 4.19551

C -1.68787 3.58518 4.25339

C -1.75392 5.00138 4.06183

C -2.98118 5.68632 4.31478

C -2.93824 7.17448 4.44058

N -3.91492 7.90670 3.86192

C -4.81261 7.43048 2.82817

C -3.97118 9.32849 4.12625

O -2.02662 7.69038 5.09771

C -4.12228 4.95357 4.50299

C -4.09591 3.54294 4.51148

N -5.33438 2.88040 4.67614

O -6.36440 3.54091 4.76869

O -5.34404 1.64902 4.71321

C -2.85827 2.84282 4.42043

S -2.83186 1.09518 4.57792

C -1.08107 0.77164 4.50188

N -0.76982 -0.52945 4.62108

C 0.61738 -0.96533 4.72670

C 0.88780 -1.51108 6.12285

C -0.08520 -2.62655 6.47512

O 0.13911 -3.03817 7.80263

C -0.05577 -4.43988 7.84226

C 0.48340 -4.88581 6.48800

C 1.97278 -5.16678 6.47567

O 0.15902 -3.76783 5.66215

C -1.52799 -2.16727 6.26676

C -1.71923 -1.60544 4.86469

N -0.13815 1.65629 4.34959

H -0.81999 5.54559 4.20398

H -4.79553 8.14209 1.98795

H -5.84865 7.35713 3.19748

H -4.48997 6.45639 2.44792

H -5.02325 9.64682 4.15995

H -3.49338 9.54883 5.08716

H -3.45865 9.90313 3.33538

H -5.08170 5.43647 4.68798

H 1.27079 -0.11854 4.49923

H 0.78415 -1.74880 3.97001

H 1.91419 -1.90157 6.18154

H 0.78189 -0.70407 6.86486

H 0.50055 -4.84910 8.69607

H -1.12626 -4.68805 7.95274

H -0.07260 -5.75549 6.10369

H 2.32545 -5.31786 5.44473

H 2.19168 -6.07935 7.05122

H 2.53485 -4.33066 6.91880

H -2.21131 -3.01435 6.42538

H -1.75966 -1.39074 7.01251

H -1.53640 -2.38879 4.11145

H -2.75013 -1.26268 4.71909

B -1.71921 5.56823 1.29309

H -1.51533 5.18101 2.52174

H -2.74776 4.96779 0.97982

H -0.71371 5.21591 0.67464

H -1.86168 6.78817 1.37846

**Compound 7c radical anion**

O 1.87154 -1.11969 -2.38496

C 1.42181 -1.00335 -1.25037

C 2.34680 -0.73985 -0.10996

C 3.72035 -0.72475 -0.42176

C 4.67383 -0.48465 0.57457

C 6.13902 -0.55409 0.27791

N 6.59649 0.16023 -0.80412

C 7.96675 -0.00620 -1.20946

C 5.88002 1.23313 -1.44763

O 6.89364 -1.24519 0.94803

C 4.26972 -0.25323 1.88030

C 2.89611 -0.22419 2.21593

N 2.54014 0.07215 3.52228

O 3.42922 0.30843 4.37222

O 1.30464 0.10327 3.80915

C 1.92083 -0.49407 1.20212

S 0.21900 -0.51027 1.65623

C -0.50926 -0.95896 0.10901

N -1.86839 -1.14530 0.17657

C -2.63321 -1.26633 -1.04847

C -3.28503 0.06842 -1.40035

C -4.11611 0.60058 -0.23923

O -4.59856 1.89829 -0.56367

C -5.99238 1.91368 -0.40764

C -6.37685 0.45553 -0.61172

C -7.65425 0.00378 0.05082

O -5.27069 -0.20319 -0.03495

C -3.31161 0.61826 1.05221

C -2.67727 -0.74621 1.30863

N 0.07239 -1.11816 -1.03749

H 3.99538 -0.92759 -1.45685

H 8.02739 -0.10617 -2.30654

H 8.37302 -0.90546 -0.73166

H 8.58875 0.85823 -0.90929

H 5.67461 1.00461 -2.50832

H 4.92347 1.41515 -0.94712

H 6.47906 2.16109 -1.40687

H 4.98800 -0.08563 2.68159

H -3.40941 -2.03566 -0.89851

H -1.95248 -1.59211 -1.84312

H -2.50507 0.80749 -1.64016

H -3.93178 -0.03234 -2.28635

H -6.43200 2.59518 -1.15171

H -6.28342 2.25466 0.60609

H -6.41950 0.23911 -1.69931

H -7.80524 -1.07511 -0.09718

H -8.51916 0.53362 -0.37721

H -7.61715 0.20156 1.13270

H -2.52251 1.38042 0.96189

H -3.97508 0.90803 1.88125

H -3.46756 -1.50263 1.45002

H -2.07809 -0.73450 2.22739

**Compound 7d**

O 1.94780 1.94669 1.41859

C 1.34781 0.90353 1.16686

C 2.12291 -0.26372 0.63885

C 3.51181 -0.12091 0.59907

C 4.34714 -1.13392 0.13425

C 5.84606 -1.03277 0.07858

N 6.35774 0.20900 0.09549

C 7.77106 0.49695 0.04077

C 8.14575 1.26961 -1.21234

O 7.44615 2.49089 -1.32479

O 6.53062 -2.05327 0.02246

C 3.76548 -2.32641 -0.28320

C 2.38565 -2.47799 -0.26770

N 1.87028 -3.75245 -0.73995

O 2.64459 -4.67454 -0.88470

O 0.67414 -3.83130 -0.95874

C 1.52234 -1.45417 0.19098

S -0.21280 -1.68128 0.20316

C -0.73431 -0.21249 1.07150

N -2.03935 -0.20304 1.36670

C -2.68322 0.99023 1.90716

C -3.56714 1.61753 0.83685

C -4.57758 0.62046 0.28568

O -5.54274 0.35359 1.28957

C -6.80631 0.31063 0.65730

C -6.65345 1.34233 -0.44623

C -7.54602 1.16345 -1.64721

O -5.28466 1.16554 -0.81060

C -3.89187 -0.67154 -0.15405

C -3.00095 -1.21643 0.95429

N 0.01614 0.80626 1.38760

H 3.90358 0.82919 0.96783

H 5.72948 1.00313 0.05528

H 8.06096 1.07422 0.93608

H 8.31513 -0.45689 0.06549

H 7.88655 0.67826 -2.10389

H 9.23859 1.43031 -1.21900

H 7.76385 3.09801 -0.64443

H 4.39110 -3.14661 -0.63485

H -1.91239 1.68735 2.24687

H -3.28288 0.67906 2.77664

H -4.10723 2.48268 1.24878

H -2.93979 1.96848 0.00298

H -7.58163 0.55939 1.39419

H -7.00954 -0.69176 0.23536

H -6.78369 2.35569 -0.02125

H -7.42244 0.15882 -2.07960

H -8.59887 1.29284 -1.35393

H -7.31446 1.91149 -2.41925

H -4.65835 -1.41486 -0.41859

H -3.29180 -0.46684 -1.05445

H -2.49434 -2.13575 0.63736

H -3.60480 -1.47666 1.83778

**Compound 7d HMC**

O 1.50178 1.22073 -2.26965

C 1.02823 0.40338 -1.46284

C 1.92855 -0.27783 -0.53143

C 3.37585 0.10671 -0.70224

C 4.32089 -0.64078 0.18897

C 5.77918 -0.44957 0.07209

N 6.19837 0.44488 -0.84418

C 7.58819 0.66316 -1.20324

C 8.46540 1.24920 -0.10529

O 8.86520 0.30663 0.86012

O 6.59258 -1.07987 0.77808

C 3.86379 -1.50347 1.12499

C 2.47691 -1.78949 1.30951

N 2.13262 -2.65331 2.31441

O 3.01123 -3.15907 3.04575

O 0.92431 -2.93552 2.50229

C 1.50911 -1.16834 0.42600

S -0.18828 -1.60655 0.61707

C -0.93445 -0.65271 -0.67994

N -2.26657 -0.83581 -0.79931

C -3.06226 0.00250 -1.68534

C -3.88399 0.99321 -0.86936

C -4.73793 0.27854 0.17395

O -5.77077 -0.46878 -0.45005

C -6.95551 0.29576 -0.36739

C -6.79052 1.01511 0.95989

C -7.53823 2.31646 1.09809

O -5.38223 1.24210 0.98576

C -3.89538 -0.67414 1.01213

C -3.08280 -1.60827 0.12315

N -0.31833 0.17146 -1.47566

H 3.64214 -0.03745 -1.76629

H 3.46893 1.20002 -0.54717

H 5.50260 0.94574 -1.38232

H 7.58294 1.35606 -2.05552

H 8.03595 -0.28431 -1.55365

H 9.37697 1.65200 -0.57684

H 7.93329 2.10270 0.35954

H 8.10038 -0.30154 0.98093

H 4.57426 -2.02005 1.76946

H -3.72754 -0.65720 -2.26526

H -2.39367 0.51752 -2.38128

H -4.54054 1.57807 -1.53115

H -3.21374 1.69619 -0.35018

H -7.81968 -0.38143 -0.39984

H -7.02490 1.01859 -1.20214

H -7.06035 0.32946 1.78548

H -7.27482 3.00487 0.28072

H -7.30541 2.80097 2.05746

H -8.62264 2.13114 1.06599

H -3.21483 -0.07665 1.63859

H -4.55757 -1.25139 1.67430

H -3.75320 -2.24696 -0.47399

H -2.46380 -2.28258 0.72673

**Compound 7d TS**

O -0.44219 3.30882 4.18742

C -1.54495 2.76967 4.32523

C -2.76587 3.60145 4.27714

C -2.55524 4.99245 3.98233

C -3.63698 5.90930 4.19006

C -3.44260 7.38552 4.12932

N -2.40614 7.79498 3.37309

C -2.04828 9.18180 3.20483

C -0.87292 9.60456 4.07234

O 0.31030 8.89140 3.78523

O -4.19750 8.16061 4.72349

C -4.89876 5.41415 4.39254

C -5.14372 4.03046 4.46425

N -6.49019 3.62186 4.61427

O -7.37876 4.46674 4.62234

O -6.73142 2.41941 4.72953

C -4.05683 3.10504 4.44747

S -4.36655 1.39680 4.69804

C -2.71169 0.74010 4.65176

N -2.66219 -0.59541 4.79105

C -1.39296 -1.28341 4.99391

C -1.28318 -1.73098 6.44730

C -2.47338 -2.58438 6.86504

O -2.38210 -3.83882 6.21105

C -2.78170 -4.82483 7.14073

C -2.27337 -4.26105 8.45686

C -3.01362 -4.70676 9.69229

O -2.44085 -2.85876 8.25280

C -3.79254 -1.89445 6.52284

C -3.80854 -1.44812 5.06730

N -1.61249 1.42310 4.51261

H -1.53376 5.34072 4.14585

H -1.91300 7.10754 2.80387

H -1.80450 9.35381 2.14357

H -2.92039 9.80469 3.45175

H -1.10532 9.41201 5.13090

H -0.72132 10.69348 3.95497

H 0.59277 9.10820 2.88764

H -5.73863 6.09834 4.51423

H -0.57607 -0.60835 4.72296

H -1.36744 -2.15142 4.31642

H -0.35942 -2.30841 6.60049

H -1.24768 -0.84614 7.10181

H -2.32239 -5.78293 6.86291

H -3.88209 -4.93831 7.15726

H -1.19392 -4.48157 8.55881

H -4.08843 -4.48716 9.60265

H -2.88661 -5.79061 9.83566

H -2.62246 -4.19822 10.58548

H -4.62494 -2.58718 6.71658

H -3.91581 -1.02286 7.18452

H -4.74808 -0.94019 4.81945

H -3.73817 -2.32178 4.40012

B -1.95165 5.30464 1.24927

H -2.30594 5.00885 2.48499

H -1.07247 6.16011 1.36235

H -3.00128 5.71857 0.76314

H -1.54833 4.23259 0.80825

**Compound 7d radical anion**

O 1.55669 -0.40054 -2.58991

C 1.10558 0.12677 -1.57843

C 1.97682 0.27785 -0.37628

C 3.29009 -0.20687 -0.51059

C 4.20178 -0.11715 0.55584

C 5.59935 -0.62603 0.46969

N 5.95643 -1.21641 -0.69817

C 7.21601 -1.89742 -0.90774

C 8.46750 -1.02577 -0.80409

O 8.90840 -0.81508 0.49997

O 6.39888 -0.52224 1.40254

C 3.79798 0.45560 1.75079

C 2.48409 0.94455 1.91846

N 2.13420 1.49594 3.14344

O 2.97966 1.56591 4.05966

O 0.94812 1.91416 3.28336

C 1.55529 0.85292 0.82901

S -0.07707 1.47141 1.05146

C -0.75821 1.14204 -0.54457

N -2.05769 1.56305 -0.68594

C -2.81983 1.11400 -1.83589

C -3.62396 -0.13053 -1.46873

C -4.50918 0.12486 -0.25736

O -5.55187 1.01997 -0.64430

C -6.75981 0.51400 -0.14381

C -6.52409 -0.98882 -0.15928

C -7.34191 -1.80088 0.81382

O -5.15404 -1.05912 0.17625

C -3.71324 0.71468 0.90058

C -2.90148 1.92090 0.43555

N -0.19065 0.56570 -1.55725

H 3.52048 -0.63202 -1.48796

H 5.24879 -1.28887 -1.41485

H 7.32178 -2.73475 -0.19288

H 7.17014 -2.33244 -1.91730

H 8.27210 -0.07249 -1.33996

H 9.28253 -1.53736 -1.34621

H 8.10612 -0.63859 1.03826

H 4.48583 0.53719 2.59116

H -2.11844 0.90022 -2.64987

H -3.49624 1.92844 -2.14252

H -2.92941 -0.94898 -1.22487

H -4.25639 -0.45568 -2.30913

H -6.95409 0.86594 0.88957

H -7.58390 0.84373 -0.79360

H -6.67736 -1.36949 -1.19000

H -7.19369 -1.43550 1.84119

H -7.04332 -2.85830 0.77950

H -8.41259 -1.73800 0.56464

H -4.41072 0.99572 1.70506

H -3.03883 -0.05902 1.29942

H -2.30206 2.33218 1.25721

H -3.58438 2.72046 0.10396

**Compound 7e**

O 1.58118 -2.96118 -0.49296

C 1.05888 -1.84864 -0.48738

C 1.91931 -0.64618 -0.24608

C 3.29810 -0.85035 -0.26272

C 4.19936 0.19034 -0.04427

C 5.64839 -0.05452 -0.07237

N 6.20815 -1.18160 -0.26993

C 7.64741 -0.95423 -0.21420

C 7.79600 0.55608 0.05387

O 6.43268 1.01967 0.12904

C 3.69998 1.46416 0.20714

C 2.32660 1.67887 0.23704

N 1.89399 3.03624 0.52110

O 2.72024 3.92422 0.50627

O 0.71154 3.21523 0.75536

C 1.39739 0.63901 0.00169

S -0.32396 0.95537 0.00476

C -0.94788 -0.60419 -0.59814

N -2.25241 -0.57967 -0.89369

C -2.97765 -1.80090 -1.22792

C -3.97080 -2.13320 -0.12103

C -4.90912 -0.97291 0.16148

O -5.79312 -0.82322 -0.92958

C -6.97784 -0.26429 -0.40214

C -7.07830 -0.88950 0.99106

C -7.63131 0.03164 2.05270

O -5.72737 -1.26615 1.27421

C -4.11758 0.31510 0.40459

C -3.13388 0.56813 -0.72888

N -0.26744 -1.70999 -0.72025

H 3.66321 -1.86050 -0.45089

H 8.11436 -1.26258 -1.16309

H 8.09526 -1.57045 0.58157

H 8.29792 1.10099 -0.75773

H 8.28953 0.78882 1.00765

H 4.36947 2.30388 0.38899

H -2.25831 -2.61177 -1.37200

H -3.50185 -1.62693 -2.18096

H -4.56922 -3.01200 -0.40289

H -3.42665 -2.37123 0.80587

H -7.81708 -0.52929 -1.05859

H -6.90751 0.83726 -0.34109

H -7.67672 -1.81628 0.94714

H -7.63575 -0.46114 3.03589

H -7.03129 0.95201 2.12021

H -8.66848 0.30826 1.80650

H -3.57654 0.21695 1.35831

H -4.80379 1.16930 0.49781

H -3.67363 0.70760 -1.67906

H -2.55854 1.48504 -0.55541

**Compound 7e HMC**

O 1.69740 1.12551 2.76546

C 1.34438 0.52399 1.73730

C 2.35076 0.18073 0.73151

C 3.75199 0.58187 1.11151

C 4.80194 0.11225 0.15572

C 6.19041 0.34628 0.51036

N 6.59617 0.94896 1.56791

C 8.05298 0.92679 1.54176

C 8.40583 0.34212 0.16368

O 7.14038 -0.11144 -0.34161

C 4.47964 -0.49869 -1.00841

C 3.12501 -0.76163 -1.38871

N 2.90589 -1.31986 -2.61658

O 1.73116 -1.56806 -2.98764

O 3.86624 -1.58132 -3.37633

C 2.06010 -0.44281 -0.45779

S 0.41636 -0.91054 -0.89484

C -0.47291 -0.41986 0.55834

N -1.78711 -0.73192 0.52963

C -2.70483 -0.18114 1.51689

C -3.51709 0.94461 0.88538

C -4.24613 0.46803 -0.36407

O -4.88203 1.55145 -1.02674

C -6.28337 1.36467 -0.96177

C -6.42101 -0.13670 -0.77623

C -7.67352 -0.60764 -0.08412

O -5.27811 -0.41560 0.02582

C -3.28508 -0.21648 -1.33384

C -2.47716 -1.29327 -0.62010

N 0.02456 0.20121 1.58694

H 3.96840 0.21160 2.12877

H 3.80112 1.68256 1.22165

H 8.46336 1.93871 1.68409

H 8.43286 0.30641 2.37178

H 8.80715 1.09432 -0.53311

H 9.09307 -0.51379 0.20409

H 5.25924 -0.81655 -1.69910

H -2.12964 0.17698 2.37590

H -3.36732 -0.99319 1.85610

H -4.25536 1.33800 1.59999

H -2.84257 1.76611 0.59786

H -6.71395 1.90822 -0.10052

H -6.73995 1.73494 -1.89056

H -6.31374 -0.64204 -1.75546

H -7.65199 -1.69751 0.05991

H -7.78158 -0.12225 0.89777

H -8.55439 -0.36225 -0.69605

H -3.86026 -0.65726 -2.16185

H -2.60888 0.54291 -1.75719

H -3.14864 -2.08414 -0.24932

H -1.76988 -1.77737 -1.30422

**Compound 7e TS**

O -5.66475 3.32849 2.81593

C -4.80999 2.50500 3.15505

C -4.29711 1.54435 2.15331

C -4.81082 1.70047 0.83013

C -4.54471 0.69398 -0.14284

C -5.23454 0.73564 -1.42788

N -6.09257 1.61396 -1.78224

C -6.54273 1.25109 -3.12057

C -5.64856 0.06455 -3.52218

O -4.93242 -0.23939 -2.31105

C -3.59287 -0.26135 0.12594

C -2.94619 -0.30916 1.37173

N -1.95336 -1.30985 1.54099

O -1.67575 -2.04522 0.60165

O -1.39463 -1.40293 2.63284

C -3.32309 0.58120 2.42079

S -2.59938 0.41314 4.00983

C -3.45104 1.69303 4.91083

N -3.06805 1.78962 6.19444

C -3.72995 2.71194 7.10915

C -4.57004 1.92802 8.10896

C -3.72925 0.89837 8.85235

O -2.83617 1.58555 9.71629

C -2.84247 0.91526 10.96015

C -4.26541 0.39242 11.04565

C -4.47136 -0.82280 11.91296

O -4.53066 0.07930 9.67943

C -2.95598 0.01741 7.87524

C -2.17538 0.86523 6.87876

N -4.35793 2.49869 4.43821

H -5.71648 2.29750 0.72940

H -6.44281 2.10412 -3.80926

H -7.61221 0.98254 -3.09233

H -4.91304 0.32059 -4.29955

H -6.20483 -0.83060 -3.83064

H -3.33337 -1.01267 -0.61859

H -4.34527 3.40499 6.52873

H -2.94778 3.28812 7.62860

H -5.03000 2.61061 8.83885

H -5.37525 1.39804 7.57681

H -2.58501 1.63117 11.75246

H -2.11489 0.08186 10.96889

H -4.93909 1.20947 11.36704

H -5.51417 -1.16769 11.85959

H -3.81039 -1.64388 11.59602

H -4.24840 -0.57666 12.96215

H -3.66922 -0.62922 7.34054

H -2.26730 -0.62832 8.44030

H -1.41422 1.46468 7.40314

H -1.63876 0.23065 6.16394

B -3.07979 3.78554 -0.06120

H -4.06067 2.99411 0.25910

H -2.71929 4.26715 1.01503

H -3.54833 4.61004 -0.84940

H -2.25227 3.02458 -0.56741

**Compound 7e radical anion**

O 1.25516 -2.74437 1.39674

C 0.84640 -1.63136 1.09489

C 1.78902 -0.61837 0.53004

C 3.11343 -1.04838 0.33954

C 4.07238 -0.16915 -0.18713

C 5.44552 -0.63350 -0.39488

N 5.90949 -1.79169 -0.12025

C 7.29670 -1.80850 -0.53804

C 7.58776 -0.35900 -0.97721

O 6.30951 0.26287 -0.94818

C 3.71844 1.12980 -0.52072

C 2.39653 1.58885 -0.33763

N 2.10147 2.89759 -0.68962

O 3.00061 3.63751 -1.15379

O 0.91217 3.30360 -0.52117

C 1.41238 0.69163 0.19244

S -0.23143 1.28606 0.40493

C -1.00997 -0.16866 1.03373

N -2.34817 -0.00031 1.30665

C -3.13254 1.08719 0.75845

C -3.77890 0.65788 -0.55650

C -4.61519 -0.59835 -0.35758

O -5.10689 -1.09231 -1.59614

C -6.48503 -0.84404 -1.66033

C -6.89736 -0.83926 -0.19587

C -8.13677 -0.04964 0.14429

O -5.76536 -0.25229 0.40765

C -3.81694 -1.69389 0.33669

C -3.15172 -1.16950 1.60624

N -0.48160 -1.32677 1.26706

H 3.37236 -2.07105 0.60827

H 7.95095 -2.13852 0.28612

H 7.43413 -2.53409 -1.36030

H 8.25643 0.17664 -0.28215

H 8.00007 -0.27324 -1.99408

H 4.43741 1.83630 -0.93083

H -2.51228 1.98091 0.61670

H -3.91266 1.35023 1.49163

H -2.99130 0.44007 -1.29421

H -4.42006 1.45470 -0.96339

H -6.96646 -1.63575 -2.25392

H -6.70163 0.13700 -2.12908

H -7.00981 -1.88680 0.15290

H -9.02031 -0.48829 -0.34473

H -8.30977 -0.05059 1.23003

H -8.03024 0.99456 -0.18649

H -3.04299 -2.05985 -0.35554

H -4.49007 -2.53602 0.56225

H -3.91993 -0.87793 2.34159

H -2.49822 -1.93121 2.04609

**Compound 7f**

O 1.31673 2.45913 -1.92940

C 1.04450 1.55254 -1.14512

C 2.09632 0.53864 -0.81931

C 3.39170 0.81547 -1.26128

C 4.45574 -0.04679 -1.01642

C 5.79950 0.30926 -1.57898

N 6.87901 0.10893 -0.79011

C 8.24229 0.32145 -1.20379

O 6.70695 -0.33393 0.49358

C 6.57959 0.73995 1.42224

O 5.89547 0.76710 -2.71510

C 4.20320 -1.24107 -0.34929

C 2.91265 -1.54465 0.06798

N 2.73580 -2.82625 0.72883

O 1.59493 -3.20699 0.92596

O 3.72093 -3.45865 1.04812

C 1.82656 -0.65961 -0.13452

S 0.22477 -1.04774 0.45764

C -0.61451 0.50153 0.16962

N -1.82052 0.57398 0.74455

C -2.71846 1.69353 0.47688

C -3.90055 1.21160 -0.35361

C -4.62070 0.05082 0.32280

O -5.27412 0.54070 1.48810

C -6.60913 0.07571 1.46857

C -6.89675 -0.04279 -0.01690

C -7.97667 -1.01635 -0.40992

O -5.63032 -0.48272 -0.50409

C -3.64138 -1.05752 0.69407

C -2.46808 -0.49855 1.48808

N -0.17404 1.48991 -0.55871

H 3.55244 1.74474 -1.80984

H 8.68438 1.19105 -0.69333

H 8.83349 -0.57683 -0.97488

H 8.24715 0.50107 -2.28429

H 5.67921 1.33676 1.20563

H 6.48076 0.26462 2.40623

H 7.47263 1.38334 1.40974

H 4.99431 -1.96306 -0.15559

H -2.16072 2.47921 -0.04000

H -3.05900 2.08673 1.44778

H -4.61219 2.03552 -0.51051

H -3.54337 0.87126 -1.33799

H -6.69478 -0.90902 1.96518

H -7.24885 0.80304 1.98705

H -7.11662 0.96029 -0.42988

H -8.94534 -0.68211 -0.00889

H -8.06513 -1.07866 -1.50421

H -7.75862 -2.02002 -0.01429

H -4.16900 -1.81725 1.28937

H -3.27750 -1.53528 -0.22907

H -2.82191 -0.07241 2.44016

H -1.75292 -1.28962 1.74207

**Compound 7f HMC**

O 1.24561 -2.86700 1.38758

C 0.91559 -1.73935 0.98420

C 1.93271 -0.86330 0.40231

C 3.32070 -1.44822 0.41634

C 4.38967 -0.48808 0.00142

C 5.78414 -0.98890 0.05799

N 6.75394 -0.11445 0.45439

C 6.62985 1.07553 1.26384

O 8.04101 -0.58882 0.40676

C 8.65487 -0.29178 -0.83923

O 6.05534 -2.14240 -0.27916

C 4.08823 0.71108 -0.53936

C 2.73598 1.17207 -0.68750

N 2.53419 2.35763 -1.32904

O 1.36550 2.80461 -1.46455

O 3.50412 3.00545 -1.78890

C 1.66151 0.36975 -0.13996

S 0.03169 1.04393 -0.16777

C -0.86900 -0.21759 0.69446

N -2.17070 0.07973 0.90444

C -2.85753 1.21941 0.31947

C -3.68202 0.77218 -0.88239

C -4.65269 -0.33679 -0.49596

O -5.30375 -0.86168 -1.64420

C -6.68538 -0.56658 -1.55897

C -6.90502 -0.37918 -0.06760

C -8.05935 0.50367 0.32874

O -5.67035 0.21862 0.31291

C -3.93406 -1.46867 0.23556

C -3.09514 -0.93478 1.39079

N -0.39095 -1.35493 1.10538

H 3.34991 -2.34893 -0.22677

H 3.53045 -1.84190 1.42687

H 5.57879 1.24043 1.51942

H 7.01465 1.95977 0.73302

H 7.20619 0.93175 2.19067

H 8.12918 -0.79467 -1.66628

H 9.68033 -0.67718 -0.76670

H 8.68039 0.79545 -1.01643

H 4.87003 1.38057 -0.89910

H -3.51180 1.64984 1.09403

H -2.14550 2.00537 0.03999

H -4.25143 1.61731 -1.29661

H -3.01148 0.38919 -1.66744

H -7.26044 -1.40119 -1.98444

H -6.92427 0.36116 -2.11110

H -6.99898 -1.36899 0.41907

H -9.00728 0.04712 0.00695

H -8.09547 0.62901 1.42054

H -7.96991 1.49579 -0.13940

H -4.67949 -2.18857 0.60601

H -3.28530 -1.99375 -0.48303

H -3.74218 -0.46318 2.14732

H -2.52518 -1.73517 1.87170

**Compound 7f TS**

O -0.75875 1.22365 1.80829

C -1.77590 0.55745 1.59102

C -3.05449 0.96381 2.21255

C -3.04928 2.23276 2.87471

C -4.14943 2.59435 3.71330

C -3.92718 3.75937 4.61786

N -4.96214 4.58967 4.89763

C -4.88808 5.69704 5.81510

O -6.18469 4.40117 4.31236

C -6.30070 5.11025 3.08129

O -2.80666 3.97021 5.08821

C -5.29211 1.83606 3.66525

C -5.36814 0.68010 2.86413

N -6.60071 -0.01492 2.86345

O -6.67591 -1.07932 2.24771

O -7.56044 0.45601 3.46505

C -4.22828 0.21698 2.14135

S -4.32017 -1.27643 1.22444

C -2.69282 -1.32680 0.49787

N -2.52711 -2.32463 -0.38548

C -1.21880 -2.61375 -0.96091

C -0.71271 -3.95438 -0.44363

C -1.71318 -5.07059 -0.71138

O -1.75642 -5.31284 -2.10882

C -1.80701 -6.71268 -2.29356

C -0.97620 -7.22693 -1.13109

C -1.28743 -8.62743 -0.66916

O -1.30092 -6.28286 -0.11177

C -3.09698 -4.69587 -0.18836

C -3.52344 -3.33252 -0.71547

N -1.70444 -0.52346 0.76789

H -2.06727 2.63933 3.11654

H -5.02274 6.65462 5.28853

H -5.66895 5.59190 6.58306

H -3.90079 5.68537 6.28820

H -5.56990 4.73643 2.34707

H -7.31987 4.91412 2.72435

H -6.16592 6.19225 3.23554

H -6.16078 2.08617 4.26970

H -0.53309 -1.80217 -0.70207

H -1.33084 -2.63911 -2.05676

H 0.24454 -4.20914 -0.92216

H -0.54913 -3.89186 0.64339

H -1.38924 -6.95740 -3.27939

H -2.84619 -7.08748 -2.23269

H 0.09862 -7.13567 -1.37815

H -1.04833 -9.34728 -1.46661

H -0.68752 -8.88874 0.21474

H -2.35377 -8.72512 -0.41460

H -3.82186 -5.46257 -0.49987

H -3.06775 -4.68324 0.91244

H -4.50907 -3.05416 -0.32485

H -3.61503 -3.36290 -1.81328

B -3.47050 3.81493 0.58758

H -3.10065 3.28057 1.72115

H -2.65143 3.38507 -0.22486

H -4.60939 3.37485 0.43165

H -3.41637 5.03120 0.77423

**Compound 7f radical anion**

O 1.56100 2.30671 -1.93998

C 1.15828 1.51720 -1.09583

C 2.07797 0.47533 -0.55173

C 3.33412 0.36866 -1.17810

C 4.28538 -0.54367 -0.70837

C 5.58581 -0.60910 -1.44126

N 6.74104 -0.61437 -0.69726

C 8.04715 -0.76123 -1.26739

O 6.71275 -0.63319 0.67356

C 6.57410 0.66411 1.21816

O 5.65213 -0.59968 -2.66219

C 3.98811 -1.36579 0.37000

C 2.72768 -1.29399 1.00554

N 2.48735 -2.12785 2.08630

O 3.36902 -2.93960 2.45748

O 1.36882 -2.02676 2.67826

C 1.75397 -0.35887 0.52758

S 0.18357 -0.30276 1.32566

C -0.63625 0.88736 0.30614

N -1.95275 1.08822 0.64451

C -2.71251 0.17293 1.47029

C -3.48829 -0.81397 0.60113

C -4.36630 -0.07322 -0.39967

O -4.97402 -1.01645 -1.27285

C -6.35966 -0.80038 -1.27202

C -6.60708 -0.18470 0.09703

C -7.82902 0.69043 0.22535

O -5.43693 0.58576 0.26643

C -3.56868 0.96403 -1.17565

C -2.79189 1.88034 -0.23404

N -0.14506 1.57884 -0.67110

H 3.54113 1.01942 -2.02668

H 7.92622 -0.80079 -2.35642

H 8.69419 0.09284 -1.00569

H 8.52234 -1.68981 -0.90949

H 7.40450 1.32084 0.90434

H 5.61015 1.11278 0.92863

H 6.59855 0.53227 2.30802

H 4.69711 -2.08620 0.77214

H -2.05465 -0.35642 2.17033

H -3.41786 0.76607 2.07613

H -4.12104 -1.46975 1.21952

H -2.78433 -1.45479 0.04768

H -6.87460 -1.76056 -1.42634

H -6.66247 -0.09775 -2.07419

H -6.64006 -0.99201 0.85759

H -7.80081 1.50179 -0.51755

H -8.74479 0.09921 0.07066

H -7.87752 1.14174 1.22667

H -2.85964 0.43592 -1.83128

H -4.25999 1.54000 -1.81007

H -3.49009 2.46643 0.38670

H -2.14618 2.56422 -0.79649

**Compound 8**

O 1.57501 2.65210 -1.78824

C 1.27109 1.60372 -1.22189

C 2.31911 0.85354 -0.46458

C 3.65203 1.21571 -0.68374

C 4.71118 0.58908 -0.03344

C 6.15978 1.00188 -0.29501

O 7.03845 0.36684 0.33045

O 6.33176 1.93528 -1.11007

C 4.41494 -0.42492 0.87037

C 3.09486 -0.78917 1.11491

N 2.87755 -1.84475 2.08556

O 3.81641 -2.54417 2.40694

O 1.75234 -1.97477 2.53892

C 2.01166 -0.17105 0.44780

S 0.36134 -0.68767 0.73958

C -0.46910 0.12442 -0.61445

N -1.71022 -0.32528 -0.83605

C -2.59041 0.32621 -1.79968

C -3.76108 0.97913 -1.07691

C -4.50775 -0.01447 -0.20117

O -5.19644 -0.92507 -1.03300

C -6.39600 -1.26054 -0.36246

C -6.77853 0.05606 0.30599

C -7.62419 0.96707 -0.56024

O -5.50016 0.63790 0.56927

C -3.54114 -0.75142 0.72586

C -2.38677 -1.35578 -0.06107

N 0.01343 1.10697 -1.32300

H 3.86024 2.01976 -1.39068

H 5.22118 -0.93231 1.39997

H -2.01165 1.06282 -2.36410

H -2.94714 -0.44590 -2.50022

H -3.39055 1.79331 -0.43532

H -4.46304 1.40851 -1.80661

H -6.22511 -2.05843 0.38189

H -7.12857 -1.60833 -1.10284

H -7.27253 -0.11839 1.27501

H -7.16654 1.10049 -1.55241

H -7.73055 1.95519 -0.08865

H -8.62993 0.53923 -0.69241

H -3.15659 -0.03891 1.47215

H -4.07961 -1.54482 1.26504

H -1.68499 -1.86994 0.60568

H -2.76299 -2.11035 -0.77067

**Compound 8 HMC**

O 1.78688 3.14219 0.23725

C 1.36872 1.97651 0.34589

C 2.27921 0.86297 0.08243

C 3.66697 1.29021 -0.31870

C 4.59397 0.17159 -0.66820

C 5.98547 0.51181 -1.15563

O 6.26511 1.73774 -1.23445

O 6.76257 -0.43363 -1.44937

C 4.20063 -1.11272 -0.55336

C 2.88735 -1.49821 -0.10272

N 2.61174 -2.82395 0.01736

O 3.48482 -3.68811 -0.25090

O 1.46891 -3.19834 0.40370

C 1.92150 -0.46064 0.19457

S 0.29906 -0.95525 0.68351

C -0.48652 0.62015 0.89415

N -1.77671 0.54231 1.29633

C -2.54544 -0.69074 1.34242

C -3.41796 -0.81201 0.09844

C -4.31501 0.40756 -0.06321

O -5.31651 0.36628 0.93460

C -6.49483 0.88952 0.35386

C -6.41025 0.37649 -1.07940

C -7.01286 -0.99945 -1.28028

O -5.00018 0.36281 -1.30395

C -3.50779 1.70247 0.02846

C -2.62930 1.72231 1.27446

N 0.06155 1.78327 0.70340

H 4.10320 1.90925 0.48847

H 3.59504 1.99986 -1.16223

H 4.89920 -1.90791 -0.81081

H -3.17213 -0.65864 2.24763

H -1.88704 -1.56129 1.44986

H -2.77896 -0.89147 -0.79479

H -4.04821 -1.71188 0.15183

H -7.36167 0.50763 0.90952

H -6.49896 1.99351 0.38836

H -6.85352 1.09515 -1.78678

H -6.77874 -1.37767 -2.28638

H -8.10791 -0.95352 -1.17638

H -6.62059 -1.71213 -0.53879

H -2.87863 1.78502 -0.87171

H -4.19706 2.56052 0.03411

H -2.00109 2.61752 1.30508

H -3.25303 1.70329 2.18212

**Compound 8 TS**

O 3.44212 -0.47594 0.19832

C 2.27651 -0.07614 0.29373

C 1.91833 1.25674 -0.22942

C 2.94488 1.93938 -0.96500

C 2.79894 3.33490 -1.24161

C 3.97701 4.14234 -1.75247

O 3.79570 5.37010 -1.94022

O 5.04324 3.50869 -1.94790

C 1.56105 3.89875 -1.08820

C 0.46611 3.16313 -0.58415

N -0.77954 3.82011 -0.51682

O -0.88710 4.96520 -0.95015

O -1.74017 3.22183 -0.02255

C 0.65411 1.83053 -0.10722

S -0.68117 0.97784 0.64915

C 0.09430 -0.57154 1.05694

N -0.74246 -1.45729 1.62829

C -0.31436 -2.82672 1.88336

C -0.90938 -3.75282 0.82913

C -2.42503 -3.63311 0.77724

O -2.96242 -4.21139 1.94931

C -4.19175 -4.80612 1.58249

C -3.89534 -5.33858 0.18460

C -3.31192 -6.73673 0.16537

O -2.95324 -4.38150 -0.30348

C -2.85780 -2.17167 0.64592

C -2.18736 -1.30468 1.70442

N 1.34235 -0.88604 0.86791

H 3.95694 1.55198 -0.84699

H 1.41322 4.94948 -1.33603

H 0.77874 -2.86536 1.87419

H -0.66735 -3.10666 2.88820

H -0.50746 -3.48852 -0.16116

H -0.64364 -4.79908 1.04031

H -5.00518 -4.05914 1.56574

H -4.43671 -5.58955 2.31196

H -4.78842 -5.28689 -0.45815

H -2.45238 -6.81260 0.84885

H -2.97552 -6.99802 -0.84880

H -4.07210 -7.47025 0.47541

H -2.58226 -1.81415 -0.35860

H -3.95198 -2.10285 0.73868

H -2.48051 -0.25377 1.59523

H -2.50324 -1.61580 2.71255

B 2.45527 0.66863 -3.41265

H 2.93420 1.27671 -2.35485

H 2.25740 -0.48383 -3.02785

H 1.42544 1.29981 -3.65165

H 3.33026 0.77877 -4.27292

**Compound 8 radical anion**

C -7.66056 0.69069 0.42724

C -6.40851 -0.05506 0.81801

C -6.20026 -1.41105 0.16055

O -4.80859 -1.57187 0.21612

C -4.20798 -0.28590 0.17364

C -3.57511 -0.01169 -1.18294

C -2.80219 1.30462 -1.17505

N -1.81893 1.29105 -0.10805

C -0.54285 0.86474 -0.39342

N -0.20743 0.73564 -1.63705

C 1.03862 0.36867 -2.07794

O 1.25193 0.26902 -3.27917

C 2.13505 0.08723 -1.10265

C 3.38377 -0.24029 -1.66113

C 4.47560 -0.52612 -0.82571

C 5.80572 -0.86362 -1.37213

O 6.80196 -1.10201 -0.72493

O 5.84176 -0.89003 -2.72157

C 4.32726 -0.50025 0.55345

C 3.08402 -0.18816 1.13922

N 2.98532 -0.19602 2.52329

O 3.98863 -0.46938 3.21896

O 1.85926 0.07600 3.03635

C 1.96860 0.12437 0.28965

S 0.43646 0.55229 1.04354

C -2.42296 1.14344 1.20054

C -3.18403 -0.17691 1.29577

O -5.26662 0.64582 0.37457

H -7.70263 0.82693 -0.66398

H -8.55604 0.13810 0.75114

H -7.68138 1.68356 0.89880

H -6.37051 -0.17686 1.92025

H -6.56861 -1.39722 -0.88502

H -6.67985 -2.24354 0.69653

H -4.36515 -0.00257 -1.94965

H -2.88060 -0.83328 -1.41598

H -2.27582 1.45317 -2.12442

H -3.48850 2.14987 -1.00272

H 3.46492 -0.26060 -2.74594

H 6.75288 -1.11669 -2.95223

H 5.16241 -0.72329 1.21610

H -1.66304 1.21634 1.98866

H -3.12063 1.98431 1.34821

H -2.47959 -1.01954 1.21705

H -3.70311 -0.26377 2.26298

**Compound 9**

O -2.10226 2.49542 -2.08430

C -1.67397 1.53071 -1.45630

C -2.56691 0.87714 -0.44442

C -3.74705 1.54998 -0.13861

C -4.66255 1.02846 0.77830

C -5.85902 1.74828 1.09211

N -6.82496 2.33805 1.34238

C -4.40150 -0.20028 1.38292

C -3.23034 -0.87732 1.07450

N -3.04002 -2.15977 1.73753

O -2.08067 -2.82902 1.40049

O -3.84070 -2.50154 2.58033

C -2.26957 -0.35375 0.17391

S -0.77491 -1.20562 -0.13497

C 0.09292 -0.00260 -1.13258

N 1.36701 -0.32915 -1.36929

C 2.08358 -1.43333 -0.74472

C 3.06156 -0.89732 0.29210

C 4.00630 0.13032 -0.32365

O 4.90547 -0.50145 -1.22433

C 6.16328 -0.59268 -0.58542

C 6.16853 0.63567 0.30635

C 7.05776 0.57190 1.52066

O 4.79807 0.69668 0.69824

C 3.23478 1.20242 -1.08479

C 2.25811 0.58566 -2.07722

N -0.41207 1.08470 -1.64447

H -3.93729 2.49957 -0.64093

H -5.09906 -0.64348 2.09355

H 2.61993 -1.97083 -1.54197

H 1.39011 -2.15182 -0.29233

H 2.50821 -0.42163 1.11688

H 3.65553 -1.72336 0.71016

H 6.95257 -0.58925 -1.34941

H 6.23953 -1.51741 0.01697

H 6.41228 1.52878 -0.30028

H 6.93732 1.47384 2.13804

H 6.81855 -0.31115 2.13250

H 8.11174 0.51020 1.21034

H 2.68047 1.81440 -0.35643

H 3.94881 1.85377 -1.60989

H 1.65772 1.35023 -2.57795

H 2.79531 0.00155 -2.84047

**Compound 9 HMC**

O -1.51152 3.09825 -1.55338

C -1.29438 2.09233 -0.85892

C -2.41342 1.20697 -0.52523

C -3.73456 1.68069 -1.07608

C -4.89548 0.82670 -0.65364

C -6.19845 1.23944 -1.03256

N -7.25248 1.61153 -1.36070

C -4.73375 -0.31509 0.06333

C -3.45124 -0.76686 0.48867

N -3.39317 -1.95915 1.16497

O -2.28504 -2.39393 1.55379

O -4.43398 -2.61126 1.38683

C -2.28504 0.05044 0.20242

S -0.73235 -0.48018 0.84699

C 0.32733 0.83918 0.31387

N 1.60389 0.71946 0.73482

C 2.65297 1.57453 0.19738

C 3.52415 0.78201 -0.77143

C 4.08287 -0.47454 -0.11197

O 5.03250 -0.12923 0.88874

C 6.32039 -0.39189 0.37055

C 6.06804 -1.56724 -0.55581

C 7.05725 -1.75239 -1.67698

O 4.78191 -1.23515 -1.07504

C 2.97270 -1.29182 0.53935

C 2.13170 -0.42181 1.46497

N -0.02161 1.85009 -0.42688

H -3.89546 2.72923 -0.76962

H -3.67510 1.72693 -2.17987

H -5.59400 -0.92411 0.34127

H 2.19096 2.43490 -0.29579

H 3.25789 1.93972 1.04254

H 4.36237 1.40265 -1.12319

H 2.92983 0.48450 -1.64979

H 6.70927 0.47685 -0.19349

H 7.00061 -0.62090 1.20227

H 5.98994 -2.49563 0.04180

H 7.13723 -0.83685 -2.28265

H 6.75466 -2.58344 -2.33047

H 8.04959 -1.98894 -1.26422

H 2.33540 -1.70635 -0.25726

H 3.41980 -2.12826 1.09650

H 2.74969 -0.03312 2.29003

H 1.32296 -1.00160 1.92571

**Compound 9 TS**

O 1.68744 0.92877 3.13077

C 1.27448 0.37792 2.10720

C 2.21145 -0.44249 1.30360

C 3.51814 -0.58721 1.85278

C 4.54405 -1.15416 1.04195

C 5.89350 -1.12422 1.49606

N 6.98590 -1.09067 1.88904

C 4.20976 -1.79439 -0.13472

C 2.88475 -1.81030 -0.58082

N 2.62765 -2.52243 -1.78948

O 1.48010 -2.53389 -2.22272

O 3.54812 -3.09843 -2.34950

C 1.86468 -1.10033 0.12399

S 0.23672 -1.04209 -0.52252

C -0.57605 -0.01976 0.69242

N -1.88276 0.16337 0.45213

C -2.59228 -0.27850 -0.73964

C -2.95201 0.91707 -1.61204

C -3.74351 1.95610 -0.82079

O -5.03513 1.46298 -0.50343

C -5.93774 2.00792 -1.44397

C -5.33956 3.37670 -1.71738

C -5.67253 3.98553 -3.05551

O -3.94260 3.10000 -1.62780

C -3.03097 2.31301 0.47615

C -2.67865 1.06588 1.27657

N -0.02295 0.52831 1.73589

H 3.78502 0.10364 2.65248

H 4.96951 -2.28338 -0.74433

H -3.50538 -0.79783 -0.40799

H -2.00211 -1.00940 -1.30479

H -2.03532 1.39014 -1.99758

H -3.55098 0.58616 -2.47344

H -6.93996 2.04410 -0.99620

H -5.97131 1.39884 -2.36702

H -5.62154 4.06991 -0.90227

H -5.14207 4.93892 -3.19293

H -5.39339 3.30419 -3.87365

H -6.75288 4.18611 -3.11740

H -2.10976 2.86189 0.22655

H -3.67756 2.97645 1.06922

H -2.11051 1.31614 2.17695

H -3.59135 0.52694 1.57618

B 2.97354 -2.82239 3.54506

H 3.31736 -1.67269 3.06178

H 2.98486 -3.57177 2.56603

H 3.82833 -3.09867 4.39093

H 1.84629 -2.63745 4.01043

**Compound 9 radical anion**

O 1.79602 3.17072 0.94755

C 1.47988 2.10296 0.43942

C 2.50770 1.03159 0.26933

C 3.80696 1.36003 0.70110

C 4.84064 0.41731 0.58533

C 6.16305 0.75721 1.01989

N 7.23080 1.04474 1.37426

C 4.59479 -0.84440 0.05568

C 3.29699 -1.19408 -0.37626

N 3.10118 -2.46657 -0.89139

O 1.93653 -2.77829 -1.27548

O 4.06533 -3.26146 -0.96159

C 2.23763 -0.23214 -0.27115

S 0.63363 -0.69059 -0.83431

C -0.24831 0.80989 -0.52222

N -1.56031 0.76905 -0.92158

C -2.47064 1.79902 -0.45855

C -3.23119 1.30183 0.76830

C -3.95766 -0.00719 0.46441

O -4.57330 -0.54933 1.61104

C -5.87795 -0.03420 1.65764

C -6.26676 0.02819 0.17974

C -6.91452 -1.24366 -0.33582

O -5.02134 0.25424 -0.45250

C -3.00646 -1.03618 -0.12386

C -2.24368 -0.44807 -1.30793

N 0.18866 1.89677 0.02906

H 3.97127 2.35267 1.11728

H 5.37479 -1.59743 -0.04532

H -3.17761 2.02944 -1.27228

H -1.88378 2.69384 -0.22345

H -3.95926 2.05794 1.10081

H -2.51939 1.12847 1.59034

H -6.50515 -0.71525 2.25052

H -5.90182 0.96830 2.12545

H -6.91871 0.89487 -0.02625

H -6.30819 -2.12123 -0.06538

H -7.00155 -1.20829 -1.43149

H -7.92345 -1.36930 0.08844

H -3.58755 -1.91905 -0.42894

H -2.29599 -1.34679 0.65770

H -2.95127 -0.19744 -2.11549

H -1.53465 -1.17744 -1.71895

**Compound 10**

I 5.36889 1.40828 0.59433

C 3.47716 0.57705 0.23672

C 3.33537 -0.46242 -0.67173

C 2.07126 -0.99705 -0.90562

N 2.00786 -2.08073 -1.87433

O 3.04552 -2.54186 -2.30037

O 0.90468 -2.47084 -2.21253

C 0.91466 -0.52567 -0.24165

C 1.09010 0.52353 0.67976

C -0.03289 1.13324 1.46450

N -1.28031 0.61213 1.40583

C -1.66176 -0.36294 0.62780

N -2.92653 -0.79841 0.67043

C -3.51859 -1.73901 -0.27117

C -4.42055 -0.99445 -1.24717

C -5.48449 -0.18993 -0.50172

O -6.20319 0.60115 -1.43502

C -7.58008 0.29027 -1.32713

C -7.56851 -1.11608 -0.75382

C -8.78801 -1.52316 0.03130

O -6.43079 -1.04825 0.10132

C -4.85625 0.68400 0.57656

C -3.93089 -0.11868 1.48177

S -0.65825 -1.22296 -0.56973

O 0.20307 2.10857 2.17412

C 2.36006 1.06158 0.90607

H 4.18429 -0.87592 -1.21602

H -4.10184 -2.46751 0.31308

H -2.74633 -2.30803 -0.80244

H -4.91470 -1.70845 -1.92257

H -3.82095 -0.30478 -1.86156

H -8.04317 0.35445 -2.32174

H -8.08970 0.99112 -0.64025

H -7.37468 -1.84390 -1.56492

H -9.66846 -1.54550 -0.62818

H -8.65742 -2.52770 0.45891

H -8.97844 -0.81116 0.84860

H -5.65686 1.15148 1.16851

H -4.28180 1.48272 0.08243

H -3.42628 0.52428 2.20838

H -4.49206 -0.89263 2.02816

H 2.43400 1.87723 1.62689

**Compound 10 HMC**

I -5.49891 1.05527 -0.13526

C -3.52658 0.31859 -0.04595

C -2.48701 1.17676 -0.67724

C -3.26755 -0.85103 0.55625

C -1.92187 -1.37499 0.62402

N -1.73803 -2.55667 1.27276

O -0.58313 -3.05400 1.35626

O -2.71090 -3.15714 1.79067

C -0.84760 -0.62737 0.01029

C -1.10379 0.57430 -0.60884

C -0.08290 1.39782 -1.25513

O -0.41589 2.47530 -1.77742

N 1.23021 1.02101 -1.30559

C 1.70243 -0.08225 -0.80424

N 3.01454 -0.37464 -0.93720

C 3.95094 0.61074 -1.45959

C 4.76821 1.19783 -0.31459

C 5.47173 0.10804 0.48462

O 6.09989 0.64809 1.63703

C 7.50184 0.57066 1.46375

C 7.65928 -0.60155 0.51171

C 8.90786 -0.60840 -0.33134

O 6.50740 -0.43948 -0.30931

C 4.49090 -0.97889 0.91567

C 3.68501 -1.48225 -0.27605

S 0.77696 -1.31232 0.07939

H -2.74245 1.38252 -1.73344

H -2.47511 2.17817 -0.20642

H -4.05037 -1.45423 1.01473

H 4.60868 0.10127 -2.18175

H 3.39116 1.38787 -1.98822

H 4.09986 1.74603 0.36749

H 5.52162 1.90154 -0.69884

H 7.97614 0.40982 2.44199

H 7.89818 1.49936 1.01273

H 7.57507 -1.54956 1.07742

H 9.79448 -0.70622 0.31290

H 8.99329 0.32558 -0.90738

H 8.90048 -1.45670 -1.03094

H 5.05054 -1.80781 1.37445

H 3.81297 -0.56262 1.67748

H 2.96565 -2.25090 0.03043

H 4.35397 -1.95467 -1.01305

**Compound 10 TS**

I -1.74760 4.82076 -2.01292

C -0.77852 3.18413 -1.12928

C 0.55715 3.23207 -0.84924

C 1.20166 2.11903 -0.26467

N 2.59356 2.23530 -0.03894

O 3.18439 1.29123 0.48720

O 3.17542 3.26251 -0.37219

C 0.45645 0.96752 0.11841

C -0.91187 0.94187 -0.15354

C -1.81970 -0.15246 0.25405

N -1.35099 -1.25615 0.89701

C -0.11108 -1.45415 1.24186

N 0.23716 -2.58347 1.88096

C 1.60021 -2.97811 2.20471

C 2.05573 -4.10431 1.28551

C 1.08897 -5.28315 1.35334

O 1.47230 -6.25045 0.38955

C 1.60612 -7.50078 1.03836

C 1.94934 -7.10522 2.46351

C 1.59494 -8.10583 3.53244

O 1.16710 -5.92416 2.61222

C -0.34721 -4.82557 1.12943

C -0.71612 -3.67287 2.05505

S 1.25601 -0.34801 0.96105

O -3.02423 -0.06412 -0.00351

C -1.51816 1.98392 -0.92685

H 1.15726 4.12006 -1.04702

H 2.28470 -2.12354 2.14805

H 1.60580 -3.31723 3.25281

H 3.06148 -4.44398 1.57479

H 2.10298 -3.74415 0.24580

H 2.39476 -8.08115 0.53969

H 0.65697 -8.06732 1.00582

H 3.02314 -6.84292 2.52505

H 2.18356 -9.02521 3.39476

H 1.81955 -7.70438 4.53117

H 0.52574 -8.36260 3.48583

H -0.44798 -4.50242 0.08164

H -1.02300 -5.67785 1.29386

H -1.72269 -3.29975 1.84521

H -0.67293 -3.99295 3.10815

H -2.60779 2.01946 -0.89127

B -1.35762 0.78470 -3.47630

H -1.68922 1.40115 -2.37086

H -1.53342 -0.40398 -3.20497

H -2.11162 1.22673 -4.34381

H -0.17582 1.09877 -3.62205

**Compound 10 radical anion**

I -5.28129 1.18467 0.84775

C -3.38154 0.35920 0.40990

C -3.14810 -0.96949 0.70641

C -1.87877 -1.53199 0.41576

N -1.67234 -2.86694 0.72596

O -2.59717 -3.53270 1.24684

O -0.53890 -3.37049 0.46418

C -0.86279 -0.71519 -0.17399

C -1.14426 0.62649 -0.45931

C -0.16545 1.57143 -1.07295

N 1.11149 1.15810 -1.35585

C 1.55370 -0.04650 -1.18754

N 2.84593 -0.32082 -1.56673

C 3.72715 0.78532 -1.89043

C 4.51360 1.20143 -0.65038

C 5.26716 0.02112 -0.05575

O 5.88268 0.36653 1.17788

C 7.27208 0.40489 0.99202

C 7.48990 -0.55222 -0.17102

C 8.72165 -0.31073 -1.00760

O 6.32859 -0.32160 -0.93829

C 4.34770 -1.17497 0.16254

C 3.56863 -1.49098 -1.11186

S 0.70427 -1.44393 -0.51724

O -0.49910 2.72481 -1.31343

C -2.41367 1.17396 -0.16536

H -3.88931 -1.62777 1.15569

H 3.11512 1.61495 -2.26121

H 4.41593 0.45625 -2.68579

H 5.23604 1.99756 -0.88756

H 3.81411 1.58793 0.10654

H 7.76966 0.09101 1.92208

H 7.61872 1.42383 0.72648

H 7.48990 -1.59463 0.20935

H 9.63199 -0.46259 -0.40736

H 8.72356 0.71710 -1.40082

H 8.75326 -1.00502 -1.85949

H 4.95170 -2.03917 0.48022

H 3.64765 -0.93921 0.97902

H 4.26887 -1.78788 -1.90988

H 2.88465 -2.33432 -0.95543

H -2.57810 2.22238 -0.40852

**Compound 11**

Br 5.69511 1.73041 -0.39614

C 4.03970 0.86449 -0.16090

C 3.90450 -0.11664 0.80925

C 2.67292 -0.74421 0.97383

N 2.61396 -1.76166 2.01266

O 1.54866 -2.32425 2.19202

O 3.61842 -2.00429 2.64736

C 1.54445 -0.41728 0.18488

C 1.71811 0.57778 -0.79584

C 0.62744 1.03484 -1.71810

N -0.61415 0.50497 -1.63975

C -0.99422 -0.42744 -0.81045

N -2.25200 -0.88258 -0.84787

C -2.83921 -1.81344 0.10658

C -3.82161 -1.07549 1.00689

C -4.88891 -0.36433 0.18429

O -5.73208 -1.34577 -0.39792

C -7.06138 -0.87872 -0.28787

C -7.03312 -0.11768 1.02631

C -8.06119 0.97454 1.17617

O -5.71744 0.43479 1.00471

C -4.25606 0.50992 -0.89493

C -3.24917 -0.27583 -1.72387

S 0.00803 -1.22241 0.43190

O 0.88365 1.90421 -2.54845

C 2.95498 1.20586 -0.95669

H 4.73573 -0.41070 1.44956

H -2.06551 -2.31607 0.69873

H -3.35494 -2.59711 -0.47026

H -4.30954 -1.77987 1.69621

H -3.28178 -0.32381 1.60336

H -7.74305 -1.73981 -0.28706

H -7.32038 -0.20700 -1.12781

H -7.10931 -0.83293 1.86722

H -7.98442 1.69579 0.34825

H -7.92568 1.51077 2.12659

H -9.07249 0.54029 1.17224

H -3.75749 1.36398 -0.41027

H -5.04918 0.90747 -1.54582

H -3.75125 -1.09236 -2.26630

H -2.74064 0.36475 -2.44961

H 3.03531 1.97187 -1.72927

**Compound 11 HMC**

Br -5.74575 -1.59598 0.06109

C -4.04636 -0.75609 0.13940

C -3.04999 -1.24206 -0.85017

C -3.80827 0.20518 1.04230

C -2.51887 0.85393 1.11468

N -2.35163 1.82051 2.05749

O -1.24802 2.42177 2.15254

O -3.29077 2.12079 2.83434

C -1.48000 0.44779 0.19550

C -1.72123 -0.53526 -0.73689

C -0.73656 -1.00399 -1.70975

O -1.06678 -1.87647 -2.52999

N 0.54222 -0.52078 -1.74117

C 0.99762 0.40834 -0.95419

N 2.27577 0.83074 -1.07956

C 3.21013 0.11014 -1.93317

C 4.08607 -0.80565 -1.08590

C 4.79567 -0.02686 0.01759

O 5.47306 -0.94003 0.86586

C 6.81945 -0.52297 0.98686

C 7.06110 0.21173 -0.31929

C 8.14718 1.25548 -0.30345

O 5.79085 0.82114 -0.52725

C 3.81282 0.82602 0.81311

C 2.95464 1.68028 -0.11314

S 0.08788 1.24674 0.31874

H -3.44199 -1.12947 -1.87841

H -2.89797 -2.33255 -0.73928

H -4.57012 0.53179 1.74891

H 3.82916 0.85631 -2.45544

H 2.64774 -0.45831 -2.67981

H 3.46479 -1.58755 -0.62127

H 4.84133 -1.30186 -1.71416

H 7.45836 -1.40694 1.12008

H 6.94909 0.15596 1.85035

H 7.25056 -0.52359 -1.12499

H 7.96095 2.00046 0.48512

H 8.20428 1.77256 -1.27221

H 9.12109 0.77954 -0.11420

H 3.16502 0.15256 1.39581

H 4.37293 1.46023 1.51604

H 3.58352 2.38842 -0.67514

H 2.23657 2.28325 0.45565

**Compound 11 TS**

Br 0.97692 -3.11646 4.72076

C 0.55896 -1.76788 3.47035

C 1.06783 -0.50920 3.60903

C 0.75385 0.48811 2.65955

N 1.27642 1.78176 2.89750

O 1.02871 2.67388 2.08552

O 1.95105 1.98328 3.90134

C -0.00977 0.17189 1.50060

C -0.51782 -1.12188 1.37336

C -1.30183 -1.59487 0.21273

N -1.49469 -0.80474 -0.87854

C -1.10218 0.43155 -0.98580

N -1.35387 1.12294 -2.11039

C -0.79663 2.43099 -2.42478

C 0.34600 2.27382 -3.41989

C -0.11998 1.54008 -4.67561

O -1.00096 2.39684 -5.39713

C -0.51617 2.52461 -6.71950

C 0.96999 2.25305 -6.56480

C 1.68105 1.73498 -7.78736

O 0.95698 1.26273 -5.53970

C -0.82400 0.23657 -4.32178

C -1.91547 0.46228 -3.28270

S -0.24097 1.38149 0.25085

O -1.78008 -2.73288 0.23308

C -0.37258 -2.06750 2.43769

H 1.73176 -0.24598 4.43211

H -0.46110 2.94816 -1.51810

H -1.60470 3.04233 -2.85544

H 0.74455 3.25984 -3.70043

H 1.15810 1.69132 -2.95717

H -0.74277 3.53404 -7.09054

H -0.98388 1.77603 -7.38582

H 1.47424 3.16298 -6.18661

H 1.19399 0.82304 -8.16507

H 2.73217 1.50662 -7.55897

H 1.66305 2.49708 -8.58089

H -0.07799 -0.47273 -3.93024

H -1.25584 -0.19225 -5.23840

H -2.70172 1.11788 -3.68841

H -2.37312 -0.48206 -2.97416

H -0.57408 -3.10812 2.18045

B -2.82067 -1.67588 3.82250

H -1.75672 -2.08645 3.18057

H -2.35503 -1.13146 4.82461

H -3.34068 -0.89788 3.02112

H -3.48433 -2.68677 4.05514

**Compound 11 radical anion**

Br 5.81601 -0.86633 -0.76064

C 4.04641 -0.33889 -0.29370

C 3.80655 0.12709 0.98381

C 2.49330 0.52262 1.34085

N 2.27887 0.97852 2.63226

O 3.23608 1.02900 3.43949

O 1.10446 1.33290 2.95145

C 1.44328 0.43818 0.37271

C 1.73168 -0.04124 -0.91081

C 0.71636 -0.18146 -1.99623

N -0.58741 0.18368 -1.77773

C -1.04197 0.67642 -0.67019

N -2.36659 1.03767 -0.61838

C -3.06083 1.33447 0.61690

C -3.76507 0.08967 1.15014

C -4.69853 -0.48810 0.09260

O -5.24429 -1.71167 0.56961

C -6.64341 -1.63765 0.50420

C -6.91230 -0.14253 0.58988

C -8.19993 0.34132 -0.02833

O -5.80795 0.37466 -0.11979

C -3.97831 -0.68459 -1.23277

C -3.26107 0.59077 -1.66911

S -0.17544 0.95032 0.84498

O 1.04965 -0.62594 -3.08778

C 3.04395 -0.43833 -1.25056

H 4.57938 0.20955 1.74559

H -3.80708 2.11837 0.40522

H -2.36810 1.74540 1.36190

H -3.01932 -0.67373 1.42025

H -4.34596 0.32434 2.05567

H -7.02688 -2.04713 -0.45182

H -7.07537 -2.21350 1.33671

H -6.86081 0.17626 1.65153

H -9.06614 -0.07947 0.50506

H -8.26495 1.43759 0.02327

H -8.25550 0.04210 -1.08594

H -3.23872 -1.49060 -1.11010

H -4.71136 -1.00471 -1.98917

H -3.99411 1.39065 -1.86536

H -2.66640 0.41683 -2.57295

H 3.22017 -0.80804 -2.25921

**Compound 23**

O 2.26181 -3.07952 -1.46244

C 1.97591 -2.04869 -0.85541

C 3.05233 -1.04613 -0.58367

C 4.36868 -1.44953 -0.83497

C 5.44577 -0.59541 -0.63896

C 5.20719 0.69677 -0.19426

C 3.90181 1.11690 0.05309

N 3.73725 2.48757 0.50061

O 4.72365 3.12007 0.81780

O 2.60635 2.94260 0.53030

C 2.79148 0.25685 -0.12152

S 1.16459 0.79973 0.25119

C 0.26837 -0.73600 0.11815

N -0.98789 -0.67003 0.57627

C -1.92400 -1.76991 0.36673

C -2.97926 -1.36214 -0.65357

C -3.68662 -0.07612 -0.23419

O -4.56508 0.33348 -1.27027

C -5.87322 0.44211 -0.74021

C -5.83325 -0.48487 0.46236

C -6.80103 -0.17218 1.57359

O -4.49281 -0.29455 0.90527

C -2.67828 1.01976 0.08520

C -1.63753 0.53052 1.08357

N 0.71375 -1.84194 -0.41077

H 4.52004 -2.46758 -1.19744

H 6.46571 -0.92860 -0.83748

H 6.02199 1.40347 -0.03649

H -1.36681 -2.65104 0.03681

H -2.39312 -1.99560 1.33728

H -2.50742 -1.20099 -1.63546

H -3.72312 -2.16542 -0.76349

H -6.59955 0.13562 -1.50605

H -6.08626 1.48153 -0.42929

H -5.96283 -1.53182 0.12662

H -7.83432 -0.29417 1.21571

H -6.67027 0.86320 1.92306

H -6.65210 -0.85507 2.42233

H -2.18199 1.31853 -0.85100

H -3.21137 1.89278 0.48898

H -0.90109 1.31306 1.30154

H -2.11530 0.26981 2.04126

**Compound 23 HMC**

O -2.48201 -2.70559 -1.81341

C -2.08836 -1.65942 -1.26791

C -3.03220 -0.86217 -0.48732

C -4.42389 -1.44029 -0.43730

C -5.39959 -0.62010 0.33198

C -5.05480 0.52491 0.94151

C -3.70354 1.04572 0.89590

N -3.43903 2.20020 1.55642

O -2.27081 2.68361 1.54635

O -4.34883 2.80126 2.18572

C -2.70269 0.31236 0.15258

S -1.06815 0.97934 0.09524

C -0.24634 -0.22166 -0.91952

N 1.05498 0.05922 -1.16060

C 1.92585 -0.93185 -1.77778

C 2.77758 -1.60278 -0.70650

C 3.56551 -0.57000 0.09951

O 4.24741 -1.16755 1.17781

C 5.54832 -1.48394 0.71953

C 5.86154 -0.32021 -0.21502

C 6.48833 0.87556 0.47402

O 4.57030 -0.00094 -0.73191

C 2.64898 0.52267 0.63228

C 1.80282 1.11059 -0.49110

N -0.77845 -1.29581 -1.42228

H -4.78601 -1.60073 -1.47080

H -4.37533 -2.46713 -0.02536

H -6.43158 -0.97764 0.39302

H -5.78548 1.10892 1.50095

H 1.31146 -1.66181 -2.31309

H 2.56543 -0.41054 -2.50740

H 3.47533 -2.31696 -1.16846

H 2.12456 -2.15809 -0.01489

H 6.22123 -1.54836 1.58501

H 5.55596 -2.44834 0.18138

H 6.48892 -0.64642 -1.05975

H 5.90069 1.17414 1.35551

H 7.51130 0.63318 0.80101

H 6.54181 1.73074 -0.21600

H 3.26440 1.30497 1.09987

H 1.99321 0.08934 1.40399

H 1.13182 1.89088 -0.11255

H 2.44996 1.59011 -1.24232

**Compound 23 TS**

O 2.32002 2.91892 1.33048

C 1.36335 2.14273 1.22851

C 0.01024 2.61106 1.58549

C -0.09266 3.99340 1.96427

C -1.30015 4.44783 2.57694

C -2.41634 3.66629 2.52359

C -2.37713 2.37372 1.94315

N -3.58824 1.65792 1.87895

O -4.62248 2.17637 2.29551

O -3.58215 0.52107 1.39658

C -1.13799 1.82158 1.50492

S -1.08080 0.16187 0.93078

C 0.66122 -0.03158 0.62079

N 0.99566 -1.25712 0.17917

C 2.34434 -1.54005 -0.29548

C 2.34610 -1.64397 -1.81616

C 1.33802 -2.68220 -2.29734

O 1.28150 -2.65438 -3.71323

C 1.43307 -3.97812 -4.18947

C 2.27142 -4.62874 -3.10378

C 2.14595 -6.12454 -2.97511

O 1.75610 -3.98738 -1.94118

C -0.03830 -2.43221 -1.69033

C 0.04975 -2.30470 -0.17491

N 1.58143 0.87518 0.77952

H 0.84869 4.47370 2.23556

H -1.33707 5.43837 3.03390

H -3.36369 4.00553 2.94289

H 3.01597 -0.74974 0.05159

H 2.66389 -2.49337 0.15422

H 2.08674 -0.66832 -2.25657

H 3.34772 -1.92457 -2.17552

H 1.92512 -3.95074 -5.17143

H 0.45292 -4.48174 -4.28360

H 3.33280 -4.34380 -3.23786

H 2.52564 -6.61286 -3.88510

H 1.09413 -6.41584 -2.83237

H 2.73382 -6.49212 -2.12164

H -0.44224 -1.50030 -2.11564

H -0.70909 -3.25893 -1.96655

H -0.93716 -2.11115 0.26207

H 0.41194 -3.24490 0.27024

B -0.18411 5.38482 -0.50072

H -0.02399 4.74794 0.63644

H 0.04791 6.56307 -0.22935

H -1.36162 5.14775 -0.77210

H 0.63112 4.86341 -1.26123

**Compound 23 radical anion**

O -1.84653 -3.23092 1.51503

C -1.69745 -2.19410 0.87816

C -2.78643 -1.17582 0.84982

C -3.95873 -1.50030 1.56536

C -5.02560 -0.60532 1.60905

C -4.94946 0.61466 0.95908

C -3.78622 0.97059 0.23318

N -3.74943 2.20497 -0.39550

O -4.73744 2.97613 -0.31336

O -2.70075 2.51613 -1.04194

C -2.69113 0.05189 0.17817

S -1.26206 0.50874 -0.74983

C -0.26276 -0.92592 -0.50641

N 0.94473 -0.87456 -1.16630

C 1.97225 -1.83555 -0.81505

C 2.89597 -1.25151 0.25076

C 3.47739 0.08188 -0.19839

O 4.40408 -0.11502 -1.26876

C 5.69846 0.05721 -0.75984

C 5.48609 1.05046 0.37263

C 6.50948 1.02529 1.48066

O 4.22668 0.64447 0.86368

C 2.38877 1.03223 -0.67759

C 1.49342 0.35058 -1.70941

N -0.52361 -1.97894 0.20036

H -3.98186 -2.46535 2.07130

H -5.93220 -0.86413 2.16352

H -5.75990 1.34253 0.97287

H 1.48066 -2.74436 -0.45010

H 2.54741 -2.07319 -1.72525

H 3.72531 -1.94034 0.47651

H 2.32734 -1.09452 1.18029

H 6.11435 -0.89341 -0.36838

H 6.35376 0.42920 -1.56176

H 5.41364 2.07405 -0.04952

H 6.58562 0.01579 1.91216

H 6.22511 1.72079 2.28313

H 7.49813 1.32541 1.09989

H 1.78153 1.33338 0.18995

H 2.85874 1.93550 -1.09609

H 2.08417 0.09323 -2.60417

H 0.69031 1.02204 -2.03783
