# Supplementary material for: Modulation of the Meisenheimer complex metabolism of nitro-benzothiazinones by targeted C-6 substitution
Source: Commun Chem. 2024 Jul 6;7:153. doi: 10.1038/s42004-024-01235-x (PMC11227536; doi:10.1038/s42004-024-01235-x)
Supplement: Supplementary file 9 — Reporting Summary [file 42004_2024_1235_MOESM9_ESM.pdf]

nature portfolio

Corresponding author(s): Florian Kloss

Last updated by author(s): 2024/06/20

Reporting Summary

Nature Portfolio wishes to improve the reproducibility of the work that we publish. This form provides structure for consistency and transparency in reporting. For further information on Nature Portfolio policies, see our Editorial Policies and the Editorial Policy Checklist.

Statistics

For all statistical analyses, confirm that the following items are present in the figure legend, table legend, main text, or Methods section.

n/a	Confirmed	
☐	☒	The exact sample size (n) for each experimental group/condition, given as a discrete number and unit of measurement
☐	☒	A statement on whether measurements were taken from distinct samples or whether the same sample was measured repeatedly
☒	☐	The statistical test(s) used AND whether they are one- or two-sided<br>*Only common tests should be described solely by name; describe more complex techniques in the Methods section.*
☒	☐	A description of all covariates tested
☒	☐	A description of any assumptions or corrections, such as tests of normality and adjustment for multiple comparisons
☐	☒	A full description of the statistical parameters including central tendency (e.g. means) or other basic estimates (e.g. regression coefficient) AND variation (e.g. standard deviation) or associated estimates of uncertainty (e.g. confidence intervals)
☐	☒	For null hypothesis testing, the test statistic (e.g. F, t, r) with confidence intervals, effect sizes, degrees of freedom and P value noted<br>*Give P values as exact values whenever suitable.*
☒	☐	For Bayesian analysis, information on the choice of priors and Markov chain Monte Carlo settings
☒	☐	For hierarchical and complex designs, identification of the appropriate level for tests and full reporting of outcomes
☒	☐	Estimates of effect sizes (e.g. Cohen's d, Pearson's r), indicating how they were calculated

Our web collection on statistics for biologists contains articles on many of the points above.

Software and code

Policy information about availability of computer code

Data collection	NMR measurements were performed on a Bruker AVANCE II 300 MHz, Bruker AVANCE III 500 MHz and a Bruker AVANCE III 600 MHz spectrometer, equipped with a Bruker Cryoplatform.<br>UHPLC measurements were performed on a Thermo Vanquish Horizon system, equipped with a 60 mm Lightpipe diode array detector, using a Phenomenex Kinetex C8 column (100 x 2.1 mm, particle size 1.7 µm, pore diameter 100 Å).<br>HPLC-HRMS measurements were performed on a Thermo Vanquish Horizon UHPLC system coupled to a Thermo QExactive HF-X Orbitrap benchtop HRMS, using a Phenomenex Kinetex C8 column (100 x 2.1 mm, particle size 1.7 µm, pore diameter 100 Å).<br>X-Ray crystallography: The single-crystal X-ray intensity data were collected on a Bruker-Nonius Kappa-CCD diffractometer equipped with a Mo-Kα IµS microfocus source and an Apex2 CCD detector, at T = 120(2) K.<br>Proliferation and cytotoxicity assay: Plates were measured on a SUNRISE microplate reader (TECAN).<br>Computational modeling was used as part of our strategic data collection. Quantum chemical calculations at density functional theory were performed using the Schrödinger Suite (version 2022-2), which includes MacroModel (version 13.1) and Jaguar (version 11.1). Additionally, the open-source Python package AutodE (version 1.2.3) linked to ORCA (version 5.0.3) was also employed. Covalent docking simulations were performed using also the Schrödinger Suite. All details are given in the Supplementary Information.
Data analysis	The authors declare that neither specific software nor custom algorithms were used for data analysis.<br>Raw NMR data (FID files) were processed with MestReNova (Version: 14.2.1-27684).<br>UHPLC measurements were analyzed on Thermo Scienfitic Dionex Chromeleon 7 Chromatography Data (Version: 7.2 SR5 MUd)<br>HPLC-HRMS measurements were analyzed and processed with the software XCalibur 4.1 (Thermo Fisher, USA).<br>X-Ray crystallography: The structures were solved using Intrinsic-phasing algorithms with SHELXT-2018/3 and refined by full matrix least-squares methods on F2 with SHELXL-2018/3, using the Olex 1.2 environment. Multi-scan absorption correction was applied to the intensity data.

Proliferation and cytotoxicity assay: Calculations of the different values of GI50 and CC50 were performed with the software Magella (TECAN).
Calculation of conceptual density functional theory descriptors was performed in Python following well-known definitions as described in the Supplementary Information. Python (version 3.9) scripts were used for data analysis.
Code used for data analysis in this study is publicly available at https://github.com/BernalFA/HMC-carboxy-BTZs.

For manuscripts utilizing custom algorithms or software that are central to the research but not yet described in published literature, software must be made available to editors and reviewers. We strongly encourage code deposition in a community repository (e.g. GitHub). See the Nature Portfolio guidelines for submitting code & software for further information.

Data

Policy information about availability of data

All manuscripts must include a data availability statement. This statement should provide the following information, where applicable:

- Accession codes, unique identifiers, or web links for publicly available datasets
- A description of any restrictions on data availability
- For clinical datasets or third party data, please ensure that the statement adheres to our policy

The authors declare that the data supporting the findings of this study are available within the article and Supplementary Information. All cited Supplementary Tables and Supplementary Figures are grouped in the Supplementary Information under the subsections Supplementary Note 1 and 2 respectively. For experimental details and compound characterization, see Supplementary Methods 1-6. For NMR Spectra, see Supplementary Data 1. For X-Ray crystallography, see Supplementary Data 2-4. The full crystallographic data can be obtained free of charge from the Cambridge Crystallographic Data Centre with the accession codes CDCC #2278946 (22), #2278947 (S7), #2278948 (S5). For cartesian coordinates of all modelled compounds, see Supplementary Data 5. For the complete computational modeling data, see Supplementary Data 6.

Research involving human participants, their data, or biological material

Policy information about studies with human participants or human data. See also policy information about sex, gender (identity/presentation), and sexual orientation and race, ethnicity and racism.

Reporting on sex and gender	n/a
Reporting on race, ethnicity, or other socially relevant groupings	n/a
Population characteristics	n/a
Recruitment	n/a
Ethics oversight	n/a

Note that full information on the approval of the study protocol must also be provided in the manuscript.

Field-specific reporting

Please select the one below that is the best fit for your research. If you are not sure, read the appropriate sections before making your selection.

☒ Life sciences ☐ Behavioural & social sciences ☐ Ecological, evolutionary & environmental sciences

For a reference copy of the document with all sections, see nature.com/documents/nr-reporting-summary-flat.pdf

Life sciences study design

All studies must disclose on these points even when the disclosure is negative.

Sample size	MIC determination and Microsomal stability assays were perfomed with one replicate. Proliferation and cytotoxicity assays were perfomed with one biological replicate and the assays were repeated 4 times. Hydride Meisenheimer propensity assay was performed with three biological replicates and the assay was repeated 3 times.
Data exclusions	Data exclusion was only conducted on the Hydride Meisenheimer Complex formation propensity assay. HMC formation of BTZ-043 served as reference and quality control. Each biological replicate series and series with correlation ("BTZ-043"-"HMC"/"BTZ-043") smaller than 1/25 in the controls were discarded. For more details, see Joch, M et al. "Whole cell hydride Meisenheimer complex biotransformation guided optimization of antimycobacterial benzothiazinones." Eur J Med Chem 264, 116023 (2024).
Replication	1-4 technical replicates per biological assay
Randomization	n/a

Blinding | n/a

Reporting for specific materials, systems and methods

We require information from authors about some types of materials, experimental systems and methods used in many studies. Here, indicate whether each material, system or method listed is relevant to your study. If you are not sure if a list item applies to your research, read the appropriate section before selecting a response.

Materials & experimental systems

n/a	Involved in the study
☒	☐ Antibodies
☐	☒ Eukaryotic cell lines
☒	☐ Palaeontology and archaeology
☒	☐ Animals and other organisms
☒	☐ Clinical data
☒	☐ Dual use research of concern
☒	☐ Plants

Methods

n/a	Involved in the study
☒	☐ ChIP-seq
☒	☐ Flow cytometry
☒	☐ MRI-based neuroimaging

Eukaryotic cell lines

Policy information about cell lines and Sex and Gender in Research

Cell line source(s)	HUVEC (ATCC CRL-1730), K-562 (DSM ACC 10), HeLa (DSM ACC 57), RAW 264.7 (ECACC 91062702)
Authentication	None of the cell lines used were authenticated.
Mycoplasma contamination	Mycoplasma contamination were assessed regularly with the MycoStrip(TM)-Mycoplasma Detection Kit of InvivoGen. All cell lines, at the exception of HUVEC, were tested free of mycoplasma.
Commonly misidentified lines (See ICLAC register)	n/a

Plants

Seed stocks	n/a
Novel plant genotypes	n/a
Authentication	n/a
